# Genomic meta-analyses of binge-eating behavior and anorexia nervosa yield insights into the unique and shared biology of eating disorder phenotypes

**DOI:** 10.1038/s44220-026-00698-2

**Published:** 2026-08-19

**Authors:** Jet D. Termorshuizen, Helena L. Davies, Sang-Hyuck Lee, Jessica K. Dennis, Christopher Hübel, Jessica S. Johnson, Yi Lu, Melissa A. Munn-Chernoff, Triinu Peters, Baiyu Qi, Katherine E. Schaumberg, Rebecca H. Signer, Karanvir Singh, Abigail R. ter Kuile, Laura M. Thornton, Jiayi Xu, Shuyang Yao, Zeynep Yilmaz, Ruyue Zhang, Johan Zvrskovec, Mohamed Abdulkadir, Ziada Ayorech, Elizabeth C. Corfield, Alexandra Havdahl, Kristi Krebs, Taralynn M. Mack, Maria Niarchou, Teemu Palviainen, Julia M. Sealock, Jessica H. Baker, Andrew W. Bergen, Andreas Birgegård, Vesna Boraska Perica, Katharina Bühren, Roland Burghardt, Matteo Cassina, Giovanni Castellini, Enrico Collantoni, James J. Crowley, Unna N. Danner, Franziska Degenhardt, Janiece E. DeSocio, Christian Dina, Monika Dmitrzak-Węglarz, Laramie E. Duncan, Karin M. Egberts, Lenka Foretova, Ina Giegling, Fragiskos Gonidakis, Scott D. Gordon, Jakob Grove, Sébastien Guillaume, Jerry D. Guintivano, Annette M. Hartmann, Konstantinos Hatzikotoulas, Stefan Herms, Hartmut Imgart, Susana Jiménez-Murcia, Antonio Julià, Gursharan Kalsi, Deborah Kaminská, Leila J. Karhunen, Hannah L. Kennedy, Kirsty M. Kiezebrink, Theresa Kolb, Janne T. Larsen, Dong Li, Lisa Lilenfeld, Mario Maj, Morten Mattingsdal, Paolo Meneguzzo, Allison L. Miller, Karen S. Mitchell, Alessio Maria Monteleone, Catherine M. Olsen, Leonid Padyukov, Richard Parker, Michaela A. Pettie, Dalila Pinto, Anu Raevuori, Samuli Ripatti, Marion E. Roberts, Paolo Santonastaso, Androula Savva, Ulrike H. Schmidt, Alexandra Schosser, Jochen Seitz, Lenka LS Slachtova, Agnieszka Slopien, Sandro Sorbi, Peter S. Straub, Jin P. Szatkiewicz, Friederike I. Tam, Elena Tenconi, Alfonso Tortorella, Artemis Tsitsika, Annemarie A. van Elburg, Gudrun Wagner, Hunna J. Watson, Roger AH Adan, Lars Alfredsson, Ole A. Andreassen, Helga Ask, Anders D. Børglum, Harry A. Brandt, David Collier, Steven Crawford, Scott Crow, Lea K. Davis, Martina de Zwaan, George Dedoussis, Danielle M. Dick, Stefan Ehrlich, Xavier Estivill, Angela Favaro, Fernando Fernández-Aranda, Krista Fischer, Andreas J. Forstner, Philip Gorwood, Hakon Hakonarson, Johannes Hebebrand, Beate Herpertz-Dahlmann, Anke Hinney, James I. Hudson, Craig Johnson, Jennifer Jordan, Allan S. Kaplan, Jaakko Kaprio, Andreas FK Karwautz, Martien JH Kas, Walter H. Kaye, James L. Kennedy, Martin A. Kennedy, Anna Keski-Rahkonen, Youl-Ri Kim, Kelly L. Klump, Mikael Landén, Stéphanie Le Hellard, Kelli Lehto, Qingqin S. Li, Jolanta Lissowska, Jurjen J. Luykx, Sarah L. Maguire, Nicholas G. Martin, Manuel Mattheisen, Sarah E. Medland, Philip Mehler, Nadia Micali, James E. Mitchell, Palmiero Monteleone, Preben Bo Mortensen, Benedetta Nacmias, Roel A. Ophoff, Hana Papezova, Nancy L. Pedersen, Liselotte V. Petersen, Luisa S. Rajcsanyi, Nicolas Ramoz, Ted Reichborn-Kjennerud, Valdo Ricca, Stephan Ripke, Dan Rujescu, Filip Rybakowski, Stephen W. Scherer, Margarita CT Slof-Op ‘t Landt, Howard Steiger, Patrick F. Sullivan, Beata Świątkowska, Eric F. van Furth, Tracey D. Wade, Thomas Werge, David C. Whiteman, D. Blake Woodside, Ya-Ke Wu, Stephan Zipfel, Disorders Working Group of the Psychiatric Genomics Consortium, Jet D. Termorshuizen, Helena L. Davies, Sang-Hyuck Lee, Jessica K. Dennis, Christopher Hübel, Jessica S. Johnson, Yi Lu, Melissa A. Munn-Chernoff, Triinu Peters, Baiyu Qi, Katherine E. Schaumberg, Rebecca H. Signer, Karanvir Singh, Laura M. Thornton, Jiayi Xu, Shuyang Yao, Zeynep Yilmaz, Ruyue Zhang, Johan Zvrskovec, Mohamed Abdulkadir, Ziada Ayorech, Elizabeth C. Corfield, Alexandra Havdahl, Kristi Krebs, Taralynn M. Mack, Maria Niarchou, Teemu Palviainen, Julia M. Sealock, Jessica H. Baker, Andrew W. Bergen, Andreas Birgegård, Vesna Boraska Perica, Katharina Bühren, Roland Burghardt, Matteo Cassina, Giovanni Castellini, Enrico Collantoni, James J. Crowley, Unna N. Danner, Franziska Degenhardt, Janiece E. DeSocio, Christian Dina, Monika Dmitrzak-Węglarz, Laramie E. Duncan, Karin M. Egberts, Lenka Foretova, Ina Giegling, Fragiskos Gonidakis, Scott D. Gordon, Jakob Grove, Sébastien Guillaume, Jerry D. Guintivano, Annette M. Hartmann, Konstantinos Hatzikotoulas, Stefan Herms, Hartmut Imgart, Susana Jiménez-Murcia, Antonio Julià, Gursharan Kalsi, Deborah Kaminská, Leila J. Karhunen, Hannah L. Kennedy, Kirsty M. Kiezebrink, Theresa Kolb, Janne T. Larsen, Dong Li, Lisa Lilenfeld, Mario Maj, Morten Mattingsdal, Paolo Meneguzzo, Allison L. Miller, Karen S. Mitchell, Alessio Maria Monteleone, Catherine M. Olsen, Leonid Padyukov, Richard Parker, Michaela A. Pettie, Dalila Pinto, Anu Raevuori, Samuli Ripatti, Marion E. Roberts, Paolo Santonastaso, Androula Savva, Ulrike H. Schmidt, Alexandra Schosser, Jochen Seitz, Lenka LS Slachtova, Agnieszka Slopien, Sandro Sorbi, Peter S. Straub, Jin P. Szatkiewicz, Friederike I. Tam, Alfonso Tortorella, Artemis Tsitsika, Gudrun Wagner, Hunna J. Watson, Roger AH Adan, Lars Alfredsson, Ole A. Andreassen, Helga Ask, Anders D. Børglum, Harry A. Brandt, David Collier, Steven Crawford, Scott Crow, Lea K. Davis, George Dedoussis, Danielle M. Dick, Stefan Ehrlich, Xavier Estivill, Angela Favaro, Fernando Fernández-Aranda, Krista Fischer, Andreas J. Forstner, Philip Gorwood, Hakon Hakonarson, Johannes Hebebrand, Beate Herpertz-Dahlmann, Anke Hinney, James I. Hudson, Craig Johnson, Jennifer Jordan, Allan S. Kaplan, Jaakko Kaprio, Andreas FK Karwautz, Martien JH Kas, Walter H. Kaye, James L. Kennedy, Martin A. Kennedy, Anna Keski-Rahkonen, Youl-Ri Kim, Kelly L. Klump, Mikael Landén, Kelli Lehto, Qingqin S. Li, Jolanta Lissowska, Jurjen J. Luykx, Sarah L. Maguire, Nicholas G. Martin, Manuel Mattheisen, Sarah E. Medland, Philip Mehler, Nadia Micali, James E. Mitchell, Palmiero Monteleone, Preben Bo Mortensen, Benedetta Nacmias, Roel A. Ophoff, Hana Papezova, Nancy L. Pedersen, Liselotte V. Petersen, Luisa S. Rajcsanyi, Nicolas Ramoz, Ted Reichborn-Kjennerud, Valdo Ricca, Stephan Ripke, Dan Rujescu, Filip Rybakowski, Stephen W. Scherer, Margarita CT Slof-Op ‘t Landt, Howard Steiger, Patrick F. Sullivan, Beata Świątkowska, Tracey D. Wade, Thomas Werge, David C. Whiteman, D. Blake Woodside, Ya-Ke Wu, Stephan Zipfel, Abigail R. ter Kuile, Elena Tenconi, Annemarie A. van Elburg, Martina de Zwaan, Stéphanie Le Hellard, Eric F. van Furth, Cynthia M. Bulik, Laura M. Huckins, Gerome Breen, Jonathan R.I. Coleman, Estonian Biobank (EstBB), Kristi Krebs, Kelli Lehto, Krista Fischer, Cynthia M. Bulik, Laura M. Huckins, Gerome Breen, Jonathan R.I. Coleman

**Affiliations:** 1 Department of Medical Epidemiology and Biostatistics, Karolinska Institutet, Stockholm, Sweden; 2 Institute of Psychiatry, Psychology and Neuroscience, Social, Genetic and Developmental Psychiatry Centre, King’s College London, London, UK; 3 Center for Eating and Feeding Disorders Research, Mental Health Center Ballerup, Copenhagen University Hospital - Mental Health Services, Copenhagen, Denmark; 4 Institute of Biological Psychiatry, Mental Health Center Sct. Hans, Mental Health Services Copenhagen, Roskilde, Denmark; 5 National Institute for Health Research Biomedical Research Centre, King’s College London and South London and Maudsley National Health Service Trust, London, UK; 6 Department of Medical Genetics, University of British Columbia, Vancouver, British Columbia Canada; 7 Graduate Program in Bioinformatics, University of British Columbia, Vancouver, British Columbia Canada; 8 National Centre for Register-Based Research, Aarhus University, Aarhus, Denmark; 9 Clinic for Child and Adolescent Psychiatry, Psychotherapy and Psychosomatics, German Red Cross Hospitals Berlin, Berlin, Germany; 10 Department of Psychiatry, University of North Carolina at Chapel Hill, Chapel Hill, NC USA; 11 Department of Community, Family and Addiction Sciences, Texas Tech University, Lubbock, TX USA; 12 Section of Molecular Genetics in Mental Disorders, LVR University Hospital Essen, University of Duisburg-Essen, Essen, Germany; 13 Institute of Sex and Gender-Sensitive Medicine, University Hospital Essen, University of Duisburg-Essen, Essen, Germany; 14 Center for Translational Neuro- and Behavioral Sciences, University Hospital Essen, University of Duisburg-Essen, Essen, Germany; 15 Department of Epidemiology, University of North Carolina at Chapel Hill, Chapel Hill, NC USA; 16 Department of Psychiatry, University of Wisconsin, Madison, WI USA; 17 Department of Psychology, University of Texas, Austin, TX USA; 18 Department of Genetics and Genomic Sciences, Icahn School of Medicine at Mount Sinai, New York, NY USA; 19 Department of Psychiatry, Yale University, New Haven, CT USA; 20 Department of Clinical, Educational and Health Psychology, University College London, London, UK; 21 Department of Biomedicine, Aarhus University, Aarhus, Denmark; 22 Department of Genetics, University of North Carolina at Chapel Hill, Chapel Hill, NC USA; 23 Department of Psychology, PROMENTA Research Centre, University of Oslo, Oslo, Norway; 24 PsychGen Centre for Genetic Epidemiology and Mental Health, Norwegian Institute of Public Health, Oslo, Norway; 25 Psychiatric Genetic Epidemiology Group, Research Department, Lovisenberg Diakonale Hospital, Oslo, Norway; 26 MRC Integrative Epidemiology Unit, Population Health Sciences, Bristol Medical School, University of Bristol, Bristol, UK; 27 Estonian Genome Centre, Institute of Genomics, University of Tartu, Tartu, Estonia; 28 Vanderbilt Genetics Institute, Vanderbilt University, Nashville, TN USA; 29 The Weindrich Department of AI and Human Health, Icahn School of Medicine at Mount Sinai, New York, NY USA; 30 Department of Genetic Medicine, Vanderbilt University Medical Center, Nashville, TN USA; 31 Institute for Molecular Medicine Finland, Helsinki Institute of Life Science, University of Helsinki, Helsinki, Finland; 32 Analytic and Translational Genetics Unit, Broad Institute of the Massachusetts Institute of Technology and Harvard University, Massachusetts General Hospital, Boston, MA USA; 33 Stanley Center for Psychiatric Research, Broad Institute of the Massachusetts Institute of Technology and Harvard University, Cambridge, MA USA; 34 Independent Researcher, Mebane, NC USA; 35 Oregon Research Institute, Springfield, OR USA; 36 Department of Medicine, New Jersey Medical School, Rutgers University, Newark, NJ USA; 37 Department for Medical Biology, University of Split School of Medicine, Split, Croatia; 38 Department of Child and Adolescent Psychiatry, Psychosomatics and Psychotherapy, Ludwig-Maximilians-Universität München, Munich, Germany; 39 Department of Child and Adolescent Psychiatry, Oberberg Fachklinik Fasanenkiez Berlin, Berlin, Germany; 40 Department of Women’s and Children’s Health, University of Padova, Padova, Italy; 41 Department of Health Sciences, University of Florence, Florence, Italy; 42 Department of Neuroscience, University of Padova, Padova, Italy; 43 Department of Clinical Neuroscience, Karolinska Institutet, Stockholm, Sweden; 44 Altrecht Eating Disorders Rintveld, Altrecht Mental Health Institute, Utrecht, the Netherlands; 45 Department of Child and Adolescent Psychiatry, Psychosomatics and Psychotherapy, LVR University Hospital Essen, University of Duisburg-Essen, Essen, Germany; 46 College of Nursing, Seattle University, Seattle, WA USA; 47 Nantes Université, CNRS, INSERM, L’Institut du Thorax, Nantes, France; 48 Department of Psychiatric Genetics, Medical Biology Center, Poznan University of Medical Sciences, Poznan, Poland; 49 Department of Psychiatry and Behavioral Sciences, Stanford University, Stanford, CA USA; 50 Department of Child and Adolescent Psychiatry, Psychosomatics and Psychotherapy, Center of Mental Health, University Hospital Wuerzburg, Würzburg, Germany; 51 Department of Child and Adolescent Psychiatry Herlaarhof, Reinier van Arkel, s-Hertogenbosch, Northern Brabant, the Netherlands; 52 Center of Medical Genetics, Masaryk Memorial Cancer Institute, Brno, Czech Republic; 53 Department of Psychiatry and Psychotherapy, Comprehensive Center for Clinical Neurosciences and Mental Health (C3NMH), Medical University of Vienna, Vienna, Austria; 54 First Department of Psychiatry, National and Kappodistrian University of Athens, Athens, Greece; 55 Department of Genetics, QIMR Berghofer Medical Research Institute, Brisbane, Queensland Australia; 56 The Lundbeck Foundation Initiative for Integrative Psychiatric Research (iPSYCH), Aarhus University, Aarhus, Denmark; 57 Center for Genomics and Personalized Medicine, Aarhus University, Aarhus, Denmark; 58 BiRC - Section for Bioinformatics and Computational Biology, Aarhus University, Aarhus, Denmark; 59 Department of Emergency and Post-Emergency Psychiatry, CHU, University of Montpellier, Montpellier, France; 60 Helmholtz Zentrum München - German Research Centre for Environmental Health, Institute of Translational Genomics, Neuherberg, Germany; 61 Human Genomics Research Group, Department of Biomedicine, University of Basel, Basel, Switzerland; 62 Department of Genomics, Life and Brain Center, University of Bonn, Bonn, Germany; 63 Institute of Human Genetics, University of Bonn, School of Medicine and University Hospital Bonn, Bonn, Germany; 64 Eating Disorders Unit, Parkland-Klinik, Bad Wildungen, Germany; 65 Department of Clinical Psychology, University Hospital Bellvitge, Hospitalet del Llobregat, Barcelona, Spain; 66 Department of Clinical Sciences, School of Medicine and Health Sciences, University of Barcelona, Hospitalet del Llobregat, Barcelona, Spain; 67 Ciber Physiopathology of Obesity and Nutrition (CIBERObn), Instituto de Salud Carlos III, Madrid, Spain; 68 Psychoneurobiology of Eating and Addictive Behaviors Research Group, Bellvitge Biomedical Research Institute (IDIBELL), Hospitalet del Llobregat, Barcelona, Spain; 69 Centre for Psychological Services, University of Barcelona, Barcelona, Spain; 70 Rheumatology Research Group, Vall d’Hebron Research Institute, Barcelona, Spain; 71 Department of Psychiatry, First Faculty of Medicine, Charles University and General University Hospital, Prague, Czech Republic; 72 Institute of Public Health and Clinical Nutrition, University of Eastern Finland, Kuopio, Finland; 73 Department of Psychological Medicine, University of Otago, Christchurch, New Zealand; 74 Institute of Applied Health Sciences, University of Aberdeen, Aberdeen, UK; 75 Division of Psychological and Social Medicine and Developmental Neurosciences, Faculty of Medicine, Technische Universität Dresden, Dresden, Germany; 76 Department of Psychological Medicine, Institute of Psychiatry, Psychology and Neuroscience, King’s College London, London, UK; 77 Center for Applied Genomics, Children’s Hospital of Philadelphia, Philadelphia, PA USA; 78 Division of Human Genetics, Children’s Hospital of Philadelphia, Philadelphia, PA USA; 79 Department of Pediatrics, University of Pennsylvania Perelman School of Medicine, Philadelphia, PA USA; 80 College of Clinical Psychology , The Chicago School, Washington, DC USA; 81 Department of Psychiatry, University of Campania ‘Luigi Vanvitelli’, Naples, Italy; 82 Department of Medical Research, Vestre Viken Hospital Trust, Bærum Hospital, Gjettum, Norway; 83 Division of Mental Health and Addiction, NORMENT KG Jebsen Centre, Oslo University Hospital, Oslo, Norway; 84 Padova Neuroscience Center, University of Padova, Padova, Italy; 85 Department of Pathology and Molecular Medicine, University of Otago, Christchurch, New Zealand; 86 National Center for PTSD, VA Boston Healthcare System, Boston, MA USA; 87 Department of Psychiatry, Boston University Chobanian and Avedisian School of Medicine, Boston, MA USA; 88 Department of Population Health, QIMR Berghofer Medical Research Institute, Brisbane, Queensland Australia; 89 Department of Medicine Solna, Division of Rheumatology, Karolinska Institutet, Stockholm, Sweden; 90 Department of Psychiatry, Division of Psychiatric Genomics, Icahn School of Medicine at Mount Sinai, New York, NY USA; 91 Department of Genetics and Genomic Sciences, Mindich Child Health and Development Institute, Friedman Brain Institute, Icahn School of Medicine at Mount Sinai, New York, NY USA; 92 Department of Psychiatry, Helsinki University Hospital, Helsinki, Finland; 93 Department of Public Health, University of Helsinki, Helsinki, Finland; 94 Department of General Practice and Primary Healthcare, Faculty of Medical and Health Sciences, The University of Auckland, Auckland, New Zealand; 95 Faculty of Medicine, Sigmund Freud University, Vienna, Austria; 96 Institute of Biology and Medical Genetics, First Faculty of Medicine, Charles University, Prague, Czech Republic; 97 Department of Child and Adolescent Psychiatry, Poznan University of Medical Sciences, Poznan, Poland; 98 Department of Neuroscience, Psychology, Drug Research and Child Health (NEUROFARBA), University of Florence, Florence, Italy; 99 Department of Psychiatry, University of Perugia, Perugia, Italy; 100 Adolescent Health Unit, Second Department of Pediatrics, ‘P. & A. Kyriakou’ Children’s Hospital, National and Kappodistrian University of Athens, Athens, Greece; 101 Department of Clinical Psychology, Faculty for Social Sciences, Utrecht University, Utrecht, the Netherlands; 102 Eating Disorders Unit, Department of Child and Adolescent Psychiatry, Medical University of Vienna, Vienna, Austria; 103 Discipline of Psychology, Curtin University, Perth, Western Australia Australia; 104 Department of Translational Neuroscience, UMC Utrecht Brain Center, University Medical Center Utrecht, Utrecht University, Utrecht, the Netherlands; 105 Department of Physiology, Institute of Neuroscience and Physiology, Sahlgrenska Academy at University of Gothenburg, Gothenburg, Sweden; 106 Institute of Environmental Medicine, Karolinska Institutet, Stockholm, Sweden; 107 Centre for Occupational and Environmental Medicine, Stockholm, Sweden; 108 Centre for Precision Psychiatry, University of Oslo, Oslo, Norway; 109 KG Jebsen Centre for Neurodevelopmental Disorders Research, University of Oslo, Oslo, Norway; 110 Eating Recovery Center, Hunt Valley, MD USA; 111 Department of Psychiatry, ERC Pathlight, University of Maryland, St. Joseph Medical Center, Baltimore, MD USA; 112 Department of Psychiatry, University of Minnesota, Minneapolis, MN USA; 113 Associate Faculty, New York Genome Center, New York, NY USA; 114 Department of Psychosomatic Medicine and Psychotherapy, Hannover Medical School, Hannover, Germany; 115 Department of Nutrition and Dietetics, Harokopio University, Athens, Greece; 116 Department of Psychiatry, Rutgers University, Piscataway, NJ USA; 117 Research Department, Quantitative Genomics Laboratories (qGenomics), Barcelona, Spain; 118 Institute of Mathematics and Statistics, University of Tartu, Tartu, Estonia; 119 Institute of Neuroscience and Medicine (INM-1), Research Center Juelich, Juelich, Germany; 120 Centre for Human Genetics, University of Marburg, Marburg, Germany; 121 Université Paris Cité, INSERM U1266 (IPNP), Institute of Psychiatry and Neuroscience of Paris, Paris, France; 122 Sainte-Anne Hospital (CMME), GHU Paris Psychiatrie et Neurosciences, Paris, France; 123 Department of Child and Adolescent Psychiatry, Psychosomatics and Psychotherapy, RWTH Aachen University, Aachen, Germany; 124 Biological Psychiatry Laboratory, McLean Hospital, Harvard Medical School, Belmont, MA USA; 125 Eating Recovery Center, Denver, CO USA; 126 Specialist Mental Health Clinical Research Unit, Health New Zealand Canterbury, Christchurch, New Zealand; 127 Department of Psychiatry, Centre for Addiction and Mental Health, University of Toronto, Toronto, Ontario Canada; 128 Department of Child and Adolescent Psychiatry, Medical University of Vienna, Vienna, Austria; 129 Groningen Institute for Evolutionary Life Sciences, University of Groningen, Groningen, the Netherlands; 130 Department of Psychiatry, University of California, San Diego, San Diego, CA USA; 131 Department of Psychiatry, University of Toronto, Toronto, Ontario Canada; 132 Tanenbaum Centre, Centre for Addiction and Mental Health, Toronto, Ontario Canada; 133 Department of Psychiatry, Ilsan Paik Hospital, Inje University, Goyang, Republic of Korea; 134 Department of Psychology, Michigan State University, East Lansing, MI USA; 135 Department of Psychiatry and Neurochemistry, Institute of Neuroscience and Physiology, University of Gothenburg, Gothenburg, Sweden; 136 Department of Clinical Science, University of Bergen, Bergen, Norway; 137 Department of Neuroscience, Janssen Research and Development LLC, Titusville, NJ USA; 138 Human Genetics and Genomics, CHDI Management Inc., Princeton, NJ USA; 139 Maria Sklodowska-Curie National Research Institute of Oncology, Warsaw, Poland; 140 Department of Psychiatry, Amsterdam University Medical Center, Amsterdam, the Netherlands; 141 Department of Psychiatry and Neuropsychology, School for Mental Health and Neuroscience, Maastricht University Medical Center, Maastricht, the Netherlands; 142 InsideOut Institute, University of Sydney, Sydney, New South Wales Australia; 143 Department of Community Health and Epidemiology, Dalhousie University, Halifax, Nova Scotia Canada; 144 Institute of Psychiatric Phenomics and Genomics, Ludwig-Maximilians-Universität München, Munich, Germany; 145 Department of Mental Health and Neuroscience, QIMR Berghofer Medical Research Institute, Brisbane, Queensland Australia; 146 School of Psychology, University of Queensland, Brisbane, Queensland Australia; 147 School of Psychology and Counselling, Queensland University of Technology, Brisbane, Queensland Australia; 148 University of Colorado School of Medicine, Aurora, CO USA; 149 Great Ormond Street Institute of Child Health, University College London, London, UK; 150 Department of Psychiatry and Behavioral Science, University of North Dakota, Fargo, ND USA; 151 Department of Medicine, Surgery and Dentistry ‘Scuola Medica Salernitana’, University of Salerno, Salerno, Italy; 152 Center for Neurobehavioral Genetics, Semel Institute for Neuroscience and Human Behavior, University of California, Los Angeles, Los Angeles, CA USA; 153 Université Paris Cité, Paris, France; 154 Institute of Clinical Medicine, University of Oslo, Oslo, Norway; 155 German Center for Mental Health (DZPG), Berlin-Potsdam, Germany; 156 Department of Psychiatry and Psychotherapy, Charité - Universitätsmedizin, Berlin, Germany; 157 Department of Adult Psychiatry, Poznan University of Medical Sciences, Poznan, Poland; 158 The Centre for Applied Genomics, Program in Genetics and Genomic Biology, The Hospital for Sick Children, Toronto, Ontario Canada; 159 McLaughlin Centre and Department of Molecular Genetics, University of Toronto, Toronto, Ontario Canada; 160 GGZ Rivierduinen Eating Disorders Ursula, Leiden, the Netherlands; 161 Department of Psychiatry, Leiden University Medical Centre, Leiden, the Netherlands; 162 Psychiatry Department, McGill University, Montreal, Quebec Canada; 163 Eating Disorders Continuum, Douglas Mental Health University Institute, Montreal, Quebec Canada; 164 Department of Environmental Epidemiology, Nofer Institute of Occupational Medicine, Lodz, Poland; 165 Discipline of Psychology, Flinders Institute for Mental Health and Wellbeing, Adelaide, South Australia Australia; 166 Department of Clinical Medicine, University of Copenhagen, Copenhagen, Denmark; 167 School of Nursing, University of North Carolina at Chapel Hill, Chapel Hill, NC USA; 168 Department of Psychosomatic Medicine and Psychotherapy, University Medical Hospital Tübingen, Tübingen, Germany; 169 German Centre for Mental Health, University of Tübingen, Tübingen, Germany; 170 Department of Nutrition, University of North Carolina at Chapel Hill, Chapel Hill, NC USA

**Keywords:** Genomics, Behavioural genetics, Psychiatric disorders, Genome-wide association studies

## Abstract

Eating disorders—including anorexia nervosa (AN), bulimia nervosa and binge-eating disorder—are clinically distinct but exhibit symptom overlap and diagnostic crossover. Genomic analyses have mostly examined AN. Here we conducted a genomic meta-analysis of case–control studies of binge-eating behavior (BE; 39,279 cases, 1,227,436 controls), alongside analyses of AN (24,223 cases, 1,243,971 controls) and its subtypes (all European ancestries). We identified six BE-associated loci, including loci associated with a higher body mass index and impulse-control behaviors. AN genome-wide association studies yielded eight loci, validating six loci. Subsequent polygenic risk score analysis demonstrated an association with AN in two East Asian ancestry studies. BE and AN exhibited similar positive genetic correlations with psychiatric disorders but opposing genetic correlations with anthropometric traits. Most of the genetic signal in BE and AN was not shared with body mass index. We have extended eating disorder genomics beyond AN; future work will incorporate multiple diagnoses and global ancestries.

## Abstract (web summary)

This research identified six areas in the genome that are associated with binge eating, and eight areas that are associated with anorexia nervosa, in people of European ancestry. Binge eating has both shared and distinct genetic features compared with anorexia nervosa.

## Main

The primary eating disorders—anorexia nervosa (AN), bulimia nervosa and binge-eating (BE) disorder—are diagnostically distinct yet show considerable overlap and diagnostic migration over time^1–3^. AN, characterized by low weight, fear of weight gain and an inability to recognize the seriousness of the low weight, has two subtypes: restricting (AN-R) and BE/purging (AN-BP), featuring BE and/or purging behaviors. Bulimia nervosa occurs in individuals with a normal or high weight and is characterized by BE and compensatory behaviors (for example, fasting, self-induced vomiting, laxative use and diuretic use). BE disorder also includes BE at normal or high weights but without recurrent compensatory behaviors^1^.

Genome-wide association studies (GWASs) of eating disorders have focused primarily on AN^4–7^, with few GWASs of other eating disorder-related phenotypes^8,9^. The most recent AN GWAS^6^ included 16,992 cases and identified eight genome-wide significant loci. Single-nucleotide polymorphism-based genetic correlations (SNP-*r*_g_s) with other psychiatric disorders were high and also suggested that metabolic and anthropometric factors might underlie AN pathophysiology^6,7^. The metabolic aspect of AN is reflected by a positive SNP-*r*_g_ with high-density lipoprotein cholesterol and negative SNP-*r*_g_s with insulin resistance, leptin and type 2 diabetes. Importantly, these SNP-*r*_g_s were independent of body mass index (BMI), which is relevant given that a low BMI is pathognomonic of AN.

Here, we expand the genetic landscape of eating disorders beyond AN. AN and bulimia nervosa show moderate family- and twin-based genetic correlations^10,11^, suggesting shared genetic risk. This may partially reflect BE, a transdiagnostic symptom common to both bulimia nervosa and AN-BP. Psychiatric diagnoses defined by the Diagnostic and Statistical Manual of Mental Disorders (DSM) and International Classification of Diseases (ICD) may not capture biological realities or patient experiences, given that disorders often exist on a continuum, overlap, co-occur, present heterogeneously and morph over time^12^. Focusing on diagnostic thresholds can bias research towards severe cases and exclude those with substantial pathology and impairment. Symptom-based approaches can complement diagnostic approaches and advance the science of eating disorders^13^. We present a GWAS of the transdiagnostic symptom of BE behavior, an augmented AN GWAS and GWASs of explicitly defined AN-R and AN-BP.

## Results

### Summary of phenotypes

The genetic ancestry and genetically derived sex of the participants are presented in Supplementary Table 1. We operationalized five phenotypes (Table 1): broadly defined BE (BE-BROAD), narrowly defined BE (BE-NARROW), AN, AN-R and AN-BP. We focus on BE-BROAD (owing to greater statistical power than BE-NARROW; Supplementary Results 1) and AN. Analyses of other phenotypes are presented in Supplementary Results 2. We contrast BE-BROAD with GWAS of a model-derived proxy BE disorder phenotype from the Million Veteran Program^9^ (Supplementary Results 3).

**Table 1:** Phenotype definitions

Phenotype	Inclusion criteria: self-report or questionnaires	Inclusion criteria: diagnostic codes
BE-BROAD BE broadly defined (including BE-NARROW)	1. Eating an unusually large amount of food in a short period of time with loss of control; frequency and duration criteria not required, or 2. If assessed by a single item such as “Have you ever experienced BE?”*, or 3. A self-reported diagnosis of bulimia nervosa or BE disorder, confirmation by healthcare professional not required**	307.51 (ICD-9, DSM-III-R, DSM-IV) F50.2, F50.3 (ICD-10) 307.51 [F50.2, F50.3] (DSM-5) F50.81 (ICD-10-CM)
BE-NARROW BE narrowly defined	1. Eating an unusually large amount of food in a short period of time with loss of control, and these episodes occurred on average at least once a week for at least 3 months, or 2. A clinical diagnosis of bulimia nervosa or BE disorder via hospital/register records or a structured clinical interview, or 3. A self-reported diagnosis of bulimia nervosa or BE disorder with confirmation by a healthcare professional	307.51 (ICD-9, DSM-III-R, DSM-IV) F50.2, F50.3 (ICD-10) 307.51 [F50.2, F50.3] (DSM-5) F50.81 (ICD-10-CM)
AN AN—no subtype specified (including AN-R and AN-BP)	1. A clinical diagnosis of AN or AN-BP subtype or AN-R subtype via hospital or register records, structured clinical interviews or online questionnaires on the basis of standardized criteria, or 2. A self-reported diagnosis of AN or AN-BP subtype or AN-R subtype; confirmation by healthcare professional not required Note: atypical AN (without low body weight) was not specifically excluded from our analyses but has been historically ill defined and was not ascertained for in most studies. As such, our AN data primarily reflect typical AN	306.50 (ICD-8) 307.1 (ICD-9, DSM-III-R, DSM-IV) F50.0 (ICD-10) F50.1 (ICD-10) 307.1 [F50.1] (DSM-5)
AN-R AN-R subtype	1. A clinical diagnosis of AN-R subtype via hospital or register records, structured clinical interviews or online questionnaires on the basis of standardized criteria, or 2. A self-reported diagnosis of AN-R subtype; confirmation by healthcare professional not required	F50.01 (ICD-10) 307.1 [F50.01] (DSM-5)
AN-BP AN-BP subtype	1. A clinical diagnosis of AN-BP subtype via hospital or register records, structured clinical interviews or online questionnaires on the basis of standardized criteria, or 2. A self-reported diagnosis of AN-BP subtype; confirmation by healthcare professional not required	F50.02 (ICD-10) 307.1 [F50.02] (DSM-5)
Controls	1. No history of any eating disorder and no history of BE (broadly or narrowly defined), or 2. No history of any eating disorder, or 3. Unscreened (that is, these individuals may have had an eating disorder or BE)	

Diagnostic codes are shown with the coding system in round brackets and equivalent codes in square brackets. Note that diagnostic codes relate to ‘diagnosis’ criteria and that some criteria for BE do not require a diagnosis. Control definitions are shown in order of preference.

Footnotes: *If assessed with terms such as ‘psychological overeating’, ‘compulsive eating’, and so on, these individuals will not be included as BE-BROAD cases. **If Other Specified Feeding or Eating Disorder or Eating Disorder Not Otherwise Specified is diagnosed, these are included only if clearly stated ‘subthreshold bulimia nervosa’ or ‘subthreshold binge-eating disorder’.

### GWAS meta-analyses

**Fig. 1:**
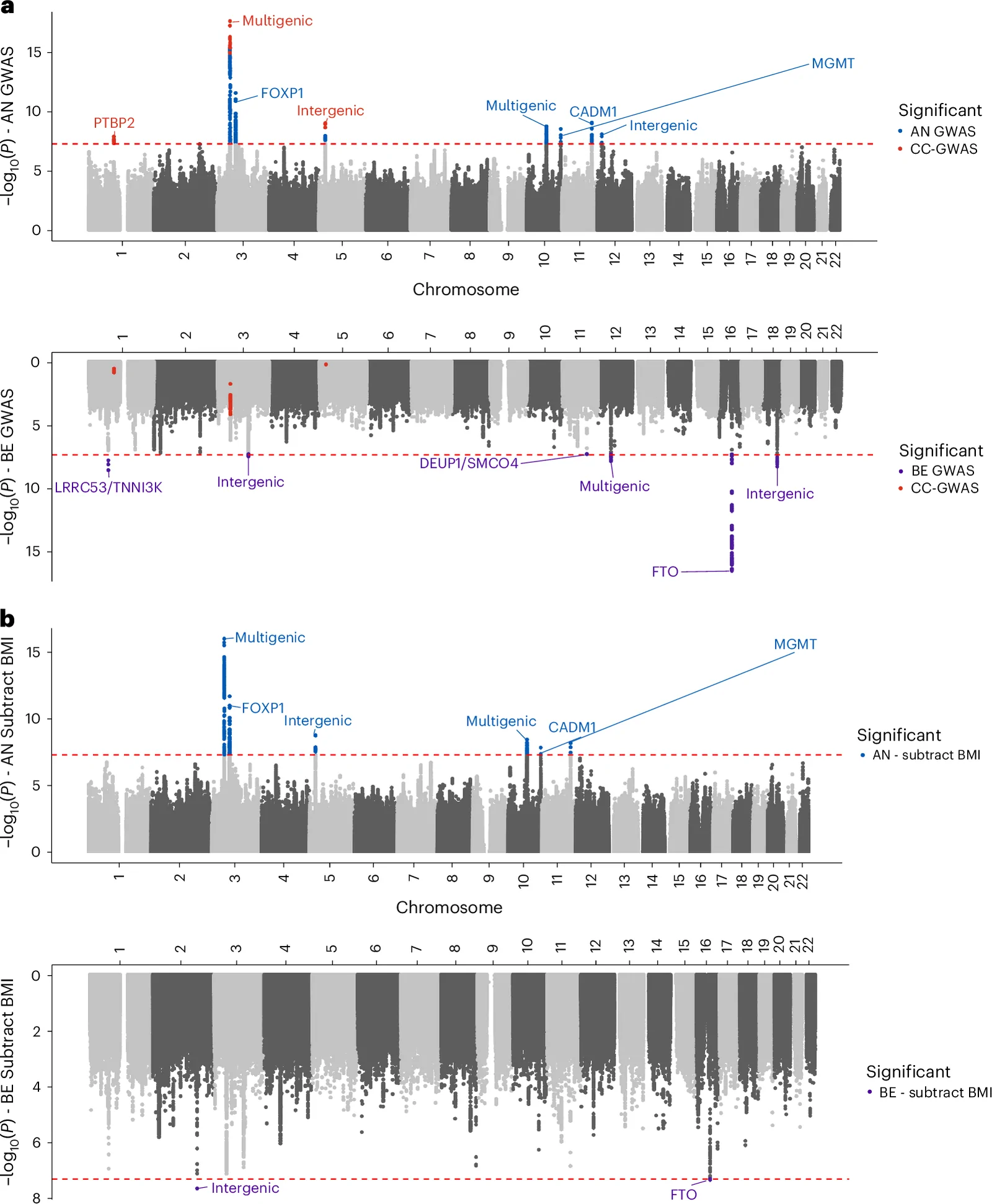
Miami plots of AN and BE-BROAD GWAS association analyses with and without the BMI component. **a**,**b**, Miami plots showing results from the AN (top; 24,223 cases, 1,243,971 controls) and BE-BROAD (bottom; 39,279 cases, 1,227,436 controls) meta-analyses. The dotted red line indicates the genome-wide significance threshold (*P* ≤ 5 × 10^−8^). We performed association analyses using two-sided logistic regression (PLINK2, SAIGE or REGENIE, depending on study characteristics) followed by fixed-effects meta-analysis. Main GWAS analyses, with variants reaching genome-wide significance colored in blue if significant in the AN GWAS and in purple if significant in the BE-BROAD GWAS. Variants reaching genome-wide significance in the case–case GWAS of BE-BROAD versus AN are colored in red (**a**). GWAS-by-subtraction analyses, showing results from the non-BMI genetic component. Variants reaching genome-wide significance are colored in blue for the AN non-BMI component and in purple for the BE-BROAD non-BMI component (**b**).

For BE-BROAD (17 European-ancestry datasets, 39,279 cases, 1,227,436 controls), we identified six independently associated loci (Fig. 1, Table 2, Supplementary Figs. 1–6 and Supplementary Tables 1 and 2). The liability-scale SNP-based heritability (h^2^_SNP_) was 5% (standard error (SE) of 0.4%, assuming population prevalence of 4.5%^14^) with a linkage disequilibrium score regression (LDSC) intercept of 1.03 and an attenuation ratio of 0.14 (SE of 0.04; Supplementary Table 3), suggesting that inflation was primarily due to polygenicity^15^.

**Table 2:** Association statistics for genome-wide significant loci for BE-BROAD and AN

Phenotype	Locus	CHR	Locus start	Locus end	Lead SNP	*P* value	REF/ALT	OR	SE	ALT frequency cases	ALT frequency controls	INFO		Genes
BE-BROAD	1	1	74,977,295	75,014,362	rs7541513	2.65 × 10^−9^	G/A	1.05	0.009	0.427	0.425	0.952	–	*LRRC53*/*TNNI3K*
	2	3	117,420,697	117,953,697	rs1456193	3.25 × 10^−8^	T/C	1.06	0.011	0.808	0.802	0.996	–	Intergenic
	3	11	93,171,140	93,231,940	rs2925354	4.91 × 10^−8^	G/A	0.93	0.013	0.107	0.112	0.995	–	*DEUP1*/*SMCO4*
	4	12	50,169,080	50,285,780	rs7953534	1.40 × 10^−8^	G/A	1.05	0.009	0.351	0.333	0.985	–	Multigenic
	5	16	53,797,904	53,845,494	rs11642015	2.97 × 10^−17^	C/T	1.07	0.009	0.429	0.417	1.000	–	*FTO*
	6	18	57,730,978	57,914,978	rs66723169	5.02 × 10^−9^	C/A	1.06	0.010	0.254	0.209	0.995	–	Intergenic
AN	1	1	96,700,455	97,285,455	rs10747478	1.43 × 10^−8^	T/G	0.94	0.011	0.570	0.587	0.998	Yes	*PTBP2*
	2	3	47,501,450	51,741,450	rs113519699	2.86 × 10^−18^	A/C	1.18	0.019	0.102	0.087	0.997	Yes	Multigenic
	3	3	70,602,234	71,074,242	rs9310201	3.12 × 10^−12^	A/T	1.08	0.011	0.427	0.408	0.982	Yes	*FOXP1*
	4	5	24,868,440	25,300,440	rs6872919	1.07 × 10^−9^	A/C	1.07	0.011	0.570	0.532	0.997	Yes	Intergenic
	5	10	75,850,972	76,524,972	rs10740439	1.91 × 10^−9^	C/T	0.93	0.012	0.711	0.716	0.994	No	Multigenic
	6	10	131,274,055	131,459,255	rs12762024	3.28 × 10^−9^	G/C	1.07	0.011	0.447	0.455	0.995	Yes	*MGMT*
	7	11	114,997,256	115,284,956	rs6589488	9.80 × 10^−9^	A/T	0.91	0.016	0.846	0.875	0.990	Yes	*CADM1*
	8	12	17,635,314	17,965,314	rs12817084	8.93 × 10^−9^	T/C	1.10	0.011	0.115	0.117	0.991	No	Intergenic

Association analyses were performed using two-sided logistic regression (PLINK2, SAIGE or REGENIE depending on study characteristics); genome-wide significance was defined as *P* < 5 × 10⁻^8^ with no additional multiple correction applied. Loci are numbered sequentially within analysis from chromosome 1 to chromosome X. Base pair start and end positions correspond to hg19. ORs are given relevant to the ALT allele. Genes are listed if at least one transcript in GENCODE version 47 lies at least partially within the locus, with more than three genes defined as multigenic. ALT, alternative (tested) allele; CHR, chromosome; INFO, imputation information metric; OR, odds ratio; REF, reference allele; SE, standard error; SNP, single-nucleotide polymorphism.

For AN (26 European-ancestry datasets, 24,223 cases, 1,243,971 controls) we identified eight independently associated loci—six known^6^ and two additional (Fig. 1, Table 2, Supplementary Figs. 7–14 and Supplementary Tables 1 and 2). Three previously significant loci (on chromosome 2 and 3 (ref. ^6^), and on chromosome 12 (ref. ^7^)) did not reach genome-wide significance (*P* = 2 × 10^−7^–6 × 10^−7^). The liability-scale h^2^_SNP_ was 13% (﻿SE of 0.7%, assuming a population prevalence of 1.5%^16^), the intercept was 1.02, significantly >1, and the attenuation ratio was 0.07 (﻿SE of 0.03), suggesting polygenicity (Supplementary Table 3).

Analyses of chromosome X yielded no genome-wide significant loci for BE-BROAD nor AN, although one genome-wide significant locus was identified for BE-NARROW (Supplementary Results 2, Supplementary Fig. 15 and Supplementary Tables 4 and 5).

Given known sex differences in eating disorders, and mostly female cases in our data (96% in BE-BROAD, 94% in AN), we conducted female-only GWASs as sensitivity analyses. Results mirrored the main analyses, with differences attributable to reduced sample size (Supplementary Results 4 and Supplementary Table 6).

### Genetic relationship between eating phenotypes and other traits

**Fig. 2:**
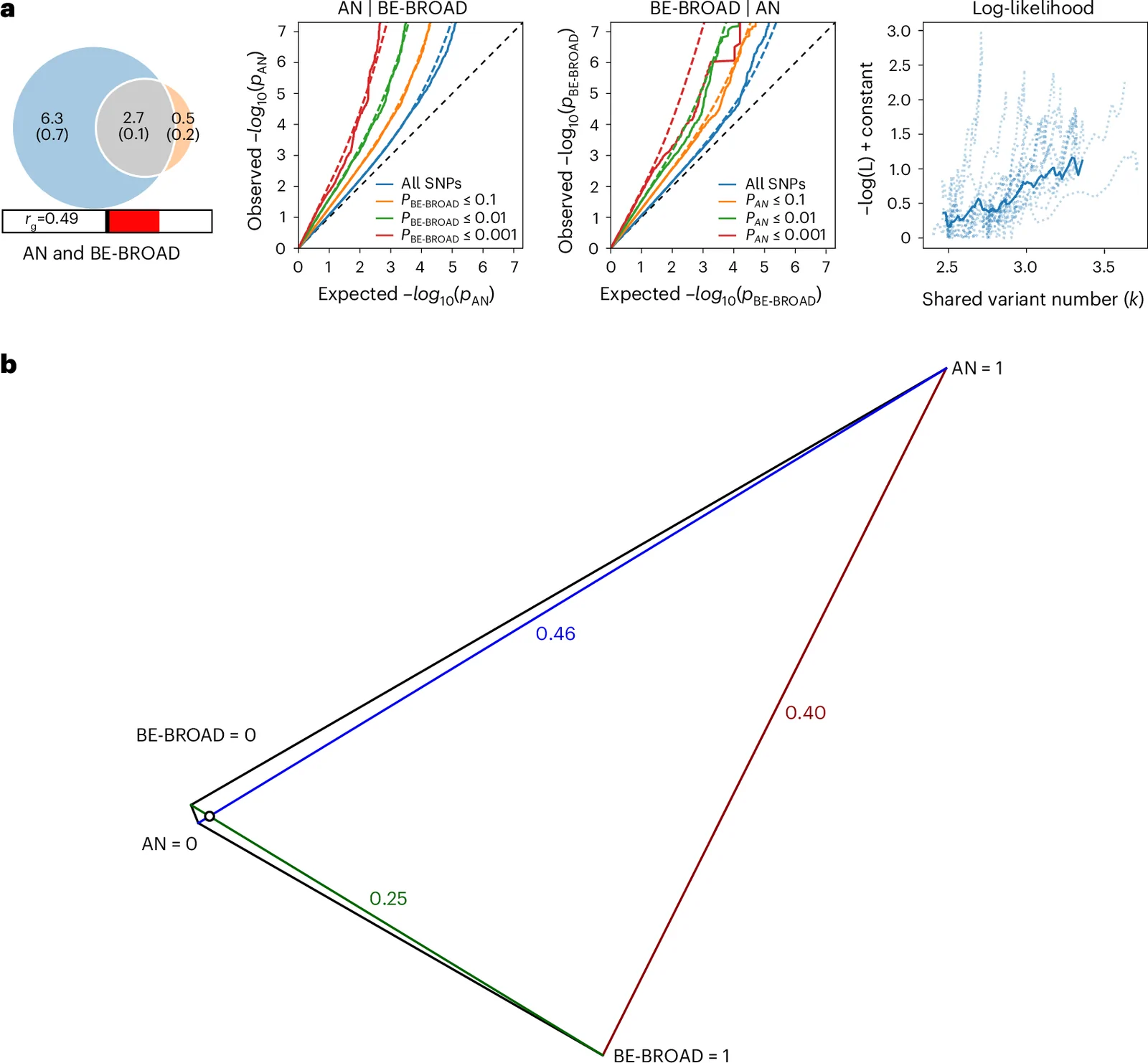
Estimates of genetic similarity between BE-BROAD and AN. **a**, Bivariate causal mixture modeling results from MiXeR (AN: 24,223 cases, 1,243,971 controls; BE-BROAD: 39,279 cases, 1,227,436 controls). Left: venn diagram showing overlap of putative causal variants in AN (blue) and BE-BROAD (orange), demonstrating high overlap (gray) and moderate genetic correlation (bar beneath). Middle: Q–Q plots of variant effects for AN conditional on BE-BROAD (left) and BE-BROAD conditional on AN (right), demonstrating enrichment for associations with each trait conditional on the other. Right: log-likelihood plot of model fit under differing models, from the minimal overlap model (leftmost) to the full overlap model (rightmost), showing that the best model fit (lowest *y* axis value) is not distinguishable from the minimal overlap. **b**, Genetic distance between cases and controls of BE-BROAD and AN estimated by case–case GWAS. The genetic distance is the square root of the average squared difference in allele frequency at causal SNPs.

We assessed the genetic similarity of BE-BROAD and AN via bivariate causal mixture modeling (‘MiXeR’), via case–case GWAS and through examining their SNP-*r*_g_ with each other and with other traits^17,18^. The SNP-*r*_g_ between BE-BROAD and AN was 0.46 (﻿SE of 0.04, *P* = 3.44 × 10^−30^), indicating moderate genetic overlap (Supplementary Table 7). Bivariate MiXeR yielded similar SNP-*r*_g_ (0.49, ﻿SE of 0.01) and indicated 2,654 overlapping causal variants (83% of all BE-BROAD causal variants) with highly concordant effects (95%; Fig. 2a). This was not distinguishable from the minimum plausible overlap given the genetic correlation observed—an overlap of >2,450 fully concordant causal variants (Supplementary Results 5 and Supplementary Table 8). Case–case GWAS uses genetic distance, the average squared difference in allele frequency at causal SNPs^17^. This was higher between individuals with AN and controls (0.46) and between case groups (0.40), than between individuals with BE-BROAD and controls (0.25, Fig. 2b). We identified case-divergent loci on chromosomes 1, 3 and 5, overlapping with loci from the AN GWAS (Fig. 1), suggesting that some loci differentiate AN from controls and from BE-BROAD.

We used LDSC to calculate pairwise SNP-*r*_g_ for BE-BROAD and AN with 225 psychiatric, personality, metabolic and anthropometric traits (Table 3 and Supplementary Table 9). We generally observed positive SNP-*r*_g_ of BE-BROAD with psychiatric and anthropometric phenotypes, except for persistent thinness and pubertal growth, which were negative. No SNP-*r*_g_ between metabolic traits and BE-BROAD were significant except for BMI-adjusted fasting insulin. We validated and extended previously observed SNP-*r*_g_ patterns with AN^6^: positive SNP-*r*_g_ with psychiatric disorders, neuroticism, educational attainment and physical activity; negative SNP-*r*_g_ with metabolic and anthropometric traits (except for total cholesterol in high-density lipoprotein); and, notably, nonsignificant SNP-*r*_g_ with persistent thinness (Table 3 and Supplementary Table 9).

**Table 3:** A representative subset of significant genetic correlations (*r*_g_) of BE-BROAD and AN with external traits

Trait	Category	PMID	*r*_g_ (AN)	SE (AN)	*P* value (AN)	*r*_g_ (BE)	SE (BE)	*P* value (BE)
BMI	Anthropometric	30239722	−0.31	0.020	**2.37** **×** **10**^−**56**^	0.36	0.032	**1.94** **×** **10**^−**28**^
Body fat percentage	Anthropometric	30593698	−0.35	0.028	**2.71** **×** **10**^−**36**^	0.20	0.042	**2.65** **×** **10**^−**6**^
Fat-free mass	Anthropometric	30593698	−0.15	0.022	**2.93** **×** **10**^−**11**^	0.30	0.034	**6.92** **×** **10**^−**19**^
Fat mass	Anthropometric	31852892	−0.32	0.023	**3.63** **×** **10**^−**43**^	0.31	0.035	**1.36** **×** **10**^−**18**^
Persistent thinness	Anthropometric	30677029	0.11	0.074	0.123	−0.46	0.110	**2.37** **×** **10**^−**5**^
Pubertal growth (height difference, 8 years to adult)	Anthropometric	23449627	−0.03	0.053	0.606	−0.37	0.079	**3.08** **×** **10**^−**6**^
Severe early-onset obesity	Anthropometric	30677029	−0.11	0.059	0.0587	0.48	0.080	**1.40** **×** **10**^−**9**^
Waist–hip ratio	Anthropometric	30239722	−0.25	0.021	**1.72** **×** **10**^−**34**^	0.19	0.034	**1.37** **×** **10**^−**8**^
Waist–hip ratio (BMI-adjusted)	Anthropometric	30239722	−0.09	0.020	**9.33** **×** **10**^−**6**^	−0.05	0.033	0.119
Fasting insulin (age- and sex-adjusted)	Metabolism	33402679	−0.30	0.048	**2.79** **×** **10**^−**10**^	0.15	0.069	0.0292
Fasting insulin (BMI-adjusted)	Metabolism	34059833	−0.17	0.035	**9.62** **×** **10**^−**7**^	−0.29	0.047	**6.29** **×** **10**^−**10**^
HbA1c	Metabolism	28898252	−0.14	0.042	6.00 × 10^−4^	−0.03	0.053	0.524
HbA1c (BMI-adjusted)	Metabolism	34059833	−0.16	0.034	**5.22** **×** **10**^−**6**^	0.01	0.044	0.765
Type 2 diabetes	Metabolism	30297969	−0.18	0.024	**1.76** **×** **10**^−**14**^	0.11	0.040	6.10 × 10^−3^
ADHD	Psychiatric	36702997	0.07	0.029	0.0126	0.31	0.042	**2.20** **×** **10**^−**13**^
Autism	Psychiatric	32747698	0.17	0.044	**1.00** **×** **10**^−**4**^	0.29	0.057	**3.96** **×** **10**^−**7**^
Bipolar disorder	Psychiatric	34002096	0.25	0.027	**3.80** **×** **10**^−**21**^	0.23	0.035	**4.22** **×** **10**^−**11**^
Insomnia	Psychiatric	30804565	0.10	0.033	2.90 × 10^−3^	0.21	0.044	**2.02** **×** **10**^−**6**^
MDD	Psychiatric	39814019	0.30	0.025	**1.04** **×** **10**^−**33**^	0.39	0.031	**1.35** **×** **10**^−**36**^
OCD	Psychiatric	28761083	0.48	0.069	**2.85** **×** **10**^−**12**^	0.08	0.081	0.322
Probable anxiety diagnosis	Psychiatric	31748690	0.28	0.039	**1.42** **×** **10**^−**12**^	0.32	0.060	**1.03** **×** **10**^−**7**^
PTSD	Psychiatric	33510476	0.11	0.049	0.0274	0.24	0.062	**1.00** **×** **10**^−**4**^
Schizophrenia	Psychiatric	35396580	0.24	0.021	**4.85** **×** **10**^−**30**^	0.23	0.033	**7.04** **×** **10**^−**12**^
General risk tolerance	Behavioral	30643258	0.00	0.028	0.991	0.21	0.040	**6.76** **×** **10**^−**8**^
Loneliness	Behavioral	31518406	0.13	0.032	**4.96** **×** **10**^−**5**^	0.37	0.044	**4.14** **×** **10**^−**17**^
Physical activity	Behavioral	36071172	0.19	0.034	**5.87** **×** **10**^−**8**^	0.09	0.046	0.0633
Educational attainment (years)	Sociodemographic	30038396	0.25	0.022	**1.25** **×** **10**^−**29**^	0.04	0.030	0.142
AUDIT-P	Substance use	30336701	0.01	0.040	0.870	0.37	0.057	**7.00** **×** **10**^−**11**^
Cannabis use disorder	Substance use	33096046	0.02	0.046	0.610	0.30	0.063	**2.78** **×** **10**^−**6**^
Heavy smoker	Substance use	28166213	−0.04	0.039	0.255	0.30	0.048	**3.82** **×** **10**^−**10**^
Lifetime cannabis use	Substance use	30150663	0.22	0.038	**8.48** **×** **10**^−**9**^	0.32	0.051	**6.36** **×** **10**^−**10**^

*r*_g_s were calculated using two-sided LDSC, with Bonferroni correction applied for 225 traits tested (*P* < 2 × 10^4^); bold values indicate *r*_g_ estimates surviving this threshold. Note: the full set of genetic correlations is presented in Supplementary Table 9. ADHD, attention deficit hyperactivity disorder; AUDIT-P, Alcohol Use Disorder Identification Test Problem items; MDD, major depressive disorder; OCD, obsessive–compulsive disorder; PMID, PubMed ID of GWAS for external trait; PTSD, post-traumatic stress disorder; *r*_g_, single-nucleotide polymorphism-based genetic correlation; ﻿SE, standard error of the mean.

**Fig. 3:**
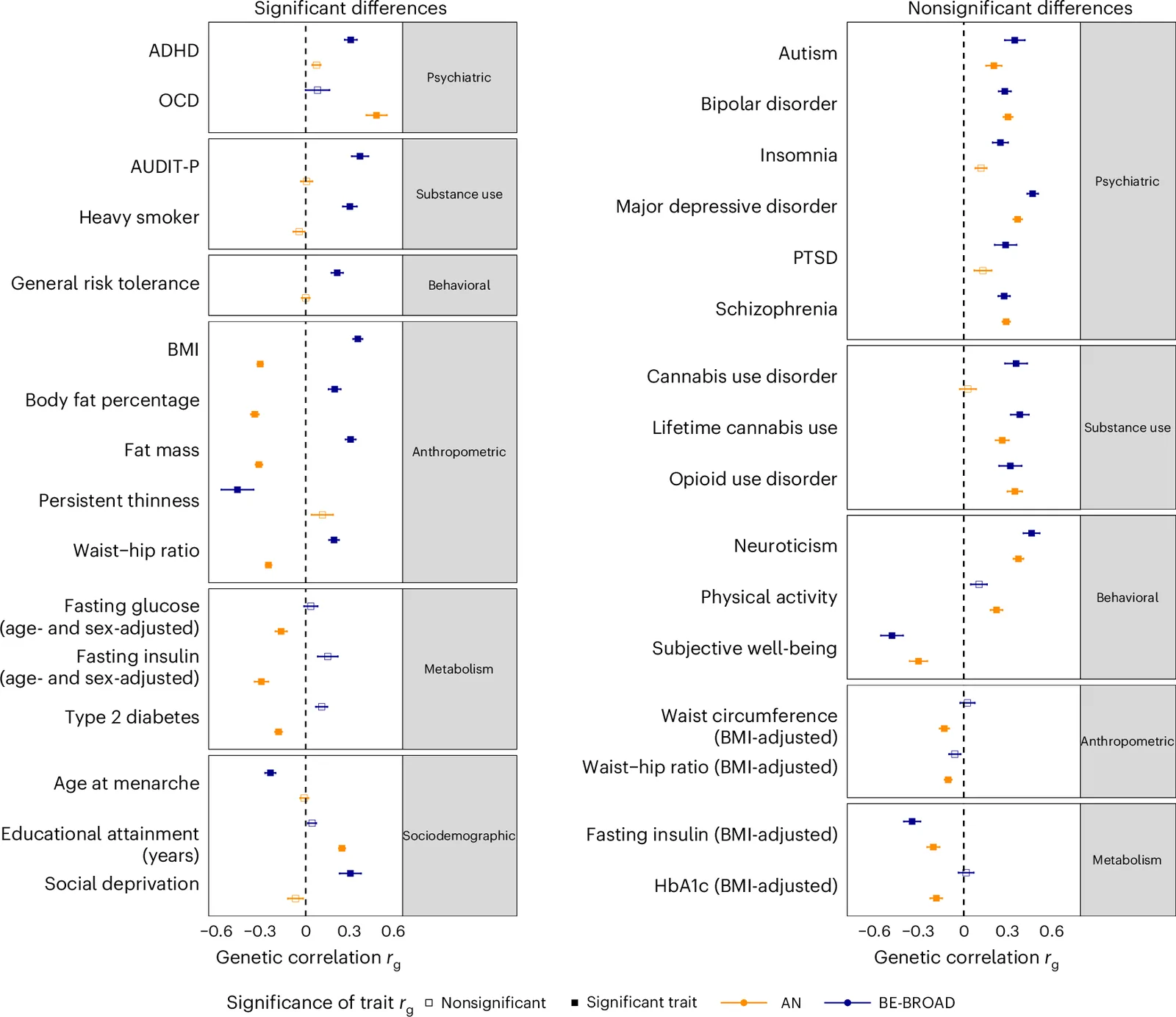
Genetic correlations (*r*_g_) of selected external traits with BE-BROAD and AN. Point estimates represent *r*_g_s as estimated by LDSC, with error bars indicating ±1 ﻿SE. Filled dots indicate traits with a significant *r*_g_ with BE-BROAD and/or AN after Bonferroni correction (*P* < 2 × 10⁻^4^, on the basis of 225 traits tested); open/unfilled dots indicate nonsignificant *r*_g_ estimates. Information about the summary statistics used in our analysis is presented in Supplementary Table 9. PGC, Psychiatric Genomics Consortium; FFM, fat-free mass. Left: genetic correlations differing significantly between BE-BROAD (39,279 cases, 1,227,436 controls) and AN (24,223 cases, 1,243,971 controls). Right: genetic correlations not differing between BE-BROAD and AN.

We tested for significant differences between the SNP-*r*_g_ of BE-BROAD and SNP-*r*_g_ of AN with other traits (Fig. 3, Supplementary Fig. 16 and Supplementary Table 10). Most psychiatric and behavioral phenotypes showed similar SNP-*r*_g_ with BE-BROAD and AN except attention deficit hyperactivity disorder (ADHD; higher with BE-BROAD, difference *P* = 1.06 × 10^−7^) and obsessive–compulsive disorder (higher with AN, *P* = 4.79 × 10^−5^). BE-BROAD, but not AN, was positively correlated with Alcohol Use Disorder Identification Test (AUDIT-P) problem items, four smoking-related phenotypes and general risk tolerance (*P* ≤ 4.83 × 10^−7^). AN, but not BE-BROAD, was negatively correlated with automobile speeding propensity (*P* = 1.21 × 10^−4^).

BE-BROAD and AN diverged in their associations with anthropometric and metabolic traits. For example, BE-BROAD was positively correlated, and AN negatively, with waist-to-hip ratio (*P* = 2.05 × 10^−31^). BE-BROAD showed a stronger pattern of SNP-*r*_g_ with certain sociodemographic traits (*P* < 2 × 10^−4^), displaying negative SNP-*r*_g_s with age at menarche and age at first birth in females and positive SNP-*r*_g_s with social deprivation and loneliness. AN showed no significant SNP-*r*_g_ with these traits but was more strongly positively associated than BE-BROAD with educational traits such as college/university completion.

As 18% of BE-BROAD cases had (known) AN (and 30% of AN cases met the criteria for BE-BROAD), BE-BROAD results may include AN-related effects (Supplementary Table 11). To test this, we repeated the BE-BROAD GWAS, excluding studies that focused on AN recruitment (Supplementary Results 6). The SNP-*r*_g_ between BE-BROAD and the reduced GWAS did not differ from unity (0.96, ﻿SE of 0.07) but SNP-*r*_g_s with anthropometric traits were stronger in the reduced GWAS, suggesting that AN cases with BE may mask BE–anthropometric genetic associations (Supplementary Fig. 17 and Supplementary Table 12).

### Role of BMI genetics

We applied GWAS-by-subtraction to assess the complex role of BMI in eating disorders by removing BMI-related genetic variance^19^. We modeled a factor shared between each eating phenotype and BMI and a non-BMI factor capturing the eating phenotype only. The shared factor explained 12% (﻿SE of 2.7%) of genetic variance in BE-BROAD, leaving 88% (﻿SE of 7.9%) accounted for by the non-BMI factor. In AN, the shared factor accounted for 10% (﻿SE 1.4%) of genetic variance and the non-BMI factor, 90% (﻿SE 5.5%). GWASs of the non-BMI factor for both phenotypes generally yielded larger *P* values for lead SNPs but consistent effect sizes (Fig. 1 and Supplementary Table 13). The non-BMI factors showed stable or slightly increased SNP-*r*_g_ with psychiatric disorders compared with the full GWASs, whereas SNP-*r*_g_ with anthropometric and metabolic traits were typically attenuated (Supplementary Fig. 18 and Supplementary Table 14).

Mendelian randomization analyses supported causal effects in both directions between a higher BMI risk and higher BE-BROAD/lower AN risk. The BE-BROAD non-BMI component was associated with a higher BMI, while the AN non-BMI component was not. Associations of BMI with both non-BMI components were inconsistent across methods (Supplementary Results 7 and Supplementary Table 15).

### Genetically regulated gene expression

We used S-PrediXcan^20^ to identify predicted genetically regulated gene expression associated with our phenotypes (Supplementary Fig. 19 and Supplementary Table 16). Within-tissue significant results are described in Supplementary Results 8. For BE-BROAD, two gene–tissue associations were experiment-wide significant (*PRKAR2A*–sigmoid colon and *KLHDC8B*–heart, atrial appendage; *P* < 8.32 × 10^−8^). In AN, 300 gene–tissue associations were experiment-wide significant (*P* < 8.32 × 10^−8^) across 29 unique genes, predominantly from the gene-dense locus on chromosome 3: 47–52 Mb.

We calculated cross-tissue-predicted genetically regulated gene expression using S-MultiXcan^21^ to identify apparent tissue-specific associations that are better interpreted as cross-tissue (Supplementary Results 9, Supplementary Fig. 20 and Supplementary Table 17). Ten genes had significant (*P* < 2.25 × 10^−6^) cross-tissue expression in BE-BROAD, including four tissue-level associations (including *KLHDC8B* but not *PRKAR2A*). A total of 43 genes showed significant cross-tissue expression in AN, including 23 tissue-level associations.

### Gene-level associations

We conducted gene-wise (Supplementary Table 18), gene-set (Supplementary Table 19), drug-set (Supplementary Table 20) and drug-class analyses using MAGMA v1.10 (ref. ^22^) (Supplementary Table 21 and Supplementary Results 10). For BE-BROAD, we identified 22 Bonferroni-significant genes (*P* < 2.59 × 10^−6^), nine of which (43%) were linked to the gene-dense locus on chromosome 3: 47–52 Mb. *FTO* had the strongest association (*P* = 2.8 × 10^−22^). Gene-set, drug-set and drug-class analysis yielded no significant results. For AN, we identified 76 significant genes (*P* < 2.58 × 10^−6^), mostly (54/76, 71%) from chromosome 3: 47–52 Mb. Gene-set analysis implicated targets of RBFOX1-3 (RNA-binding proteins that regulate neuronal alternative splicing^23^) and mutation-constrained genes with a probability of loss-of-function intolerance (pLI) >0.9. No significant drug sets emerged, but antimigraine preparations as a class were significantly associated with AN, consistent with previous associations between AN and migraine polygenic risk scores (PRSs)^24^.

Combined evidence from proximity and expression suggests greater functional relevance than proximity alone^25^. Restricting MAGMA gene-wise results to S-PrediXcan tissue-level significant genes prioritized 7 genes across four loci in BE-BROAD and 38 genes across eight loci in AN (Supplementary Table 22).

### Tissue and cell-type analyses

We used stratified LDSC^26^ to estimate h^2^_SNP_ enrichment for BE-BROAD and AN among genes specifically expressed in GTEx human tissues (Supplementary Results 11, Supplementary Fig. 21 and Supplementary Table 23) and cell types from the Human Brain Atlas^27,28^ (Supplementary Fig. 22 and Supplementary Table 24). After accounting for multiple testing, no associations were significant.

### Polygenic prediction

In leave-one-study-out (LOO) PRS analyses, BE-BROAD PRSs were significantly associated with a higher risk of BE-BROAD (average liability-scale variance of 0.32%) and AN PRSs were significantly associated with a higher risk of AN (average liability-scale variance of 2.32%; Supplementary Results 12, Supplementary Fig. 23 and Supplementary Table 25). PRSs from the European ancestry AN GWAS were positively associated with a higher risk of AN in East Asian ancestry studies from Korea and Japan (odds ratio (OR) 1.36, 95% confidence interval (CI) 1.09–1.70, *P* = 0.0066), explaining 1.3% of the variance (assuming a population prevalence of 1.5%). In cross-sex analyses, PRSs from females were positively associated with risk in males (BE-BROAD: OR 1.06–1.20, AN: 1.07–1.41), but the results were not consistently significant across studies owing to variable power (Supplementary Results 12, Supplementary Fig. 24 and Supplementary Table 26). BE-BROAD and AN PRS were elevated in BE-BROAD only, AN-only and combined BE-BROAD and AN case groups compared with controls (*P* ≤ 0.019). Both BE-BROAD groups typically had a significantly higher BE-BROAD PRS than the AN-only group, while both AN groups had a significantly higher AN PRS than the BE-BROAD only in one study but not in another (Supplementary Results 12, Supplementary Fig. 25 and Supplementary Table 27).

## Discussion

In this BE GWAS, we implicate six genomic loci. These have been previously associated with smoking^29^, risk-taking behavior^30^ and age at menarche^31^. An overlap between BE and impulse-control behaviors was further observed in positive SNP-*r*_g_ with smoking, general risk tolerance and problematic alcohol use. Loss of control is a key component of BE, and impulse-control behaviors have been associated with binge-type eating disorders clinically^32–34^. Our findings imply that BE shares genetic underpinnings with psychiatric disorders and impulse-control behaviors.

Loci associated with BE-BROAD have also been implicated in anthropometric traits, including a BMI-related signal near *FTO*^35,36^ that was not associated with AN or its subtypes. The *FTO* locus has been studied extensively, but the causal mechanism contributing to high BMI remains unclear^37^. Our results imply that the relationship between *FTO* and a high BMI could result partly from BE, consistent with previous findings that BE was related to *FTO* independent of BMI and could mediate the pathway between *FTO* and a high BMI^38^. Genetic correlations of BE with anthropometric traits and impulse-control behaviors mirror clinical observations in individuals with BE and suggest that the relationship of BE to these traits and behaviors partly reflects pleiotropy rather than being purely environmental or a consequence of BE itself.

For AN, we validated six previously identified loci, identified two additional loci and identified one locus for AN-R. The four single-gene loci identified by Watson et al.^6^ remained genome-wide significant, suggesting that genes located in these regions—*CADM1*, *MGMT*, *FOXP1*, *PTBP2*—may warrant further investigation in the etiology of AN^39^. The AN-R-identified locus narrowly missed genome-wide significance in AN (*P* = 5.89 × 10^−8^) and has previously been implicated in schizophrenia^40^. The locus contains several genes, but only *DLX1* was indicated by both proximity-based and expression-based gene mapping. The product of *DLX1* is differentially expressed in the brain and may be involved in several processes of neural development^41^. Further studies are needed to confirm that *DLX1* is implicated in AN-R, given the multigenic locus. Our gene-level results should be viewed cautiously, as greater power is needed to effectively fine-map associated loci and link causal variants to genes.

Despite increasing our effective sample size for AN by 64% since our previous freeze^6^, we identified only two additional loci and two previously implicated loci were no longer significant. Several added studies were population based and used more lenient case criteria compared with previous clinical diagnoses and targeted AN-specific recruitment^42^. Consistent with this, AN PRSs typically captured less variance in AN in new studies (Supplementary Results 12 and Supplementary Table 25). Genetic signal becomes more heterogeneous as GWAS sample sizes increase^43,44^, as our data reflect: although the lead AN-associated SNPs showed no evidence of heterogeneity (Supplementary Figs. 7–14), variant heterogeneity statistics (*I*^2^) were higher in our AN GWAS than in that by Watson et al.^6^ (Supplementary Results 13 and Supplementary Table 28). Nonetheless, our AN GWAS measured variants with greater precision (average variant ﻿SE of 0.0199 versus ﻿SE of 0.0255 in Watson et al.), indicating increased power despite increased heterogeneity (Supplementary Results 13).

Previously, we hypothesized that AN is a metabo-psychiatric disorder^6^—here, we investigated shared and distinct metabolic/anthropometric and psychiatric components across multiple eating disorder-related phenotypes. BE-BROAD shares genetic features with other psychiatric phenotypes, including significant SNP-*r*_g_ with psychiatric traits^6,7,24,45^. Notably, BE-BROAD showed no significant association with obsessive–compulsive disorder, whereas AN had a positive SNP-*r*_g_. BE-BROAD was positively genetically correlated with ADHD, while AN showed no significant association; however, this may result from an underlying positive SNP-*r*_g_ between ADHD and the psychiatric component of AN being nullified by negative SNP-*r*_g_ between ADHD and the component of AN shared with BMI.

We observed key differences for SNP-*r*_g_s with nonpsychiatric traits. We validated our previous finding of significant SNP-*r*_g_s between AN and metabolic-related traits^6^ but not with BE-BROAD. BE-BROAD displayed a negative SNP-*r*_g_ with age at menarche, while AN showed no genetic overlap. Observational studies find that a later age of menarche is associated with AN^46,47^; however, disentangling this from the effects of starvation and a low BMI is difficult. In previous research, early-onset AN was negatively associated with age at menarche but typical-onset AN was not^48^. An earlier age at menarche is associated with impulsive traits, such as substance use and risky behavior^46^, and with BMI^49^. Both impulsivity and BMI are often higher in those with BE^1,32,33^.

Striking differences were observed in anthropometric traits: eating disorders and their component features share genetic factors with anthropometric traits but these effects act in opposite directions depending on the presentation. Significant SNP-*r*_g_s between BE-BROAD and anthropometric traits were positive compared with the negative SNP-*r*_g_s observed in AN, consistent with previous research^24,45^. To investigate this further, we assessed BE-BROAD and AN after subtracting the genetic component each shares with BMI. The BMI component accounted for 12% of the genetic variance of BE-BROAD and 10% of AN, despite a low BMI being central to AN diagnosis. The low variance explained by the BMI component argues that neither our AN nor BE-BROAD GWAS is BMI GWAS by proxy. This is further supported by the *FTO* locus association with BE-BROAD, which is observed in AN-ascertained studies where affected individuals are likely to have a lower BMI than unaffected individuals. Consistent with previous literature^50^, we found no evidence for genetic overlap between persistent thinness and AN, indicating that the cognitive–behavioral component of AN distinguishes these low-BMI phenotypes genetically.

BMI is a blunt measure of body composition, with a partly behavioral etiology^26,51^ and a complicated relationship with eating disorders. The negative SNP-*r*_g_ between AN and BMI may be driven by genetic enrichment for AN risk in females with a lower BMI, rather than a uniform linear relationship across BMI^52^. Both BE and AN are heterogeneous, and subtypes may have differing relationships with BMI. More sophisticated analyses with a wider range of body composition measurements and eating disorder presentations (including atypical AN in the normal- or high-BMI range) will improve understanding.

We present a GWAS of BE, a large-scale investigation of AN and AN subtypes and chromosome X analyses for all phenotypes. We extend eating disorder genetic research beyond AN alone and demonstrate that BE is a psychiatric phenotype with distinctive genetic relationships with external traits. Individuals with AN were as genetically distant from individuals with BE as they were from unaffected individuals, emphasizing the distinct nature of these eating disorder-related phenotypes. Nonetheless, we highlight the following limitations. We focused on individuals of European genetically inferred ancestry, limiting generalizability. The analysis of PRSs in two small East Asian studies used European prevalence estimates, potentially introducing bias. Differences in population structure, genetic architecture and environmental factors, such as a lower average BMI in East Asia^53^, could influence cross-ancestry prediction of AN. Global populations need to be included in GWAS to strengthen genetic risk prediction and our understanding of eating disorders^13^.

Given the known diagnostic crossover between eating disorders, cross-sectional studies cannot account for later symptom emergence; for example, an individual with AN-R could develop AN-BP^2^. It is not possible to fully mitigate this limitation. However, (1) many studies included individuals well beyond the typical age of diagnosis, making new diagnoses or diagnostic crossover less likely; (2) hidden diagnostic crossover probably contributes to false negatives and underestimation of differences between GWAS, rather than introducing false positives^54^; and (3) our previous work shows that extremely high rates of diagnostic contamination (a similar effect to hidden diagnostic crossover) are needed to affect locus discovery^55^.

Despite our best efforts at harmonization, heterogeneity might exist within the phenotypes owing to factors such as distinct ascertainment methods. Ideally, a single approach such as clinic-based recruitment with structured diagnostic interviews would be used but is untenable given the need for large sample sizes. Also, our sample is mostly female and results may not generalize to those who are not female. Finally, although strongly associated with BE-BROAD and AN risk, PRSs remain weak predictors of BE-BROAD and AN status. Improving prediction accuracy will require combining PRSs with other risk factors.

We redress the historical focus on AN in genetic studies of eating disorders by identifying six genetic loci relating to BE-BROAD, validating six loci related to AN, reporting two additional loci and finding one locus related to AN-R. We demonstrate that BE is genetically related to other psychiatric phenotypes, with both shared and distinct patterns from AN, providing genetic substantiation of clinically observed comorbidity. The number of loci we implicate in BE-BROAD and AN is typical of early-phase GWAS studies^56^, motivating GWAS meta-analyses of all eating disorders (AN, bulimia nervosa, BE disorder and avoidant/restrictive food intake disorder) and transdiagnostic behaviors (for example, BE and restriction) to drive variant discovery and further refine our understanding of shared and unique genetic features that distinguish presentations and inform a genetically informed nosology of eating disorders^12^.

## Methods

### Ethics

This work is a secondary analysis of data from individual studies. We provide ethics and statements of informed consent (or exclusion from informed consent) and institutional review board approval for each study as a Supplementary Note. Data access information is presented in Supplementary Table 1.

### Summary of studies

Details of the ascertainment and definition of cases and controls for each study are presented in Supplementary Table 1. Broadly, we identified cases and controls on the basis of clinical diagnoses, diagnostic algorithms and/or self-report questionnaires^42^. AN (and its subtypes) is a diagnostic phenotype, requiring case participants to meet clinical or research diagnostic definitions. BE-BROAD is a symptom phenotype: cases either explicitly endorsed BE as a symptom or had a diagnosis requiring BE (specifically bulimia nervosa or BE disorder; Table 1). The BE/purging subtype of AN can be diagnosed without BE and so is not sufficient for inclusion as a BE-BROAD case. Controls had no history of BE nor of an eating disorder, where possible. If this information was unavailable, unscreened controls were included assuming minimal misclassified individuals in the control groups, given that the collective lifetime prevalence of eating disorders is ~5%^16^.

We included data from 14 previously analyzed^6^ studies from the Eating Disorders Working Group of the Psychiatric Genomics Consortium (PGC-ED) and 13 additional studies (Supplementary Table 1). These data were restricted to individuals of European ancestry owing to few non-European ancestry studies being available at the time of analysis—we included two studies with individuals of East Asian ancestry for follow-up cross-ancestry PRS analyses. Details on genetic ancestry definition are provided in the Supplementary Methods.

Data from studies providing individual-level data (*n* = 11) were combined with studies that contributed summary statistics (*n* = 16; Supplementary Table 1). Detailed descriptions of each of the studies are provided in the Supplementary Note. We included data if the total number of cases for any phenotype before quality control was >100. If cases for an individual phenotype were <50, we excluded that phenotype from analyses.

### Genotype quality control and imputation

Approaches to quality control and imputation differed between studies and are described in detail in the Supplementary Methods.

### Association analyses

For case–control studies of unrelated individuals, we used PLINK2 to conduct logistic regression using a study-appropriate number of genomic principal components to account for ancestry (Supplementary Table 4). For studies with related individuals or unbalanced case–control ratios, we used SAIGE^57^ or REGENIE^58^. Further details on study-specific aspects of association analysis are provided in the Supplementary Methods. All analyses were two-tailed. Post-GWAS processing and quality control is described in the Supplementary Methods.

### Meta-analysis and quality control

Using the postimputation module of Ricopili^59^, we performed inverse-variance weighted fixed-effect meta-analyses in METAL^60^, including assessing variant heterogeneity as *I*^2^ values (a comparison of variant heterogeneity between our AN GWAS and that of Watson et al.^6^ is included in the Supplementary Methods). We included all variants but primarily report results for variants with an imputation INFO score >0.6 and a minor allele frequency (MAF) ≥0.01. We defined independent significant SNPs as those with a genome-wide significant *P* value (*P* < 5 × 10^−8^) that were independent (*r*^2^ < 0.6) from each other. We defined significant genomic loci by merging linkage disequilibrium (LD) blocks of neighboring independent significant SNPs (<250 kb). We defined independent lead SNPs as independent significant SNPs independent of each other at *r*^2^ < 0.1. We defined independently associated SNPs (conditional on lead SNPs) at each locus using a stepwise conditional analysis performed using the Genome-wide Complex Trait Analysis software conditional and joint association analysis tool (GCTA-COJO)^61^, using one of our largest studies (usa2) as our LD reference.

### Female-only analyses and female-to-male polygenic risk scoring

We conducted a supplementary female-only GWAS for BE-BROAD and AN and generated female-only PRS using PRS-CS, which we applied on male-only datasets with sufficient data (that is, *n* case and *n* control >100). For the BE-BROAD female-only meta-analysis, all studies except usa1 and biov were included, and alsp, moba and ukd2 were included as male-only target studies. For the AN female-only meta-analysis, all studies except itgr, spa1, ukd1 and net2 were included, and ipsy, fngn and ukb2 were included as male-only target studies.

### SNP-based heritability and distinguishing polygenicity from other sources of inflation

We used LDSC^62^ to estimate SNP-based heritability (*h*^2^_SNP_). These estimates were transformed to the liability scale, assuming population prevalences as follows: BE-BROAD 4.5%^14^, BE-NARROW 3.5%^14^, AN 1.5%^16^, AN-R 0.8%^16,63^ and AN-BP 0.7%^16,63^. For all analyses using LDSC, we applied an LD reference panel from the European subset of the 1000 Genomes Project (1kGP), restricted to SNPs present in the HapMap 3 panel^64^. For *N*, we calculated the sum of effective *N* across all studies and specified 0.5 for sample prevalence^65^. All LDSC analyses, including tests of the difference of *h*^2^_SNP_ and SNP-*r*_g_ from 0 and from 1, were two-tailed tests of deviation from a chi-squared distribution with jack-knifed standard errors.

Test statistics from GWAS of a polygenic trait are expected to be inflated, but inflation may also be due to spurious SNP associations caused by population stratification and cryptic relatedness of study participants. We used statistics from LDSC^62^ to determine the source of inflation. Although the LDSC intercept is commonly used to distinguish polygenicity from spuriously inflated statistics, we calculated the attenuation ratio statistic, defined as (LDSC intercept − 1)/(mean of association chi-squares statistics − 1), which may be a less biased metric compared with the LDSC intercept^15^. We included variants with MAF ≥0.01 and INFO ≥0.6.

### Genetic relationship between traits

We used LDSC to calculate SNP-*r*_g_ with several aims. First, we assessed the genetic relationships among the five eating disorder-related phenotypes. Second, we calculated SNP-*r*_g_ between BE-BROAD/AN and 225 traits covering eight categories: (1) psychiatric trait or disorder, (2) substance use, (3) psychological/personality/behavioral, (4) anthropometric, (5) metabolism, (6) blood, (7) sociodemographic and (8) somatic trait or disease. We selected these traits from an internal catalog on the basis of their power (*h*^2^_SNP_ *Z*-score >4)^66^. We tested whether SNP-*r*_g_ differed from zero and applied a Bonferroni-corrected *P* value threshold of 2.20 × 10^−4^ on the basis of 225 traits. For significant SNP-*r*_g_ with BE-BROAD and/or AN, we compared the SNP-*r*_g_ between BE-BROAD and AN using the LDSC block-jackknife procedure (Bonferroni-corrected *P* value threshold of 2.20 × 10^−4^)^67^.

We used MiXeR v1.2.0 to conduct univariate and bivariate causal mixture modeling of BE-BROAD and AN, limited to autosomal variants with INFO ≥0.6 and MAF ≥0.01, and with the major histocompatibility complex (MHC) region (chromosome 6: 26–34 Mb) excluded^18^. MiXeR refines the interpretation of heritability and genetic correlation by extending LDSC, using GWAS summary statistics to model the number of causal variants underlying a trait (polygenicity) and the average contribution of each causal variant to heritability (discoverability). Bivariate MiXeR enables the number of shared causal variants between traits to be estimated and the extent to which they have the same direction of effect. We followed protocols and used LD reference files provided by the authors of MiXeR (https://github.com/precimed/mixer).

We used case–case GWAS (CC-GWAS) to test for allele frequency differences between BE-BROAD and AN cases (as opposed to traditional GWAS, which compares cases with controls)^17^. We included nonambiguous SNPs from HapMap 3 with INFO ≥0.6 and MAF ≥0.01. CC-GWAS calculates genetic distances between cases and controls of the two traits using equation (1),

1 $$\sqrt{m\,\times {F}_{\rm{ST},{\rm{causal}}}}.$$

In equation (1), *m* is the number of independent causal variants and *F*_ST,causal_ is the average normalized squared differences in allele frequencies based on liability-scale *h*^2^_SNP_, SNP-*r*_g_ and population prevalence. We assumed *m* = 10,000, consistent with psychiatric disorder polygenicity^17^ and set the BE-BROAD population prevalence to 4.5% (range 0.1–10%) and AN to 1.5% (range 0.1–4.3%). We defined independent CC-GWAS loci with PLINK 1.9 (--clump-p1 5e-8 --clump-p2 5e-8 --clump-r2 0.1 --clump-kb 3000) and defined a genome-wide significant SNP if *P* < 5 × 10^−8^ in the CC-GWAS ordinary least squares test and *P* < 10^−4^ in the CC-GWAS exact test.

### Influence of BMI

We used GWAS-by-subtraction^19^, an application of genomic structural equation modeling (genomic SEM)^68^, in R v4.3.1 (ref. ^69^) to estimate the proportion of variance in BE-BROAD and AN independent of BMI (Supplementary Methods). Latent genomic SEM factors model shared genomic covariance across traits, making results less influenced by spurious biases than would conditioning on phenotypic traits in a GWAS^70^. We used GWAS summary statistics from BE-BROAD, AN and BMI^35^.

We specified two latent variables as a function of BMI and (for example) BE-BROAD: a shared variable (‘BMI’) as ‘BMI = ~NA × BE-BROAD + start(0.4) × BMI’ and a non-BMI variable (‘non-BMI’) as ‘non-BMI = ~NA × BE-BROAD’ (Supplementary Fig. 26). In line with previous applications of GWAS-by-subtraction^19^, we set latent variable variance to 1 and covariance to 0 and constrained the model such that all (co)variance in BMI and BE-BROAD was captured by BMI and non-BMI. We used the diagonally weighted least squares estimator^68^. Additional computational settings are presented in Supplementary Table 30.

We regressed the two latent factors on individual SNPs, yielding a GWAS of the BMI and non-BMI factors. We used LDSC^62^ to calculate SNP-*r*_g_ of the non-BMI factor with all traits identified in initial SNP-*r*_g_s. As a sensitivity analysis, we restricted BE-BROAD to studies that were not ascertained for AN, reasoning that this might better capture BE behavior outside of AN. We applied the same GWAS-by-subtraction model on that selection of studies (Supplementary Table 11).

We also conducted two-sample Mendelian randomization analyses of BE-BROAD/AN with BMI, testing causal effects in both directions with two-tailed tests. We used SNPs in linkage equilibrium as genetic instruments, with *P* < 5 × 10^−6^ for BE-BROAD and AN and *P* < 5 × 10^−9^ for BMI. The instrument used for BMI was that suggested by the authors of the BMI GWAS^35^. We repeated analyses using the non-BMI factor GWAS of BE-BROAD and of AN. To test robustness to potential violations of the assumptions of Mendelian randomization, we conducted analyses using inverse-variance weighted analysis, MR-Egger, mode-based estimation and median-based estimation, implemented in R 4.3.2, using the packages TwoSampleMR, MendelianRandomisation and MR-PRESSO^69,71,72^ (Supplementary Methods). We determined the strength of association of our genetic instruments using *F* statistics^73^. We used Cochran’s *Q* statistic to test for instrument heterogeneity, with *P* < 0.05 indicating heterogeneity^74^. To investigate potential confounding via horizontal pleiotropy, we assessed the deviation of the MR-Egger intercept from 0 and performed a global bias test in MR-PRESSO. We excluded from the analysis SNPs identified by MR-PRESSO as pleiotropic. We ran further methods robust to heterogeneity, including the penalized weighted median estimator, the contamination mixture method and MR-Lasso^75^. We assessed results visually (Supplementary Methods).

### Identification of gene–tissue associations with eating phenotypes

We used S-PrediXcan^20^ to identify genetically regulated gene expression associated with our phenotypes. We tested the association of gene expression using available GTEx v8 MASHR^20,76,77^ and CommonMind DLPFC^78,79^ tissue models. MASHR-based PredictDB models use fine-mapping methods for selection of eQTLs included in the predictor models, improving prediction^20,76,77^. We included 45 GTEx v8 MASHR models, removing non-natural tissues (cell lines), tissues with *N* < 100 individuals (kidney cortex) and testis^80^. We performed liftover of our GWAS summary statistics to hg38, harmonization and imputation on the basis of recommended preprocessing by Barbeira et al.^78^ using GWAS tools (https://github.com/hakyimlab/MetaXcan/wiki/Best-practices-for-integrating-GWAS-and-GTEX-v8-transcriptome-prediction-models and https://github.com/hakyimlab/summary-gwas-imputation/wiki/GWAS-Harmonization-And-Imputation). We tested whether imputed differences in genetically regulated gene expression between cases and controls differed from 0 as a two-tailed *Z* test. We used two different Bonferroni significance thresholds: an experiment-wide threshold, correcting for 600,382–602,744 tests performed across all tissues (*P* < 0.05/Tests_Total_ = *P* < 8.32 × 10^−8^), and a tissue-specific threshold, correcting for varying numbers of tests performed within each tissue (*P* < 0.05/Tests_Tissue *X*_; Supplementary Table 16). We performed two-tailed exact binomial tests for tissue enrichment using binom.test() in R for associations at three different significance thresholds: experiment-wide significance (*P* < 0.05/Tests_Total_), tissue-specific significance (*P* < 0.05/Tests_Tissue *X*_) and nominally significance (*P* < 0.05).

S-MultiXcan measures the joint association of genetically regulated gene expression across tissues with a phenotype of interest using summary statistics, leveraging shared eQTLs across tissues^21^. Using our GTEx v8 MASHR S-PrediXcan results as input, we ran S-MultiXcan on each of our phenotypes for all genes (*N* = 22,241). S-MultiXcan returns the *P* value for association of multi-tissue gene expression with the trait of interest (S-MultiXcan *P*), along with best single-tissue *P* value, both from two-tailed tests. To account for potential false positive associations, we removed any significant S-MultiXcan associations where the single best tissue *P* value was >1 × 10^−4^ and Bonferroni-corrected for all genes tested (*P* < 0.05/22,241 = 2.25 × 10^−6^)^21^.

### Gene-wise and gene-set analysis, including drug-target and drug-class analyses

Following previous publications^81^, we used MAGMA v1.10 (ref. ^22^) to test the association between each phenotype and the aggregate effect of SNPs mapped to protein-coding genes (gene analysis); groups of genes with shared functional, biological or other characteristics (gene-set analysis); sets of genes targeted by drugs (drug-set analysis) and signal enrichment within drug classes. We used SNPs with MAF ≥0.01 and INFO ≥0.6 present in ≥80% of the total sample and ≥50% of studies. We mapped SNPs to protein-coding genes, including variants 35-kb upstream and 10-kb downstream of hg19 gene positions from Ensembl release 75 (ref. ^82^). We obtained gene-wise *P* values with the multi-SNP-wise model (Supplementary Methods). We tested 19,332–19,418 ENSEMBL genes across the five phenotypes and applied a Bonferroni correction of *P* < 2.60 × 10^−6^. We used the 1kGP reference panel for estimating between-SNP LD.

For gene-set analyses, we applied a competitive analysis (one-tailed test; Supplementary Methods). We defined biological pathways on the basis of Gene Ontology and canonical pathways from MSigDB v6.1 and psychiatric pathways identified from literature. We tested 7,324–7,325 pathways across the five phenotypes and applied a Bonferroni correction of *P* < 6.83 × 10^−6^.

For drug-set analyses, we defined drug sets on the basis of drug targets from the Drug–Gene Interaction database DGIdb v4.2.0^83^, the Psychoactive Drug Screening Database Ki DB^84^, CheMBL v27^85^, the Target Central Resource Database v6.7.0^86^ and DSigDB v1.0^87^, all downloaded in October 2020. We applied a competitive analysis and subsequently grouped the results on the basis of the Anatomical Therapeutic Chemical class of the respective drugs^88^. For drug-class analysis, we determined statistical significance from one-tailed Wilcoxon Mann–Whitney tests of the area under the receiver operating characteristic curve of ranked drug–gene sets (Supplementary Methods). We applied a Bonferroni correction of *P* < 3.23 × 10^−5^ (on the basis of 1,546–1,547 drug sets) for the drug-set analysis and *P* < 3.08 × 10^−4^ (on the basis of 162 drug classes) for the drug-class analysis to account for multiple testing.

### Tissue and cell-type specific analyses

Tissue and cell-type specific analyses are described in the Supplementary Methods.

### Polygenic prediction

We used PRS-CS^89^ to generate PRS for BE-BROAD and AN. The inclusion of target studies for each phenotype was based on the effective sample size (*N*_eff_ half >1,000), study characteristics and availability of individual-level data (more detailed information provided in the Supplementary Methods). We generated LOO GWAS summary statistics, excluding each target study, and used these as the base data to calculate individual-level PRS in each target study. We included nonambiguous SNPs with INFO ≥0.6 and MAF ≥0.01. We used the 1kGP Phase 3 EUR LD reference panel and provided median sample size per LOO meta-analysis as input for PRS-CS. Posterior SNP effect size estimates from PRS-CS were combined across chromosomes to calculate individual PRS via PLINK (--score 2 4 6 sum)^90^. We standardized individual PRSs in R (version 4.3.2)^69^. We performed two-tailed logistic regression of BE-BROAD and AN PRS on BE-BROAD and AN, adjusting for study-specific genetic principal components. ORs reflected one standard deviation increase in the PRS. We assessed the proportion of variance explained by the standardized PRS for each phenotype as the Nagelkerke’s pseudo-*R*^2^ of the full model minus that of the null model excluding the PRS^91^, converted to the liability scale using population prevalences of BE-BROAD and AN as used for LDSC^92^. We also divided individuals into PRS deciles and assessed their relative risk of BE-BROAD and AN compared with the lowest PRS decile. We examined the sensitivity, specificity and precision of each PRS to predict BE-BROAD and AN status using the pROC^93^ and pracma^94^ packages in R, comparing the area under the receiver operating characteristic curve and under the precision recall curve of the full model against the null model.

We used female-only GWAS summary statistics as base data in PRS-CS^89^ to assess whether female BE-BROAD PRS was associated with BE-BROAD risk in males in three studies (alsp, moba, ukd2, *N*_case_male_total_ = 1,055, *N*_control_male_total_ = 38,046). Parallel analysis of the association of female AN PRS with male AN risk was conducted in three studies (ukb2, fngn, ipsy, *N*_case_male_total_ = 1,524, *N*_control_male_total_ = 388,891).

We assessed whether BE-BROAD PRS and AN PRS differed across subgroups, including (1) those with BE-BROAD only, (2) those with both BE-BROAD and AN and (3) those with AN only. We selected aunz and sedk to assess individuals with both BE-BROAD and AN and to assess the AN only group. We selected ukb2 and ukd2 for assessing BE-BROAD and AN PRS levels in all subgroups (Supplementary Methods and Supplementary Table 1). We performed linear regression on each PRS for each subgroup compared with controls, adjusting for study-specific genetic principal components. We compared differences in BE-BROAD PRS and AN PRS between subgroups and controls and across different subgroups.

We applied AN PRS from our European genetic ancestry meta-analysis to two East Asian genetic ancestry studies (Japanese: *N*_case_ = 77, *N*_control_ = 117, Korean: *N*_case_ = 75, *N*_control_ = 109). Raw genetic data from both studies were merged, and pre-imputation quality control and imputation were conducted as for the European ancestry studies. We used the 1kGP Phase 3 EUR LD reference panel for PRS, which aligns with the ancestry of the base GWAS^89^. We used European AN prevalence estimates for liability scale conversion, as what sparse estimates exist from East Asia approximately align with estimates in countries with European genetic ancestry majorities^95^.

### Sensitivity analyses using down-sampled BE-BROAD data

A considerable portion (13%) of BE-BROAD cases were recruited through studies ascertained for AN. To understand the influence of AN on this BE-BROAD phenotype, we conducted an additional BE-BROAD meta-analysis excluding studies ascertained for AN (Supplementary Table 11). We calculated SNP-*r*_g_ to compare the genetic relationship of the ‘not-ascertained-for-AN’ BE-BROAD GWAS and the original BE-BROAD GWAS with traits significantly correlated with the original BE-BROAD GWAS or with the AN GWAS (hypothesizing that AN-related effects may mask or drive SNP-*r*_g_s between such traits and the original BE-BROAD GWAS).

### Reporting summary

Further information on research design is available in the Nature Portfolio Reporting Summary linked to this article.

## Supplementary information

Supplementary Information Supplementary Note (cohort descriptions), Methods, Results 1–14, Figs. 1–28 and References.

Reporting Summary

Peer Review File

Supplementary Tables 1–31 Supplementary Tables 1–31.

## Data Availability

Data access is described in Supplementary Table 1. Available individual-level genotype data (except in countries where sharing of individual-level data is prohibited by national law) and summary statistics from individual studies used in this study are available to researchers via collaboration with study principal investigators (PIs) or (for a subset of studies) via a secondary analysis proposal to the Eating Disorders Working Group of the Psychiatric Genomics Consortium (PGC) at https://pgc.unc.edu/for-researchers/data-access-committee/data-access-information/. Summary statistics of all GWAS meta-analyses reported in this work are available via Figshare and the PGC website, conditional on agreeing to terms and conditions of use, at https://pgc.unc.edu/for-researchers/download-results/.

## Code availability

Code underlying this work are available via GitHub at https://github.com/psychiatric-genomics-consortium/PGC3_EAD_Public.
