# Supplementary material for: Genomic meta-analyses of binge-eating behavior and anorexia nervosa yield insights into the unique and shared biology of eating disorder phenotypes
Source: Nat Ment Health. 2026 Aug 19;4(9):1361–77. doi: 10.1038/s44220-026-00698-2 (PMC13553282; doi:10.1038/s44220-026-00698-2)
Supplement: Supplementary file 1 [file 44220_2026_698_MOESM1_ESM.pdf]

nature mental health

Supplementary information https://doi.org/10.1038/s44220-026-00698-2

Genomic meta-analyses of binge-eating behavior and anorexia nervosa yield insights into the unique and shared biology of eating disorder phenotypes

In the format provided by the authors and unedited

Supplementary Material: *"Genome-wide association studies of binge-eating behaviour and anorexia nervosa yield insights into the unique and shared biology of eating disorder phenotypes"*, Termorshuizen, Davies, Lee et al.

Cohort descriptions 5
  Summary 5
  GCAN/WTCCC-3 6
  chop 8
  ANGI & BEGIN 9
    aunz 10
    ipsy 11
    sedk 12
    usa2 14
    sebe 15
  Other cohorts 16
    agds 16
    alsp 17
    biov 18
    esbb 19
    fngn 20
    gcfm 21
    jans 22
    moba 23
    net2 25
    ukb2 26
    ukch 27
    ukd2 28
    jpan 30
    kran 31
Supplementary Methods 32
  Genotype quality control 32
  Imputation 33
  Association analyses and post-analysis processing and quality control 34
  Cohort by cohort deviations from established quality control and GWAS protocol 35
    gns2 35
    net1 35
    poco 35
    spa1 35
    ukd1 36
    usa1 36
    aunz 36
    ipsy 36
    usa2 37
    agds 37
    esbb 37
    fngn 37
    ukb2 38
    ukch 38
    ukd2 38
  Power analysis 38
  Polygenic prediction 39
    Cohort selection 39
  Sex-specific analyses 40
    Cohort selection 40

Causal mixture modelling 41
Mendelian randomisation 42
Variant heterogeneity in AN 44
Gene-wise and gene set analysis, including drug target and drug class analyses 45
Tissue and cell-type specific analyses 46
Supplementary Results 47
1. Comparison of the two binge-eating behaviour phenotypes 47
2. Association meta-analyses 47
BE-NARROW 47
AN subtypes 48
3. Comparison of BE-BROAD with Burstein et al. 48
4. Female-only analyses 49
BE-BROAD 49
AN 50
5. Causal mixture modelling 50
6. BE-BROAD in cohorts not ascertained for anorexia nervosa ($BE\text{-}BROAD_{NAAN}$) 51
7. Influence of BMI 52
GWAS-by-subtraction 52
Mendelian randomisation 55
8. S-PrediXcan 58
BE-BROAD 58
AN 59
BE-NARROW 59
AN subtypes 59
9. S-MultiXcan 60
BE-BROAD 60
BE-NARROW 60
AN 60
AN subtypes 61
10. MAGMA 61
BE-NARROW 61
AN subtypes 61
11. Cell and tissue gene expression specificity 62
12. Polygenic prediction 62
Association 62
Liability-scale R^2 63
PRS predictive performance 64
Sex-specific PRS 66
Subgroup PRS 67
13. Variant heterogeneity in AN 68
14. Power analyses (AN-R, AN-BP, BE-NARROW) 68
Supplementary Figures 69
Supplementary Figures 1-6: Forest plots for the six genome-wide significant associations with BE-BROAD in order of position on the genome 69
Supplementary Figure 1 70
Supplementary Figure 2 71
Supplementary Figure 3 72
Supplementary Figure 4 73
Supplementary Figure 5 74
Supplementary Figure 6 75
Supplementary Figures 7-14: Forest plots for the eight genome-wide significant associations with AN in order of position on the genome 76
Supplementary Figure 7 77
Supplementary Figure 8 78
Supplementary Figure 9 79

Supplementary Figure 10 ... 80
Supplementary Figure 11 ... 81
Supplementary Figure 12 ... 82
Supplementary Figure 13 ... 83
Supplementary Figure 14 ... 84
Supplementary Figure 15: Manhattan plots ... 85
a) BE-BROAD (39,279 cases, 1,227,436 controls) ... 85
b) AN (24,223 cases, 1,243,971 controls) ... 85
c) BE-NARROW (15,175 cases and 1,217,725 controls) ... 86
d) AN-R (2524 cases, 33,374 controls) ... 86
e) AN-BP (5209 cases, 33,951 controls) ... 87
Supplementary Figure 16: Significant differences between the genetic correlations (r_g) of external traits with BE-BROAD and AN ... 88
a) Significant differences ... 88
b) Non-significant differences ... 89
Supplementary Figure 17: Comparison of genetic correlations (r_g) of selected external traits with not-ascertained-for-AN BE-BROAD (BE-BROAD$_{NAAN}$) and BE-BROAD ... 90
Supplementary Figure 18: Comparison of the genetic correlations of BE-BROAD and AN and their respective NonBMI components with external traits ... 91
Supplementary Figure 19: Gene-tissue associations from S-PrediXcan ... 92
a) BE-BROAD (39,279 cases, 1,227,436 controls) ... 92
b) AN (24,223 cases, 1,243,971 controls) ... 93
c) BE-NARROW (15,175 cases and 1,217,725 controls) ... 94
d) AN-R (2524 cases, 33,374 controls) ... 94
e) AN-BP (5209 cases, 33,951 controls) ... 95
Supplementary Figure 20: Cross-tissue gene-wise associations from S-MultiXcan ... 96
a) BE-BROAD (39,279 cases, 1,227,436 controls) ... 96
b) AN (24,223 cases, 1,243,971 controls) ... 96
c) BE-NARROW (15,175 cases and 1,217,725 controls) ... 97
d) AN-R (2524 cases, 33,374 controls) ... 97
e) AN-BP (5209 cases, 33,951 controls) ... 98
Supplementary Figure 21: Tissue analyses in GTEx human tissues ... 99
Supplementary Figure 21a ... 99
Supplementary Figure 21b ... 100
Supplementary Figure 22: Cell-type analyses in human brain cell types ... 101
Supplementary Figure 22a ... 101
Supplementary Figure 22b ... 102
Supplementary Figure 23: Within-and-cross-trait polygenic scoring ... 103
Supplementary Figure 23a: Decile plots and area under the receiver operating characteristic (AU-ROC) curve for BE-BROAD PRS with BE-BROAD ... 104
Supplementary Figure 23b: Decile plots and AU-ROC curve for AN PRS with AN ... 105
Supplementary Figure 23c: Decile plots and AU-ROC curve for BE-BROAD PRS with BE-NARROW ... 107
Supplementary Figure 23d: Decile plots and AU-ROC curve for BE-BROAD PRS with AN ... 108
Supplementary Figure 23e: Decile plots and AU-ROC curve for BE-NARROW PRS with BE-NARROW ... 110
Supplementary Figure 23f: Decile plots and AU-ROC curve for BE-NARROW PRS with BE-BROAD ... 111
Supplementary Figure 23g: Decile plots and AU-ROC curve for BE-NARROW PRS with AN ... 112
Supplementary Figure 23h: Decile plots and AU-ROC curve for AN PRS with BE-BROAD ... 114

Supplementary Figure 23i: Decile plots and AU-ROC curve for AN PRS with BE-NARROW 115
Supplementary Figure 24: Cross-sex polygenic scoring 116
Supplementary Figure 24a: Decile plots, AU-ROC curve, and area under the precision-recall (AU-PR) curve for female BE-BROAD PRS with BE-BROAD in males 117
Supplementary Figure 24b: Decile plots, AU-ROC curve, and AU-PR curve female AN PRS with AN in males 118
Supplementary Figure 25: Polygenic scoring across comorbid AN and BE-BROAD, BE-BROAD-only, and AN-only subgroups 119
Supplementary Figure 26: Graphic representation of the GWAS-by-subtraction model 120
Supplementary Figure 27: Forest and scatter plots from Mendelian randomisation analyses with BE-BROAD 121
Supplementary Figure 27a 122
Supplementary Figure 27b 122
Supplementary Figure 27c 123
Supplementary Figure 27d 123
Supplementary Figure 27e 124
Supplementary Figure 27f 124
Supplementary Figure 27g 125
Supplementary Figure 27h 125
Supplementary Figure 28: Forest and scatter plots from Mendelian randomisation analyses with AN 126
Supplementary Figure 28a 127
Supplementary Figure 28b 127
Supplementary Figure 28c 128
Supplementary Figure 28d 128
Supplementary Figure 28e 129
Supplementary Figure 28f 129
Supplementary Figure 28g 130
Supplementary Figure 28h 130
References 131

Cohort descriptions

Summary

The current analysis includes data that have been previously published in combination with new datasets. Previously published datasets include:

- Case-only data from the Genetics Consortium for Anorexia Nervosa/Wellcome Trust Case Control Consortium-3 (GCAN/WTCCC-3) as described in Watson et al [1]. These data were first included in Boraska et al [2], and thereafter in Duncan et al [3]. Furthermore, we included the control data for these datasets as described in Watson et al [1]. We refer to these (10) datasets as follows: *fin1, fre1, gns2, itgr, net1, poco, spa1, ukd1, usa1.* The dataset *gcfm* is part of GCAN/WTCCC-3 but has not been previously published.
- Case-control data from the Children's Hospital of Philadelphia/Price Foundation Collaborative Group (CHOP/PFCG) (dataset *chop*), first published in Wang et al [4], and thereafter included in Duncan et al [3] and Watson et al [1].
- Datasets including case-control data from the Anorexia Nervosa Genetics Initiative (ANGI) studies from different sites have partly been previously published in Watson et al [1], and partly updated. Here, we included ANGI-Australia and New Zealand (dataset *aunz*), ANGI-Denmark (dataset *ipsy*), ANGI-Sweden (part of dataset *sedk*), and ANGI-United States (dataset *usa2*) as described in Watson et al [1].
- Part of the UK Biobank dataset (*ukb2*) has been previously published in Watson et al [1].

The remaining datasets (*agds*, *alsp, biov, esbb, fngn, gcfm, jans, moba, net2, sebe, ukch, ukd2*) include data that have not been previously published as part of a GWAS.

Non-cohort funding statements: Katherine Schaumberg is supported by NIH K01MH123914. Karanvir Singh is supported by the Canadian Institutes of Health Research.

Jiayi Xu is supported by NIMH R01MH136149, R01MH124839, R01ES033630. Jerry Guintivano reports funding from NIMH K01 MH116413.

GCAN/WTCCC-3

GCAN/WTCCC-3 is short for “Genetics Consortium for Anorexia Nervosa / Wellcome Trust Case Control Consortium-3” [2]. In 2007, the Genetic Consortium for Anorexia Nervosa (GCAN) was established to combine existing genetic samples from individuals with AN into a single resource. GCAN contains data from different countries, and study participants were recruited through clinical sites or existing studies.

In the current publication, the following datasets contain GCAN cases: *fin1, fre1, gcfm, gns2, itgr, net1, poco, spa1, ukd1, and usa1*. These case-only data were first included in Boraska et al [2]. As the GCAN consists of cases only, these were combined with a separate set of controls which were sourced as described in Duncan et al [3] (cohorts *fin1, fre1, itgr, net1, spa1, ukd1, usa1*). The Swedish cases have been merged with German and Norwegian cases, forming the dataset *gns2*.

To summarise, a set of PGC controls was selected based on Illumina genotyping and geographical location. Then, we performed quality control to identify suitably matched controls of European genetic ancestry (Supplementary Methods). It is important to note that some of the controls were not screened for AN which, although ideal, was not feasible. This could potentially result in an attenuation of the GWAS signal because of misclassification; however, we do not anticipate false results because of the low prevalence of AN.

Some GCAN cohorts were not included in Duncan et al. [3] (*gcfm*, *gns2*, *poco*) due to a small number of cases (Sweden, N<100) or a lack of controls (Poland) but were included in Watson et al [1] and are also part of the current study. A detailed description is given in Watson et al [1]. Briefly, study participants consisted of females only and were diagnosed with AN via a semi-structured or structured interview based on the DSM or via DSM-based

population assessments. Cases consisted of individuals with a lifetime DSM-IV diagnosis of AN (restricting or binge-purge subtype) or lifetime DSM-IV EDs ‘not otherwise specified’ AN subtype (i.e., all criteria of AN without amenorrhea OR all criteria for AN except that, despite significant weight loss, the weight is in the normal range). Individuals were excluded if medical or psychiatric conditions were present that could have confounded a diagnosis of AN (i.e., conditions causing weight loss, schizophrenia, or intellectual disability). Controls for the Polish sample were gathered from a hospital-based case-control sample and a population-based case-control sample in Łódź and Warsaw for a study of upper aerodigestive tract cancers [5]. Controls were unscreened for psychiatric phenotypes and were included in the dataset *poco*.

Acknowledgements: Fernando Fernández-Aranda thanks CERCA for its institutional support.

Ethics statement: Each study site obtained informed consent from all participants, as well as permission from local ethical committees.

Funding statement: This study was funded by the Wellcome Trust Case Control Consortium 3 (WTCCC3) WT088827/Z/09 (Collier/Bulik/Sullivan) A Genome-wide Association Study of Anorexia Nervosa. *Spa1* was partially funded by the European Union’s Horizon Europe innovation programme, under grant agreement No. 101080219 (eprObes, Early Prevention of Obesity). *Spa1* was also funded by Instituto de Salud Carlos III (ISCIII) (FORT23/00032), co-funded by FEDER funds/European Regional Development Fund (ERDF), a way to build Europe. CIBERobn is an initiative of ISCIII. Additional funding for *spa1* was received by AGAUR-Generalitat de Catalunya (2021-SGR-00824). *Gns2* was supported by the German Research Foundation collaborative research center grant (DFG, SFB 940/3) bzw. neuerdings “SFB940 TP C03” (laut Mail von Solveig Otto an Julius vom 06.01.2022). DFG research grant: "Hormonal modulation of neural networks in anorexia nervosa" EH 367/5-1 (PI S. Ehrlich). DFG research grant: EH 367/7-1 "Dynamische

Veränderungen des strukturellen und funktionellen Hirn-Konnektoms bei Patientinnen mit Anorexia Nervosa" (PI S. Ehrlich), the Schweizer Anorexia Nervosa Stiftung and the B. Braun-Stiftung; Marga und Walter Boll-Stiftung. Anke Hinney reports support from Deutsche Forschungsgemeinschaft (DFG, HI 865/2-1), BMBF (01GS0820). Lars Alfredsson reports funding from the Swedish Research Council, Swedish Brain Foundation, Swedish Council for Health, Working Life and Welfare. Stéphanie Le Hellard reports support from the Trond Mohn Fondation. *Fre1* was supported by EC framework V 'Factors in healthy eating'. *Poco* was part supported by the Ministry of Health of the Czech Republic under grant number NU22-04-00010 and NU23-04-00381, by the Cooperatio Program (research areas Neuroscience and Health Sciences), and by the project MH CZ – DRO VFN64165. James Kennedy reports support from the Larry and Judith Tanenbaum Family Foundation. Stephen Scherer is the Northbridge Chair in Paediatric Research, and reports support from the SickKids Foundation.

chop

This dataset combines two different cohorts: (1) Cases obtained from the Price Foundation Collaborative Group (PFCG) [6,7], and (2) controls obtained from the Children's Hospital of Philadelphia (CHOP) [4]. The PFCG was established in 2000 through collaboration between The Price Foundation, the University of Pittsburgh, and other universities in North America and Europe [6]. Cases were recruited from different sites based on ED assessment experience, number of records of individuals treated for AN, and the geographical distribution (rationale described in more detail in Kaye et al [6]). Included study sites were Cornell University, University of California at Los Angeles, University of Pittsburgh, University of Toronto, University of London, and University of Munich.

Cases included individuals with a DSM-IV diagnosis of AN with or without amenorrhea via the Structured Interview of Anorexia nervosa and Bulimic Syndromes (SIAB) [8]. Furthermore, cases had to be aged between 13 and 65 years and have fulfilled the

AN criteria for at least three years prior to ascertainment. Individuals were excluded if the onset of AN was more than 25 years previously, or if they had a lifetime history of any of the following: organic brain syndrome; binge ED; “regular” binge eating (i.e., binge eating at least once weekly for three or more consecutive months); a medical illness that could affect appetite, eating behaviour, or body weight; IQ<70; dementia; schizophrenia; bipolar illness; obesity.

Unscreened controls were recruited by CHOP clinicians and medical staff. The recruiters were part of the CHOP Health Care Network, which includes multiple outpatient practices and primary care clinics. The controls were paediatric; nonetheless, we assume that the loss of power related to the few potential cases that might develop AN will not significantly influence the current analysis because of the low prevalence of AN. The controls had a mean (SD) age of 12.75 (4.2) years. Multidimensional scaling was used to confirm a proper match of genetic backgrounds.

Ethics statement: Ethical approval was obtained by each of the individual study sites (PFCG). For the CHOP controls, CHOP’s Research Ethics Board approved the study. Informed consent was obtained from all participants or their parents.

Funding statement: Funded by the Price Foundation. Andrew Bergen reports funding from Price Foundation Collaborative Group and NIH R01DA044014 and R01DA050495.

ANGI & BEGIN

The Anorexia Nervosa Genetics Initiative (ANGI) was a multi-site effort to collect biological samples and clinical information from individuals with AN and healthy controls [9]. Prof. Cynthia M. Bulik was the lead investigator, based at the University of North Carolina at Chapel Hill (dataset *usa2*), and collaborated with sites in Sweden (dataset *sedk*, PI: Dr. Mikael Landén), Denmark (dataset *ipsy*, PI: Prof. Preben Bo Mortensen), and Australia

combined with New Zealand (dataset *aunz*, PIs: Prof. Nick Martin, Prof. Martin Kennedy, Dr. Jenny Jordan). Generally, across all sites (except *ipsy*), potential cases were recruited via professional organisations such as the Academy for Eating Disorders, conventional media, social media, (press) conferences and support groups, and word-of-mouth. The studies were designed as such to attract individuals who are most likely to fulfil the case criteria.

The Binge Eating Genetics INitiative (BEGIN) is a similarly designed follow-up study of ANGI, performed in the US and Sweden, and is focused on recruiting individuals with EDs other than AN, particularly BN and BED (dataset *sebe* [10]). This is an ongoing project—initial data from Sweden only are included in this paper.

aunz

A detailed description of the Australia and New Zealand arm of ANGI is provided by Kirk et al., 2017 [11]. Residents from Australia (≥13 years) and New Zealand (≥14 years) were recruited between May 2013 and July 2016 and presented with an online questionnaire that was adapted from the Structured Clinical Interview for DSM-IV, section H (SCID-H). Amenorrhea was not required for being included as an AN case in the study. Controls were recruited via the QSkin Sun and Health Study [12]. The QSkin Sun & Health Study was established in 2010 to assess risk factors for skin cancers and is a randomly sampled cohort of individuals between 40 and 69 years residing in Queensland. The study participants were asked at the time of saliva collection whether they had ever been diagnosed with or treated for bipolar disorder, schizophrenia/psychosis, depression, obsessive-compulsive disorder, bulimia, anorexia nervosa, attention deficit hyperactivity disorder, or autism. Those who indicated no ED history were included as controls.

Ethics statement: For ANGI-ANZ, all participants provided informed consent. Ethical approval for the Australian arm of the study was obtained by the QIMR Berghofer Human Research Ethics Committee; for New Zealand, this was provided by the Health and Disability

Ethics Committee of the New Zealand Ministry of Health. For QSkin, ethical approval was obtained by the QIMR Berghofer Human Research Ethics Committee (QIMR-HEC approval P1309, P2034).

Funding statement: The Anorexia Nervosa Genetics Initiative was an initiative of the Klarman Family Foundation. The QSkin Study is supported by a Clinical Trials and Cohort Grant [APP1185416] from the National Health and Medical Research Council of Australia (NHMRC) and was previously supported by grants APP1073898, APP552429, APP1063061. Jenny Jordan reports support from the Klarman Foundation, the University of Otago Research Fund, and the NIH. Tracey Wade reports support from the National Health and Medical Research Council Project Grant 480420.

ipsy

This dataset consists of cases and controls recruited via ANGI Denmark (ANGI-DK) plus additional cases and controls from the Lundbeck Foundation Initiative for Integrative Psychiatric Research (iPSYCH, https://ipsych.dk/) [13]. The recruitment approach for ANGI-DK was through the Danish national registers. The Danish Civil Registration System [14] was established in 1968 and allows for the linkage of the national population registers. Everyone born between 1981 and 2005, who was a resident of Denmark on their first birthday, and who had a known mother was considered. Individuals in the Danish Psychiatric Central Research Register [15] with a lifetime AN diagnosis (ICD-10 F50.0 or F50.1) were included as a case, and controls—matched on sex, date of birth, and without any major psychiatric disorder—were randomly selected from the same nationwide birth cohort. After identifying eligible cases and controls, their biological samples (phenylketonuria cards) were extracted from the Danish Neonatal Screening Biobank (DNSB) through their national identification number. Specifically, these biological samples consist of blood samples obtained by a heel prick postpartum, which were dried and subsequently stored at the DNSB. After extraction, the samples were genotyped on the Illumina PsychArray at the Broad Institute. Additional

cases and controls were recruited through iPSYCH and genotyped similarly to the individuals recruited through the national registers.

Ethics statement*:* Participants from ANGI-DK and the iPSYCH study did not provide written informed consent, as an exemption from consent was provided by the Danish Scientific Ethics Committee (Videnskabsetisk Komité). This exemption was most recently approved in 2018.

Funding statement: ANGI-DK was part of the Anorexia Nervosa Genetics Initiative, which was an initiative of the Klarman Family Foundation (Mortensen, local PI; Bulik, PI). The iPSYCH was supported by grants from the Lundbeck Foundation (R102-A9118; R155-2014-1724; R248-2017-2003) and the Universities and University Hospitals of Aarhus and Copenhagen. Zeynep Yilmaz is supported by Independent Research Fund Denmark (DFF; Sapere Aude no. 1052-00029B); DFF (grant no. 3166-00063B, 4309-00050B); Lundbeck Foundation Ascending Investigator (R434-2023-269). Liselotte Petersen and Janne Larsen report funding from the Lundbeck Foundation (grant no. R276-2018-4581, recipient Petersen) and NIMH (R01MH120170).

sedk

The *sedk* cohort merged case-control data from ANGI recruitment efforts in Sweden with additional case individuals from clinical sites in Denmark.

ANGI-Sweden

Individuals were recruited from four different sources: (1) the Swedish ED quality registers RIKSÄT and Stepwise [16]; (2) the Stockholm Centre for Eating Disorders (SCÄ); (3) the community via conventional media and social media including the ANGI website (https://angi.se/); (4) LifeGene [17], a study that was running from 2009 to 2019 to study how genes, environment, and lifestyle affect health. Most cases were recruited via the ED quality

registers and were approached with a letter asking them to complete a questionnaire including the ED100K (described below). Cases approached through SCÄ were identified by research nurses who discussed the study and reviewed the consent. After providing consent, the study participant received an online questionnaire.

The ED100K questionnaire—used to assess case status—is a self-report assessment based on the Structured Clinical Interview for the DSM-5 Eating Disorder chapter and assesses criteria for AN, BN, BED, and OSFED. Individuals who met the criteria for AN were included as AN cases and if information was available, these individuals were subsequently assessed for AN subtypes (AN-R and AN-BP). Individuals who met the criteria for BN or BED were included as BE-NARROW and BE-BROAD cases. Individuals who have or have ever had regular episodes of binge eating with loss of control were included as BE-BROAD cases. Participants from LifeGene completed an online questionnaire that was similar to the ED100K algorithm and were included as cases if they met self-report criteria as defined above, or as controls if they screened negative for a history of EDs.

Ethics statement: ANGI-Sweden has been approved by the ethical review board in Sweden (dnr 2013/112-31/2). For LifeGene, the Regional Ethical Review Board of Stockholm provided ethical approval, and all participants provided informed consent online.

Funding statement: ANGI-Sweden was part of the Anorexia Nervosa Genetics Initiative, which was an initiative of the Klarman Family Foundation (Landén local PI; Bulik, PI). Cynthia Bulik is supported by the U.S. National Institute of Mental Health (R01MH136149;R01MH134039,R56MH129437; R01MH120170; R01MH124871; R01MH119084; R01MH118278; R01MH124871) and the Swedish Research Council (Vetenskapsrådet, award: 538-2013-8864). Patrick Sullivan reports support from the Swedish Research Council (Vetenskapsrådet, award D0886501), and NIMH R01 MH124871. Ruyue Zhang is supported by the Swedish Research Council (Vetenskapsrådet)

grant no. 2022-00242. Andreas Birgegård reports funding from the Swedish Research Council (Vetenskapsrådet) grant no. 538-2013-8864, PI Bulik.

Danish clinical cases

Women born between 1947 and 1980 with at least one recorded hospital admission with an ICD-10 code of F50.0 or F50.1 identified through the Danish Psychiatric Biobank were included as AN cases.

Ethics statement: All participants provided written informed consent before participating in the study. The study was approved by the Capital Region of Denmark's Committees on Health Research Ethics (De Videnskabsetiske Komiteer for Region Hovedstaden) (approval number H-KF-01-024/01).

Funding statement: Genotyping of these cases was supported by the Anorexia Nervosa Genetics Initiative, which was an initiative of the Klarman Family Foundation (Werge, local PI; Bulik, PI).

usa2

Cases and controls were recruited as described under ANGI, and additional controls were sourced from the Price Foundation AN Trios Study [18,19]. Case recruitment focused on reaching not just those in treatment for AN but also those who never received treatment. Potential participants (aged ≥12 years) either self-identified or were referred to take part in the study and received a brief screener to determine their eligibility for the study. Those interested and eligible progressed to sign an informed consent and online questionnaire (ED100K.v1). Upon completion of the questionnaire, participants provided blood samples in EDTA tubes which were drawn at a local laboratory, UNC, a physician's office, or through a mobile phlebotomy company. DNA was extracted at the Rutgers University Cell and DNA repository of the National Institute of Mental Health, and samples were genotyped with the

Illumina GSA chip. Furthermore, additional controls were recruited as part of the AN Trios study by advertisements in local communities of the various participating sites in the US and Europe. Individuals were included as controls if they were female, between the ages of 18 and 65 years, with a lifetime adult minimum BMI>19 and maximum BMI<27 kg/m^2, no history of an ED, and no first-degree relative with an ED. Controls were matched to cases by study site, age, ancestry, and education. Samples were genotyped on the Illumina GSA array at the Broad Institute.

Ethics statement: ANGI-US was approved by the Institutional Review Board of the University of North Carolina at Chapel Hill (IRB: 13-0081). All participants were broadly consented for research.

Funding statement: The Anorexia Nervosa Genetics Initiative was an initiative of the Klarman Family Foundation (Bulik, PI).

sebe

The Binge Eating Genetics INitiative (BEGIN) Sweden study includes individuals with a diagnosis of BN or BED in the Swedish ED quality register Riksät, or individuals who answered the ANGI questionnaire but were not eligible for ANGI [9]. Individuals who consented to partake in the study received a saliva sampling kit. Furthermore, additional controls who were matched for age, sex, and geographical region were recruited via Ipsos, a telemarketing company. All individuals were aged ≥18 years. Cases were ascertained similarly to ANGI Sweden (see *sedk* - ANGI-Sweden).

Controls received screening questions before participating in BEGIN. Individuals were not included as controls if: they had ever had or had ever been treated for an ED; if they had a close family member with an ED; if they had received weight loss surgery; or if they received hormonal treatment (excl. anticonception).

Ethics statement: This project has been approved by the ethical review board in Sweden. The fundament of the ethical permit comes from ANGI Sweden (dnr 2013/112-31/2) which has later been amended to also include individuals with another diagnosis than AN (dnr 2014/1563) and to change the study name to BEGIN (dnr 2016/1852-32). All participants provide informed consent.

Funding statement: Funding was from the Swedish Research Council Swedish Research Council (Vetenskapsrådet, award: 538-2013-8864) (Bulik, PI).

Other cohorts

agds

Cases were recruited via the Australian Genetics of Depression Study (AGDS) [20]. AGDS was established in Australia to recruit a cohort of individuals with lifetime depression and investigate genetic and environmental risk factors, as well as response to antidepressants. Individuals were recruited based on (1) a media publicity campaign and (2) a pharmaceutical prescription history of common antidepressant medications in the last 4.5 years. Recruitment was initiated in September 2016. Controls were recruited via the QSkin Sun & Health Study (see summary under *aunz*) [12].

AGDS study participants were asked to submit an online questionnaire. The question '*Have you ever been diagnosed with any of the following? Please select all that apply.*' was used to assign individuals a case status. This was one of the first questions in the extensive online questionnaire. Individuals who answered 'Yes' to 'Anorexia nervosa' were coded as cases for AN, and individuals who answered 'Yes' to 'Bulimia nervosa' as cases for BE-BROAD. These individuals were not included in the BE-NARROW definition because of the single-item self-report nature of this questionnaire.

Ethics statement: Ethics approval for all aspects of the project was obtained from the QIMR Berghofer Human Research Ethics Committee (P2118 & P3434). All participants provided broad informed consent for genetic research investigating mental illness.

Funding statement: The AGDS was primarily funded by National Health and Medical Research Council (NHMRC) of Australia grant 1086683. Sarah Medland is supported by NHMRC Leadership Grant APP1172917.

<u>*alsp*</u>

The Avon Longitudinal Study of Parents And Children (ALSPAC, https://www.bristol.ac.uk/alspac/) study is an ongoing population-based birth-cohort study of 14,541 mothers and their children (who were born between April 1, 1991 and December 31, 1992) residing in the southwest of England (UK). Criteria for defining cases (children and mothers) is detailed in Micali et al., 2015 [21]. Specifically, individuals with a diagnosis of AN at waves 14+ and 16+ were included as AN cases, and individuals who reported episodes of binge eating via the Youth Risk Behavior Surveillance System questionnaire [22] ("eating a very large amount of food ≥ once a week and feeling out of control") were included as cases for BE-BROAD (not BE-NARROW). Controls were individuals with no history of binge eating or any ED.

Acknowledgements: This publication is the work of the authors and Nadia Micali will serve as guarantor for the contents of this paper.

Ethics statement: The authors assert that all procedures contributing to this work comply with the ethical standards of the relevant national and institutional committees on human experimentation and with the Helsinki Declaration of 1975, as revised in 2008. Ethical approval for the study was obtained from the ALSPAC Ethics and Law Committee and the Local Research Ethics Committees. Informed consent for the use of data collected via questionnaires and clinics was obtained from participants following the recommendations

of the ALSPAC Ethics and Law Committee at the time. The main caregiver initially provided consent for child participation, and from the age of 16 years, the offspring themselves have provided informed written consent.

Funding statement: This study represents independent research partly funded by the UK National Institute for Health Research (NIHR) Biomedical Research Centre at South London and Maudsley NHS Foundation Trust and King's College London. High-performance computing facilities were funded with capital equipment grants from the GSTT Charity (TR130505) and Maudsley Charity (980). This work was supported by the UK Medical Research Council and the Medical Research Foundation (MR/R004803/1). The UK Medical Research Council and Wellcome (102215/2/13/2 and 217065/Z/19/Z) and the University of Bristol provide core support for ALSPAC. A comprehensive list of grants is available on the ALSPAC website. This research was specifically funded by the NIHR (CS/01/2008/014) and the NIH (MH087786-01). GWAS data were generated by sample logistics and genotyping facilities at Wellcome Sanger Institute and LabCorp (Laboratory Corporation of America) using support from 23andMe. Mohamed Abdulkadir acknowledges grant support from the National Institute of Mental Health (R01MH120170).

biov

The study was conducted in the Synthetic Derivative (SD) database at the Vanderbilt University Medical Center (VUMC, Nashville, Tenessee, USA). The SD is a de-identified mirror image of the VUMC Electronic Health Records which was established in 1994. DNA was obtained from approximately 275,000 patients who consented to allow leftover clinical blood draws to be banked and linked to de-identified clinical data (BioVU). Further information on BioVU can be found at https://victr.vumc.org/biovu-description/.

AN cases were defined as individuals who have more than one of the following ICD codes: 307.1, F50.0, F50.00, F50.01, F50.02, F50.1 in the electronic health record.

Individuals were defined as having BE-NARROW (and BE-BROAD) if they had more than one of the following ICD codes: 307.51, F50.81, F50.2. Controls consisted of all individuals without any ED ICD codes (i.e., 307.1, 307.5, 307.50, 307.51, 307.52, 307.53, 307.54, 307.59, F50, F50.0, F50.00, F50.01, F50.02, F50.2, F50.8, F50.81, F50.82, F50.89, F50.9, F50.3, F50.1, 307.81).

Ethics statement: The study was approved by the VUMC Institutional Review Board (IRB #201609).

Funding statement: This study was funded by R01MH118233 to Lea K Davis.

esbb

The Estonian Biobank is a large data-rich population-based biobank, covering approximately 20% of the adult population in Estonia (N=~210,000) [23]. All participants have signed an informed consent form and provided blood samples for genotyping. Electronic health records are regularly retrieved by linking to the national health databases and registries, such as the National Health Insurance Funds (NHIF) database, cause of death register, and hospital records.

Individuals with an ICD-10 diagnosis of F50.0 or F50.1 were defined as AN cases. Individuals with a F50.2 diagnosis were included as BE-NARROW cases, and individuals with a F50.2 or F50.3 diagnosis were included as BE-BROAD cases. Controls were defined as individuals without a diagnosis in the ICD-10 F50 chapter.

Ethics statement: The research project has obtained approval from the Estonian Council on Bioethics and Human Research (1.1-12/624).

Funding statement: This research in the Estonian Biobank was supported by the European Union through the European Regional Development Fund (Project No. 2014-

2020.4.01.15-0012), and the Estonian Research Council's grant No. PSG615. Krista Fischer reports support from grant PRG1197 by the Estonian Research Council.

fngn

The FinnGen research project (www.finngen.fi) was launched in 2017 with an aim to improve human health through genetic research [24]. The project combines genome information with digital health care data from national registries: the genotype data are linked to national hospital discharge, death, cancer, and medication reimbursement registries using the national personal identification numbers. The FinnGen study has combined approximately 200,000 existing samples from Finnish biobanks with approximately 300,000 samples from ongoing collections, primarily of hospitalised patients. The final data resource covers roughly 10% of the Finnish population.

Phenotypic information was obtained from Finnish hospital discharge registers. Individuals were defined as AN cases by the following criteria: those with an ICD-10 F50.0, F50.1 code; ICD-9 7830A, 3071A code; and/or ICD-8 784 code. Individuals were defined as BE-NARROW (and BE-BROAD) cases by the following: ICD-10 F50.2, F50.3; and/or ICD-9 3075B. Individuals who did not have any of the diagnoses mentioned in the above case definition were included as controls; individuals with any behavioural syndromes associated with psychological disturbances and physical factors were excluded.

Analysis in this paper was approximately based on the Freeze 7 public release. The final release of FinnGen (~500,000 participants) is now available.

Ethics statement: The study was conducted in accordance with the principles of the Helsinki declaration. Written informed consent was obtained from all the study participants. For the Finnish Institute of Health and Welfare (THL)-driven FinnGen preparatory project and

FinnGen project, all patients and control subjects had provided informed consent for biobank research, based on the Finnish Biobank Act. Alternatively, FINRISK and Health 2000 cohorts were based on study specific consents and later transferred to the THL Biobank after approval by Valvira, the National Supervisory Authority for Welfare and Health. Recruitment protocols followed the biobank protocols approved by Valvira.

Funding statement: The funding agencies had no role in the design and conduct of the study; collection, analysis, and interpretation of data; or the writing of the manuscript or the decision to submit it for publication. The FinnGen project is funded by two grants from Business Finland (HUS 4685/31/2016 and UH 4386/31/2016) and nine industry partners (AbbVie, AstraZeneca, Biogen, Celgene, Genentech, GSK, MSD, Pfizer, and Sanofi). Samuli Ripatti and Jaakko Kaprio report funding from the Academy of Finland Center of Excellence in Complex Disease Genetics (grant # 352792).

gcfm

French GCAN samples (described under "GCAN/WTCCC-3") that were not included in previous AN GWAS efforts were combined with new data from the Eating Disorders Continuum (EDC) at the Douglas Mental Health University Institute in Montreal. The EDC is the only large-scale, supraregional (province-wide) clinical-research-teaching program specialised in eating disorders (EDs) in the Province of Quebec, Canada. Having a supraregional mandate, the EDC serves as a main hub in the effort to care for Quebecers with EDs. The EDC offers inpatient, day program and outpatient services to people living in various regions of the province.

DNA samples provided by the Douglas EDC were sourced from blood obtained from people who participated in various genetic and epigenetic studies conducted in the program between 2001 and 2016. The studies implicated people with anorexia nervosa, bulimia

nervosa, Eating Disorder Not Otherwise Specified, Other Specified Eating or Feeding Disorder, or no lifetime history of eating disorder. Individuals with EDs were recruited mainly from the EDC patient population. Controls were solicited mainly through public announcements. DSM-IV or DSM-5 diagnostic determinations were drawn using data from the Eating Disorder Examination Interview and/or the Eating Disorder Examination Questionnaire. Normal eaters had a body mass index greater than 18, lifetime absence of eating disorder, and no current major psychiatric disorder. Findings involving specific subgroups of the individuals described have been included in several previous publications from the EDC group, addressing gene-environment interactions acting in the EDs, or alterations in DNA methylation linked to the EDs and their comorbid features.

Ethics statement: In all cases, there was consent to participation in one or more of the original studies, as well as to sharing of DNA with other research groups for additional genetic studies. All aspects were approved by the Research Ethics Boards of the Douglas Mental Health University Institute or Montreal West Island Integrated University Health and Social Services Centre.

Funding statement: The original studies were funded by three different grants from the Canadian Institutes of Health Research (MOP-79490, MOP-57929, MOP-142717) or by industry support from Cogir Immobilier ("COGIR"). Funders had no role in study design, data collection, analysis and interpretation, report writing, or in decisions surrounding submission of papers for publication.

jans

Study participants were from the CAPSS220BED (ClinicalTrials.gov ID: NCT00210808) study, which is a multicentre, randomised, double-blind, placebo-controlled, flexible-dose study to assess the safety and efficacy of Topiramate in the treatment of moderate to severe BED associated with obesity.

All cases had a diagnosis of BED as defined by the DSM-IV and supported by the Structured Clinical Interview for DSM-IV Axis I Disorder Patient Edition (SCID-I/P). In addition, they reported binge-eating episodes >3 days per week in the week prior to baseline. Control samples were drawn from NINDS Human Genetics Resource center (neurologically normal White control panel NDPT020, NDPT079, NDPT084, NDPT090, NDPT093, NDPT094, NDPT095, NDPT096, NDPT098, and NDPT099) with known medical and family history managed by the Coriell Institute for Medical Research (Camden, NJ).

Ethics statement: The clinical study was approved by the appropriate ethical review boards and followed the principles outlined in the Declaration of Helsinki for all human investigations. In addition, informed consent has been obtained from the study participants.

Funding statement: The study was funded by Johnson & Johnson Pharmaceutical Research & Development, L.L.C.

moba

The Norwegian Mother, Father and Child Cohort Study (*MoBa*, https://www.fhi.no/en/ch/studies/moba/) is a population-based pregnancy cohort study conducted by the Norwegian Institute of Public Health [25,26]. Participants were recruited from all over Norway from 1999-2008. The women consented to participation in 41% of the pregnancies. The cohort includes approximately 114,500 children, 95,200 mothers and 75,200 fathers. The current study is based on version 12 of the quality-assured data files released for research in January 2019. Blood samples were obtained from both parents during pregnancy and from mothers and children (umbilical cord) at birth [27]. The Medical Birth Registry, a national health registry containing information about all births in Norway from 1967 onwards, was used to identify sex registered at birth for the offspring generation. Full details of genotyping, quality control, phasing, imputation, and post-imputation quality

control have been previously described [28]. After post-imputation quality control, 207,569 individuals with 6,981,748 autosomal, 174,462 chromosome X and 3,200 pseudoautosomal SNPs were available for analysis.

Parental cases were selected based on a combination of questionnaire data (collected from mothers and fathers during pregnancy and when their children were 8 years old) and diagnostic codes from specialist health care (reported in the Norwegian Patient Registry between 2008 and 2020). Offspring cases were based on maternal reports when children were 8 years old and diagnostic codes from specialist health care as above. Individuals were excluded as controls if they were (1) an AN or BE case or (2) were offspring of a parent who was an AN or BE case.

Acknowledgements: The Norwegian Mother, Father and Child Cohort Study is supported by the Norwegian Ministry of Health and Care Services and the Ministry of Education and Research. We are grateful to all the participating families in Norway who take part in this on-going cohort study. Data from the Norwegian Patient Registry has been used in this publication. The interpretation and reporting of these data are the sole responsibility of the authors, and no endorsement by the Norwegian Patient Registry is intended nor should be inferred.

Ethics statement: The establishment of MoBa and initial data collection was based on a licence from the Norwegian Data Protection Agency and approval from The Regional Committees for Medical and Health Research Ethics. The MoBa cohort is currently regulated by the Norwegian Health Registry Act. The current study was approved by The Regional Committees for Medical and Health Research Ethics (20311).

Funding statement: We thank the Norwegian Institute of Public Health (NIPH) for generating high-quality genomic data. This research is part of the HARVEST collaboration, supported by the Research Council of Norway (#229624) and the South-Eastern Norway Regional Health Authority (#2023031). We also thank the NORMENT Centre, in

collaboration with deCODE Genetics, for providing genotype data, funded by the Research Council of Norway (#223273), South-Eastern Norway Health Authorities and Stiftelsen Kristian Gerhard Jebsen. We also acknowledge funding from the U.S. National Institute of Child Health and Development (R01-HD047186, Bulik, PI). We further thank the Center for Diabetes Research, the University of Bergen for providing genotype data and performing quality control and imputation of the data funded by the ERC AdG project SELECTionPREDISPOSED, Stiftelsen Kristian Gerhard Jebsen, Trond Mohn Foundation, the Research Council of Norway, the Novo Nordisk Foundation, the University of Bergen, and the Western Norway Health Authorities. Ole Andreassen was funded by the Research Council of Norway (#324499, #324252), EU's H2020 RIA grant (#964874), NordForsk project (#164218) and KG Jebsen Stiftelsen, Alexandra Havdahl was funded by the South-Eastern Norway Health Authorities (#2019097, #2020022), Helga Ask was funded by the Research Council of Norway (#324620) and NordForsk (#156298). Elizabeth Corfield was supported by the Research Council of Norway (#274611) and South-Eastern Norway Health Authorities fellowship (#2021045).

This work was performed on the Tjeneste for Sensitive Data facilities, owned by the University of Oslo, operated and developed by the Tjeneste for Sensitive Data service group at the University of Oslo, IT Department (USIT). The computations were performed on resources provided by Sigma2 - the National Infrastructure for High-Performance Computing and Data Storage in Norway.

net2

Dutch GCAN samples (described under "GCAN/WTCCC-3") that were not included in previous AN GWAS efforts were combined with new data from the Utrecht Research Group Eating Disorders (URGE, https://urge-eatingdisorders.nl/). URGE is a case-only cohort and patients were recruited from the Eating Disorder clinic Rintveld (Altrecht, Zeist, the Netherlands) and provided informed consent and a blood sample to extract DNA.

ED diagnoses were determined according to the DSM-IV or DSM-5 criteria (whichever was appropriate at the time of data collection) by psychiatrists or (clinical) psychologists with ample experience in the treatment and classification of people with EDs. Each diagnosis was verified using questions from the Eating Disorders Examination.

Ethics statement: Informed consent was obtained from all patients who provided DNA. The Biobank Ethics Committee of the University of Utrecht gave ethical approval for this work.

Funding statement: Roger Adan was supported by grants of Netherlands Organisation for Scientific Research (NWO: 024.004.012, ALWOP.137 and OCENW.KLEIN.071).

ukb2

The UK Biobank represents a large population-based prospective study designed for in-depth exploration of both genetic and non-genetic factors that influence diseases prevalent in middle and old age [29]. By 2010, 500,000 participants were part of the UK Biobank. Cases for the current study were identified via:

1. Mental health questionnaire (MHQ data) [30]: A questionnaire to examine lifetime and current mental health problems.
2. Hospital Episode Statistics (HES data): This category contains summary fields relating to diagnoses made during hospital inpatient admissions. The diagnosis data-fields lists, for each participant, all the distinct values for that data-field in the inpatient data, including main and secondary diagnoses, coded according to the International Classification of Diseases (ICD-9 & ICD-10).
3. Death register (DR data): This category contains coded data on the cause of death (International Classification of Diseases [ICD10]), obtained through linkage to national death registries.

4. Primary care linked data (GP data): UK Biobank has been liaising with various data suppliers and other intermediaries (including the main primary care computer system suppliers in England) to obtain primary care data for UK Biobank participants, all of whom have provided written consent for linkage to their health-related records.

Controls were defined as UK Biobank participants without a reported ED or binge eating (i.e., answered and indicated NOT having an ED or binge eating) or missing for that information.

Ethics statement: UK Biobank is registered as a Research Tissue Bank (North West – Haydock Research Ethics Committee, 21/NW/0157). All data and sample applications may use this ethical clearance to conduct their research. Separate Research Ethics Committee ("REC") or other ethical clearance is not required. This research was conducted under application 82087 (PI: Jonathan Coleman). Participants gave broad informed consent for their data to be used for health-related research purposes.

Funding statement: This study represents independent research partly funded by the UK National Institute for Health Research (NIHR) Biomedical Research Centre at South London and Maudsley NHS Foundation Trust and King's College London. High-performance computing facilities were supported by the King's College London Computational Research, Engineering and Technology Environment [31].

ukch

This dataset consists of a combination of 1) cases recruited via the Charlotte's Helix Project and 2) controls recruited via the National Institute for Health and Care Research BioResource (NBR, https://bioresource.nihr.ac.uk). Charlotte's Helix was supported by the Charlotte's Helix charity, in memory of the activist Charlotte Bevan, and consists of volunteers who had a diagnosis of anorexia nervosa or bulimia nervosa. Cases were ascertained through algorithms based on answers to the ED100K, reported diagnoses from

clinicians, self-reported diagnoses, or registration notes. The genotyping data for cases were generated using Illumina Global Screening Array, while the genotyping data for controls were generated using the Affymetrix Axiom microarray (Supplementary Table 4).

Ethics statement: Charlotte's Helix participants were recruited using ethics approval provided by the Research Ethics Committee South Central Oxford C (15/SC/0388). Participants in Charlotte's Helix provided informed consent for the study. Participants in the NIHR Bioresource consent to long-term de-personalized data storage, donating a biological sample, being contacted for future research projects based on genetic/phenotypic data, receiving additional questionnaires, and linkage of medical records.

Funding statement: This study represents independent research partly funded by the UK National Institute for Health Research (NIHR) Biomedical Research Centre at South London and Maudsley NHS Foundation Trust and King's College London. Ulrike Schmidt receives salary support from the NIHR BRC, and from the Medical Research Council/Arts and Humanities Research Council/Economic and Social Research Council Adolescence, Mental Health and the Developing Mind initiative as part of the EDIFY programme (grant number MR/W002418/1).

ukd2

This dataset consists of the Genetic Links to Anxiety and Depression (GLAD) Study [32], Charlotte's Helix (described under *ukch*), and the COVID19 Psychiatry and Neurological Genetics study (COPING) drawn from participants in the UK National Bioresource (NBR). The GLAD Study is a recontactable data resource of ~40,000 individuals based in the UK with lifetime anxiety and/or depression and forms part of the National Institute for Health and Care Research (NIHR) Mental Health BioResource. Participants are recruited online and as part of this answer multiple questionnaires, including the optional ED100K which provides information about their ED status. Participants have to be over 16

years old and live in the UK to participate in the GLAD Study. The Charlotte's Helix data included in *ukd2* are independent and non-overlapping with regards to *ukch.* These data were separated because they were genotyped on different platforms – the genotyping data of the Charlotte's Helix participants in *ukd2* were generated using the Affymetrix Axiom array.

The NBR is a databank and recontactable resource of volunteers who have provided medical, clinical, and biological data. Volunteers were recruited through a variety of approaches, including National Health Service (NHS) blood transfusion services and various disease/disorder-focused research efforts. Throughout the pandemic, NBR participants were given the opportunity to join the COPING study, which launched in April 2020. The COPING study contained questionnaires from the sign-up surveys of the GLAD study [32] and the Eating Disorders Genetics Initiative (EDGI UK), as well as additional questionnaires to assess COVID-related variables, that is, experiences related to the COVID-19 pandemic. Notably, the ED100K questionnaire was mandatory in COPING. Participants first completed a baseline survey and then follow-up surveys, initially every two weeks but then monthly from August 2020. The last round of invites was sent on 19th January 2021.

COPING study participants come from multiple sub-cohorts of the NBR, including: the GLAD Study (n=14,948); EDGI UK (n=1010); the Inflammatory Bowel Disease BioResource (IBD; n=3203); NHS blood and transplant studies, including INTERVAL (n=4656), COMPARE (n=1928), and STRategies to Improve Donor Experiences (STRIDES; n=2808); and the Research Tissue Bank—Generic (n=4343). We restricted the number of controls from IBD via random selection to reflect the population prevalence of inflammatory bowel disease.

Both for GLAD and NBR, we ascertained AN or BE cases via self-reported diagnoses with (AN, BE-NARROW, and BE-BROAD) and without (AN and BE-BROAD) specification that this has been confirmed by a healthcare professional and/or DSM-5 algorithm-derived

diagnoses (AN, AN-R, and AN-BP) via answers to the ED100K, or via self-reported binge eating plus frequency and duration via answers to the ED100K (BE-BROAD and BE-NARROW). Controls were defined as those without algorithm-derived or self-reported AN or BE and have indicated that they "have never suspected an eating disorder".

Ethics statement: The GLAD Study was approved by the London - Fulham Research Ethics Committee on 21st August 2018 (REC reference: 18/LO/1218) following a full review by the committee. The NIHR BioResource has been approved as a Research Tissue Bank by the East of England - Cambridge Central Committee (REC reference: 17/EE/0025). Prior to submission for ethical approval, this research was reviewed by a team with experience of mental health problems and their carers who have been specially trained to advise on research proposals and documentation through the Feasibility and Acceptability Support Team for Researchers (FAST-R): a free, confidential service in England provided by the NIHR Maudsley Biomedical Research Centre via King's College London and South London and Maudsley NHS Foundation Trust. Participants in the NIHR Bioresource consent to long-term de-personalized data storage, donating a biological sample, being contacted for future research projects based on genetic/phenotypic data, receiving additional questionnaires, and linkage of medical records.

Funding statement: This work was supported by the National Institute of Health Research (NIHR) BioResource, NIHR Biomedical Research Centre [IS-BRC-1215-20018], HSC R&D Division, Public Health Agency [COM/5516/18], MRC Mental Health Data Pathfinder Award (MC_PC_17,217), and the National Centre for Mental Health funding through Health and Care Research Wales.

jpan

Some DNA samples from Japanese participants collected as part of the GCAN effort (referenced under "GCAN/WTCCC-3") were not included in previous AN GWAS efforts.

These samples were newly genotyped for inclusion in this paper. As described in a previous publication [2], cases met DSM-IV criteria for AN, with AN-free controls recruited in parallel.

Ethics statement: Please refer to “GCAN/WTCCC-3”.

Funding statement: The data and sample collection were supported by Grants-in-Aid for Scientific Research (20390201 and 23390201) awarded to G. Komaki by the Ministry of Education, Culture, Sports, Science, and Technology of Japan.

kran

Study participants were from HD16A1353 (cris.nih.go.kr ID: KCT 00210808) study, which was a multicentre study to investigate eating behaviours and the physical and psychological status of women with abnormal weight or patients with EDs.

Cases had a diagnosis of anorexia nervosa (restricting type or binge-purging type) as defined by the DSM-IV and supported by the Structured Clinical Interview for DSM-IV Axis I Disorder Patient Edition (SCID-I/P). Controls were university students recruited for the study and confirmed to have no ED after screening.

Ethics statement: The study was conducted according to the guidelines of the Declaration of Helsinki and approved by the Institutional Review Board of Inje University (INJE 2016-01-003-002). All participants provided informed consent.

Funding statement: This work was supported by the Korea Centers for Disease Control and Prevention Research Fund [grant number: 2016-ER6310-00, 2016].

Supplementary Methods

Genotype quality control

Information about genotyping platforms for each cohort, and availability of chromosome X data, is documented in Supplementary Table 4. For cohorts included in our previous publication [1], quality-controlled data were used for analyses of anorexia nervosa (AN). For analyses of AN subtypes and for the broad and narrow binge-eating phenotypes (BE-BROAD and BE-NARROW), additional exclusions of single-nucleotide polymorphisms (SNPs) and individuals were made where necessary to align these data with the quality control of newly included data, as described below. Summary-level data were available from contributing cohorts (Supplementary Table 1). Quality control of these datasets followed a basic shared protocol, with deviations as necessary for each cohort as described below.

In general, newly included data underwent the following process of quality control. Duplicate SNPs were removed, excluding the variant with the lower call rate. We also removed monomorphic variants, and variants with a call rate<95%. We then included samples with a call rate of ≥98%, and then applied a second SNP filter, excluding variants with a call rate of ≤98%, variants with >2% difference in call rates between cases and controls, and variants with significant Hardy-Weinberg Equilibrium (HWE) deviations of $P<10^{-10}$ (cases) and $P<10^{-6}$ (controls).

For the following quality control steps, we performed pruning of the variants based on linkage disequilibrium (LD) to only retain SNPs in relative linkage equilibrium with each other (PLINK --indep-pairwise 1500 150 0.2; window size was 1500 SNPs, 150 step size, excludes SNP pairs with $r^2>0.2$). We also excluded high LD-regions (4 Mb inversion on chromosome 8 and an 8 Mb region covering the extended MHC region on chromosome 6) and only retained chromosomes 1-22. We compared sex assignments in the dataset with those imputed from chromosome X inbreeding coefficients (F^{het}), excluding samples with a sex mismatch. We also excluded individuals with a genome-wide F^{het} deviation of >0.2. We calculated the

proportion of identity-by-descent (IBD) or pi-hat for each pair within a cohort. The cohorts that used mixed models included relatives, but for the cohorts that did not use mixed models, we removed one member of each related pair (pi-hat>0.1875).

We then calculated principal components (PCs) on the set of pruned SNPs using reference data from the 1000 Genomes Project [33], which we limited to variants with genotyping call rate>99%, minor allele frequency (MAF)>0.01, and HWE deviations of $P>10^{-3}$. For each cohort, we then plotted the PCs to visualise the largest homogenous cluster. This clustering indicates approximately shared genetic ancestry. Individuals outside of this largest cluster were typically in the minority and were excluded from further analysis. For studies included in the main analysis, the participants in the largest cluster self-identified as from European ethnic groups and were labelled as being of European genetic ancestry. For the Korean and Japanese studies used in the follow-up analyses, the participants in the largest cluster self-identified as from East Asian ethnic groups and so were labelled as being of East Asian genetic ancestry.

We then repeated the entire quality control process and calculated PCs for each cohort separately–without reference to the 1kGP–to use at a later stage in the association analyses (Supplementary Table 4). We restricted chromosome X analyses to individuals included in the autosomal analysis and conducted quality control as for autosomal SNPs.

Imputation

For all individual-level data cohorts where patient consent and national data privacy laws allowed, autosomal and chromosome X genotypes were imputed to TOPMed freeze 8 [34] using Minimac4, and phasing was performed with Eagle 2.4 [35] via the TOPMed Imputation Server [36]. For individual-level data cohorts that could not be imputed to TOPMed, we imputed to the Haplotype Reference Consortium (HRC) [37] using Minimac3, with phasing conducted via Eagle v2.3.5, implemented in the Ricopili pipeline [38]. Cohorts supplying summary statistics used TOPMed, HRC, or in some cases used bespoke imputation

reference panels (Supplementary Table 4). We plotted imputation INFO scores against various MAF bins to check imputation quality, and we assessed missingness for each MAF bin.

Association analyses and post-analysis processing and quality control

For studies using whole genome regression in REGENIE step 1, we used a set of pruned SNPs with MAF>0.01, excluding high LD regions and only including autosomal chromosomes (PLINK command: --indep-pairwise 1500 150 0.2).

We aligned the summary statistics of each GWAS to the TOPMed reference panel in Genome Reference Consortium Build 37 (GRCh37/hg19), using variant positions from ENSEMBL (https://ftp.ensembl.org/pub/grch37/release-113/data_files/homo_sapiens/GRCh37/variation_genotype/TOPMED_GRCh37.vcf.gz). For studies that were in GRCh38, we first linked the datasets with the GRCh38 TOPMed reference panel (https://ftp.ensembl.org/pub/grch37/release-113/data_files/homo_sapiens/GRCh38/variation_genotype/TOPMED_GRCh38.vcf.gz) by chromosome/base pair, then selected the SNP rsID labels and linked these labels to the GRCh37 TOPMed reference panel, and finally extracted the GRCh37 chromosome/base pair information for each SNP. Variants without a rsID label in the GRCh38 TOPMed reference panel were lifted over to GRCh37 using the liftOver tool (https://ftp.ensembl.org/pub/grch37/release-113/data_files/homo_sapiens/GRCh38/variation_genotype/TOPMED_GRCh38.vcf.gz). After alignment, we applied an INFO and MAF filter to include SNPs with INFO>0.3 and MAF>0.01 in cases and controls. We then used DENTIST [39] to remove variants with effects inconsistent with their linkage disequilibrium pattern with other assessed variants, estimating linkage disequilibrium from European ancestry individuals in Phase 3 of the 1000 Genomes project.

Cohort by cohort deviations from established quality control and GWAS protocol

The following cohorts are fully described by the established quality control and GWAS protocol: *chop, fin1, fre1, itgr, sedk, gcfm, jans, net2, sebe, biov, alsp, moba*. Other cohorts, and their important deviations from these protocols, are listed below. For cohorts included in our previous publication (*gns2*, *net1*, *poco*, *spa1*, *ukd1*, *usa1*, *aunz*, *usa2*), these deviations were carried over from that publication [1].

gns2

Variants failing the Hardy-Weinberg test at $P<10^{-6}$ in AN cases were excluded.

net1

Variants failing the Hardy-Weinberg test at $P<10^{-6}$ in AN cases were excluded, as were variants with a call rate difference>1% between AN cases and controls. The minor allele frequency of rs4492741 differed notably from reference data and this variant was removed.

poco

Variants with a call rate difference>1% between AN cases and controls were excluded, as were variants with a call rate<99% in AN cases, and variants with a minor allele frequency<1% in controls.

spa1

Variants with a call rate<99% in AN cases were excluded, as were variants failing the Hardy-Weinberg test at $P<1.26\times10^{-6}$ in AN cases.

ukd1

Variants with a call rate difference>1% between AN cases and controls were excluded, as were variants with a call rate<99% in AN cases, variants with a minor allele frequency<1% in controls, and variants failing the Hardy-Weinberg test at $P<10^{-6}$ in AN cases.

usa1

Variants with a call rate<99% in AN cases and controls combined were excluded, as were variants failing the Hardy-Weinberg test at $P<10^{-6}$ in AN cases.

aunz

Quality control for this cohort was conducted at the Queensland Institute of Medical Research. Prior to principal component analysis, variants were excluded for missing strand or position information, if they were duplicates of other genotyped variants, or if they showed inadequate cluster separation during genotyping (Illumina GenCall GenTran score<0.6). Variants were then excluded if they had an overall call rate<95% across AN cases and controls combined, or if they failed the Hardy-Weinberg test at $P<10^{-6}$ in AN cases and controls combined. Participants were excluded if they were duplicates of existing individuals. Following principal component analysis, quality control proceeded as per protocol, except that variants were excluded if they had a call rate<99% across AN cases, and that one of each pair of related participants were excluded (pi-hat>0.2).

ipsy

iPSYCH analyses used the same participants included in our previous analyses [1], with additional control filtering criteria. Specifically, controls were filtered for evidence of anorexia nervosa, bulimia nervosa, or eating disorders not otherwise specified. Quality

control and imputation was performed on the entire iPSYCH cohort (N≈86,000), including psychiatric diagnoses beyond eating disorders [40]. Unlike our previous analyses [1], in which 24 separate GWAS analyses were conducted within genotyping batches, all genotyping batches were included in a single mega-analysis with a covariate for genotyping batch.

usa2

Variants with a call rate<99% in AN cases were excluded, as were variants with minor allele frequency<1% in AN cases and in controls (considered separately), and variants failing the Hardy-Weinberg test at $P<2.24\times10^{-3}$ in controls. In addition, the minor allele frequency of rs183114681 differed notably from reference data, and the association of rs6917603 did not propagate to variants in linkage disequilibrium – both of these variants were removed.

agds

Imputed variants were limited to an INFO threshold of 0.6.

esbb

Imputation in the Estonian Biobank was conducted to a bespoke panel comprising whole genome sequence data from 2244 Estonians [41].

fngn

Data from FinnGen underwent central quality control and imputation as described here [24]. Data release R7 was used for these analyses. In brief, quality control was similar to our established protocol, but some parameter settings differed, such as higher cut-offs for individual call rate (99.5%), heterozygosity outliers being determined based on standard deviations (+/- 4SD), and allele frequencies being compared to the bespoke imputation

panel. Imputation was conducted to a bespoke panel constructed from high-coverage whole genome sequencing data from 3775 Finns (SISu v3).

ukb2

The imputed data were restricted to variants with $R^2 \geq 0.4$, MAF≥0.01. GWAS analysis was conducted using REGENIE's mixed model with 10 PCs, genotyping batch, and UK Biobank recruitment centre as covariates.

ukch

Charlotte's Helix (Illumina) and NBR (Affymetrix) datasets were imputed separately, and then merged [42]. Each set of imputed data was limited to variants with $R^2 \geq 0.3$, MAF≥0.01. Following merging, we excluded variants with differences in MAF and R^2 greater than 0.05 between the two datasets. GWAS analysis followed the established protocol.

ukd2

GLAD, Charlotte's Helix, and the NBR dataset underwent quality control and imputation separately and were restricted to variants with $R^2 \geq 0.3$, MAF≥0.001. Imputed data were merged, and we excluded variants with MAF<0.01, with differences in MAF and $R^2 > 0.05$ between any pairing of the three datasets, and/or with significant Hardy-Weinberg Equilibrium deviations of $P < 10^{-10}$ (cases) and $P < 10^{-6}$ (controls). GWAS analysis was conducted using REGENIE's mixed model with 10 principal components and array version as covariates.

Power analysis

We estimated what magnitude of relative risk we were powered to detect using Genetic Power Calculator [43]. Assuming perfect linkage disequilibrium between the marker and the risk SNP (D'=1), we set the number of cases to ½ N_{eff} (Supplementary Table 1) and

the control:case ratio to 1. We specified lifetime risk as described for SNP-based heritability in the main text. We assumed an additive model, set the p-value significance threshold to 5×10^{-8}, and assessed MAF ranging from 0.01 to 0.5. For AN, we also conducted analyses based on the previous GWAS of Watson et al for comparison [1], calculating number of cases as the sum of ½ Neff across cohorts, which totalled 23,161 (compared to 36,753 in our analyses).

Polygenic prediction

<u>*Cohort selection*</u>

We selected the target cohorts based on effective sample size (N_{eff} half>1000). The predictive performance of BE-BROAD, BE-NARROW and AN PRS on each available outcome were tested in these cohorts. The target cohorts for BE-BROAD, BE-NARROW, and AN are as follows:

- BE-BROAD:
  - *aunz, sedk, ukb2, ukd2* (data available to PGC-ED analysts);
  - *alsp, moba, esbb* (data from cohorts that contributed summary statistics).
- BE-NARROW:
  - *aunz, sedk, ukb2, ukd2* (data available to PGC-ED analysts);
  - *esbb, fngn,* and *moba* (data from cohorts that contributed summary statistics).
- AN:
  - *gns2, chop, aunz, sedk, ukb2, ukd2* (data available to PGC-ED analysts);
  - *usa2, esbb, fngn, moba* and *ipsy* (data from cohorts that contributed summary statistics).

We also sought to explore the difference of BE-BROAD and AN PRS in three subgroups of individuals with eating disorders compared to controls (i.e., individuals without

BE-BROAD and AN): (a) those with BE-BROAD only; (b) individuals with both BE-BROAD and AN (BE+AN), and (c) those with AN only.

We selected cohorts with sufficient sample size for ≥2 subgroups to assess differences between subgroups. Specifically, we selected *aunz* and *sedk* to assess how the PRS levels differ from controls in BE+AN individuals and in those with AN only. In these cohorts, we assessed binge-eating behaviour (and thus fulfilling criteria for BE-BROAD) in individuals with AN. Consequently, the AN-only group reflects a mix of individuals with both AN-R and AN-BP as some individuals with AN-BP may never have had a binge-eating episode but are classified for AN-BP due to the presence of purging behaviour. BE+AN individuals will always have a diagnosis of AN-BP. Both *aunz* and *sedk* comprise individuals recruited through the Anorexia Nervosa Genetics Initiative (ANGI) and thus consist only of individuals with AN and do not have any individuals with BE-BROAD-only.

We also performed subgroup analyses in cohorts not specifically recruited for AN. We selected *ukb2* and *ukd2* as additional target cohorts to assess differences between all subgroups. In *ukb2,* no symptom or AN subtype-level data were available, so individuals with lifetime diagnoses of both AN and BN/BED are considered to be in the BE+AN group. *ukd2* mostly comprises data on eating disorders in the context of depression. Therefore, it may not be representative of the general clinical population of individuals with AN and binge-eating behaviour.

Sex-specific analyses

<u>*Cohort selection*</u>

As the participants from most cohorts are predominantly female, the combined sample sizes were not sufficient for a male-only GWAS meta-analysis. We therefore opted to perform female-only meta-analyses for BE-BROAD and AN and use this as base data for generating a PRS. We tested this PRS in males in selected cohorts to examine whether BE-

BROAD and AN PRS built in females could be applied to predict BE-BROAD and AN risk in males.

Both for the female-only GWAS and the male-only target, we included only cohorts where sex-specific case and control n were both >100. For female-only BE-BROAD GWAS (37,580 cases, 691,808 controls), we included all cohorts except *fin1, fre1, gcfm, gns2, itgr, net1, poco, spa1, ukd1,* and *net2*. For female-only AN GWAS (21,898 cases, 694,722 controls), we included all cohorts except *itgr, spa1, ukd1, jans,* and *net2.* For the male-only BE-BROAD target (1055 cases, 38,046 controls), we included *alsp, ukd2,* and *moba* (we opted to include *moba* despite N_{case}=98 after excluding relatives). For the male-only AN (1524 cases, 388,891 controls) target, we included *fngn, ipsy,* and *ukb2*.

Causal mixture modelling

To validate estimates of polygenicity and discoverability from MiXeR, we additionally conducted analyses using SBayesRC [44]. SBayesRC is a method for enhancing polygenic risk scoring through identifying likely causal variants from GWAS summary statistics, leveraging functional annotations. As part of this, it outputs estimates of polygenicity, as well as grouping the proposed causal variants into sets of zero, very small (per SNP heritability=0.001%), small (0.01%), medium (0.1%), and large (1%) effect. In this instance, we were interested only in these, rather than the broader functions of SBayesRC. SBayesRC and MiXeR are distinct methods, reliant on different assumptions about genetic architecture, and as such results are not expected to align perfectly but should be broadly consistent when applied to the same data. We conducted analyses on our BE-BROAD and AN summary statistics, restricted to variants with MAF≥0.01 and INFO≥0.6, using per-variant Neff (calculated by Ricopili as Neff_half, and then doubled) in place of N, and following the protocols provided on the SBayesRC GitHub (https://github.com/zhilizheng/SBayesRC).

Mendelian randomisation

Three main assumptions are made for Mendelian randomisation (MR): (1) the genetic instrument must have a strong association with the exposure, (2) the genetic instrument is independent of confounding factors in the relationship between the exposure and the outcome, and (3) the outcome is associated with the genetic instrument only through the effect of the exposure [45,46].

Of these three assumptions, only the first can be tested directly by calculating the F-statistic [46]. An $F<10$ suggests potential weak instrument bias [47]. In addition, for BMI as exposure independent genome-wide significant SNPs (the threshold of $P<5x10^{-9}$ was used in GWAS on BMI due to denser imputation data) are used as instrument variables (IV). To create IV for BE and AN, we used a threshold of $P<5x10^{-6}$ to include more SNPs. Heterogeneity of IV and pleiotropy lead to a violation of the second and third assumption and to biased effect estimates. We used Cochran's Q-statistic to test for IV heterogeneity. Cochran's Q-statistic assesses whether causal estimates of SNPs are comparable, with a significant finding ($P<0.05$) indicating heterogeneity [48]. Heterogeneity may have several causes, of which horizontal pleiotropy is the most likely [49]. To specifically investigate horizontal pleiotropy, we calculated MR–Egger regression and performed an MR-PRESSO analysis. If Egger's intercept is not significantly different from zero, horizontal pleiotropy is unlikely [50]. MR PRESSO uses a global bias test to evaluate whether the removal of potentially pleiotropic instruments results in a significant difference in the overall causal estimate and provides a corrected causal estimate after the removal of pleiotropic instruments [51]. Simulation studies have shown that MR PRESSO and Cochran’s Q-statistic are substantially more sensitive and powerful to horizontal (global) pleiotropy than Egger's intercept [49,51]. We excluded from the analysis SNPs identified by MR PRESSO as pleiotropic.

Since it is unlikely that all IVs fulfil the assumptions for instrumental variables, and since it is not possible to test the fulfilment of these assumptions, many different MR methods have been developed. These methods differ in their robustness to various violations of assumptions. Because no method alone can provide infallible proof of causality, the use of different methods was recommended to assess whether a causal effect detected by MR is robust [52,53]. Accordingly, we applied the following MR methods. The inverse-variance-weighted (IVW) method assumes that all ratio estimates provide independent evidence of the causal effect and that all genetic variants are valid instruments [50,54]. MR–Egger considers an intercept term interpreted as the average pleiotropic effect of the genetic variants included in the analyses. If the pleiotropic effects are distributed independently of the genetic associations with the risk factor (InSIDE assumption: INstrument Strength Independent of Direct Effect), then the MR–Egger estimate is a consistent estimate of the causal effect as both the sample size and the number of genetic variants increase [50,55]. Mode-based estimation (MBE; simple mode, weighted mode) consistently estimates the true causal effect under the assumption that across all instruments, the most frequent value of bias due to pleiotropy is zero (ZEro-modal pleiotropy assumption (ZEMPA)) [55]. Simple MBE is less accurate than weighted MBE, but simple MBE is less prone to bias due to violations of the InSIDE assumption. Median-based estimators are consistent even when up to 50% of the instruments are invalid. The weighted median estimator has similar efficiency to the IVW method, but the simple median estimator is less efficient than either the IVW or the weighted median method [56]. The penalised weighted median estimator is robust in the case of IV heterogeneity [57].

In the case of IV heterogeneity as indicated by Cochran's Q statistic, two methods robust to heterogeneity were used in addition to the penalised weighted median method [57]. The contamination mixture method has a good overall performance (the lowest mean squared error) with up to 40% invalid instruments compared to other robust methods. The contamination mixture method identifies distinct subgroups of genetic variants with mutually

similar causal estimates [58]. MR-Lasso extends the IVW model to include an intercept term for each genetic variant. These intercept terms represent associations between genetic variants and the outcome that bypasses the risk factor. The causal effect is estimated by weighted linear regression, with the intercept terms subjected to lasso penalisation. Lasso penalisation shrinks the intercept of the valid instruments to zero [57].

Forest and scatter plots were used to visualise the combined results of single and multi-SNP analyses (Supplementary Figures 27 and 28). The scatter plots show single SNP effects on the exposure against single SNP effects on the outcome with corresponding standard deviations and estimated regression lines of the multi-SNP analyses.

In case of unavailability of SNPs in GWAS for the outcome phenotype, we used proxy-SNPs [59]. The SNPs with linkage disequilibrium (LD) of at least $r^2 \geq 0.8$ (on the basis of GRCh37, Ensembl version 87, 1000 genomes: phase 3 version 5 for European ancestry) were extracted from the *in silico* tool SNIPA (http://www.snipa.org. Accessed in November 2024) [60]. Selection criteria for proxy-SNPs were defined: 1st highest r2, 2nd smallest distance to the lead SNP [59].

Analyses were performed using R 4.3.2, and packages *TwoSampleMR* (0.6.8; https://github.com/MRCIEU/TwoSampleMR) [61], *Mendelian Randomization* (0.10.0; https://CRAN.R-project.org/package=MendelianRandomization), and *MR-PRESSO* (1.0; https://github.com/rondolab/MR-PRESSO) [51]. Figures were created with the R package *forestplot* 3.1.5.

Variant heterogeneity in AN

Despite an increase in N of 64%, variant discovery in our AN GWAS did not increase markedly compared to the previous AN GWAS [1]. We hypothesise that this is due to the ascertainment strategy of new cohorts, resulting in a more heterogeneous signal in the new analyses. To determine whether this hypothesis is supported by our results, we examined I^2

heterogeneity statistics in three sets of summary statistics: (1) our main AN GWAS, (2) the AN GWAS from Watson et al [1], and (3) a repeat of our AN GWAS restricted to only the new AN cohorts (i.e. gcfm, sebe, agds, alsp, biov, esbb, fngn, moba, net2, ukb2, ukch, ukd2). We included updated analyses from the UK Biobank in our new GWAS, so UK Biobank is present in both the Watson GWAS and in the new AN cohorts. All sets of summary statistics were derived from the Ricopili pipeline and had heterogeneity statistics calculated in METAL (Main Methods). For each set of summary statistics, we calculated the mean and standard deviation of I^2 across all variants, and binned I^2 across all variants into bins of ≤25%, 25-50%, 50-75%, and >75%.

We then conducted a series of sensitivity analyses. We restricted variants to those shared across the different summary statistics (1+2, and 1+2+3). We also restricted variants to those in linkage equilibrium, in two ways. First, we clumped all variants from the Watson et al GWAS summary statistics without filtering on *P* using PLINK2 [62], with the European ancestry individuals from 1000 Genomes as an LD reference [33]. We removed variants in linkage disequilibrium (r^2>0.9) with variants with a lower p-value within 1Mb. Second, we pruned the set of variants shared across all three sets of summary statistics using PLINK and the European ancestry individuals from 1000 Genomes as an LD reference, removing variants in linkage disequilibrium (r^2>0.9) with a window of 1Mb. We then repeated the I^2 calculations above, and additionally compared the I^2 values for each shared variant across the summary statistics using linear regression and cross-tabulating I^2 bins. Finally, we repeated analyses of shared variants limiting only to variants with $P<5x10^{-8}$ and $P<0.05$ in Watson et al [1].

Gene-wise and gene set analysis, including drug target and drug class analyses

For MAGMA gene-wise analysis, we obtained gene-wise p-values with the multi snp-wise model, which combines the lowest and mean p-values of all SNPs mapped to the gene. For gene-set analyses, we applied a competitive analysis, which regresses the phenotype

on the mean effect of genes within the gene set, with the mean effect of genes outside the gene set as a covariate, in a one-tailed test that the mean effect of genes within the gene set is greater than 0. For drug-class analysis,we first ranked all drug-gene sets according to their association in the drug-set analysis. We then generated enrichment curves for specific drug classes, assigning a 'hit' if the drug-gene set belonged to the class or a "miss" if it was outside the class. We calculated the area under the curve and determined statistical significance with the Wilcoxon Mann-Whitney test, comparing drug-gene sets within the class to those outside the class as a one-tailed test of whether in-class drugs showed greater association with the disorder than out-of-class drugs.

Tissue and cell-type specific analyses

We performed tissue and cell-type heritability (h^2_{SNP}) enrichment analyses to identify tissues and cell-types related to the common genetic risk of BE-BROAD and AN. We analysed h^2_{SNP} enrichment in 27 tissues from GTEx gene expression data (v8), excluding tissues with <100 donors, non-natural tissues (such as cell lines), and testis tissues (an expression outlier) [63]. We further analysed 31 superclusters and 461 cell clusters based on single-nucleus RNA sequencing data from >3,000,000 nuclei from 100 dissections of adult human brain [64]. We defined gene expression specificity as RNA counts per million per gene within each tissue or cell type (assessing superclusters and clusters separately) divided by the total expression of this gene across all tissues or cell types [65]. We compared the per-SNP heritability of SNPs 100kb upstream and downstream of genes with the top 10% specificity in each tissue or cell type against the per-SNP heritability of other SNPs using stratified LDSC, adjusting for 53 baseline annotations [65,66]. We calculated one-sided p-values from coefficient *z*-scores, and accounted for multiple testing using FDR per trait for the tissues, superclusters, and clusters separately.

Supplementary Results

1. Comparison of the two binge-eating behaviour phenotypes

The two BE phenotype definitions balanced phenotypic certainty with sample size—BE-BROAD had a larger sample size and was potentially a better-powered GWAS than BE-NARROW but was likely to be more heterogeneous and could lack specificity to BE. To determine which phenotype to carry forward to follow-up analyses, we considered the LDSC intercept and attenuation ratio statistics and calculated the genetic correlation (SNP-r_g) between the two BE phenotypes. Inflation was a more sizable component of the signal in BE-NARROW (attenuation ratio 0.21 ± 0.06) than in BE-BROAD (0.14 ± 0.04). Furthermore, the SNP-r_g between BE-NARROW and BE-BROAD did not differ from unity (1.00 ± 0.03). We therefore concluded that BE-BROAD appropriately captured the common genetic component of BE with greater statistical power than BE-NARROW.

2. Association meta-analyses

LDSC attenuation ratio estimates were significantly >0 across all phenotypes (0.070 [AN] - 0.59 [AN-R]). A larger attenuation ratio can indicate that the observed signal results from genome-wide inflation rather than true polygenic signal. Ratios were larger in the comparatively underpowered BE-NARROW and the AN subtypes, but smaller in BE-BROAD and AN.

BE-NARROW

For BE-NARROW, we analysed 15 datasets comprising 15,175 cases and 1,217,725 controls and identified five genomic loci (Supplementary Table 5, Supplementary Figure 15).

AN subtypes

For the AN subtypes, we estimated a h^2_{SNP} of 9% (SE=3%) for AN-R and 15% (SE=2%) for AN-BP (Supplementary Table 3). We identified one genomic locus for AN-R (13 datasets, 2524 cases, 33,374 controls) on chromosome 2 which was not reported in the previous AN GWAS [1] (Supplementary Table 5, Supplementary Figure 15). We did not identify any genome-wide significant loci for AN-BP (14 datasets, 5209 cases, 33,951 controls; Supplementary Figure 15).

3. Comparison of BE-BROAD with Burstein et al

A previous publication reported a GWAS of a model-derived phenotype aimed at capturing binge eating disorder in the Million Veteran Program [71]. As this study primarily reported an algorithmic phenotype distinct from our BE definitions, and additionally controlled for BMI, we did not include it within our BE cohorts. Our BE-BROAD definition captures binge eating across multiple eating disorders (and in the absence of a diagnosed disorder), whereas the phenotype from Burstein et al sought to approximate a specific eating disorder (binge eating disorder), which has characteristics beyond the symptom of binge eating alone.

Within individuals from European ancestries, Burstein et al implicated two loci at genome-wide significance in BE. Both loci were on chromosome 6, and the authors mapped these to the *HFE* and *MCHR2* genes respectively. The lead variants rs79220007 and rs17789218 had P=0.018 and 0.0025 respectively in BE-BROAD with a consistent magnitude and direction of effect. Results were similar in the *non-BMI* BE-BROAD analysis (P=0.0089 and 0.0077 respectively). As such, we replicated these findings controlling for two tests, but lacked power to replicate them at a genome-wide level of significance. Notably, rs17789218 is significantly associated with BMI [72] (and also passes genome-wide significance in the GWAS of the shared component between BMI and BE-BROAD) and so this finding may be at risk of collider bias [73].

Burstein et al additionally report genetic correlations of their model-derived phenotype with external traits. Burstein et al report modest positive correlations of BE with psychiatric disorders, ranging from 0.21 with AN to 0.52 with depression. The equivalent correlations with BE-BROAD are 0.43 for AN (previous freeze) and 0.39 for depression (Supplementary Table 9), suggesting the pattern of correlations with psychiatric disorders is similar, but correlations with specific disorders differ. Results for *non-BMI* BE-BROAD are similar to those for BE-BROAD (Supplementary Table 14). Burstein et al also report small negative genetic correlations with cognitive phenotypes (-0.11 to -0.13), which we do not observe with BE-BROAD; a modest negative genetic correlation with subjective well-being (-0.21, we observe -0.41 with BE-BROAD), and modest positive genetic correlations with risk taking (0.30) and lifetime smoking (0.19), which mirror our results from BE-BROAD (0.21 and 0.30 respectively). These results are not materially different when considering *non-BMI* BE-BROAD. Overall, we see both agreement and disagreement between genetic correlations with BE-BROAD and those reported by Burstein et al.

Burstein et al report several significant gene-sets associated with BE – we did not find any significant gene-sets associated with BE-BROAD. It is difficult to make strong conclusions about this difference, given methodological differences in the gene-set analyses performed in the two studies.

4. Female-only analyses

<u>*BE-BROAD*</u>

The female-only GWAS in BE-BROAD was largely consistent with the full GWAS (Supplementary Table 6). The three top loci remained genome-wide significant, along with the locus on chromosome 3. The two loci on chromosomes 11 and 12 did not pass genome-wide significance in female-only analyses, and two loci on chromosomes 1 and 11 passed genome-wide significance in the female-only analyses but not the full analyses. However, the effect estimates of the top SNPs across all loci (full GWAS and female-only) did not

significantly differ between GWAS (z-test $P>0.05$), indicating differences in results were an artefact of sampling error, consistent with the smaller size of the female-only analyses.

AN

For AN, the three top loci from the main GWAS remained genome-wide significant in female-only GWAS. However, remaining loci were more variable—the chromosome 10 locus remained genome-wide significant, but the remaining four loci (on chromosomes 1, 5, 10, and 12) did not pass significance in the female-only analyses. Six additional loci passed genome-wide significance in the female-only analyses but not the full analyses, including the chromosome 2 locus previously observed in Watson et al [1] and loci on chromosomes 3, 4, 6, 20 and 22. However, the effect estimates of the top SNPs across all loci (full GWAS and female-only) did not significantly differ between GWAS (z-test $P>0.05$).

5. Causal mixture modelling

Univariate MiXeR analysis of BE-BROAD and of AN fit well, with positive AIC values compared to the LDSC model, suggesting MiXeR modelling is justified (Supplementary Table 8). Univariate analysis for BE-BROAD resulted in comparable discoverability estimates to psychiatric disorders like ADHD and ASD (2.73×10^{-5}), but much lower polygenicity than is typically observed in psychiatric disorders (3195 variants explaining 90% of heritability) [67]. Estimates of BE-BROAD heritability on the observed scale were comparable to those from LDSC (h^2=6%, SE=0.1%). In comparison, univariate MiXeR analyses for AN resulted in a higher discoverability (3.53×10^{-5}) and polygenicity (8926 variants), similar to that previously reported for bipolar disorder [67]. Again, estimates of AN heritability on the observed scale were comparable those from LDSC (h^2=20%, SE=0.4%).

Bivariate MiXeR analyses should be viewed with caution in this instance. MiXeR compares the best overlap model (fit by optimised weighted log likelihood) with models for the extremes of plausible overlap. First, it compares to a maximal model, where all causal

variants estimated for the first trait overlap with those estimated for the second trait (where the first trait has the fewer causal variants estimated). Second, it compares to the minimal model, which is the least overlap of causal variants possible given the observed genetic correlation. In our analyses, the low polygenicity of BE-BROAD restricts the modelling space for the MiXeR model. If one assumes that all causal variants that are shared are concordant in their direction of effect, then >75% of the putative causal variants for BE-BROAD have to be shared with AN. As a result, although the MiXeR best overlap model fit better than the maximal model, it did not fit better than the minimal model (Supplementary Table 8). This caveat aside, the results from the bivariate analysis are consistent with other results in this study, showing a sizable overlap of causal variants between BE-BROAD and AN with highly concordant directions of effect, as well as non-trivial non-overlapping causal components for both traits, consistent with the differing genetic correlations we observed.

Estimates of observed-scale heritability from SBayesRC were slightly higher than from LDSC for BE-BROAD (h^2=8%, SE=0.1%) and comparable for AN (h^2=21%, SE=0.1%). However, unlike univariate MiXeR, polygenicity estimates were similar between BE-BROAD (53,739 non-zero effect variants) and AN (52,054), with very small and small effect components predominant in both traits (Supplementary Table 8). MiXeR and SBayesRC rely on different assumptions and underlying models, and so inconsistent results can occur, but nonetheless, we caution against over-interpreting our MiXeR results given this lack of validation.

6. BE-BROAD in cohorts not ascertained for anorexia nervosa (BE-BROAD$_{NAAN}$)

Genetic signal in our BE-BROAD GWAS may partly be influenced by AN, given that 18% of our BE-BROAD cases have (known) AN (Supplementary Table 11). As a sensitivity analysis, we conducted an additional BE-BROAD GWAS, excluding cohorts that specifically focused on AN recruitment. We will refer to this phenotype as “not-ascertained-for-AN BE-BROAD” (BE-BROAD$_{NAAN}$). We calculated SNP-r_g between BE-BROAD$_{NAAN}$ and a selection

of other traits based on their associations with BE-BROAD and AN, and tested for significant differences from the respective SNP-r_g with BE-BROAD (Supplementary Figure 17; Supplementary Table 12). BE-BROAD$_{NAAN}$ had significantly stronger genetic associations with the anthropometric traits: body fat percentage was more strongly associated with BE-BROAD$_{NAAN}$ than with BE-BROAD, as was BMI, suggesting that AN cases in the excluded cohorts may be masking this genetic signal. Further, whilst non-significant, the SNP-r_g between BE-BROAD and OCD changed significantly after removing the AN-focused cohorts, remaining non-significant but becoming negative. We observed a similar significant change for physical activity: BE-BROAD displayed a non-significant but stronger positive SNP-r_g than BE-BROAD$_{NAAN}$. Such findings suggest that some of the originally observed genetic signal may be attributed to AN cases.

7. Influence of BMI

The role of BMI in eating disorders is complex, with low BMI being pathognomonic of AN and individuals with binge-eating disorder often being overweight [68]. If the genetics of BMI were the only genetic cause of BE and AN (in opposite directions), then our GWASs would just be proxy GWASs for BMI.

GWAS-by-subtraction

In cohorts restricted to those not ascertained by AN, BMI accounted for 22% of the genetic variance in BE-BROAD.

We calculated SNP-r_g between the full BE-BROAD and AN phenotypes and their *NonBMI* components to assess how subtracting BMI affects the relationship with other traits (Supplementary Figure 18, Supplementary Table 14). Among psychiatric traits, the BE-BROAD *NonBMI* factor had a higher positive SNP-r_g with schizophrenia, but a lower SNP-r_g with ADHD. The SNP-r_g of BE-BROAD with educational attainment increased and became significant after subtracting BMI, and the association with self-rated health became non-

significant. Subtraction of BMI-related genetic risk attenuated positive SNP-r_g between BE-BROAD and most metabolic and anthropometric phenotypes, except childhood obesity and childhood BMI, which still showed significantly positive SNP-r_g with BE-BROAD.

We see a mirrored pattern in the *NonBMI* AN factor: first, subtracting BMI significantly increased correlations between AN and several psychiatric phenotypes, including ADHD, posttraumatic stress disorder symptoms, and cross-disorder risk. Furthermore, subtracting BMI significantly reduced associations between AN and educational attainment. We observed reduced associations between AN and most other metabolic and anthropometric phenotypes, with only waist-hip ratio, body fat percentage, fat mass, fasting insulin, and BMI-adjusted fasting insulin still being significantly negatively correlated with *NonBMI* AN.

Most of the anthropometric genetic correlations with BE-BROAD and with AN were reduced following our subtraction of the genetic variance of BMI. However, some correlations remained significant, such as those between BE-BROAD and childhood BMI, and of AN with fat mass and body fat percentage. Similarly, the metabolic genetic associations with AN were attenuated to non-significance when subtracting BMI, except for the correlation with fasting insulin (age-and-sex adjusted and BMI-adjusted). In contrast, with a few exceptions like the AN-ADHD genetic correlation discussed above, genetic correlations with psychiatric traits were largely unaffected by subtracting the BMI component. Even when BMI is accounted for, BE and AN are genetically distinct psychiatric phenotypes. Similar patterns of SNP-r_g between the EDs and other psychiatric disorders (with the notable exception of OCD) persist, as do differing patterns of SNP-r_g with impulsive traits (positive with BE-BROAD, not correlated with AN), and with education and fasting insulin levels (positive with AN, not correlated with BE-BROAD).

Overall, BMI accounts for a small but meaningful portion of BE-BROAD and AN risk, acting in opposing directions. Genetic correlations between BE-BROAD and AN, and

between the EDs and associated traits converge when assessing the non-BMI components. When considering specific SNPs, there were no major changes in patterns of associations after accounting for BMI. Ultimately, this leads to the question of, beyond BMI, what differentiates risk for BE from that of AN? Though patterns of genetic correlation converge in the *NonBMI* analysis, certain traits show notable divergence, including OCD (associated with AN and not with BE-BROAD), and risk tolerance (associated with BE-BROAD and not with AN), highlighting a potential influence of biological predisposition relevant to uncertainty and risk in differentiating BE and AN risk. Accounting for BMI affected the genetic correlation of EDs with diverse traits, not just with anthropometric traits, indicating that shared risk with anthropometric factors may be relevant to many psychological, psychiatric, and behavioural traits.

One major consideration in understanding how BMI, specifically, impacts genetic risk for AN, is that low BMI is definitional to an AN diagnosis in the current samples. However, there is ongoing debate over the degree to which low BMI is a necessary diagnostic criterion. For instance, individuals may lose significant amounts of weight and experience all other hallmark symptoms of AN and yet not receive this diagnosis. Recent research suggests that this presentation (currently termed 'atypical AN') may be quite common and, compared to AN with low weight presentations, has similarly debilitating symptoms, medical complications, and non-ED comorbidities [69]. Relevant to the current analysis, genetic associations between AN and (low)BMI and associated traits may be higher in the current sample as compared to an ascertainment and phenotyping strategy in which low BMI is not central to an AN case definition.

With regards to BE-BROAD, a phenotyping and ascertainment strategy which included individuals across the weight spectrum (including a potential oversampling of those with binge eating who also meet AN criteria), and focused on behavioural indicators rather than diagnostics, suggests that the positive associations between BEBROAD and BMI are likely to be robust.

Mendelian randomisation

Summary

We conducted two-sample Mendelian randomisation analyses of BE-BROAD and AN with BMI, testing causal effects in both directions. For each analysis, we used the full BE-BROAD or AN GWAS, and the GWAS of the respective *non-BMI* component (Supplementary Table 15). Significant results were found in both directions between increased BE-BROAD risk and increased risk for higher BMI. BE-BROAD was still associated with increased risk for higher BMI when using just the *non-BMI* component. When examining the effect of BMI on the *non-BMI* component of BE-BROAD, odds ratios (ORs) were consistently >1, but the different methods were not consistently significant. Significant results were found in both directions between increased risk of AN and decreased BMI. However, the *non-BMI* component of AN risk was not associated with BMI, and results were inconsistent across MR methods when examining the effect of BMI on the *non-BMI* component of AN.

Exposure: BE-BROAD, Outcome: BMI

Of a total of 41 SNPs associated with BE-BROAD at $P<5x10^{-6}$, MR-PRESSO identified 22 as pleiotropic. These were excluded from further analysis, leaving an instrumental variable (IV) of 19 SNPs. All these SNPs are strong instruments (minimum F-value 19.47). Neither heterogeneity nor pleiotropy were indicated by the relevant tests. Multiple methods supported the hypothesis that BE-BROAD is causal for higher BMI (Supplementary Figure 27a, Supplementary Table 15).

Exposure: *NonBMI* BE-BROAD, Outcome: BMI

Of the 28 SNPs with $P<5x10^{-6}$, 27 were present in the summary statistics for BMI. Ten SNPs were classified as pleiotropic by MR PRESSO. Thus, an IV was formed with 17 SNPs. All SNPs can be classified as strong instruments (minimum F-value 21.09). Tests

indicated possible heterogeneity and pleiotropy, but multiple methods consistently supported the hypothesis that BE-BROAD is causal for a higher BMI, even after accounting for shared genetics (Supplementary Figure 27c, Supplementary Table 15).

<u>Exposure: AN, Outcome: BMI</u>

There were 84 independent SNPs available as an IV (P<5x10^{-6}). MR-PRESSO identified 16 SNPs as pleiotropic and these SNPs were excluded from the analysis, leaving an IV formed from 68 SNPs. All these SNPs can be considered as strong instruments (minimum F-value 17.81). The Q-test indicated that the IV was heterogeneous, therefore attention should be focused on the heterogeneity robust methods when looking at the results. The MR-Egger intercept did not indicate pleiotropy, but the MR-PRESSO global test was significant. However, no pleiotropic SNPs were identified. Multiple methods supported the hypothesis that AN is causal for lower BMI (Supplementary Figure 28a, Supplementary Table 15).

<u>Exposure: *NonBMI* AN, Outcome: BMI</u>

A total of 67 independent SNPs (P<5x10^{-6}) associated with *NonBMI* AN were available. 15 SNPs were classified as pleiotropic by MR-PRESSO and were therefore excluded, so the final IV was constructed from 51 SNPs (minimum F-value 20.85). The Q-test showed that IV was significantly heterogeneous. The intercept of MR-Egger was not significant, but the global test of MR-PRESSO indicated horizontal pleiotropy. No methods yielded an estimate significantly different from zero, and so there was no support for the hypothesis that AN has a causal effect on BMI after accounting for shared genetics (Supplementary Figure 28c, Supplementary Table 15).

<u>Exposure: BMI, Outcome: BE-BROAD</u>

For BMI acting on BE-BROAD, 670 independent SNPs (P<5x10^{-9}) were available as an IV. Of these, 95 were not present in the summary statistics for BE-BROAD, but 58

proxies were available. Of the 633 SNPs, 6 SNPs were classified as pleiotropic by MR-PRESSO and excluded to leave 627 SNPs (minimum F-value 25.00). The Q-test indicated heterogeneity, and pleiotropy was also probable. Multiple methods were consistent with the hypothesis that there may be a causal effect of a higher BMI on a higher risk of BE (Supplementary Figure 27b, Supplementary Table 15).

Exposure: BMI, Outcome: *NonBMI* BE-BROAD

An IV was constructed from 642 SNPs (minimum F-value 25.00): out of the 670 index SNPs for BMI, 76 were not available in the *NonBMI* BE-BROAD summary statistics, 53 proxy SNPs were included, and four SNPs were excluded as pleiotropic. Both pleiotropy and heterogeneity were indicated by statistical tests. Although point estimates were consistently OR>1 (which would be in support of the hypothesis that higher BMI causally increases the risk of BE), the different methods are not consistently significant and therefore no statement can be made as to whether BMI causally affects BE after accounting for shared genetics (Supplementary Figure 27d, Supplementary Table 15).

Exposure: BMI, Outcome: AN

For BMI acting on AN, 670 independent SNPs ($P<5\times10^{-9}$) were available as an IV. Of these SNPs, 11 were identified as pleiotropic by MR-PRESSO and therefore excluded, and 71 were not available in summary statistics for AN. Proxies were found for 49 unavailable SNPs, resulting in an IV of 642 SNPs (minimum F-value 25.00). The Q-test indicated heterogeneity. The MR-Egger intercept was not significant, but the MR-PRESSO global test result was consistent with horizontal pleiotropy, although MR-PRESSO did not detect further pleiotropic SNPs. Robust methods supported the hypothesis that low BMI causally increases the risk of AN (Supplementary Figure 28b, Supplementary Table 15).

Exposure: BMI, Outcome: *NonBMI* AN

The same 642-SNP IV was available for BMI as in the BMI-to-AN analysis above. Again, tests indicated heterogeneity of the IV and pleiotropy. Although point estimates were consistently OR>1 (which would be in support of the hypothesis that *higher* BMI causally increases the risk of AN), the different methods are not consistently significant and therefore no statement can be made as to whether there is an effect of BMI on AN after accounting for shared genetics (Supplementary Figure 28d, Supplementary Table 15).

8. S-PrediXcan

We report results with a focus on central nervous system tissues (brain and cervical spinal cord) and gastrointestinal tissues (oesophagus, colon, stomach, and small intestine), as these are tissue types of a priori interest in eating disorders.

BE-BROAD

In addition to two experiment-wide significant results (Main Text), 41 gene-tissue associations (representing 17 unique genes) were significant at the within-tissue threshold (Supplementary Table 16). Among these, 11 within-tissue associations were in the central nervous system, and five in gastrointestinal tissues.

To test whether tissue association patterns were significant or merely artifactual findings reflecting uneven power across sample sizes, we performed exact binomial tests to assess tissue enrichment in our associations (Supplementary Table 31). None of these tests were significant after accounting for multiple testing.

AN

In addition to 300 experiment-wide significant gene-tissue associations (Main Text), 280 gene-tissue associations were significant at a within-tissue threshold (Supplementary Table 16).

In exact binomial tests for tissue enrichment in AN, no tests were significant after accounting for multiple testing (Supplementary Table 31).

BE-NARROW

For BE-NARROW, we observed nine experiment-significant gene-tissue associations in two loci on chromosome 3 (four genes: *HADHA*, *KLHDC8B*, *PODXL2*, *PRKAR2A*; nine tissues) with the most significant association in *KLHDC8B*-Heart, Atrial appendage (P=2.08x10^{-12}; Supplementary Figure 19, Supplementary Table 16).

In exact binomial tests for tissue enrichment in BE-NARROW, no tests were significant after accounting for multiple testing (Supplementary Table 31).

AN subtypes

For AN-BP subtype S-PrediXcan, we found eight experiment-significant gene-tissue associations in two loci encompassing three genes (*CALML6*, *KLHDC8B*, *PRKAR2A*) and eight tissues, with the most significant association in *PRKAR2A*-Sigmoid colon (P=1.33x10^{-13}; Supplementary Figure 19, Supplementary Table 16). At a tissue-specific significance threshold, there were 108 gene-tissue associations (14 genes: *C3orf62*, *CALML6*, *CELSR3*, *DALRD3*, *HSPA2*, *KLHDC8B*, *MLKL*, *NEDD4*, *NICN1*, *PPOX*, *PRKAR2A*, *RP11-57206.1*, *ZBTB25*, *ZNF33A*; 44 tissues).

In AN-R, we observed one experiment-significant hit in *DLX1* (chr2) in brain frontal cortex tissue (P=5.96x10^{-8}; Supplementary Figure 19, Supplementary Table 16). An

additional 25 gene-tissue associations were significant at a tissue-specific significance level (five genes: *DLX1*, *PRKAR2A*, *LIPT2*, *EPM2A*, *NBPF20*; 23 tissues).

No tests were significant in exact binomial tests for tissue enrichment after accounting for multiple testing (Supplementary Table 31).

9. S-MultiXcan

We ran S-MultiXcan using harmonised GWAS summary statistics and S-PrediXcan MASHR model output for each of our ED traits for all genes (N=22,241).

BE-BROAD

S-MultiXcan results of BE-BROAD phenotype found ten significantly associated genes (eight loci, six chromosomes) (P<2.22x10^{-6}), with the most significant association with gene *SAP30* on chromosome 4 (P=6.97x10^{-46}; Supplementary Figure 20, Supplementary Table 17).

BE-NARROW

Sixteen genes were significantly associated (13 loci, 10 chromosomes) with the BE-NARROW phenotype (P<1.40x10^{-6}), with gene *SPTBN2* on chromosome 11 most significantly associated (P=1.45x10^{-34}; Supplementary Figure 20, Supplementary Table 17).

AN

Forty-three genes across 14 loci on 13 chromosomes were significantly associated with AN phenotype (P<1.45x10^{-6}), with the most significant association of AN with the chromosome 3 gene *USP19* (P=2.00x10^{-40}; Supplementary Figure 20, Supplementary Table 17).

AN subtypes

For AN subtypes, nine genes were significantly associated with AN-BP (four loci, three chromosomes) ($P<4.87\times10^{-7}$) with the most significant association on chromosome 14 with gene *AKAP5* ($P=1.31\times10^{-15}$). For AN-R, there were eight genes (eight loci, seven chromosomes) significantly associated with the phenotype ($P<1.83\times10^{-6}$), with the top gene *CCNB1IP1* on chromosome 14 ($P=7.38\times10^{-24}$; Supplementary Figure 20, Supplementary Table 17).

10. MAGMA

BE-NARROW

In the gene analysis for BE-NARROW, MAGMA identified 13 genes significant after Bonferroni correction ($P<2.58\times10^{-6}$; Supplementary Table 18). As in BE-BROAD, the FTO gene had the strongest association ($P=2.10\times10^{-17}$). The MAGMA gene set and drug set analyses did not yield significant results, but drug-class analyses identified enrichment in corticosteroids (particularly those in group III, potent) as well as enrichment in anxiolytics (Supplementary Table 21).

AN subtypes

In the gene analysis for AN-R, MAGMA identified 6 genes significant after Bonferroni correction ($P<2.59\times10^{-6}$), all in the same locus on chromosome 2q31 (Supplementary Table 18). The MAGMA gene set and drug set analyses did not yield significant results, but drug-class analyses identified enrichment in hypnotics and sedatives, and anti-epileptic drugs, as well as in multiple classes of anti-inflammatory drugs (Supplementary Table 21).

In the gene analysis for AN-BP, MAGMA identified 11 genes significant after Bonferroni correction ($P<2.59\times10^{-6}$) across two loci – the gene-dense locus on chromosome 3p21 and a locus on chromosome 14q23 (Supplementary Table 18). The MAGMA gene set

and drug-class analyses did not identify Bonferroni-corrected significant gene sets or drug classes, but the targets of inositol (vitamin B8) were enriched for signal (Supplementary Table 20).

11. Cell and tissue gene expression specificity

We used stratified LDSC to estimate the enrichment of SNP-based heritability for BE-BROAD and AN among genes specifically expressed in GTEx human tissues and in human brain cell types from the Human Brain Atlas. After accounting for multiple testing, none of the associations was significant. However, we observed two notable trends. First, both phenotypes had greater enrichment in brain tissues than in other tissues. Second, the strongest relationship was with cortical excitatory pyramidal neurons in BE-BROAD (supercluster "Upper_layer_intratelencephalic"). For AN, we mainly observed enrichment in the cortical and subcortical inhibitory neurons represented in five superclusters.

12. Polygenic prediction

Association

We tested whether higher BE-BROAD and AN PRS were associated with a higher risk of BE-BROAD and AN, using a leave-one-study-out design (Methods; Supplementary Table 25). BE-BROAD PRS was significantly associated with BE-BROAD (average OR=1.11, average 95% confidence interval [CI] across studies: 1.08–1.14; *P* range: 2.38×10^{-15}–0.022; Supplementary Figure 23). AN PRS was significantly associated with AN (average OR=1.50, average 95% CI 1.42–1.59, all $P < 2 \times 10^{-16}$; Supplementary Figure 23).

Liability-scale R^2

BE-BROAD

The average liability-scale variance of BE-BROAD explained by BE-BROAD PRS was 0.32% (range 0.08% – 2.02%), compared to 0.29% (range 0.09% – 1.35%) by BE-NARROW PRS and 0.53% (range 0.01% – 4.84%) by AN PRS, respectively (Supplementary Table 25). After excluding cohorts in which all the BE-BROAD cases also have AN diagnosis (*aunz, sedk*), the average liability-scale variance of BE-BROAD explained by BE-BROAD PRS did not change (0.32%, range 0.08% – 2.02%), but that explained by AN PRS dropped to 0.12% (range 0.01% – 1.6%), suggesting a larger variance of BE-BROAD being explained by BE-BROAD PRS than AN PRS in individuals with binge-eating behaviours who do not have AN.

BE-NARROW

Likewise, the average liability-scale variance of BE-NARROW explained by BE-NARROW PRS was 0.64% (range 0.19% – 1.81%), compared to 0.73% (range 0.21% – 1.99%) by BE-BROAD PRS and 0.70% (range 0.01% – 2.44%) by AN PRS, respectively (Supplementary Table 25). After excluding cohorts in which all the BE-BROAD cases also have AN diagnosis (*aunz, sedk*), the average liability-scale variance of BE-NARROW explained by BE-NARROW PRS and BE-BROAD PRS changed minimally to 0.57% (range 0.19% – 1.81%) and 0.67% (range 0.21% – 1.99%), respectively, but dropped to 0.46% (range 0.01% – 2.02%) for the liability-scale R^2 of BE-NARROW explained by AN PRS.

AN

The average liability-scale variance of AN explained by AN PRS was 2.32% (range 1.21% – 4.72%), compared to 0.32% (range 0.06% – 0.85%) by BE-BROAD PRS and 0.29% (range 0.14% – 0.73%) by BE-NARROW PRS, respectively (Supplementary Table 25).

PRS predictive performance

We evaluated predicted performance of PRS by subtracting the ROC AUC (or PR AUC) of the null model (i.e., a model with only genetic PCs as predictors) from the ROC AUC (or PR AUC) of the full model (i.e., a model with PRS and genetic PCs as predictors). Given a random prediction model has a ROC AUC of 0.5 and a perfect prediction model has a ROC AUC of 1, we observed wide variability in the null model such that ROC AUC varied from 0.519 to 0.828 when only using genetic PCs to predict BE-BROAD and varied from 0.525 to 0.864 when only using genetic PCs to predict AN. Since some of our cohorts are very imbalanced in terms of case:control ratio (i.e., proportion of cases ranges from 0.3% to 52% for AN and 0.1% to 59% for BE-BROAD), we included PR AUC in addition to ROC AUC to evaluate prediction performance as PR AUC provides a more accurate evaluation of prediction performance in imbalanced datasets, in which ROC AUC may provide misleading conclusions on prediction performance due to false interpretation on specificity [70].

BE-BROAD

Individuals with 1SD higher BE-BROAD PRS had an average OR of 1.11 for BE-BROAD (average 95% CI: 1.08 – 1.14, all $P \leq 0.022$), an average OR of 1.20 for BE-NARROW (average 95% CI: 1.14 – 1.27, all $P \leq 7.14 \times 10^{-7}$), and an average OR of 1.16 for AN (average 95% CI: 1.10 – 1.22, all $P \leq 0.023$; Supplementary Figure 23, Supplementary Table 25).

BE-BROAD PRS contributed to an average increase of 0.009 (range: 0.005 - 0.027) in the area under the receiver operating characteristic curve (ROC AUC) and 0.004 (range: 0.001 - 0.019) in the precision-recall curve (PR AUC) for BE-BROAD prediction. In addition, it contributed to an average increase of 0.013 (ROC AUC) and 0.004 (PR AUC) for BE-NARROW prediction and an average increase of 0.010 (ROC AUC) and 0.005 (PR AUC) for AN prediction (Supplementary Table 25).

BE-NARROW

Individuals with 1 SD higher BE-NARROW PRS had an average OR of 1.18 for BE-NARROW (Supplementary Figure 23; average 95% CI: 1.12 – 1.25, all $P \leq 1.72 \times 10^{-5}$), an average OR of 1.11 for BE-BROAD (average 95% CI: 1.08 – 1.14, all $P \leq 8.40 \times 10^{-9}$ except *alsp* where P=0.058), and an average OR of 1.15 for AN (average 95% CI: 1.09 – 1.21, all $P \leq 6.62 \times 10^{-4}$; Supplementary Figure 23, Supplementary Table 25).

Using BE-NARROW PRS, the average improvement in AUC was 0.012 (ROC) and 0.004 (PR) to predict BE-NARROW, 0.01 (ROC) and 0.004 (PR) to predict BE-BROAD, and 0.009 (ROC) and 0.005 (PR) to predict AN.

AN

Individuals with 1 SD higher AN PRS had an average OR of 1.50 for AN (average 95% CI: 1.42 – 1.59, all $P \leq 2 \times 10^{-16}$), an average OR of 1.10 for BE-BROAD (average 95% CI: 1.07 – 1.13, all $P \leq 4.54 \times 10^{-3}$ except *alsp* where P=0.11), and an average OR of 1.17 for BE-NARROW (average 95% CI: 1.11 – 1.23, all $P \leq 4.23 \times 10^{-6}$ except *moba* where P=0.176; Supplementary Figure 23, Supplementary Table 25). This suggests a diminished association of AN PRS with BE in large population-based cohorts where individuals with BE mostly do not have AN (e.g., *moba, alsp*). Indeed, after excluding the two cohorts where all BE cases are AN-BE cases (*aunz, sedk*), individuals with 1 SD higher AN PRS had a lowered average OR of 1.05 for BE-BROAD (average 95% CI: 1.02 – 1.08), and a lowered average OR of 1.12 for BE-NARROW (average 95% CI: 1.06 – 1.17).

AN PRS contributed to an average increase of 0.046 (range: 0.006 - 0.084) in ROC AUC and 0.034 (range: 0.001 - 0.073) in PR AUC for AN prediction. Among all prediction models tested, the prediction of AN using AN PRS produced the highest prediction improvement (an average increase of 0.046 in ROC AUC and an average increase of 0.034 in PR AUC). The average improvement in AUC to predict BE-BROAD using AN PRS was

0.011 (ROC) and 0.005 (PR), and 0.012 (ROC) and 0.005 (PR) to predict BE-NARROW. When excluding cohorts where all the BE cases also have AN diagnosis (*aunz, sedk*), the average improvement in AUC for AN PRS to predict BE-BROAD dropped substantially to 0.004 (ROC) and 0.0004 (PR) and dropped to 0.004 (ROC) and 0.001 (PR) to predict BE-NARROW, suggesting little predictive capability of AN PRS on binge-eating behaviour outside of the context of AN.

Sex-specific PRS

BE-BROAD

We examined whether the effect of BE-BROAD PRS on BE-BROAD was different in males versus in all samples across three selected cohorts. We found that the OR of BE-BROAD per 1 SD higher BE-BROAD PRS in males in *ukd2* was 1.20 [95% CI: 1.09, 1.33], compared to OR of 1.23 [95% CI: 1.17, 1.30] in all samples in *ukd2* (P_{diff}=0.68). In *moba*, the OR was 1.15 [95% CI: 0.94, 1.40] in males, with a wide confidence interval due to small case number (N=98), and 1.06 [95% CI: 1.05, 1.08] in all samples (P_{diff}=0.47). In *alsp*, the OR was 1.06 [95% CI: 0.91, 1.24] in males and 1.09 [95% CI: 1.01, 1.18] in all samples (P_{diff}=0.74). Given the PRS result in males was not significant in both *moba* (*P*=0.18, N_{case}=98) and *alsp* (*P*=0.43, N_{case}=179) due to small samples, future studies with more male BE cases are required to explore if there is any sex-specific genetic difference in binge-eating behaviour (Supplementary Table 26, Supplementary Figure 24).

AN

The OR of AN per 1 SD higher AN PRS in males in *fngn* was 1.07 [95% CI: 0.96, 1.20], compared to OR of 1.32 [95% CI:1.26, 1.39] in all samples in *fngn* (P_{diff}=8.75x10^{-4}). In *ipsy*, the OR was 1.41 [95% CI: 1.23, 1.60] in males and 1.49 [95% CI: 1.44, 1.54] in all samples (P_{diff}=0.40). In *ukb2*, the OR was 1.09 [95% CI: 0.90, 1.31] in males and 1.45 [95% CI: 1.38, 1.54] in all samples (P_{diff}=0.003). Given the limited data, it is hard to draw a

definitive conclusion on the transferability of AN PRS based on female-only GWAS for AN risk prediction in male individuals. Future studies with more samples and data from men with AN are needed to confirm whether there is a sex-specific genetic effect on AN (Supplementary Table 26, Supplementary Figure 24).

Subgroup PRS

We assessed whether BE-BROAD and AN PRS differed across BE and AN subgroups, comparing control individuals to (a) those with BE-BROAD only; (b) with both BE-BROAD and AN (BE+AN); and (c) with AN only. Overall, we found both BE-BROAD and AN PRS to be elevated in all subgroups compared to controls ($P \leq 0.019$). Among the subgroups, the BE+AN and BE-BROAD-only groups did not significantly differ on BE-BROAD PRS, and typically had a significantly higher BE-BROAD PRS than the AN-only group (Supplementary Figure 25). The BE+AN and AN-only groups did not significantly differ from each other on AN PRS, while the BE-BROAD-only group had a significantly lower AN PRS than both other groups in one study, but not in another (Supplementary Figure 25, Supplementary Table 27). Our results are consistent with BE-BROAD PRS predicting BE-BROAD both in comparison to controls and in the context of AN. Similarly, AN PRS is broadly able to distinguish individuals with AN from controls and in the context of broadly defined BE.

We also assessed the difference in BE-NARROW PRS across the three subgroups. Overall, we found BE-NARROW PRS to be elevated in all subgroups compared to controls ($9.76x10^{-19}$ $9.76x10^{-19} \leq P \leq 0.036$). Among the subgroups, comorbid and BE-BROAD-only subgroups tended to have the highest BE-NARROW PRS with no significant difference across these two groups ($P \geq 0.19$). The AN-only group tended to have lower BE-NARROW PRS, although the difference between the AN-only and the comorbid groups was only significant in *sedk* ($P=1.10x10^{-4}$) and that between AN-only and BE-BROAD-only groups was only significant in *ukb2* ($P=1.9x10^{-4}$; Supplementary Table 27, Supplementary Figure 25).

13. Variant heterogeneity in AN

Mean I^2 across variants was lower in Watson et al (7.52%, SD=11.13%) than in our main AN analysis (7.96%, SD=11.97%), and was considerably higher in the new AN cohorts (10.5%, SD=15.95%). Bins of I^2 are shown in Supplementary Table 28. More variants were present in higher bins (>25%) in the main AN analysis and the new AN cohorts than in Watson et al, supporting the assertion that the new AN cohorts introduced greater heterogeneity into the analyses. Analyses of shared variants across the three sets of summary statistics showed good concordance in I^2 bins between analyses, with a general pattern of higher I^2 in the main AN analysis and the new AN cohorts than in Watson et al (Supplementary Table 28). Sensitivity analyses limited to variants in linkage equilibrium and variants associated with AN in Watson et al were consistent with the unrestricted analyses (Supplementary Table 28).

14. Power analyses (AN-R, AN-BP, BE-NARROW)

We had 80% power to detect a genotypic relative risk of 1.05-1.26 for BE-BROAD, 1.09-1.44 for BE-NARROW, 1.07-1.36 for AN, 1.21-2.19 for AN-R, and 1.15-1.81 for AN-BP. The AN GWAS of Watson et al had 80% power to detect a genotypic relative risk of 1.09-1.46, indicating an increase in power with our analyses, although we caution that these analyses assume all cases contribute equally, which is unlikely in this instance (Main Discussion, Heterogeneity analyses in Supplementary Results).

Supplementary Figures

Supplementary Figures 1-6: Forest plots for the six genome-wide significant associations with BE-BROAD in order of position on the genome

Each row represents one cohort, with the final row showing the meta-analytic result. Initial information gives the variant name, effect and non-effect alleles, and variant position, followed by a summary of directions of effect, heterogeneity p-value, and heterogeneity I statistic. Main data columns include the imputation information metric (info), the association p-value, the frequency of the variant in cases (f_ca) and in controls (f_co), and the log odds ratio and standard error of the association, which are also visualised in the plot as the point (log odds ratio, with width proportional to N) and bars (95% confidence interval, 1.96 standard errors). The genotyping indicator (ngt) was set to missing for all cohorts and should be ignored.

Supplementary Figure 1

**rs7541513**		**A/G**		**1:75014195**			
		−+++−+++−++++??−+		het_P:	0.355	het_I:	8.8
	**ngt**	**info**	**p_value**	**f_ca(n)**	**f_co(n)**	**ln(OR)**	**STDerr**
dan_agds.BEBROAD.DENTIST.	–	1.00	0.71	0.423(407)	0.430(6850)	−0.0274	0.0736
dan_alsp.BEBROAD.DENTIST.	–	1.00	0.849	0.436(840)	0.429(2861)	0.0106	0.0558
dan_aunz.BEBROAD_new.b37.DENTIST.	–	1.00	0.361	0.432(1416)	0.423(15626)	0.0364	0.0399
dan_biov.BE.DENTIST.	–	1.00	0.512	0.460(87)	0.432(61474)	0.1	0.153
dan_chop.BEBROAD.DENTIST.	–	1.00	0.0711	0.404(368)	0.423(3568)	−0.148	0.0818
dan_esbb.BEBROAD.b37.noChrX.DENTIST.	–	0.98	0.0422	0.411(727)	0.387(206468)	0.109	0.0539
dan_fngn.BE.b37.noChrX.DENTIST.	–	0.99	0.322	0.472(770)	0.459(301716)	0.0513	0.0518
dan_ipsy.BE.DENTIST.	–	1.00	0.0845	0.437(466)	0.413(20439)	0.116	0.0673
dan_jans.BENARROW.b37.noChrX.DENTIST.	–	1.01	0.963	0.402(214)	0.414(902)	−0.0052	0.111
dan_moba.BEBROAD.DENTIST.	–	0.91	1.18e−07	0.425(24094)	0.412(150480)	0.0546	0.0103
dan_sebe.BEBROAD.noChrX.DENTIST.	–	1.01	0.606	0.419(562)	0.414(577)	0.0462	0.0896
dan_sedk.BEBROAD.DENTIST.	–	0.99	0.312	0.429(1757)	0.419(3793)	0.0427	0.0423
dan_ukb2.BEBROAD.DENTIST.	–	0.92	0.355	0.434(652)	0.423(445105)	0.0542	0.0586
dan_usa1.BEBROAD.DENTIST.	–	1.01	0.449	0.388(98)	0.412(750)	−0.121	0.16
dan_usa2.BEBROAD.b37.DENTIST.	–	1.00	0.00719	0.473(606)	0.426(1296)	0.191	0.0711
**dan_**	**–**	**0.95**	**2.65e−09**	**0.427(39279)**	**0.425(1227436)**	**0.0523**	**0.0088**

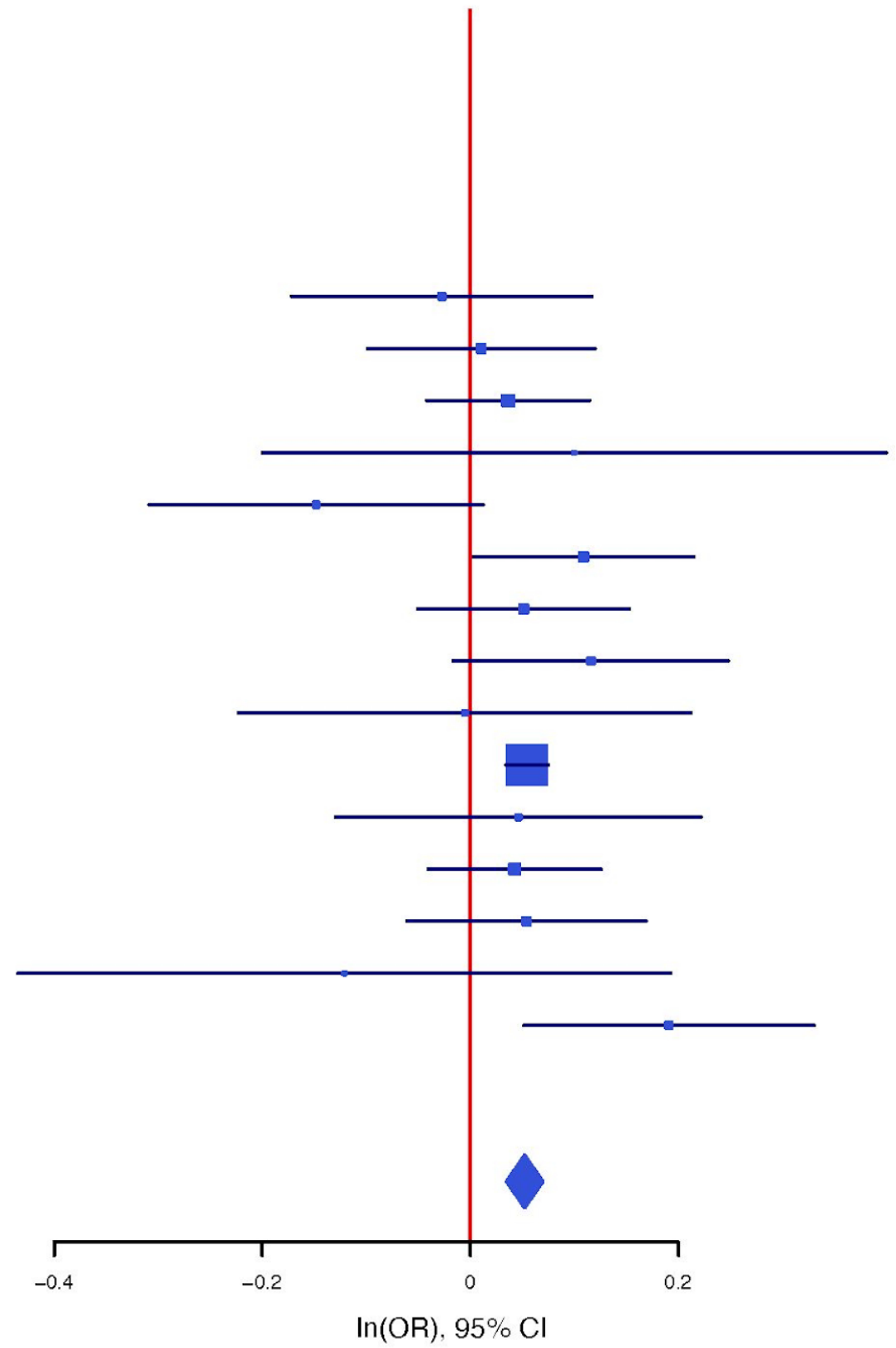

Supplementary Figure 2

rs1456193		C/T		3:117637697			
		---++----------+-		het_P:	0.649	het_I:	0.0
	ngt	info	p_value	f_ca(n)	f_co(n)	ln(OR)	STDerr
dan_agds.BEBROAD.DENTIST.	–	0.94	0.244	0.832(407)	0.816(6850)	0.112	0.0959
dan_alsp.BEBROAD.DENTIST.	–	1.00	0.739	0.180(840)	0.181(2861)	0.0241	0.0724
dan_aunz.BEBROAD_new.b37.DENTIST.	–	1.00	0.000406	0.845(1416)	0.818(15626)	0.191	0.0541
dan_biov.BE.DENTIST.	–	0.99	0.646	0.810(87)	0.822(61474)	−0.0915	0.2
dan_chop.BEBROAD.DENTIST.	–	1.00	0.207	0.817(368)	0.830(3568)	−0.132	0.104
dan_esbb.BEBROAD.b37.noChrX.DENTIST.	–	1.00	0.788	0.819(727)	0.818(206468)	0.0182	0.0679
dan_fngn.BE.b37.noChrX.DENTIST.	–	1.00	0.215	0.774(770)	0.761(301716)	0.0749	0.0605
dan_ipsy.BE.DENTIST.	–	0.99	0.205	0.827(466)	0.813(20439)	0.112	0.0883
dan_jans.BENARROW.b37.noChrX.DENTIST.	–	1.03	0.867	0.827(214)	0.816(902)	0.0237	0.142
dan_moba.BEBROAD.DENTIST.	–	1.00	3.68e−06	0.823(24094)	0.814(150480)	0.0602	0.013
dan_sebe.BEBROAD.noChrX.DENTIST.	–	0.99	0.379	0.824(562)	0.806(577)	0.101	0.115
dan_sedk.BEBROAD.DENTIST.	–	1.03	0.784	0.818(1757)	0.817(3793)	0.0146	0.053
dan_ukb2.BEBROAD.DENTIST.	–	0.99	0.19	0.833(652)	0.819(445105)	0.0949	0.0725
dan_ukch.BEBROAD.b37.noChrX.DENTIST.	–	1.03	0.464	0.821(264)	0.805(1357)	0.0902	0.123
dan_ukd2.BEBROAD.b37.noChrX.DENTIST.	–	0.97	0.703	0.816(5951)	0.809(4174)	0.0189	0.0495
dan_usa1.BEBROAD.DENTIST.	–	1.00	0.892	0.847(98)	0.851(750)	−0.0293	0.216
dan_usa2.BEBROAD.b37.DENTIST.	–	0.99	0.69	0.825(606)	0.821(1296)	0.0371	0.0931
**dan_**	**–**	**1.00**	**3.25e−08**	**0.808(39279)**	**0.802(1227436)**	**0.0597**	**0.0108**

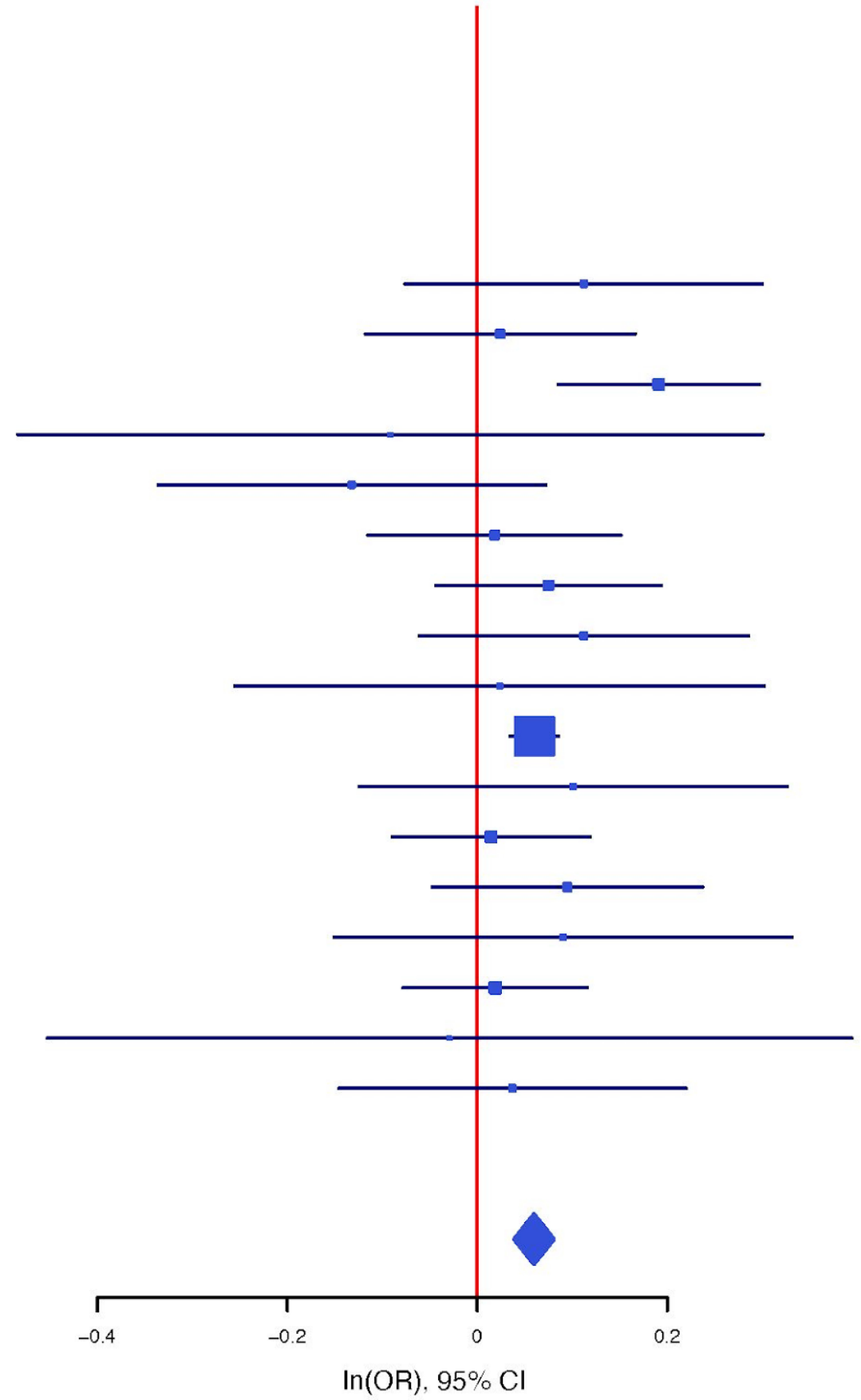

Supplementary Figure 3

**rs2925354**		**A/G**		**11:93206740**			
−+−−−−−+−−−−−−−−−				het_P:	0.959	het_I:	0.0
	**ngt**	**info**	**p_value**	**f_ca(n)**	**f_co(n)**	**ln(OR)**	**STDerr**
dan_agds.BEBROAD.DENTIST.	−	0.99	0.168	0.102(407)	0.118(6850)	−0.157	0.114
dan_alsp.BEBROAD.DENTIST.	−	0.99	0.728	0.123(840)	0.122(2861)	0.0294	0.0846
dan_aunz.BEBROAD_new.b37.DENTIST.	−	1.01	0.0669	0.109(1416)	0.120(15626)	−0.115	0.063
dan_biov.BE.DENTIST.	−	0.99	0.267	0.092(87)	0.118(61474)	−0.261	0.235
dan_chop.BEBROAD.DENTIST.	−	0.99	0.277	0.115(368)	0.128(3568)	−0.136	0.125
dan_esbb.BEBROAD.b37.noChrX.DENTIST.	−	1.00	0.516	0.095(727)	0.100(206468)	−0.057	0.0878
dan_fngn.BE.b37.noChrX.DENTIST.	−	1.00	0.589	0.108(770)	0.113(301716)	−0.0442	0.0817
dan_ipsy.BE.DENTIST.	−	0.99	0.928	0.123(466)	0.122(20439)	0.00928	0.102
dan_jans.BENARROW.b37.noChrX.DENTIST.	−	1.02	0.174	0.112(214)	0.130(902)	−0.233	0.171
dan_moba.BEBROAD.DENTIST.	−	0.99	2.02e−05	0.105(24094)	0.112(150480)	−0.0685	0.0161
dan_sebe.BEBROAD.noChrX.DENTIST.	−	1.10	0.206	0.102(562)	0.118(577)	−0.169	0.134
dan_sedk.BEBROAD.DENTIST.	−	0.98	0.0259	0.095(1757)	0.108(3793)	−0.157	0.0703
dan_ukb2.BEBROAD.DENTIST.	−	0.99	0.762	0.115(652)	0.116(445105)	−0.0261	0.0862
dan_ukch.BEBROAD.b37.noChrX.DENTIST.	−	0.96	0.96	0.118(264)	0.117(1357)	−0.00752	0.152
dan_ukd2.BEBROAD.b37.noChrX.DENTIST.	−	0.98	0.246	0.112(5951)	0.118(4174)	−0.0695	0.0599
dan_usa1.BEBROAD.DENTIST.	−	1.02	0.914	0.117(98)	0.117(750)	−0.0266	0.246
dan_usa2.BEBROAD.b37.DENTIST.	−	0.96	0.358	0.106(606)	0.117(1296)	−0.105	0.115
**dan_**	**−**	**0.99**	**4.91e−08**	**0.107(39279)**	**0.112(1227436)**	**−0.0726**	**0.0133**

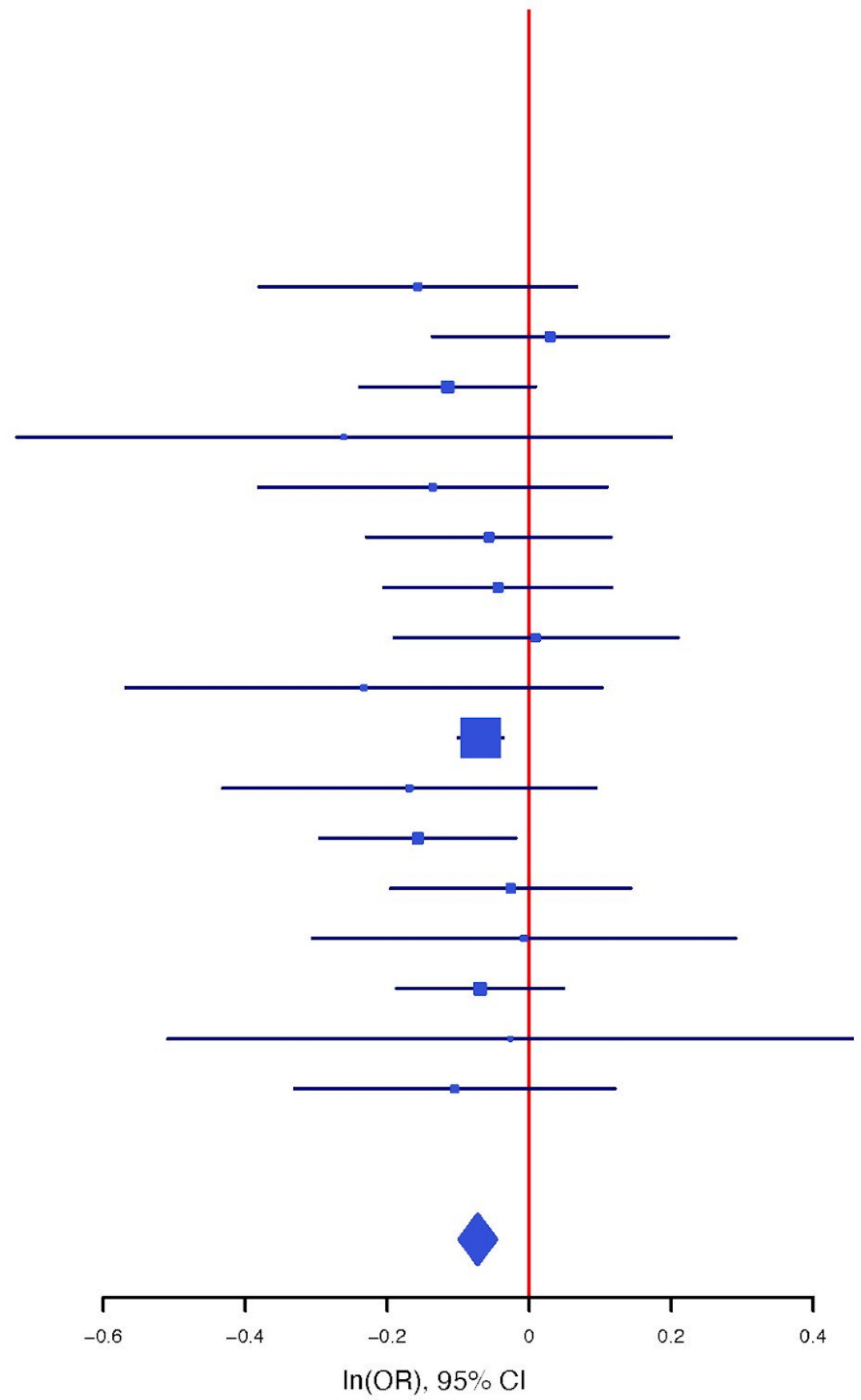

Supplementary Figure 4

**rs7953534**			**A/G**		**12:50197880**			
	+−−++?+++++++++−−				het_P:	0.0123	het_I:	49.8
	**ngt**	**info**	**p_value**	**f_ca(n)**	**f_co(n)**	**ln(OR)**	**STDerr**	
dan_agds.BEBROAD.DENTIST.	−	1.00	0.393	0.335(407)	0.321(6850)	0.0675	0.079	
dan_alsp.BEBROAD.DENTIST.	−	0.99	0.66	0.308(840)	0.319(2861)	−0.0264	0.06	
dan_aunz.BEBROAD_new.b37.DENTIST.	−	1.00	0.659	0.320(1416)	0.324(15626)	−0.0186	0.0423	
dan_biov.BE.DENTIST.	−	1.00	0.0404	0.385(87)	0.315(61474)	0.336	0.164	
dan_chop.BEBROAD.DENTIST.	−	1.01	0.00174	0.385(368)	0.318(3568)	0.258	0.0824	
dan_fngn.BE.b37.noChrX.DENTIST.	−	0.99	0.787	0.351(770)	0.348(301716)	0.0146	0.0542	
dan_ipsy.BE.DENTIST.	−	0.99	0.417	0.351(466)	0.334(20439)	0.0572	0.0705	
dan_jans.BENARROW.b37.noChrX.DENTIST.	−	0.96	0.0538	0.376(214)	0.325(902)	0.227	0.117	
dan_moba.BEBROAD.DENTIST.	−	1.00	2.19e−05	0.359(24094)	0.349(150480)	0.0449	0.0106	
dan_sebe.BEBROAD.noChrX.DENTIST.	−	0.99	0.000735	0.387(562)	0.322(577)	0.315	0.0932	
dan_sedk.BEBROAD.DENTIST.	−	1.01	0.0543	0.368(1757)	0.353(3793)	0.0824	0.0428	
dan_ukb2.BEBROAD.DENTIST.	−	0.97	0.906	0.324(652)	0.321(445105)	0.00716	0.0603	
dan_ukch.BEBROAD.b37.noChrX.DENTIST.	−	0.99	0.085	0.369(264)	0.331(1357)	0.173	0.1	
dan_ukd2.BEBROAD.b37.noChrX.DENTIST.	−	0.97	0.0126	0.326(5951)	0.312(4174)	0.103	0.0413	
dan_usa1.BEBROAD.DENTIST.	−	0.95	0.67	0.286(98)	0.309(750)	−0.0763	0.179	
dan_usa2.BEBROAD.b37.DENTIST.	−	1.00	0.989	0.321(606)	0.322(1296)	−0.00108	0.0756	
**dan_**	**−**	**0.98**	**1.4e−08**	**0.351(39279)**	**0.333(1227436)**	**0.0506**	**0.0089**	

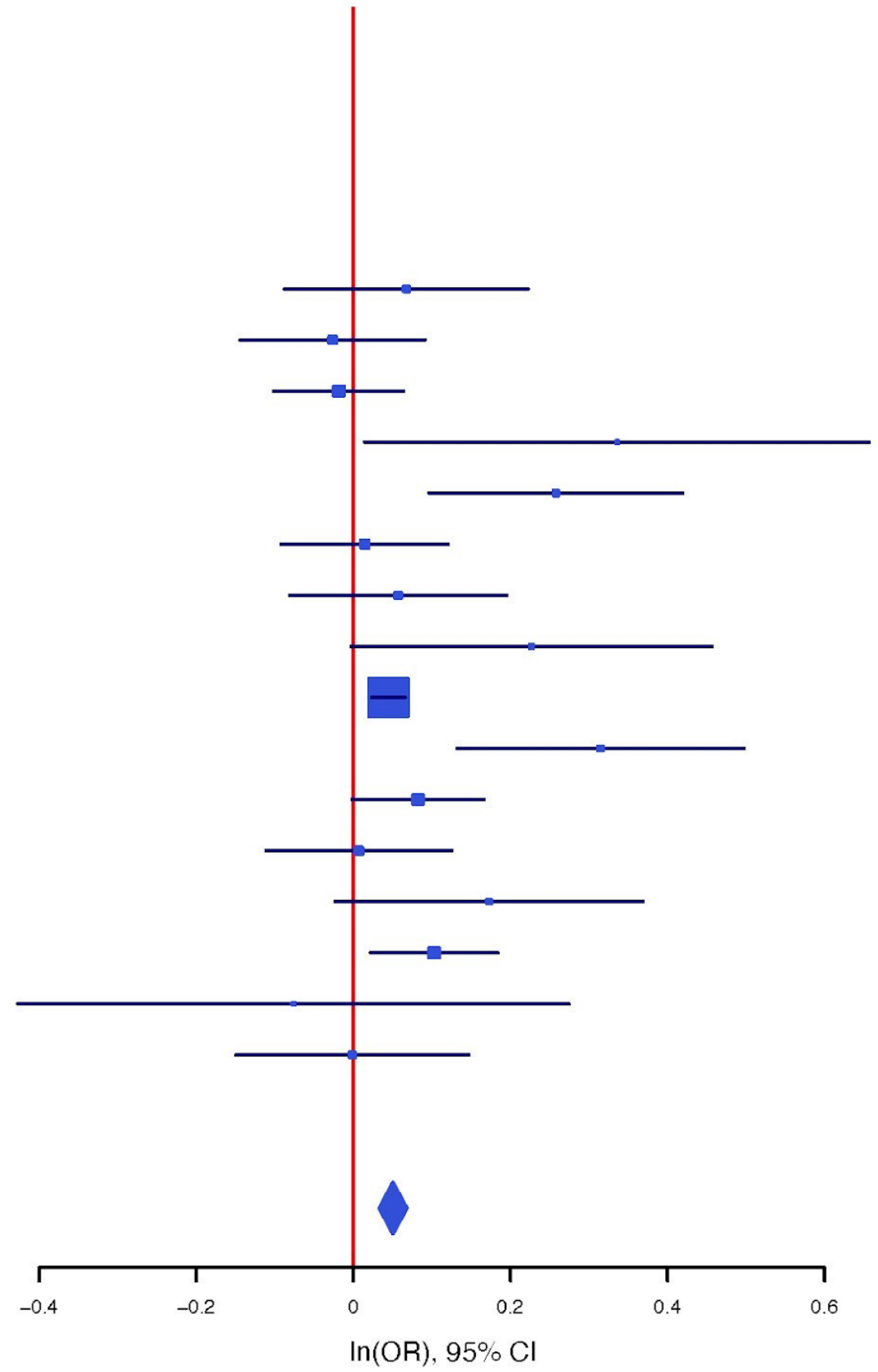

Supplementary Figure 5

rs11642015 **T/C** **16:53802494**

++++–+++–+++++++– het_P: 0.000659 het_I: 60.5

	ngt	info	p_value	f_ca(n)	f_co(n)	ln(OR)	STDerr
dan_agds.BEBROAD.DENTIST.	–	0.98	0.00902	0.448(407)	0.401(6850)	0.193	0.0738
dan_alsp.BEBROAD.DENTIST.	–	1.00	0.000198	0.446(840)	0.402(2861)	0.209	0.0562
dan_aunz.BEBROAD_new.b37.DENTIST.	–	1.00	0.513	0.412(1416)	0.403(15626)	0.0262	0.0401
dan_biov.BE.DENTIST.	–	1.00	0.357	0.443(87)	0.409(61474)	0.142	0.154
dan_chop.BEBROAD.DENTIST.	–	1.01	0.11	0.406(368)	0.430(3568)	–0.129	0.0806
dan_esbb.BEBROAD.b37.noChrX.DENTIST.	–	1.00	0.459	0.463(727)	0.453(206468)	0.0392	0.0528
dan_fngn.BE.b37.noChrX.DENTIST.	–	1.00	0.0157	0.448(770)	0.418(301716)	0.126	0.0522
dan_ipsy.BE.DENTIST.	–	0.99	0.0147	0.447(466)	0.408(20439)	0.164	0.0673
dan_jans.BENARROW.b37.noChrX.DENTIST.	–	1.00	0.931	0.423(214)	0.422(902)	–0.00948	0.11
dan_moba.BEBROAD.DENTIST.	–	1.00	8.58e–09	0.425(24094)	0.411(150480)	0.0589	0.0102
dan_sebe.BEBROAD.noChrX.DENTIST.	–	0.99	0.46	0.433(562)	0.405(577)	0.067	0.0905
dan_sedk.BEBROAD.DENTIST.	–	1.01	0.0311	0.433(1757)	0.412(3793)	0.0899	0.0417
dan_ukb2.BEBROAD.DENTIST.	–	1.00	0.000132	0.460(652)	0.404(445105)	0.208	0.0545
dan_ukch.BEBROAD.b37.noChrX.DENTIST.	–	1.02	0.007	0.466(264)	0.400(1357)	0.259	0.096
dan_ukd2.BEBROAD.b37.noChrX.DENTIST.	–	0.99	0.000826	0.435(5951)	0.407(4174)	0.13	0.0388
dan_usa1.BEBROAD.DENTIST.	–	1.01	0.171	0.474(98)	0.414(750)	0.216	0.158
dan_usa2.BEBROAD.b37.DENTIST.	–	0.99	0.189	0.407(606)	0.428(1296)	–0.0947	0.0721
**dan_**	**–**	**1.00**	**2.97e–17**	**0.429(39279)**	**0.417(1227436)**	**0.0716**	**0.0085**

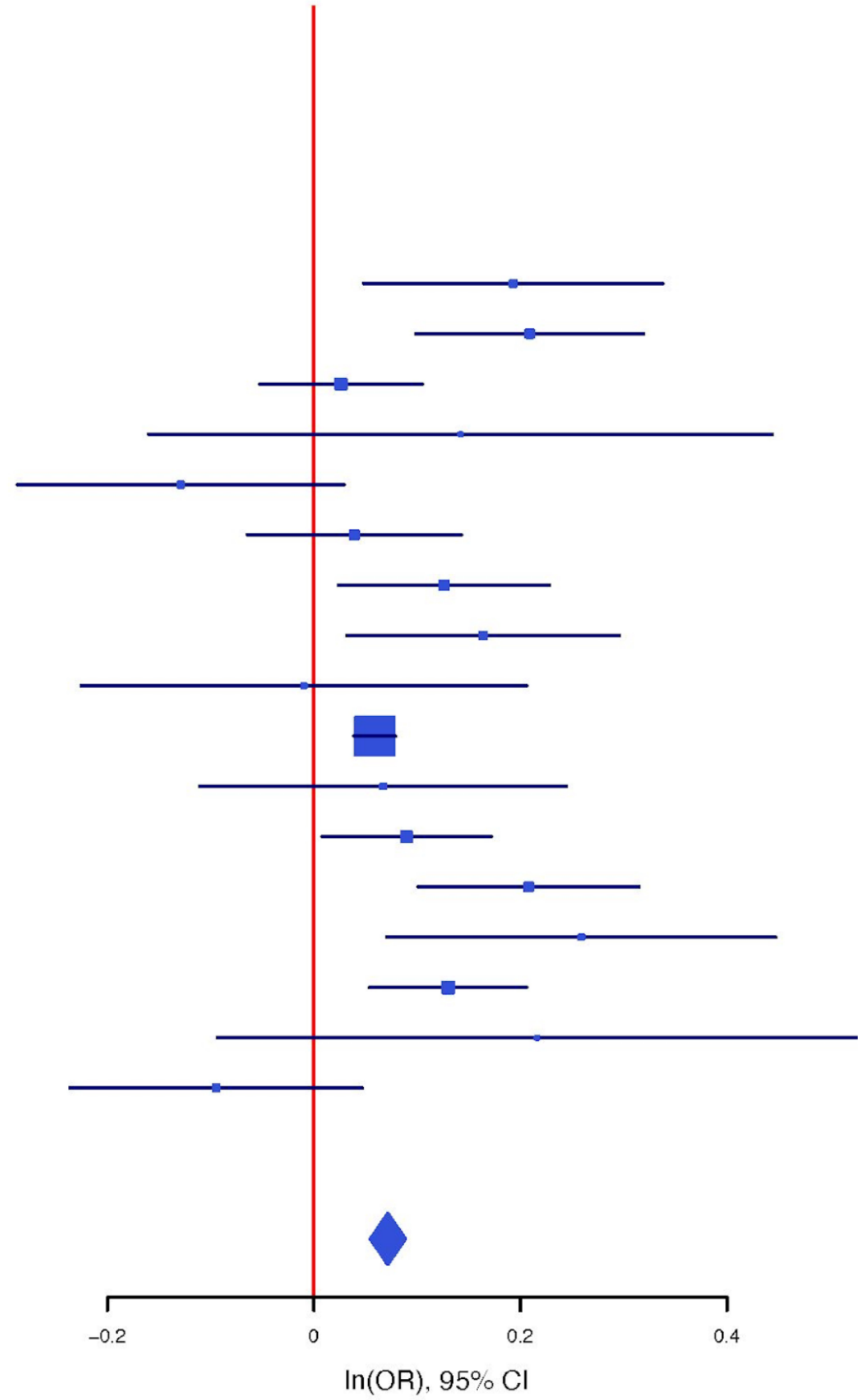

Supplementary Figure 6

rs66723169		A/C		18:57808978			
	++−−−++−++++++?−−			het_P:	0.277	het_I:	15.4
	**ngt**	**info**	**p_value**	**f_ca(n)**	**f_co(n)**	**ln(OR)**	**STDerr**
dan_agds.BEBROAD.DENTIST.	−	0.99	0.861	0.226(407)	0.223(6850)	0.0154	0.0884
dan_alsp.BEBROAD.DENTIST.	−	0.99	0.169	0.248(840)	0.224(2861)	0.0887	0.0644
dan_aunz.BEBROAD_new.b37.DENTIST.	−	1.00	0.348	0.224(1416)	0.231(15626)	−0.0444	0.0474
dan_biov.BE.DENTIST.	−	1.00	0.424	0.200(87)	0.227(61474)	−0.145	0.181
dan_chop.BEBROAD.DENTIST.	−	1.02	0.299	0.219(368)	0.236(3568)	−0.0992	0.0955
dan_esbb.BEBROAD.b37.noChrX.DENTIST.	−	1.00	0.0591	0.190(727)	0.172(206468)	0.132	0.0699
dan_fngn.BE.b37.noChrX.DENTIST.	−	1.00	0.128	0.190(770)	0.174(301716)	0.103	0.0676
dan_ipsy.BE.DENTIST.	−	0.98	0.353	0.232(466)	0.245(20439)	−0.0744	0.0801
dan_jans.BENARROW.b37.noChrX.DENTIST.	−	0.97	0.469	0.238(214)	0.215(902)	0.0957	0.132
dan_moba.BEBROAD.DENTIST.	−	0.99	8.47e−09	0.263(24094)	0.250(150480)	0.067	0.0116
dan_sebe.BEBROAD.noChrX.DENTIST.	−	0.99	0.181	0.252(562)	0.240(577)	0.138	0.103
dan_sedk.BEBROAD.DENTIST.	−	0.99	0.485	0.243(1757)	0.237(3793)	0.0342	0.049
dan_ukb2.BEBROAD.DENTIST.	−	0.99	0.0473	0.253(652)	0.230(445105)	0.126	0.0635
dan_ukch.BEBROAD.b37.noChrX.DENTIST.	−	1.03	0.556	0.236(264)	0.232(1357)	0.066	0.112
dan_usa1.BEBROAD.DENTIST.	−	1.03	0.555	0.224(98)	0.248(750)	−0.109	0.185
dan_usa2.BEBROAD.b37.DENTIST.	−	0.96	0.808	0.236(606)	0.241(1296)	−0.0206	0.0846
**dan_**	**−**	**0.99**	**5.02e−09**	**0.254(39279)**	**0.209(1227436)**	**0.0585**	**0.01**

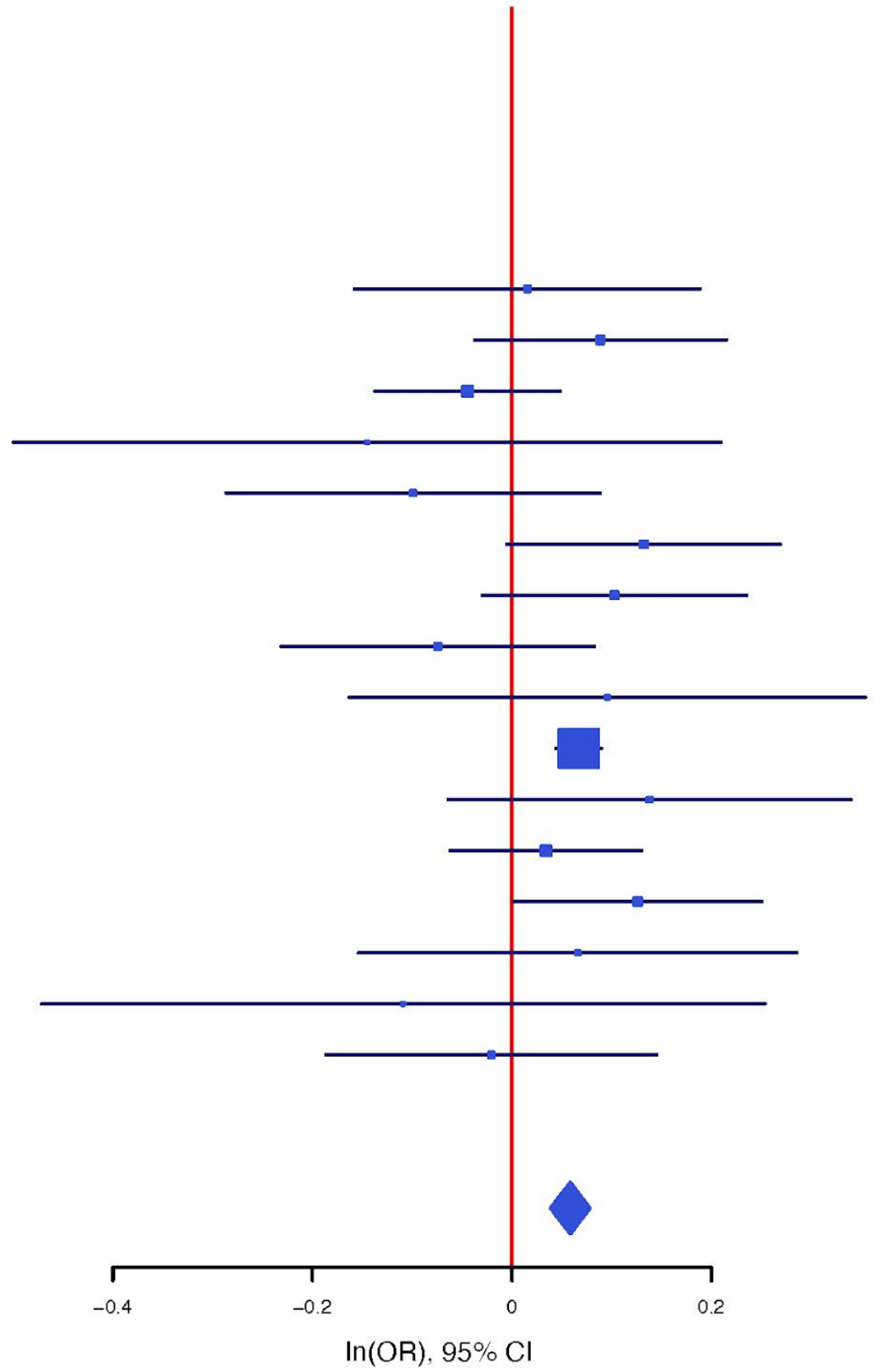

Supplementary Figures 7-14: Forest plots for the eight genome-wide significant associations with AN in order of position on the genome

Each row represents one cohort, with the final row showing the meta-analytic result. Initial information gives the variant name, effect and non-effect alleles, and variant position, followed by a summary of directions of effect, heterogeneity p-value, and heterogeneity I statistic. Main data columns include the imputation information metric (info), the association p-value, the frequency of the variant in cases (f_ca) and in controls (f_co), and the log odds ratio and standard error of the association, which are also visualised in the plot as the point (log odds ratio, with width proportional to N) and bars (95% confidence interval, 1.96 standard errors). The genotyping indicator (ngt) was set to missing for all cohorts and should be ignored.

Supplementary Figure 7

rs10747478		G/T		1:96901455			
+−+?++++−++++−+++++++−+++−				het_P:	0.557	het_I:	0.0
	ngt	info	p_value	f_ca(n)	f_co(n)	ln(OR)	STDerr
dan_agds.AN.DENTIST.	–	0.98	0.436	0.577(435)	0.591(6822)	−0.0567	0.0727
dan_alsp.AN.DENTIST.	–	1.00	0.898	0.407(216)	0.410(2861)	0.0131	0.101
dan_aunz.AN.DENTIST.	–	1.00	0.0204	0.578(2535)	0.598(15626)	−0.0716	0.0309
dan_chop.AN.DENTIST.	–	1.00	0.198	0.561(1032)	0.573(3568)	−0.0683	0.053
dan_esbb.AN.b37.noChrX.DENTIST.	–	1.00	0.142	0.545(526)	0.567(206468)	−0.0917	0.0624
dan_fin1.AN.DENTIST.	–	0.96	0.122	0.588(130)	0.633(503)	−0.24	0.155
dan_fngn.AN.b37.noChrX.DENTIST.	–	1.00	0.285	0.593(1489)	0.603(301716)	−0.0404	0.0377
dan_fre1.AN.DENTIST.	–	1.02	0.82	0.584(286)	0.571(990)	0.0226	0.0993
dan_gcfm.AN.b37.noChrX.DENTIST.	–	1.06	0.426	0.578(153)	0.616(159)	−0.131	0.164
dan_gns2.AN.DENTIST.	–	0.97	0.128	0.548(657)	0.571(2263)	−0.0994	0.0652
dan_ipsy.AN.DENTIST.	–	1.00	1.97e−05	0.565(4618)	0.585(20439)	−0.103	0.0242
dan_itgr.AN.DENTIST.	–	1.03	0.925	0.496(117)	0.500(129)	−0.0196	0.209
dan_moba.AN.DENTIST.	–	1.00	0.564	0.583(1252)	0.578(150480)	0.0237	0.041
dan_net1.AN.DENTIST.	–	0.96	0.46	0.570(258)	0.579(1337)	−0.075	0.102
dan_net2.AN.b37.noChrX.DENTIST.	–	0.90	0.102	0.579(325)	0.622(293)	−0.205	0.125
dan_poco.AN.DENTIST.	–	1.00	0.769	0.556(239)	0.551(604)	−0.0342	0.117
dan_sebe.AN.noChrX.DENTIST.	–	0.96	0.0291	0.514(249)	0.561(577)	−0.251	0.115
dan_sedk.AN.DENTIST.	–	1.02	0.139	0.569(4105)	0.578(3793)	−0.0483	0.0326
dan_spa1.AN.DENTIST.	–	0.96	0.322	0.582(183)	0.611(157)	−0.185	0.187
dan_ukb2.AN.DENTIST.	–	1.00	0.261	0.579(1351)	0.590(455105)	−0.0441	0.0393
dan_ukch.AN.b37.noChrX.DENTIST.	–	1.00	0.23	0.618(416)	0.593(1357)	0.0985	0.082
dan_ukd1.AN.DENTIST.	–	1.02	0.0917	0.581(242)	0.625(957)	−0.177	0.105
dan_ukd2.AN.b37.noChrX.DENTIST.	–	1.00	0.134	0.572(1559)	0.597(4174)	−0.0835	0.0558
dan_usa1.AN.DENTIST.	–	0.94	0.46	0.568(417)	0.589(750)	−0.0691	0.0936
dan_usa2.AN.b37.DENTIST.	–	0.97	0.987	0.579(1315)	0.579(1296)	0.000949	0.0577
**dan_**	**–**	**1.00**	**1.43e−08**	**0.570(24223)**	**0.587(1243971)**	**−0.0623**	**0.011**

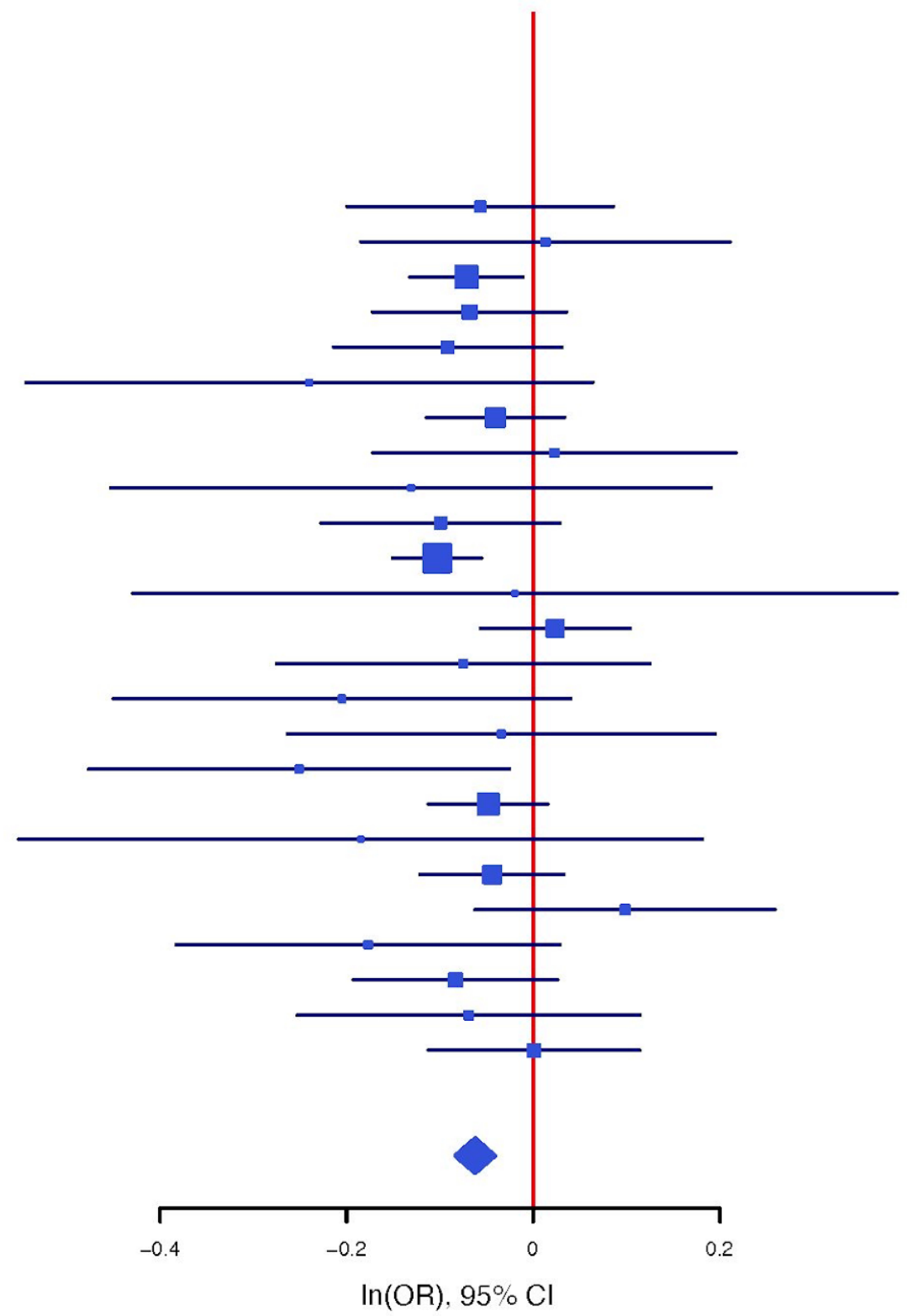

Supplementary Figure 8

rs113519699		C/A		3:49071450			
−+−−−−−?+−−−−−−−−−+−−−−−−−−				het_P:	0.948	het_I:	0.0
	ngt	info	p_value	f_ca(n)	f_co(n)	ln(OR)	STDerr
dan_agds.AN.DENTIST.	–	0.99	0.0177	0.111(435)	0.087(6822)	0.295	0.124
dan_alsp.AN.DENTIST.	–	0.98	0.964	0.095(216)	0.095(2861)	−0.00773	0.17
dan_aunz.AN.DENTIST.	–	1.01	8.42e−06	0.107(2535)	0.089(15626)	0.222	0.0498
dan_biov.AN.DENTIST.	–	1.00	0.937	0.093(118)	0.093(61547)	0.0177	0.225
dan_chop.AN.DENTIST.	–	1.00	0.413	0.098(1032)	0.089(3568)	0.0723	0.0883
dan_esbb.AN.b37.noChrX.DENTIST.	–	1.00	0.034	0.091(526)	0.074(206468)	0.233	0.11
dan_fin1.AN.DENTIST.	–	1.01	0.826	0.092(130)	0.091(503)	0.0562	0.256
dan_fre1.AN.DENTIST.	–	0.97	0.894	0.110(286)	0.111(990)	−0.0212	0.159
dan_gcfm.AN.b37.noChrX.DENTIST.	–	1.11	0.869	0.118(153)	0.113(159)	0.0407	0.247
dan_gns2.AN.DENTIST.	–	1.04	0.0773	0.109(657)	0.091(2263)	0.182	0.103
dan_ipsy.AN.DENTIST.	–	0.98	7.97e−06	0.105(4618)	0.089(20439)	0.18	0.0403
dan_itgr.AN.DENTIST.	–	1.10	0.904	0.060(117)	0.050(129)	0.0513	0.427
dan_moba.AN.DENTIST.	–	0.99	0.254	0.090(1252)	0.083(150480)	0.0832	0.0729
dan_net1.AN.DENTIST.	–	1.02	0.708	0.109(258)	0.101(1337)	0.0596	0.159
dan_net2.AN.b37.noChrX.DENTIST.	–	0.94	0.59	0.092(325)	0.083(293)	0.115	0.213
dan_poco.AN.DENTIST.	–	0.96	0.684	0.077(239)	0.067(604)	0.0924	0.227
dan_sebe.AN.noChrX.DENTIST.	–	0.98	0.661	0.083(249)	0.093(577)	−0.0873	0.199
dan_sedk.AN.DENTIST.	–	0.99	0.0264	0.097(4105)	0.089(3793)	0.125	0.0563
dan_spa1.AN.DENTIST.	–	1.07	0.354	0.104(183)	0.083(157)	0.271	0.292
dan_ukb2.AN.DENTIST.	–	1.00	0.00573	0.109(1351)	0.093(455105)	0.172	0.0621
dan_ukch.AN.b37.noChrX.DENTIST.	–	0.99	0.128	0.101(416)	0.087(1357)	0.206	0.135
dan_ukd1.AN.DENTIST.	–	1.00	0.0803	0.103(242)	0.077(957)	0.311	0.178
dan_ukd2.AN.b37.noChrX.DENTIST.	–	1.00	0.00199	0.111(1559)	0.096(4174)	0.277	0.0898
dan_usa1.AN.DENTIST.	–	1.02	0.191	0.104(417)	0.087(750)	0.194	0.149
dan_usa2.AN.b37.DENTIST.	–	1.02	0.046	0.097(1315)	0.081(1296)	0.196	0.0984
**dan_**	**–**	**1.00**	**2.86e−18**	**0.102(24223)**	**0.087(1243971)**	**0.166**	**0.019**

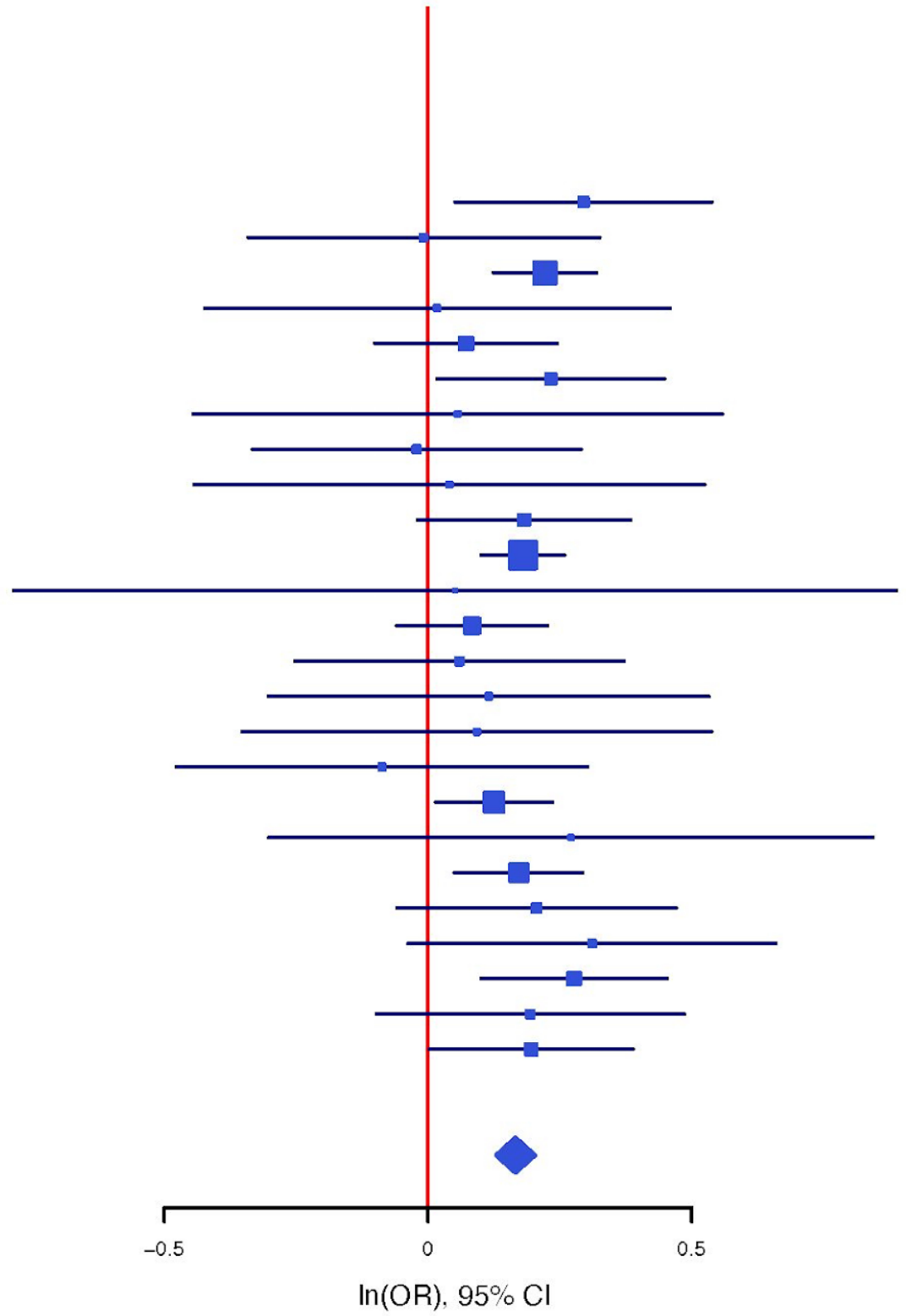

Supplementary Figure 9

rs9310201		T/A		3:70991842			
		---?--+-++------+-------+--+		het_P:	0.0441	het_I:	35.1
	**ngt**	**info**	**p_value**	**f_ca(n)**	**f_co(n)**	**ln(OR)**	**STDerr**
dan_agds.AN.DENTIST.	–	0.94	0.429	0.428(435)	0.414(6822)	0.0585	0.0739
dan_alsp.AN.DENTIST.	–	0.99	0.144	0.461(216)	0.426(2861)	0.144	0.0988
dan_aunz.AN.DENTIST.	–	0.99	0.072	0.432(2535)	0.418(15626)	0.0556	0.0309
dan_chop.AN.DENTIST.	–	0.99	5.65e–05	0.467(1032)	0.418(3568)	0.212	0.0528
dan_esbb.AN.b37.noChrX.DENTIST.	–	0.96	0.0103	0.485(526)	0.446(206468)	0.16	0.0625
dan_fin1.AN.DENTIST.	–	1.06	0.23	0.358(130)	0.391(503)	–0.179	0.149
dan_fngn.AN.b37.noChrX.DENTIST.	–	0.99	0.585	0.371(1489)	0.370(301716)	0.021	0.0384
dan_fre1.AN.DENTIST.	–	0.99	0.717	0.425(286)	0.426(990)	–0.0363	0.1
dan_gcfm.AN.b37.noChrX.DENTIST.	–	0.88	0.651	0.432(153)	0.431(159)	–0.0813	0.18
dan_gns2.AN.DENTIST.	–	1.00	0.323	0.437(657)	0.423(2263)	0.0643	0.065
dan_ipsy.AN.DENTIST.	–	0.96	0.000988	0.428(4618)	0.411(20439)	0.0817	0.0248
dan_itgr.AN.DENTIST.	–	1.01	0.819	0.474(117)	0.484(129)	0.0496	0.217
dan_moba.AN.DENTIST.	–	0.95	0.0807	0.422(1252)	0.407(150480)	0.073	0.0418
dan_net1.AN.DENTIST.	–	1.03	0.0455	0.479(258)	0.432(1337)	0.196	0.0978
dan_net2.AN.b37.noChrX.DENTIST.	–	0.91	0.715	0.427(325)	0.434(293)	–0.0452	0.124
dan_poco.AN.DENTIST.	–	1.03	0.0224	0.487(239)	0.425(604)	0.266	0.116
dan_sebe.AN.noChrX.DENTIST.	–	0.91	0.00222	0.449(249)	0.379(577)	0.369	0.121
dan_sedk.AN.DENTIST.	–	1.00	0.203	0.399(4105)	0.384(3793)	0.0453	0.0355
dan_spa1.AN.DENTIST.	–	1.07	0.606	0.454(183)	0.408(157)	0.0849	0.165
dan_ukb2.AN.DENTIST.	–	1.00	0.00557	0.442(1351)	0.415(455105)	0.106	0.0382
dan_ukch.AN.b37.noChrX.DENTIST.	–	0.97	0.0647	0.446(416)	0.407(1357)	0.151	0.082
dan_ukd1.AN.DENTIST.	–	1.01	0.224	0.411(242)	0.443(957)	–0.128	0.105
dan_ukd2.AN.b37.noChrX.DENTIST.	–	0.98	0.152	0.435(1559)	0.415(4174)	0.0807	0.0564
dan_usa1.AN.DENTIST.	–	1.00	0.0326	0.459(417)	0.414(750)	0.192	0.0897
dan_usa2.AN.b37.DENTIST.	–	0.96	0.988	0.431(1315)	0.431(1296)	–0.00089	0.0581
**dan_**	**–**	**0.98**	**3.12e–12**	**0.427(24223)**	**0.408(1243971)**	**0.0777**	**0.0111**

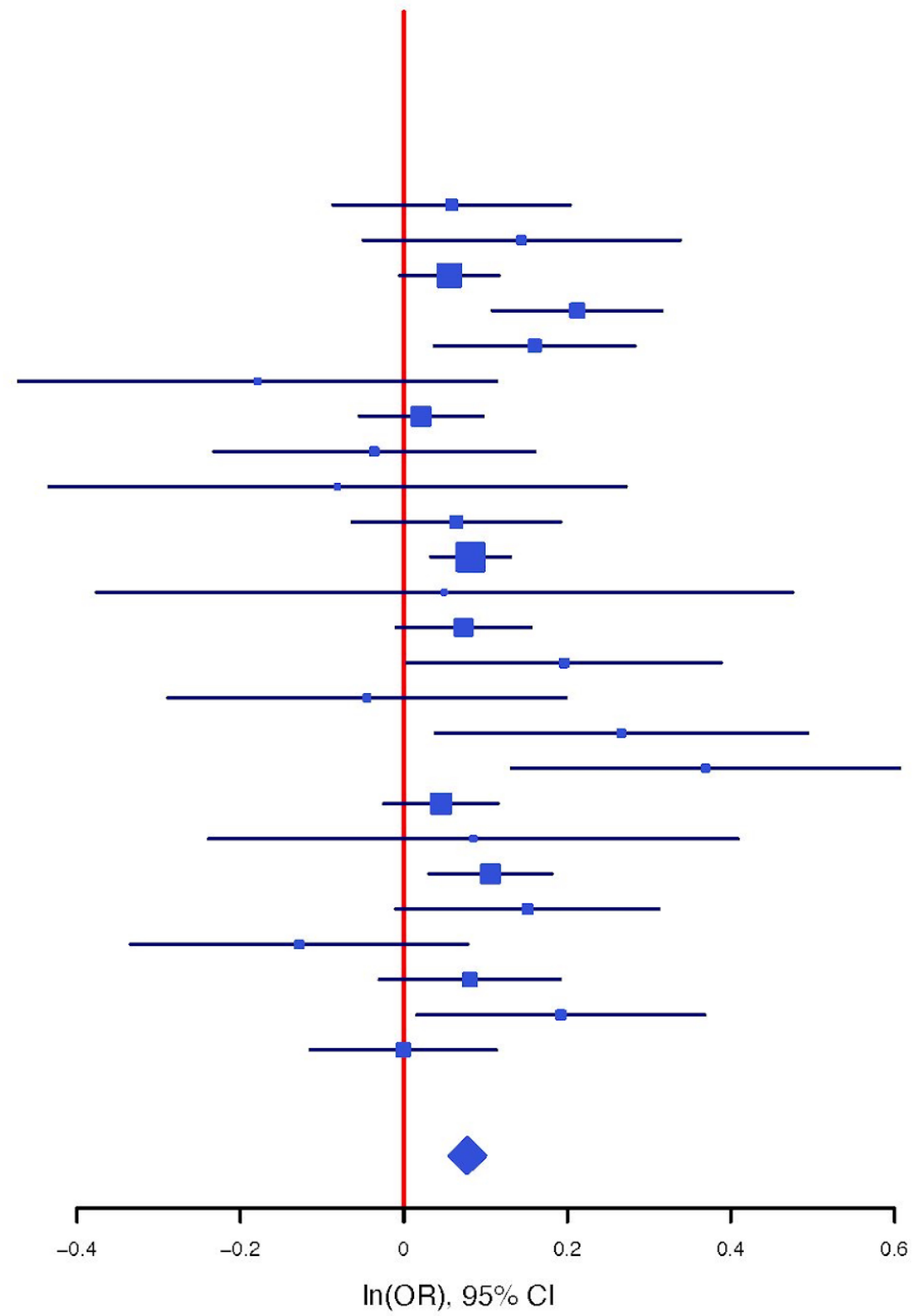

Supplementary Figure 10

rs6872919		C/A		5:25086440			
----------+--------+-+------+				het_P:	0.213	het_I:	17.5
	**ngt**	**info**	**p_value**	**f_ca(n)**	**f_co(n)**	**ln(OR)**	**STDerr**
dan_agds.AN.DENTIST.	–	1.00	0.273	0.570(435)	0.550(6822)	0.0775	0.0707
dan_alsp.AN.DENTIST.	–	1.00	0.223	0.407(216)	0.439(2861)	0.123	0.101
dan_aunz.AN.DENTIST.	–	1.01	0.117	0.574(2535)	0.558(15626)	0.0481	0.0307
dan_biov.AN.DENTIST.	–	1.00	0.0914	0.619(118)	0.559(61547)	0.223	0.132
dan_chop.AN.DENTIST.	–	0.99	0.000483	0.604(1032)	0.560(3568)	0.188	0.054
dan_esbb.AN.b37.noChrX.DENTIST.	–	1.00	0.589	0.496(526)	0.489(206468)	0.0334	0.0619
dan_fin1.AN.DENTIST.	–	0.99	0.411	0.531(130)	0.496(503)	0.123	0.15
dan_fngn.AN.b37.noChrX.DENTIST.	–	1.00	0.961	0.503(1489)	0.506(301716)	0.00183	0.037
dan_fre1.AN.DENTIST.	–	1.02	0.471	0.615(286)	0.586(990)	0.0723	0.1
dan_gcfm.AN.b37.noChrX.DENTIST.	–	0.98	0.901	0.611(153)	0.610(159)	−0.0213	0.171
dan_gns2.AN.DENTIST.	–	1.00	0.071	0.590(657)	0.563(2263)	0.118	0.0652
dan_ipsy.AN.DENTIST.	–	0.99	0.00324	0.588(4618)	0.571(20439)	0.0721	0.0245
dan_itgr.AN.DENTIST.	–	0.97	0.521	0.603(117)	0.632(129)	0.149	0.232
dan_moba.AN.DENTIST.	–	1.00	0.906	0.553(1252)	0.552(150480)	0.00481	0.0408
dan_net1.AN.DENTIST.	–	1.04	0.591	0.597(258)	0.579(1337)	0.0529	0.0984
dan_net2.AN.b37.noChrX.DENTIST.	–	1.06	0.0587	0.602(325)	0.551(293)	0.218	0.115
dan_poco.AN.DENTIST.	–	0.96	0.217	0.542(239)	0.514(604)	0.145	0.117
dan_sebe.AN.noChrX.DENTIST.	–	1.02	0.281	0.530(249)	0.556(577)	−0.121	0.112
dan_sedk.AN.DENTIST.	–	1.01	0.0622	0.559(4105)	0.546(3793)	0.0605	0.0324
dan_spa1.AN.DENTIST.	–	0.95	0.411	0.609(183)	0.653(157)	−0.15	0.183
dan_ukb2.AN.DENTIST.	–	1.00	0.0431	0.576(1351)	0.555(455105)	0.0775	0.0383
dan_ukch.AN.b37.noChrX.DENTIST.	–	0.98	0.189	0.578(416)	0.551(1357)	0.107	0.0816
dan_ukd1.AN.DENTIST.	–	1.04	0.0591	0.579(242)	0.533(957)	0.196	0.104
dan_ukd2.AN.b37.noChrX.DENTIST.	–	0.97	0.000323	0.583(1559)	0.545(4174)	0.205	0.057
dan_usa1.AN.DENTIST.	–	0.97	0.541	0.588(417)	0.568(750)	0.056	0.0916
dan_usa2.AN.b37.DENTIST.	–	0.96	0.769	0.584(1315)	0.590(1296)	−0.0172	0.0585
**dan_**	**–**	**1.00**	**1.07e−09**	**0.570(24223)**	**0.532(1243971)**	**0.0665**	**0.0109**

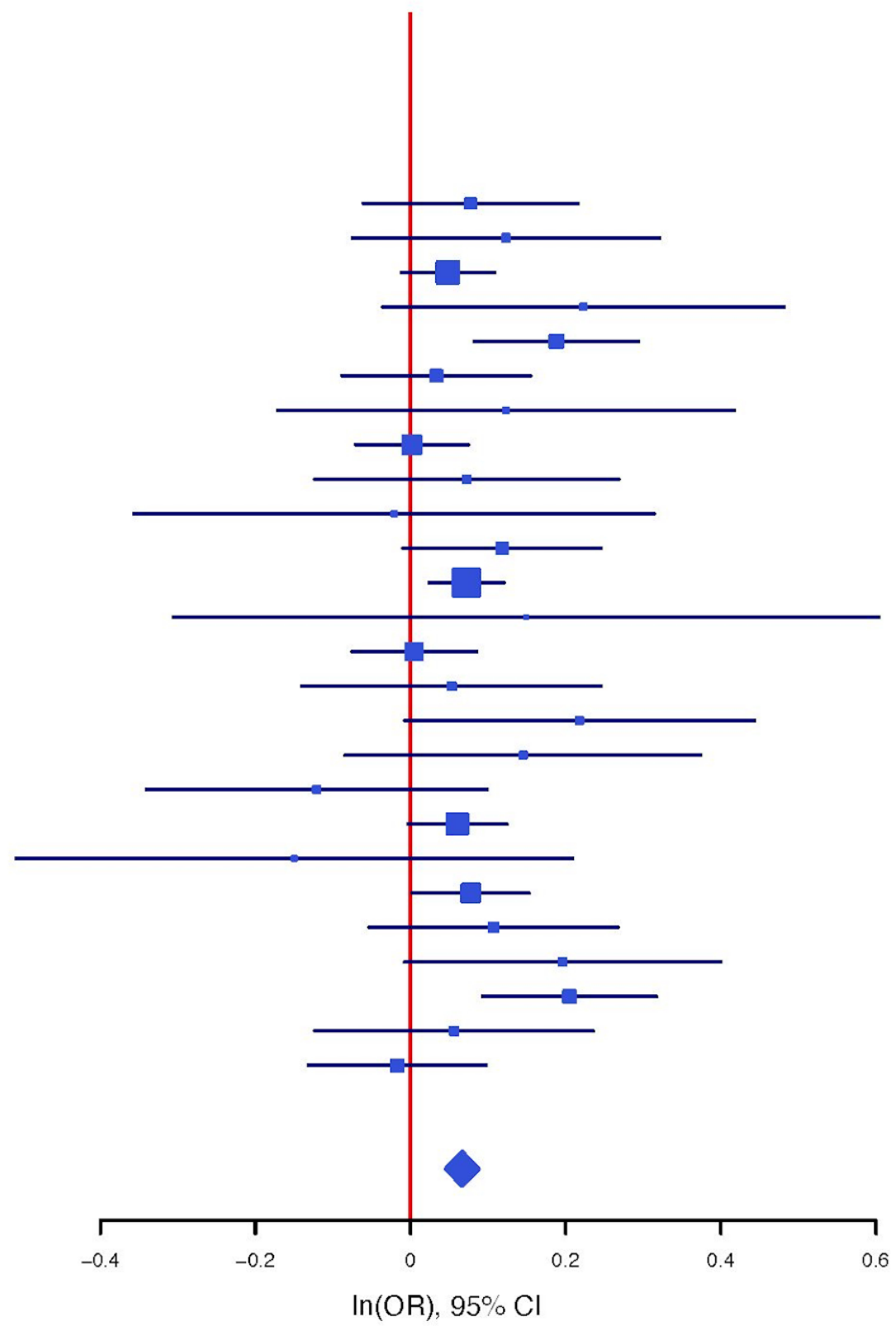

Supplementary Figure 11

rs10740439		T/C		10:76307972			
		-----+------------+-+---?--		het_P:	0.234	het_I:	16.2
	ngt	info	p_value	f_ca(n)	f_co(n)	ln(OR)	STDerr
dan_agds.AN.DENTIST.	–	0.94	0.456	0.722(435)	0.733(6822)	−0.0617	0.0827
dan_alsp.AN.DENTIST.	–	1.00	0.719	0.266(216)	0.260(2861)	−0.0409	0.114
dan_aunz.AN.DENTIST.	–	1.01	0.00223	0.734(2535)	0.750(15626)	−0.106	0.0345
dan_biov.AN.DENTIST.	–	0.96	0.12	0.704(118)	0.748(61547)	−0.234	0.151
dan_chop.AN.DENTIST.	–	0.98	0.0986	0.746(1032)	0.768(3568)	−0.101	0.0614
dan_esbb.AN.b37.noChrX.DENTIST.	–	1.00	0.128	0.758(526)	0.736(206468)	0.107	0.0702
dan_fin1.AN.DENTIST.	–	0.96	0.609	0.654(130)	0.659(503)	−0.0804	0.157
dan_fngn.AN.b37.noChrX.DENTIST.	–	0.99	0.168	0.661(1489)	0.675(301716)	−0.0543	0.0394
dan_fre1.AN.DENTIST.	–	0.98	0.789	0.787(286)	0.780(990)	−0.0326	0.122
dan_gcfm.AN.b37.noChrX.DENTIST.	–	1.01	0.0568	0.755(153)	0.807(159)	−0.39	0.205
dan_gns2.AN.DENTIST.	–	1.01	0.0326	0.728(657)	0.761(2263)	−0.154	0.0719
dan_ipsy.AN.DENTIST.	–	0.99	0.000304	0.715(4618)	0.730(20439)	−0.0972	0.0269
dan_itgr.AN.DENTIST.	–	1.08	0.626	0.782(117)	0.798(129)	−0.129	0.265
dan_moba.AN.DENTIST.	–	1.00	0.000958	0.655(1252)	0.685(150480)	−0.144	0.0437
dan_net1.AN.DENTIST.	–	1.00	0.673	0.731(258)	0.743(1337)	−0.0467	0.111
dan_net2.AN.b37.noChrX.DENTIST.	–	0.97	0.0299	0.687(325)	0.738(293)	−0.285	0.131
dan_poco.AN.DENTIST.	–	0.98	0.186	0.732(239)	0.748(604)	−0.175	0.133
dan_sebe.AN.noChrX.DENTIST.	–	0.98	0.344	0.720(249)	0.708(577)	0.118	0.125
dan_sedk.AN.DENTIST.	–	1.01	0.621	0.697(4105)	0.702(3793)	−0.0175	0.0354
dan_spa1.AN.DENTIST.	–	0.90	0.361	0.806(183)	0.774(157)	0.203	0.222
dan_ukb2.AN.DENTIST.	–	1.00	0.575	0.737(1351)	0.741(455105)	−0.0248	0.0442
dan_ukch.AN.b37.noChrX.DENTIST.	–	0.97	0.97	0.727(416)	0.727(1357)	−0.00343	0.0909
dan_ukd1.AN.DENTIST.	–	0.99	0.145	0.698(242)	0.734(957)	−0.166	0.114
dan_usa1.AN.DENTIST.	–	1.08	0.325	0.741(417)	0.751(750)	−0.0973	0.0988
dan_usa2.AN.b37.DENTIST.	–	1.01	0.444	0.743(1315)	0.755(1296)	−0.0496	0.0649
**dan_**	**–**	**0.99**	**1.9e−09**	**0.711(24223)**	**0.716(1243971)**	**−0.074**	**0.0123**

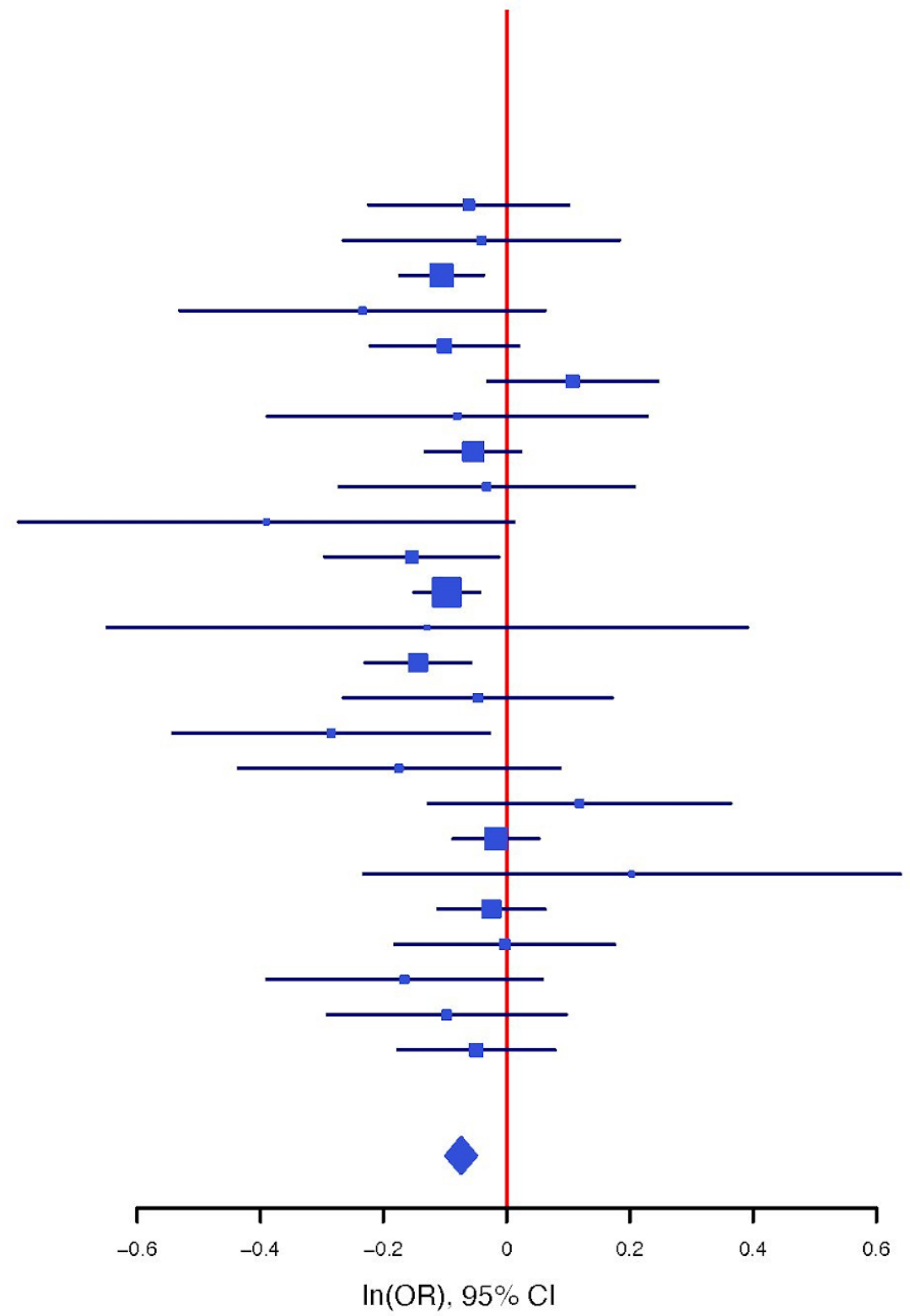

Supplementary Figure 12

rs12762024 **C/G** **10:131441055**

−++??+++++++−+−++++++++−+− het_P: 0.453 het_I: 0.6

	ngt	info	p_value	f_ca(n)	f_co(n)	ln(OR)	STDerr
dan_agds.AN.DENTIST.	–	0.84	0.773	0.410(435)	0.415(6822)	−0.021	0.073
dan_alsp.AN.DENTIST.	–	1.00	0.0456	0.477(216)	0.435(2861)	0.201	0.1
dan_aunz.AN.DENTIST.	–	1.00	0.013	0.444(2535)	0.426(15626)	0.0762	0.0307
dan_esbb.AN.b37.noChrX.DENTIST.	–	1.00	0.0421	0.494(526)	0.464(206468)	0.125	0.0615
dan_fin1.AN.DENTIST.	–	1.04	0.261	0.546(130)	0.498(503)	0.163	0.145
dan_fngn.AN.b37.noChrX.DENTIST.	–	1.00	0.063	0.525(1489)	0.506(301716)	0.0688	0.037
dan_fre1.AN.DENTIST.	–	0.97	0.0126	0.474(286)	0.418(990)	0.25	0.1
dan_gcfm.AN.b37.noChrX.DENTIST.	–	0.93	0.992	0.400(153)	0.409(159)	0.00179	0.177
dan_gns2.AN.DENTIST.	–	0.99	0.0195	0.441(657)	0.410(2263)	0.152	0.065
dan_ipsy.AN.DENTIST.	–	0.99	0.003	0.439(4618)	0.421(20439)	0.0721	0.0243
dan_itgr.AN.DENTIST.	–	1.07	0.0778	0.355(117)	0.446(129)	−0.386	0.219
dan_moba.AN.DENTIST.	–	1.00	0.0483	0.443(1252)	0.424(150480)	0.0809	0.041
dan_net1.AN.DENTIST.	–	0.98	0.887	0.405(258)	0.404(1337)	−0.0145	0.101
dan_net2.AN.b37.noChrX.DENTIST.	–	0.97	0.277	0.436(325)	0.402(293)	0.13	0.12
dan_poco.AN.DENTIST.	–	1.02	0.904	0.416(239)	0.432(604)	0.014	0.116
dan_sebe.AN.noChrX.DENTIST.	–	0.97	0.568	0.444(249)	0.441(577)	0.0664	0.116
dan_sedk.AN.DENTIST.	–	0.99	0.0492	0.445(4105)	0.430(3793)	0.0657	0.0334
dan_spa1.AN.DENTIST.	–	0.91	0.899	0.426(183)	0.436(157)	0.0229	0.18
dan_ukb2.AN.DENTIST.	–	1.00	0.322	0.440(1351)	0.431(455105)	0.0388	0.0392
dan_ukch.AN.b37.noChrX.DENTIST.	–	1.00	0.346	0.449(416)	0.432(1357)	0.0758	0.0804
dan_ukd1.AN.DENTIST.	–	1.03	0.421	0.448(242)	0.426(957)	0.0832	0.103
dan_ukd2.AN.b37.noChrX.DENTIST.	–	0.98	0.333	0.423(1559)	0.430(4174)	−0.0544	0.0562
dan_usa1.AN.DENTIST.	–	1.00	0.103	0.463(417)	0.429(750)	0.145	0.0892
dan_usa2.AN.b37.DENTIST.	–	0.96	0.886	0.439(1315)	0.440(1296)	−0.00829	0.0578
**dan_**	**–**	**0.99**	**3.28e−09**	**0.447(24223)**	**0.455(1243971)**	**0.0663**	**0.0112**

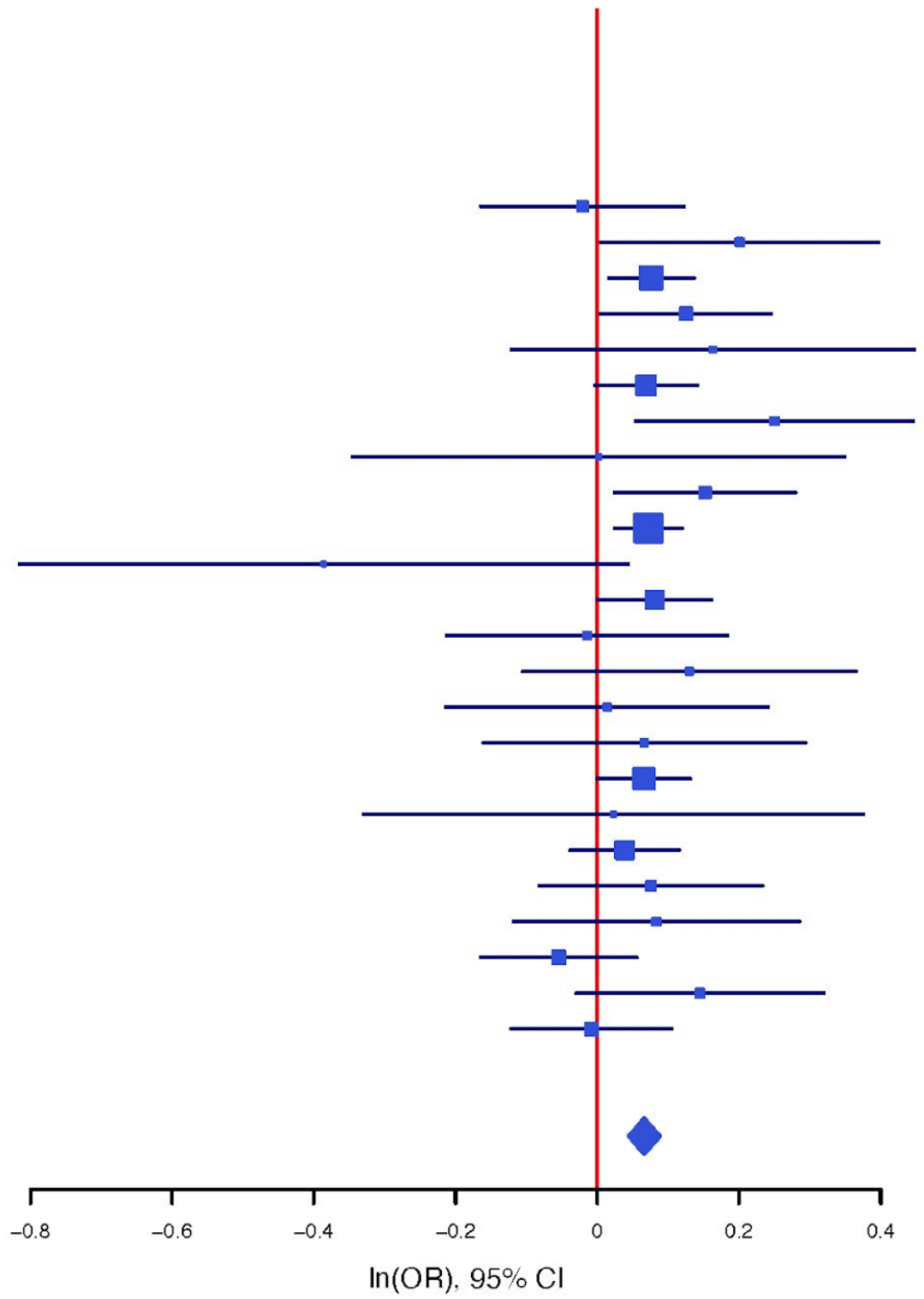

Supplementary Figure 13

	ngt	info	p_value	f_ca(n)	f_co(n)	ln(OR)	STDerr
**rs6589488**		**T/A**		**11:115096956**			
	+−++++−++−++−−++++++++−+?++			het_P:	0.295	het_I:	11.8
dan_agds.AN.DENTIST.	–	0.95	0.739	0.851(435)	0.855(6822)	−0.0347	0.104
dan_alsp.AN.DENTIST.	–	0.99	0.488	0.124(216)	0.141(2861)	0.106	0.153
dan_aunz.AN.DENTIST.	–	0.99	0.000731	0.842(2535)	0.861(15626)	−0.143	0.0423
dan_biov.AN.DENTIST.	–	1.00	0.0255	0.805(118)	0.856(61547)	−0.415	0.186
dan_chop.AN.DENTIST.	–	0.97	0.24	0.837(1032)	0.847(3568)	−0.0849	0.0722
dan_esbb.AN.b37.noChrX.DENTIST.	–	1.00	0.223	0.857(526)	0.870(206468)	−0.112	0.0918
dan_fin1.AN.DENTIST.	–	0.94	0.869	0.923(130)	0.921(503)	0.0464	0.281
dan_fngn.AN.b37.noChrX.DENTIST.	–	0.98	0.287	0.918(1489)	0.922(301716)	−0.0744	0.0699
dan_fre1.AN.DENTIST.	–	1.00	0.0239	0.811(286)	0.852(990)	−0.293	0.13
dan_gcfm.AN.b37.noChrX.DENTIST.	–	0.97	0.264	0.861(153)	0.834(159)	0.262	0.235
dan_gns2.AN.DENTIST.	–	0.96	0.14	0.837(657)	0.850(2263)	−0.131	0.0891
dan_ipsy.AN.DENTIST.	–	0.98	8.88e−06	0.840(4618)	0.858(20439)	−0.148	0.0334
dan_itgr.AN.DENTIST.	–	0.91	0.269	0.838(117)	0.822(129)	0.329	0.297
dan_moba.AN.DENTIST.	–	0.99	0.567	0.870(1252)	0.866(150480)	0.034	0.0594
dan_net1.AN.DENTIST.	–	1.00	0.358	0.841(258)	0.847(1337)	−0.125	0.136
dan_net2.AN.b37.noChrX.DENTIST.	–	1.01	0.14	0.832(325)	0.862(293)	−0.242	0.164
dan_poco.AN.DENTIST.	–	0.97	0.843	0.808(239)	0.810(604)	−0.0292	0.147
dan_sebe.AN.noChrX.DENTIST.	–	1.00	0.178	0.857(249)	0.879(577)	−0.229	0.17
dan_sedk.AN.DENTIST.	–	1.01	0.131	0.862(4105)	0.874(3793)	−0.0724	0.0479
dan_spa1.AN.DENTIST.	–	0.98	0.433	0.822(183)	0.834(157)	−0.178	0.226
dan_ukb2.AN.DENTIST.	–	0.99	0.348	0.853(1351)	0.859(455105)	−0.0523	0.0558
dan_ukch.AN.b37.noChrX.DENTIST.	–	0.93	0.287	0.858(416)	0.844(1357)	0.125	0.118
dan_ukd1.AN.DENTIST.	–	0.98	0.408	0.843(242)	0.857(957)	−0.121	0.146
dan_usa1.AN.DENTIST.	–	0.98	0.211	0.837(417)	0.857(750)	−0.156	0.125
dan_usa2.AN.b37.DENTIST.	–	0.99	0.193	0.847(1315)	0.858(1296)	−0.104	0.0797
**dan_**	**–**	**0.99**	**9.8e−10**	**0.846(24223)**	**0.875(1243971)**	**−0.0975**	**0.0159**

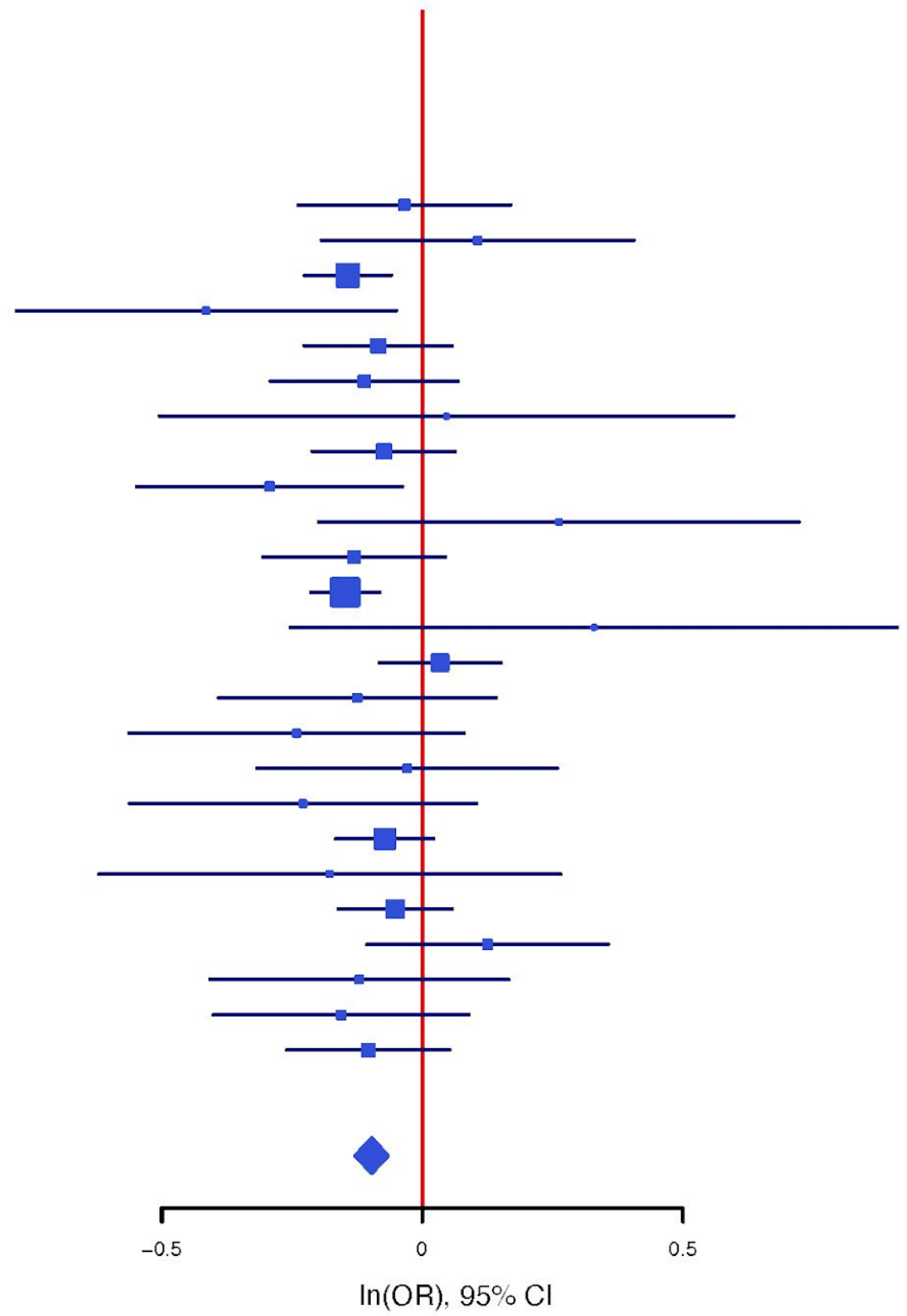

Supplementary Figure 14

rs12817084		C/T		12:17763314			
------+--+------++-+--+---				het_P:	0.415	het_I:	3.4
	ngt	info	p_value	f_ca(n)	f_co(n)	ln(OR)	STDerr
dan_agds.AN.DENTIST.	–	1.00	0.0499	0.125(435)	0.104(6822)	0.226	0.115
dan_alsp.AN.DENTIST.	–	0.99	0.125	0.124(216)	0.098(2861)	0.234	0.152
dan_aunz.AN.DENTIST.	–	1.00	0.627	0.112(2535)	0.109(15626)	0.0235	0.0484
dan_biov.AN.DENTIST.	–	1.00	0.63	0.114(118)	0.106(61547)	0.103	0.213
dan_chop.AN.DENTIST.	–	0.99	0.785	0.108(1032)	0.114(3568)	0.0227	0.0835
dan_esbb.AN.b37.noChrX.DENTIST.	–	1.00	0.02	0.139(526)	0.115(206468)	0.212	0.0911
dan_fin1.AN.DENTIST.	–	1.02	0.159	0.108(130)	0.149(503)	−0.318	0.225
dan_fngn.AN.b37.noChrX.DENTIST.	–	0.99	0.34	0.150(1489)	0.144(301716)	0.0505	0.0529
dan_fre1.AN.DENTIST.	–	0.97	0.494	0.121(286)	0.106(990)	0.107	0.156
dan_gcfm.AN.b37.noChrX.DENTIST.	–	0.98	0.518	0.095(153)	0.123(159)	−0.177	0.274
dan_gns2.AN.DENTIST.	–	0.98	0.0511	0.103(657)	0.085(2263)	0.212	0.109
dan_ipsy.AN.DENTIST.	–	0.95	0.00737	0.107(4618)	0.099(20439)	0.108	0.0402
dan_itgr.AN.DENTIST.	–	0.93	0.409	0.132(117)	0.116(129)	0.29	0.351
dan_moba.AN.DENTIST.	–	0.98	0.0137	0.125(1252)	0.108(150480)	0.16	0.0649
dan_net1.AN.DENTIST.	–	1.02	0.0615	0.134(258)	0.100(1337)	0.276	0.148
dan_net2.AN.b37.noChrX.DENTIST.	–	1.03	0.559	0.115(325)	0.102(293)	0.108	0.185
dan_poco.AN.DENTIST.	–	0.99	0.976	0.100(239)	0.094(604)	−0.00588	0.196
dan_sebe.AN.noChrX.DENTIST.	–	1.06	0.618	0.112(249)	0.108(577)	−0.0856	0.171
dan_sedk.AN.DENTIST.	–	1.00	0.12	0.110(4105)	0.103(3793)	0.0815	0.0524
dan_spa1.AN.DENTIST.	–	1.01	0.701	0.093(183)	0.086(157)	−0.114	0.297
dan_ukb2.AN.DENTIST.	–	0.99	0.533	0.110(1351)	0.107(455105)	0.0393	0.0631
dan_ukch.AN.b37.noChrX.DENTIST.	–	0.96	0.0157	0.123(416)	0.096(1357)	0.309	0.128
dan_ukd1.AN.DENTIST.	–	1.00	0.664	0.093(242)	0.098(957)	−0.0776	0.179
dan_ukd2.AN.b37.noChrX.DENTIST.	–	1.00	0.00748	0.123(1559)	0.103(4174)	0.232	0.0867
dan_usa1.AN.DENTIST.	–	1.01	0.124	0.112(417)	0.095(750)	0.222	0.144
dan_usa2.AN.b37.DENTIST.	–	0.97	0.102	0.111(1315)	0.100(1296)	0.152	0.093
**dan_**	**–**	**0.99**	**8.93e−09**	**0.115(24223)**	**0.117(1243971)**	**0.0991**	**0.0172**

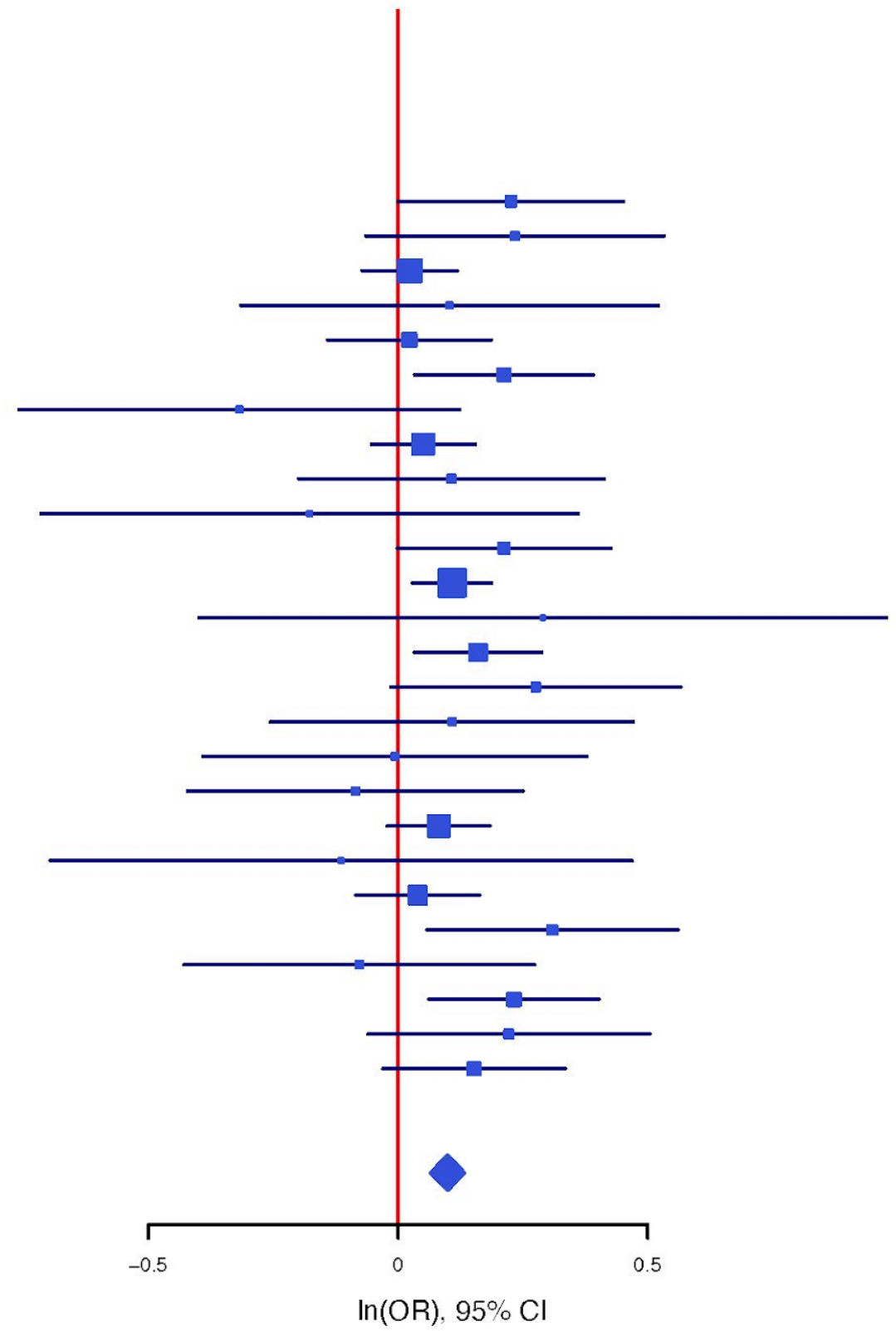

Supplementary Figure 15: Manhattan plots

Manhattan plots showing genome-wide association study results for each phenotype. The dotted red line is the genome-wide significance threshold ($P \leq 5x10^{-8}$). We performed association analyses using two-sided logistic regression (PLINK2, SAIGE, or REGENIE depending on study characteristics) followed by fixed-effects meta-analysis.

a) BE-BROAD (39,279 cases, 1,227,436 controls)

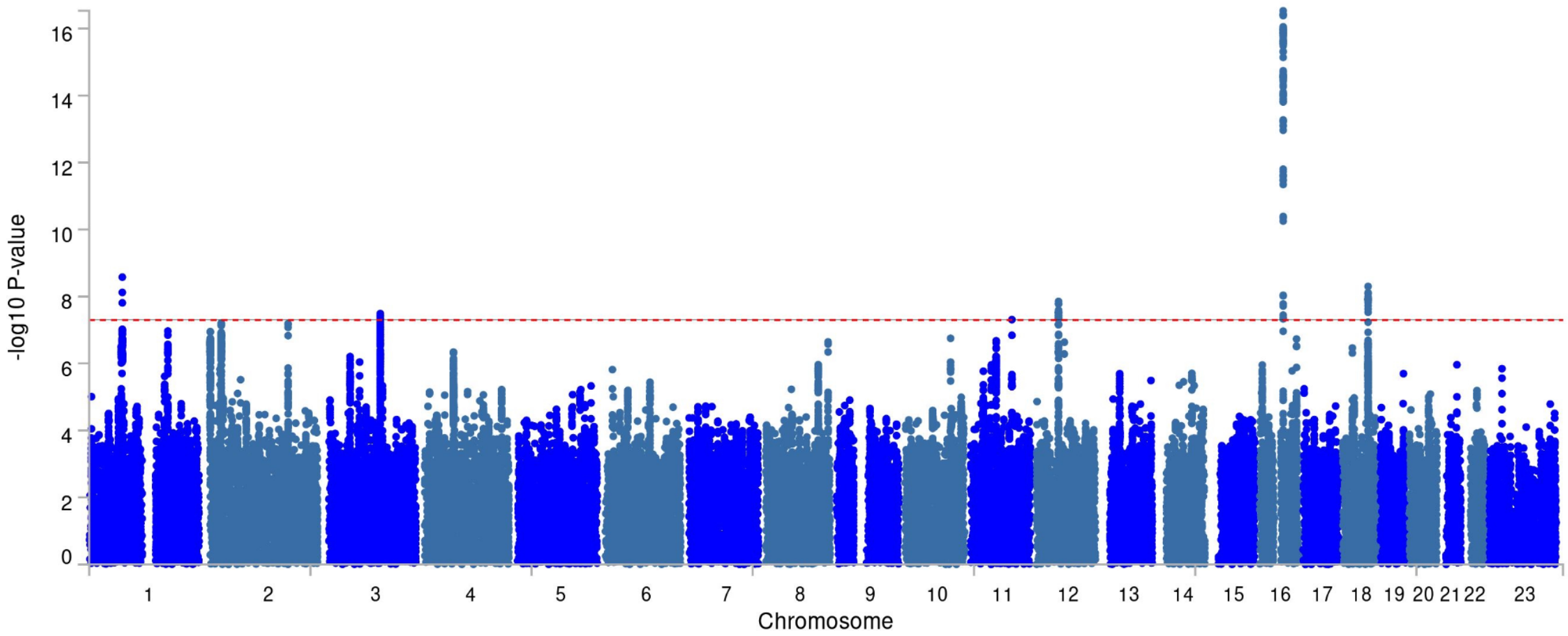

b) AN (24,223 cases, 1,243,971 controls)

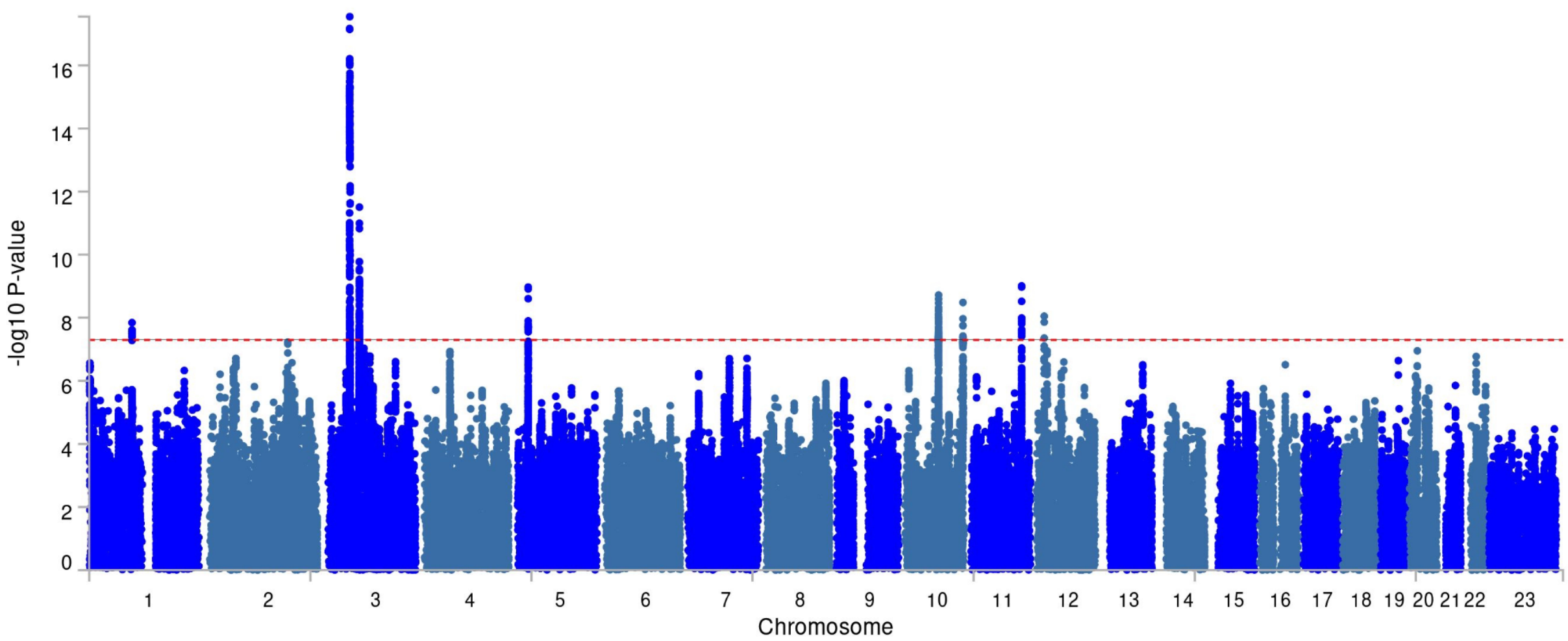

c) BE-NARROW (15,175 cases and 1,217,725 controls)

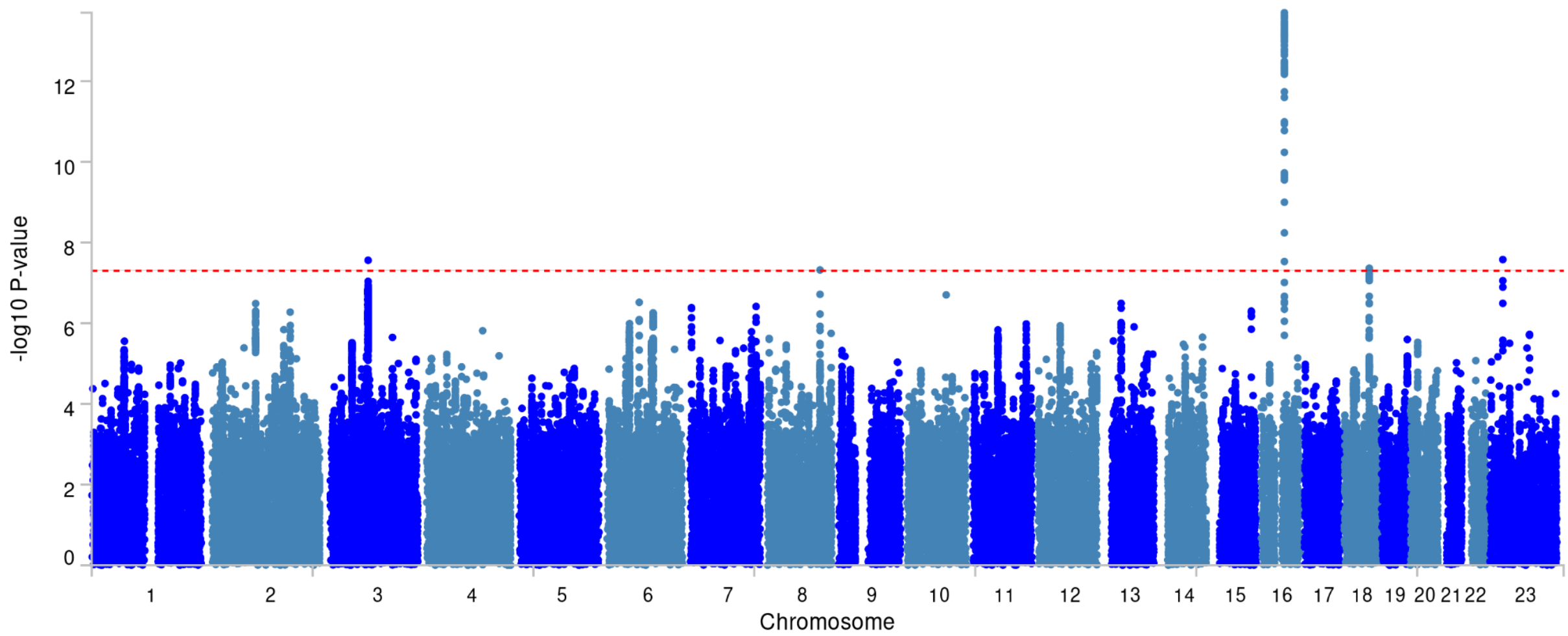

d) AN-R (2524 cases, 33,374 controls)

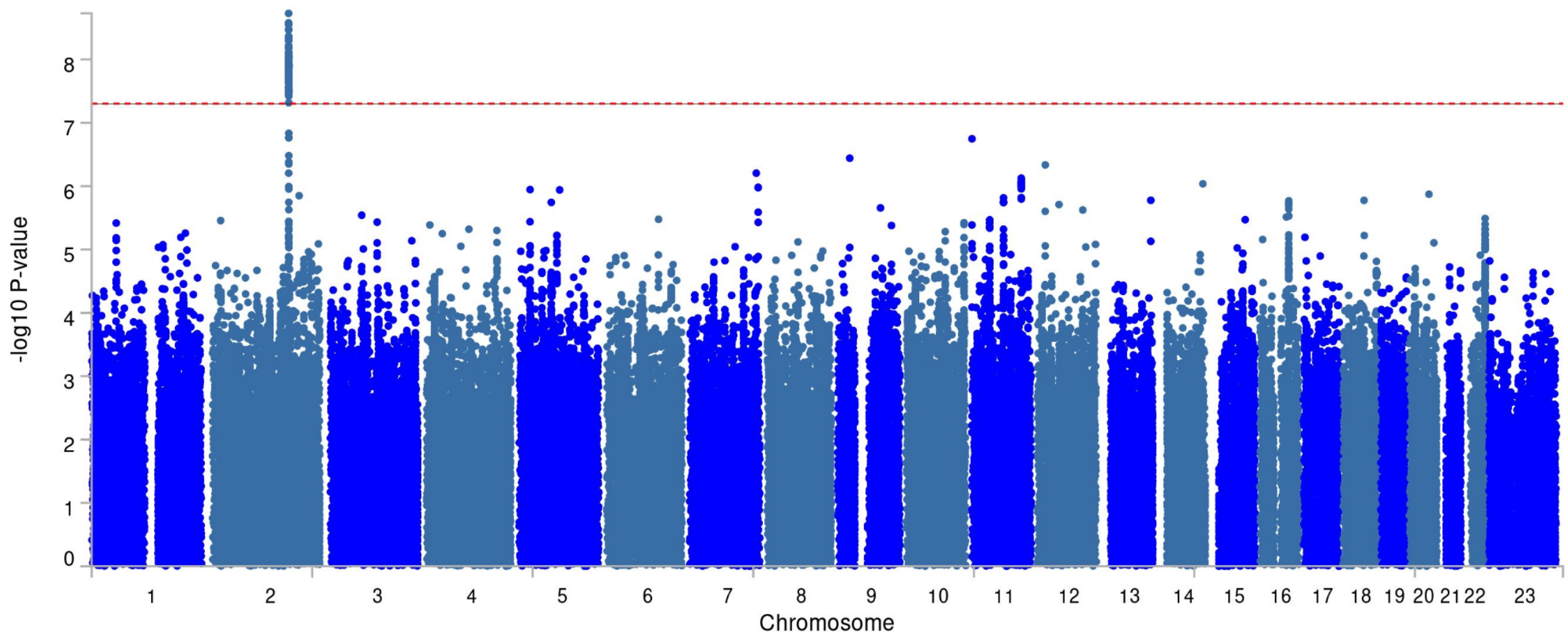

e) AN-BP (5209 cases, 33,951 controls)

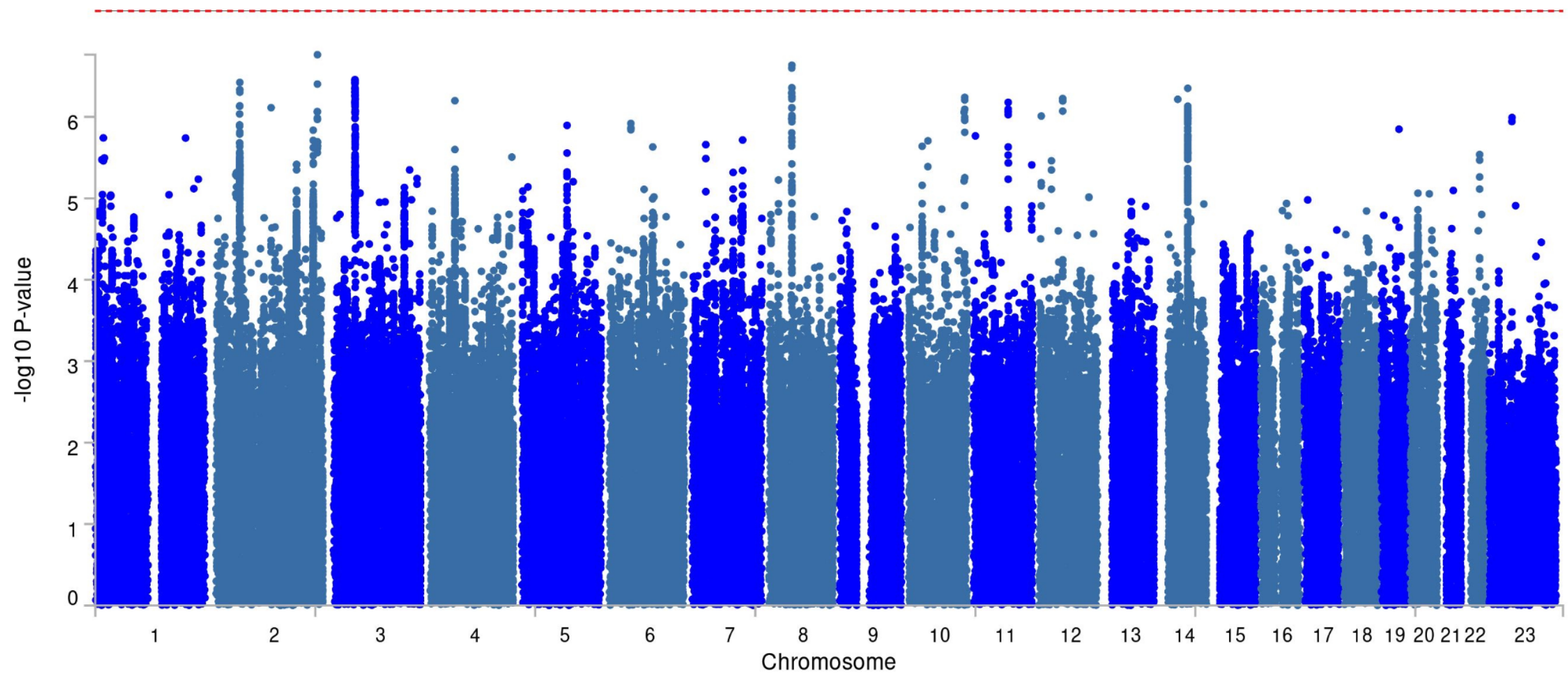

Supplementary Figure 16: Significant differences between the genetic correlations (r_g) of external traits with BE-BROAD and AN

Genetic correlations were computed by Linkage Disequilibrium Score Regression (LDSC). Dot = r_g estimate, lines = standard errors. Significance determined via the Bonferroni method. Information about the summary statistics used in our analysis can be found in Supplementary Table 8.

a) Significant differences

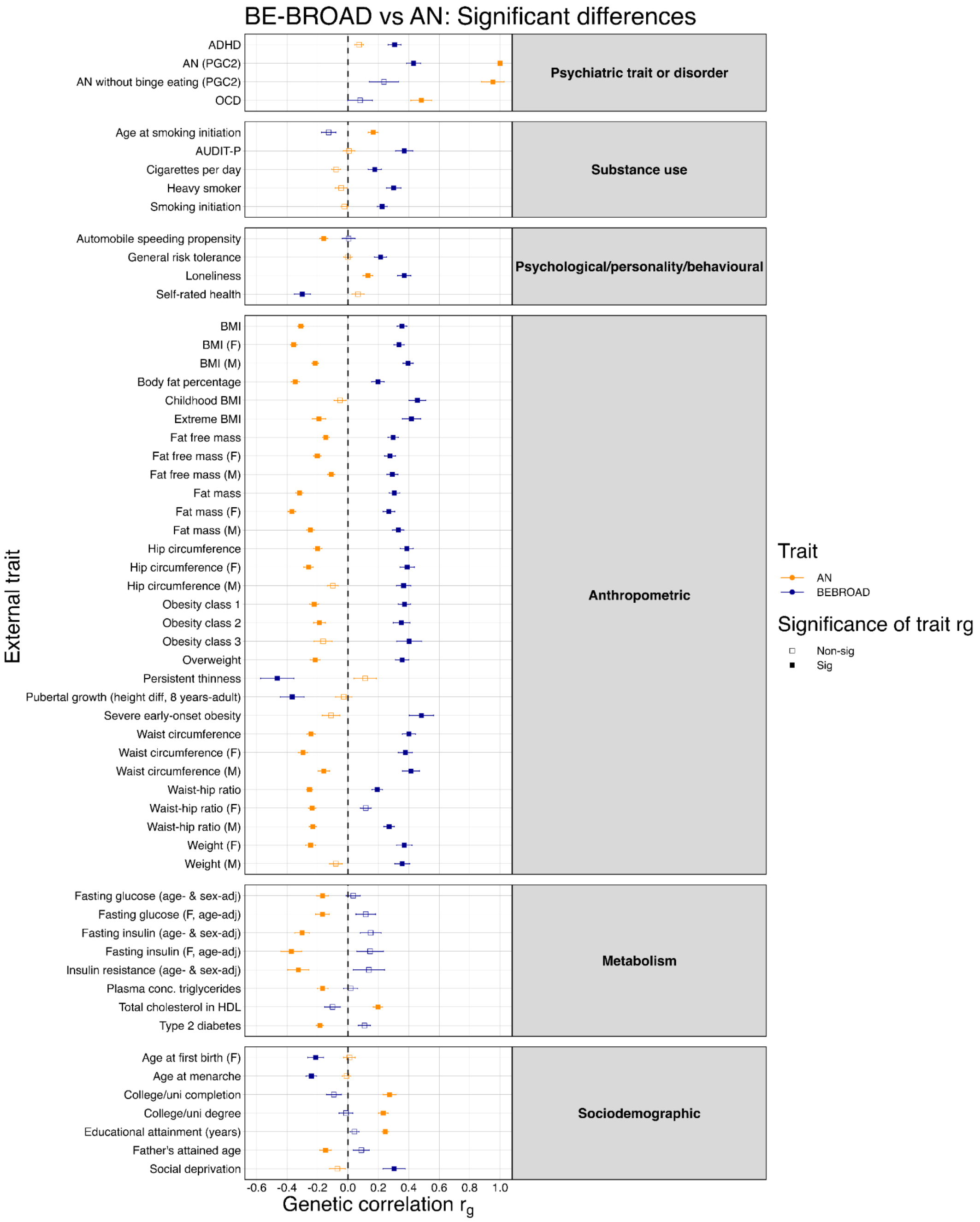

BE-BROAD = binge-eating broad definition; AN = anorexia nervosa; ADHD = attention deficit hyperactivity disorder; PGC2 = Psychiatric Genomics Consortium Freeze 2; OCD = obsessive compulsive disorder; AUDIT-P = Alcohol Use Disorder Identification Test problem items; BMI = body mass index; F = female; M = male; HDL = high-density lipoprotein

b) Non-significant differences

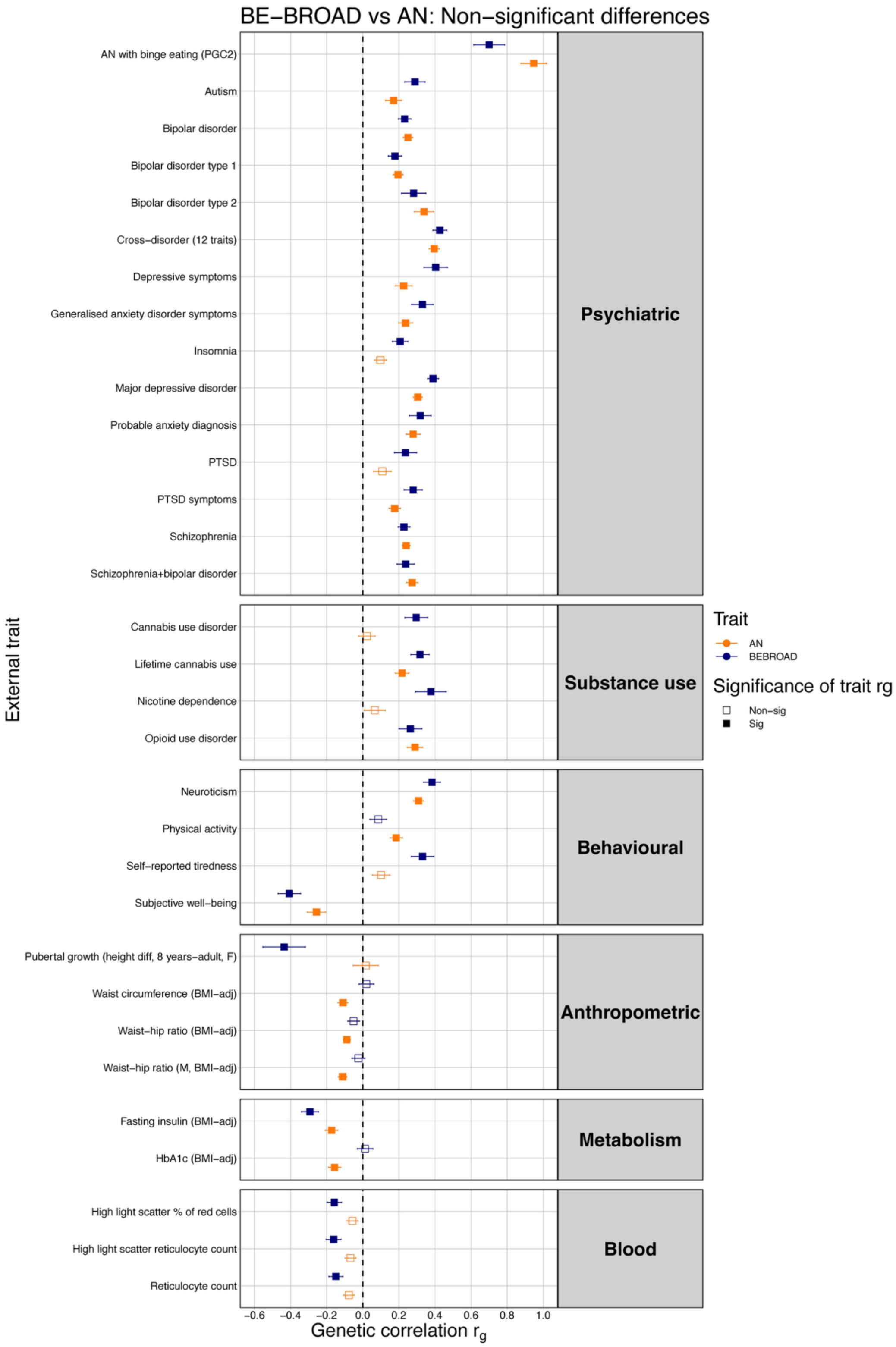

BE-BROAD = binge-eating broad definition; AN = anorexia nervosa; PGC = Psychiatric Genomics Consortium; PTSD = post-traumatic stress disorder; BMI-adj = adjusted for body mass index

Supplementary Figure 17: Comparison of genetic correlations (r_g) of selected external traits with not-ascertained-for-AN BE-BROAD (BE-BROAD$_{NAAN}$) and BE-BROAD

Genetic correlations were computed by Linkage Disequilibrium Score Regression (LDSC). The rg estimation is indicated by the dot and standard errors are indicated by the lines on either side of each dot. rg estimates have been corrected for multiple testing via the Bonferroni method. Information about the summary statistics used in our analysis can be found in Supplementary Table 8.

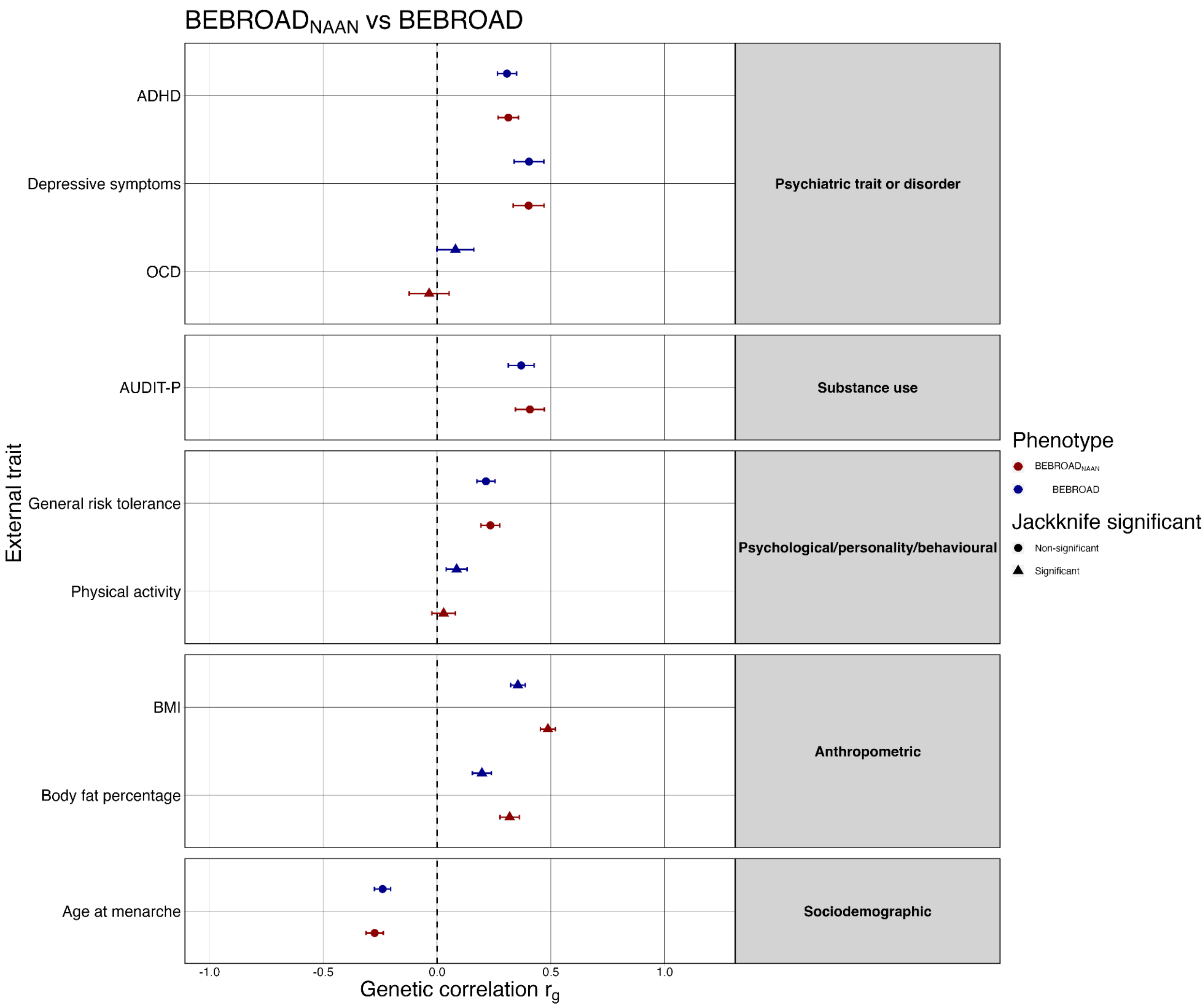

BE-BROAD$_{NAAN}$ = BE-BROAD from cohorts not-ascertained-for-AN; BE-BROAD = binge-eating broad definition; AN = anorexia nervosa; ADHD = attention deficit hyperactivity disorder; OCD = obsessive compulsive disorder; AUDIT-P = Alcohol Use Disorder Identification Test problem items; BMI = body mass index

Supplementary Figure 18: Comparison of the genetic correlations of BE-BROAD and AN and their respective NonBMI components with external traits

Experiment-wide significance set at $p < 2.22 \times 10^{-4}$, consistent with p-value used for genetic correlations throughout the paper. Trait-level significance represented by darkened circles; jackknife significance represented by solid black connection between original and nonBMI trait. Traits tested noted on y-axis, trait categories indicated between BE-BROAD (left) and AN (right).

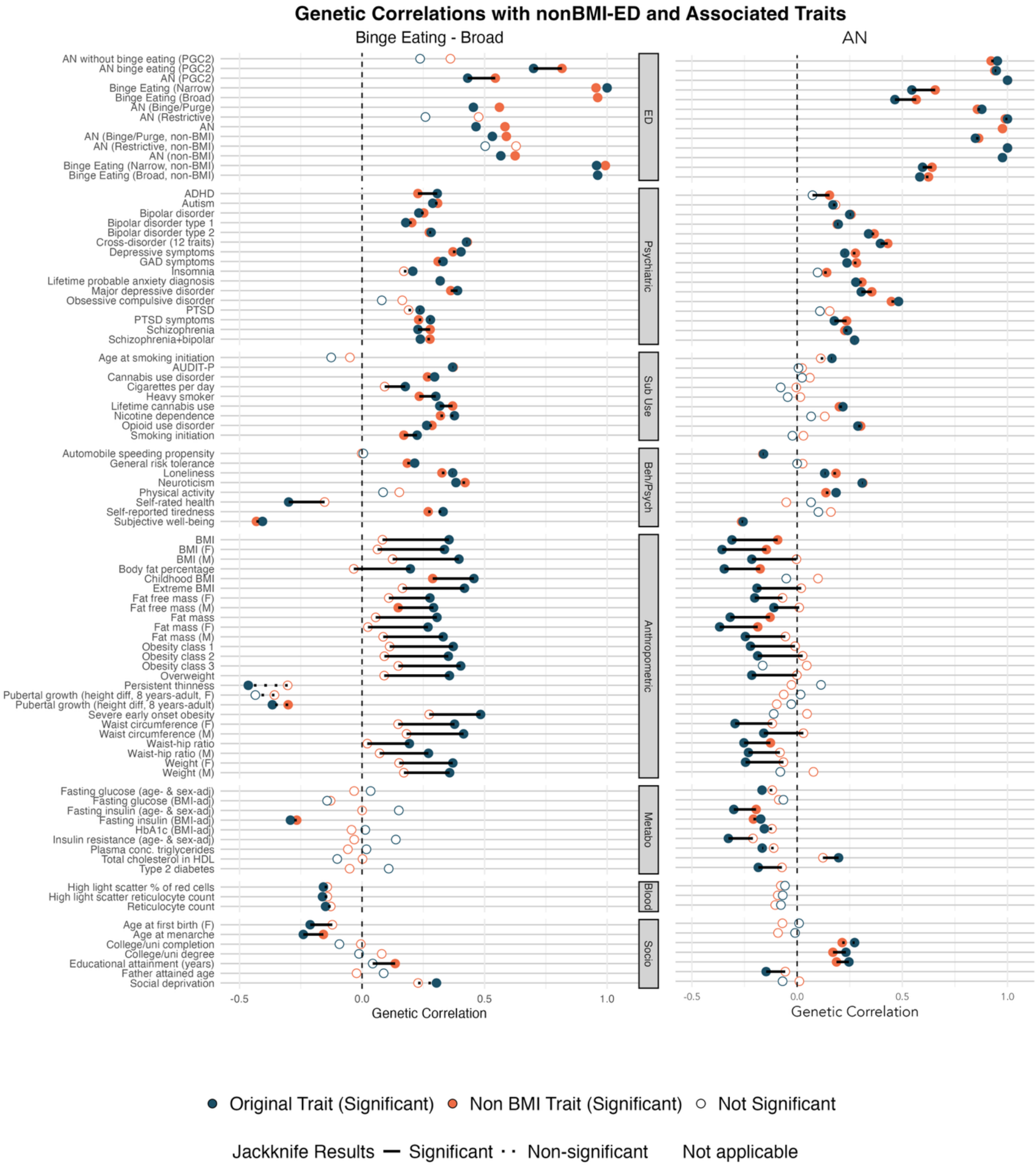

Supplementary Figure 19: Gene-tissue associations from S-PrediXcan

Enrichment of gene-tissue associations using two-tailed exact binomial tests, testing for enrichment of associations at (1) nominal significance, $p<0.05$, (2) tissue-specific significance, $p<0.05/\text{Tests}_{\text{TissueX}}$, and (3) experiment-wide significance, $p<8.32 \times 10^{-8}$. Manhattan plot of S-PrediXcan gene-tissue associations with annotations of top genes. Experiment-wide significance threshold of $p<8.32 \times 10^{-8}$ (dotted line); experiment-wide associations are highlighted in red; tissue-specific associations ($p<0.05/\text{Tests}_{\text{TissueX}}$) are shown in other colours.

a) BE-BROAD (39,279 cases, 1,227,436 controls)

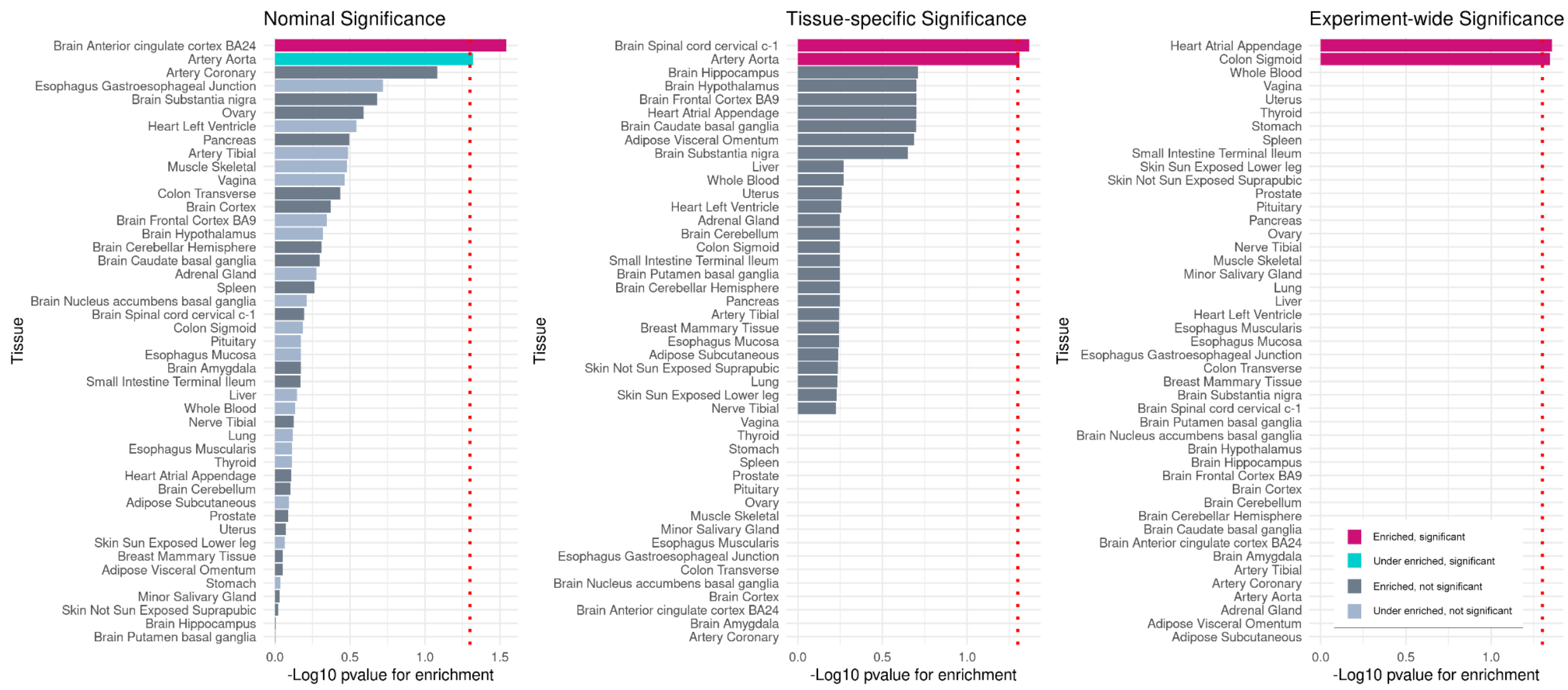

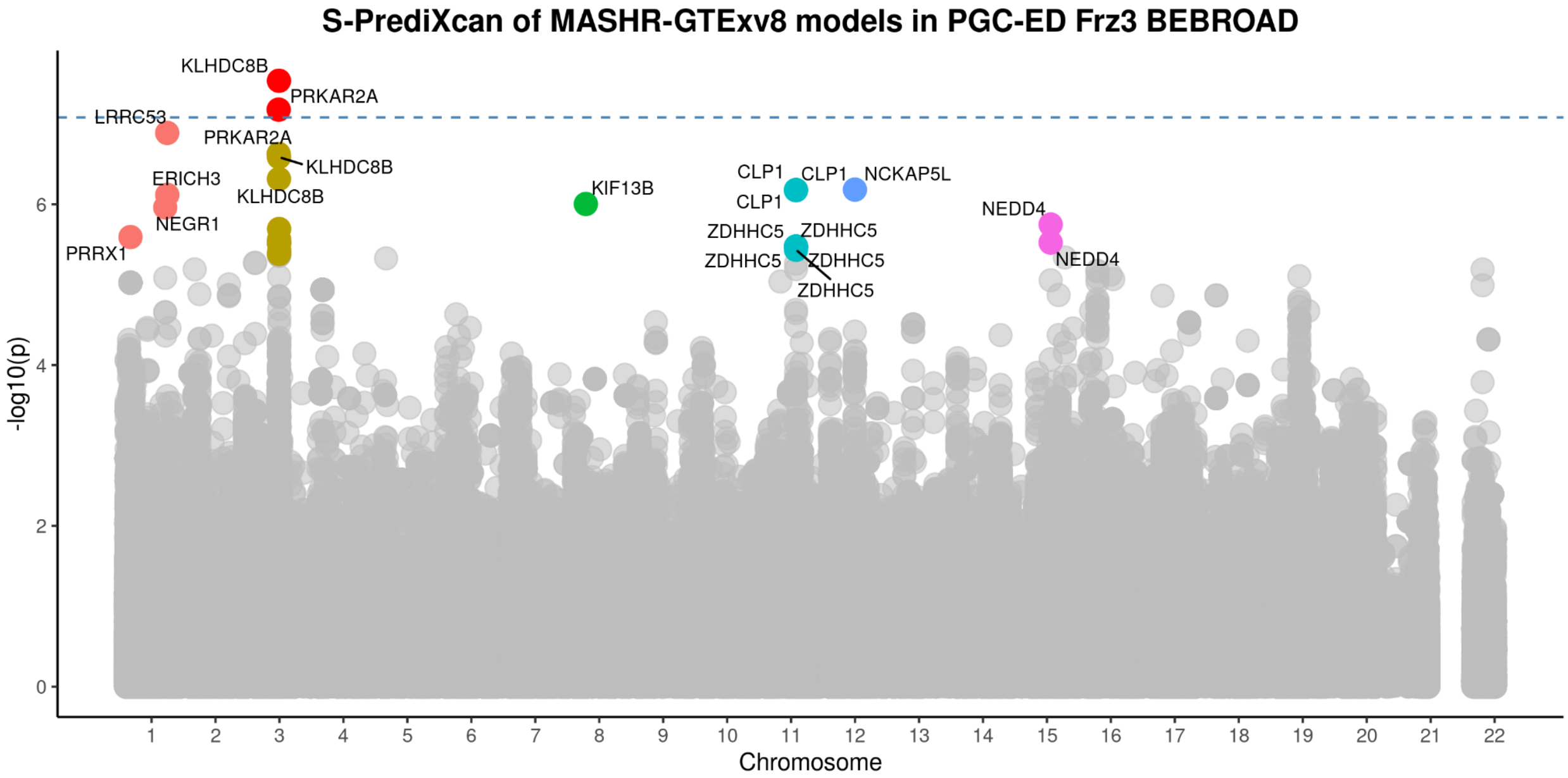

b) AN (24,223 cases, 1,243,971 controls)

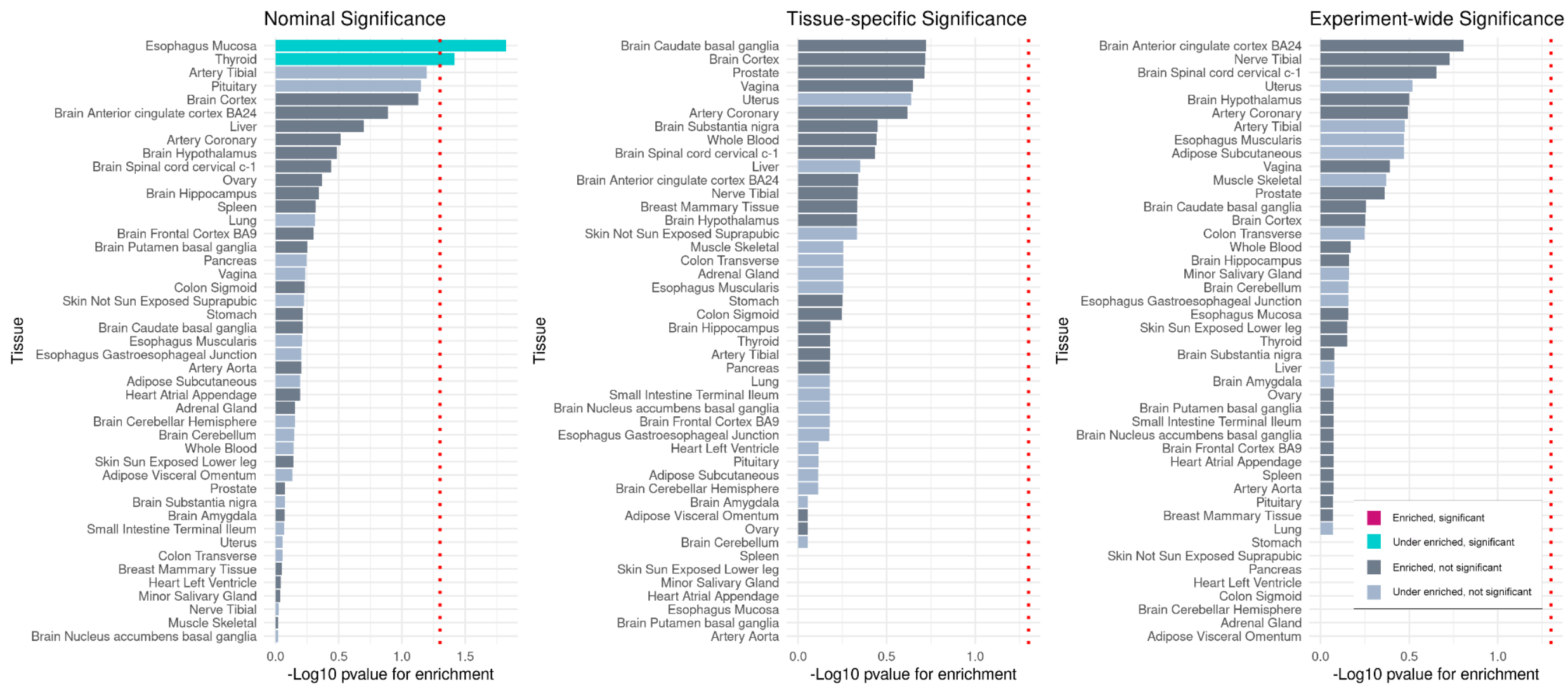

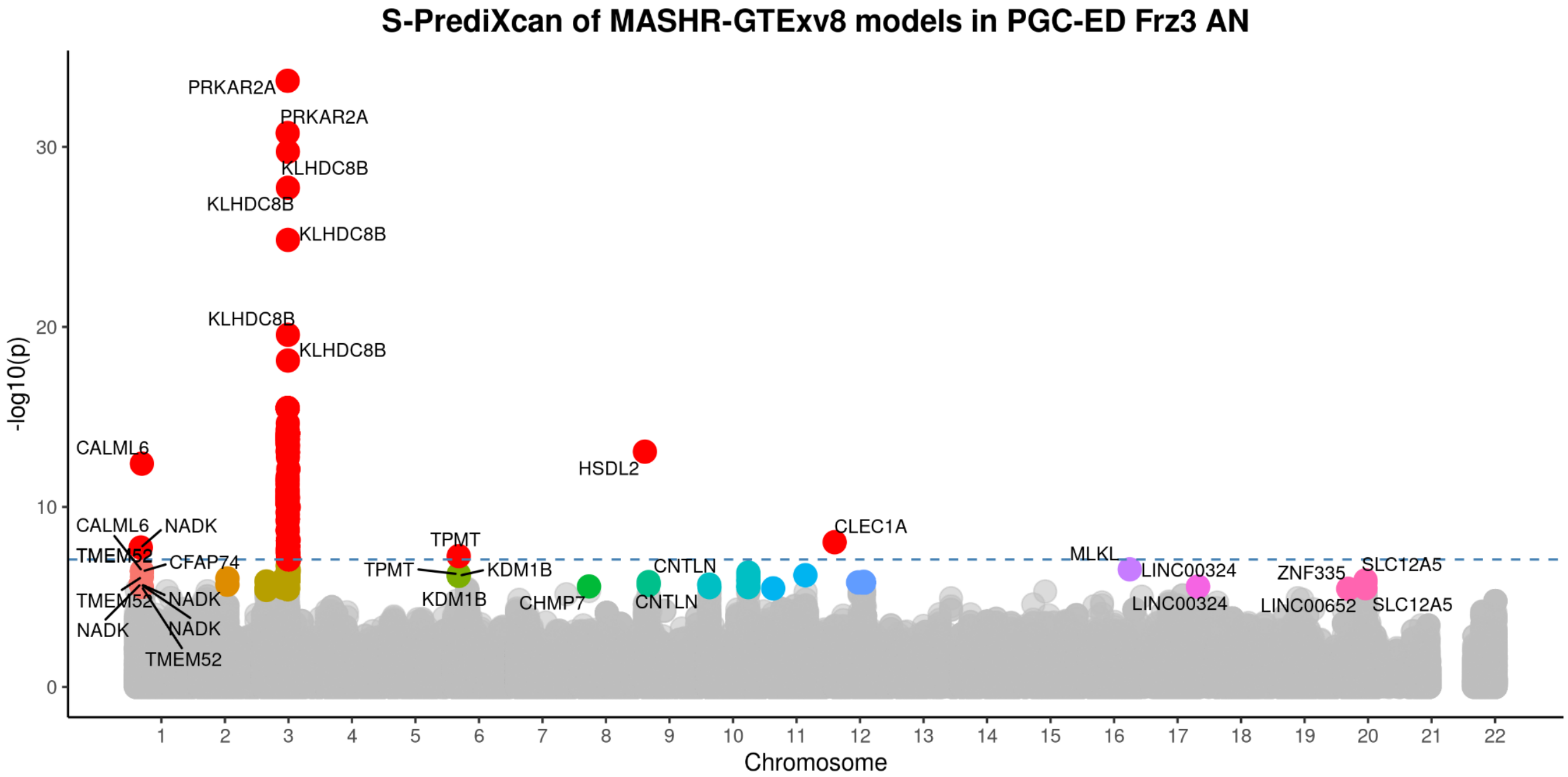

c) BE-NARROW (15,175 cases and 1,217,725 controls)

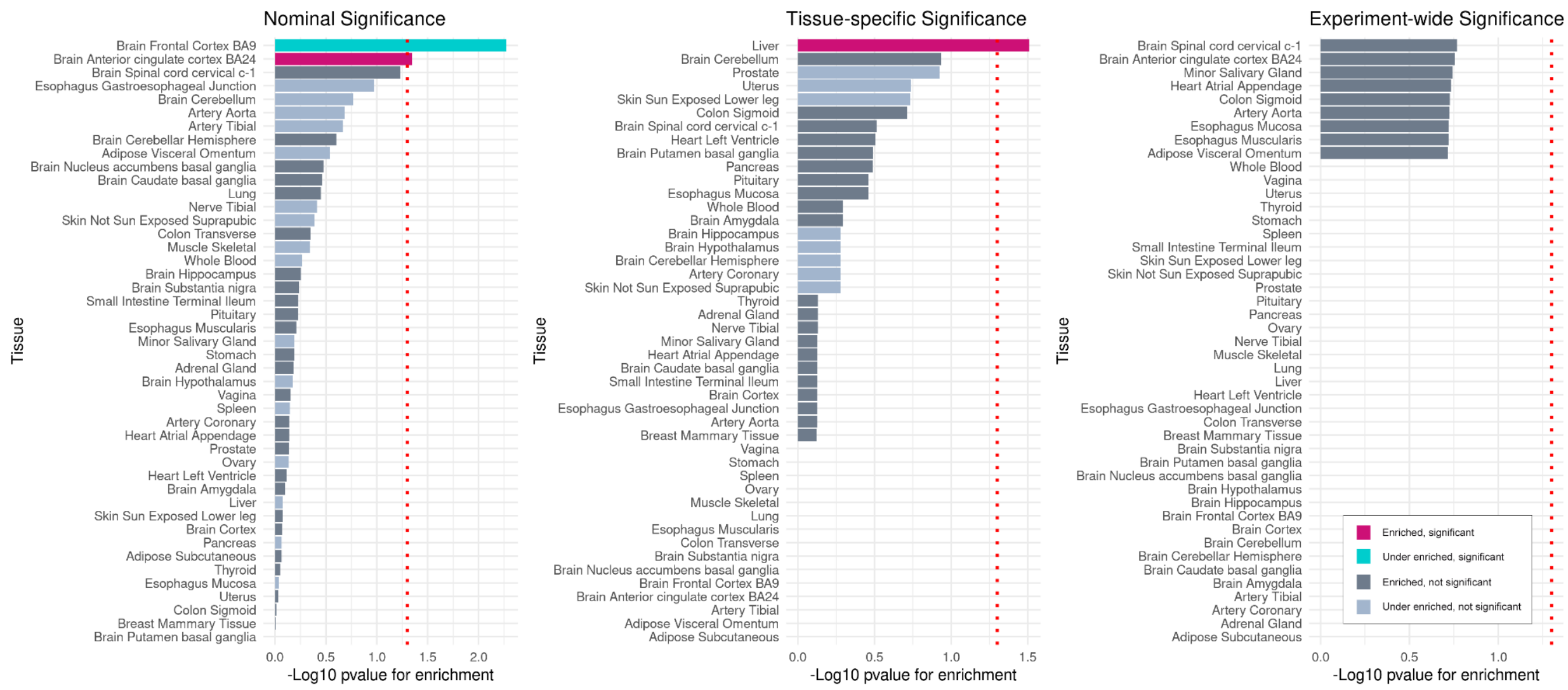

d) AN-R (2524 cases, 33,374 controls)

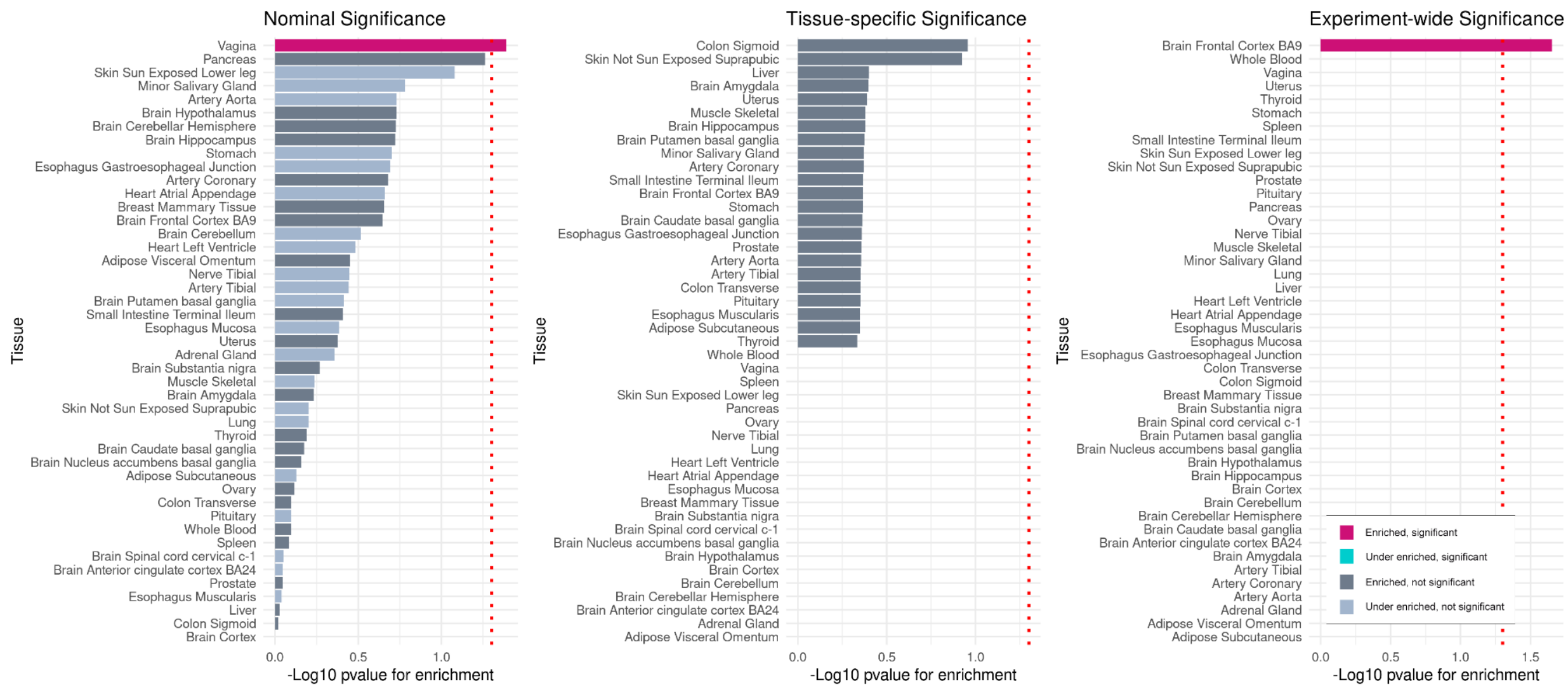

e) AN-BP (5209 cases, 33,951 controls)

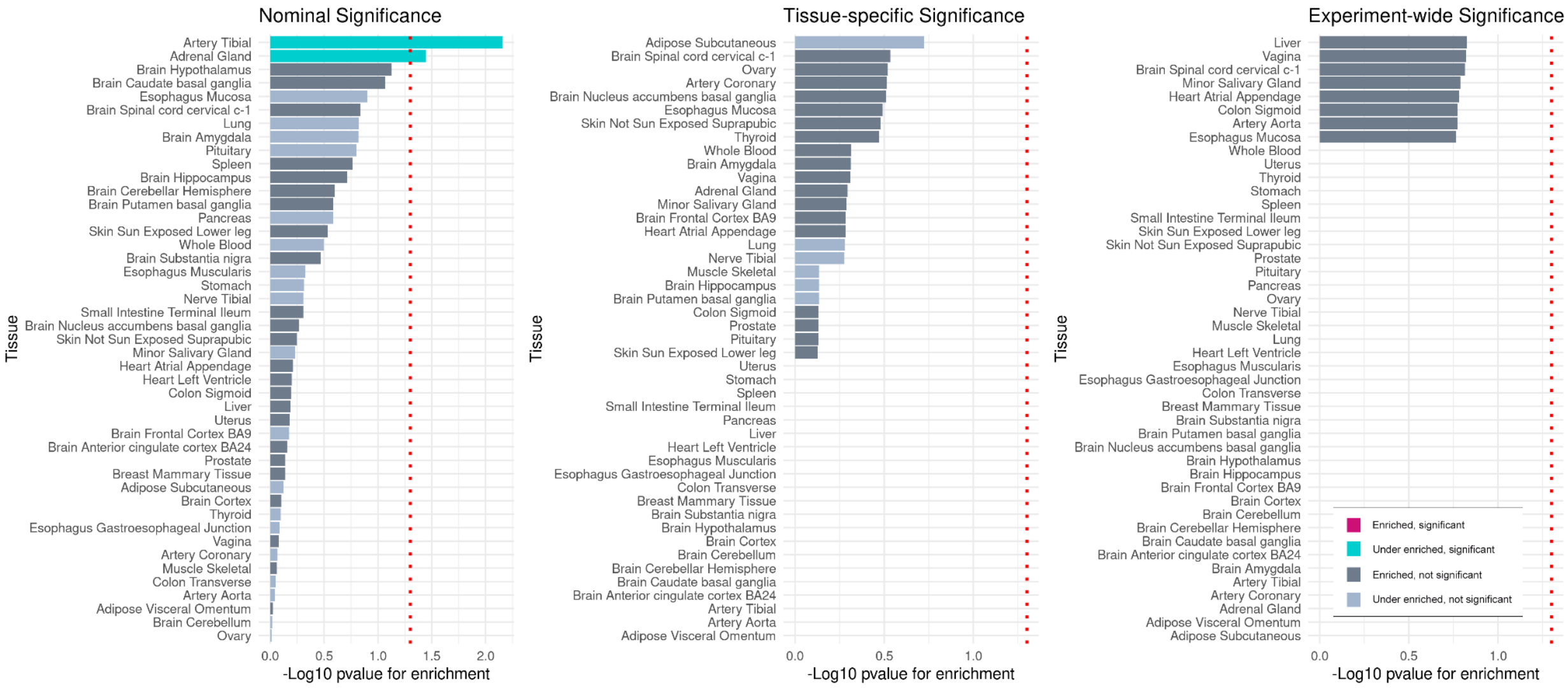

Supplementary Figure 20: Cross-tissue gene-wise associations from S-MultiXcan

Manhattan plot of significant gene-tissue associations (coloured points) from S-MultiXcan results, with annotation of top genes. Experiment-wide significance threshold of $p<2.25 \times 10^{-6}$ (dotted line).

a) BE-BROAD (39,279 cases, 1,227,436 controls)

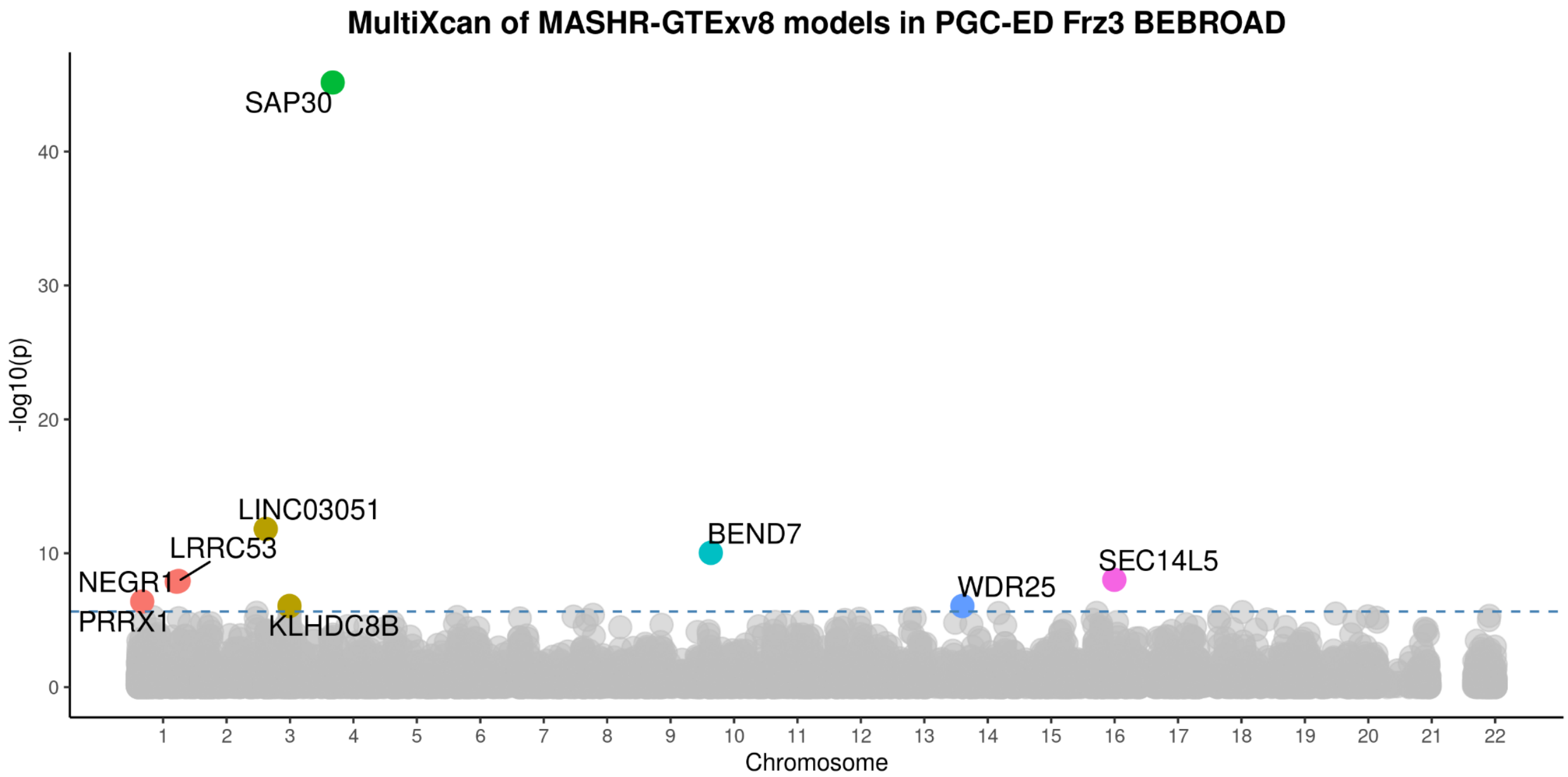

b) AN (24,223 cases, 1,243,971 controls)

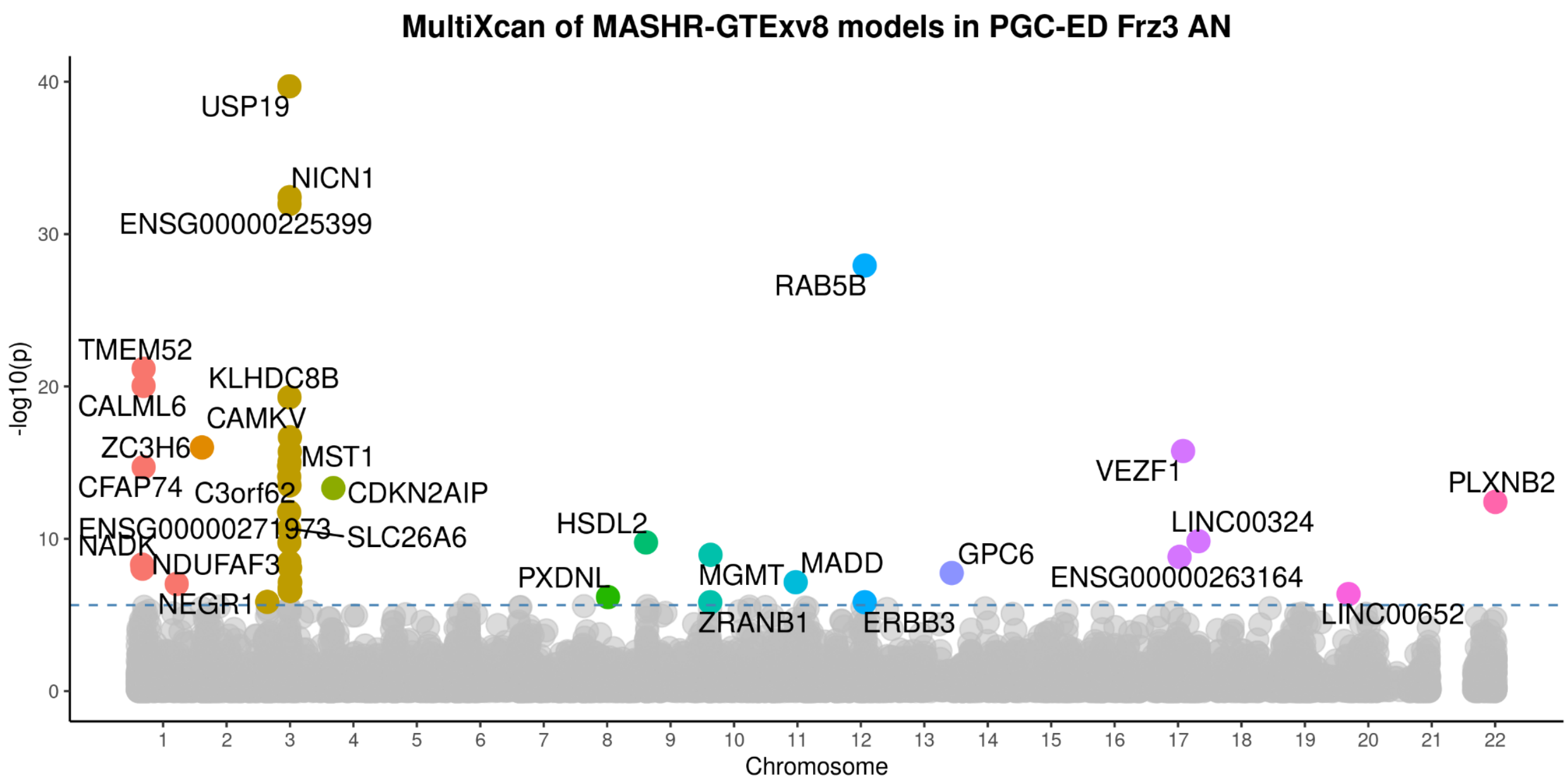

c) BE-NARROW (15,175 cases and 1,217,725 controls)

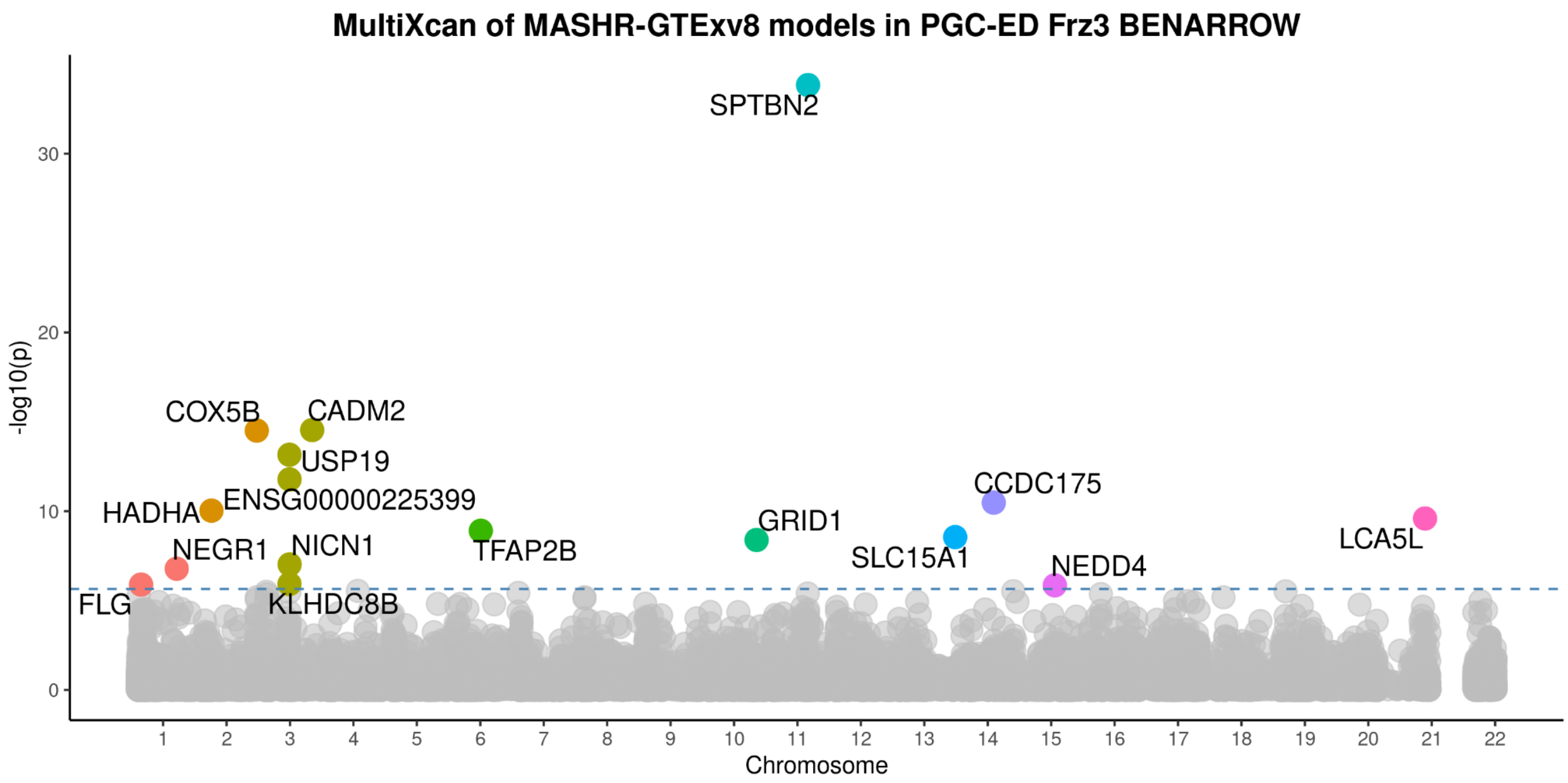

d) AN-R (2524 cases, 33,374 controls)

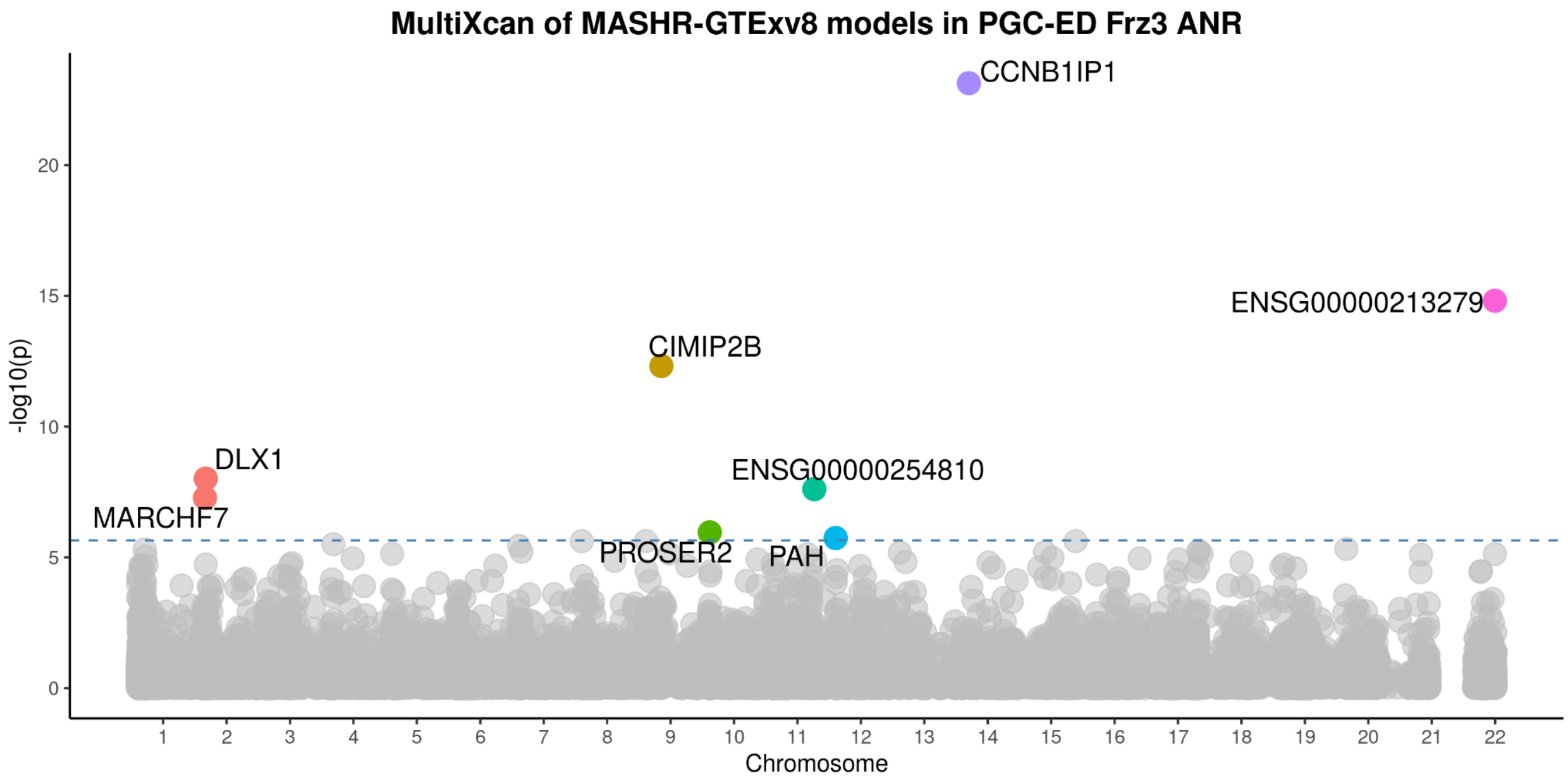

e) AN-BP (5209 cases, 33,951 controls)

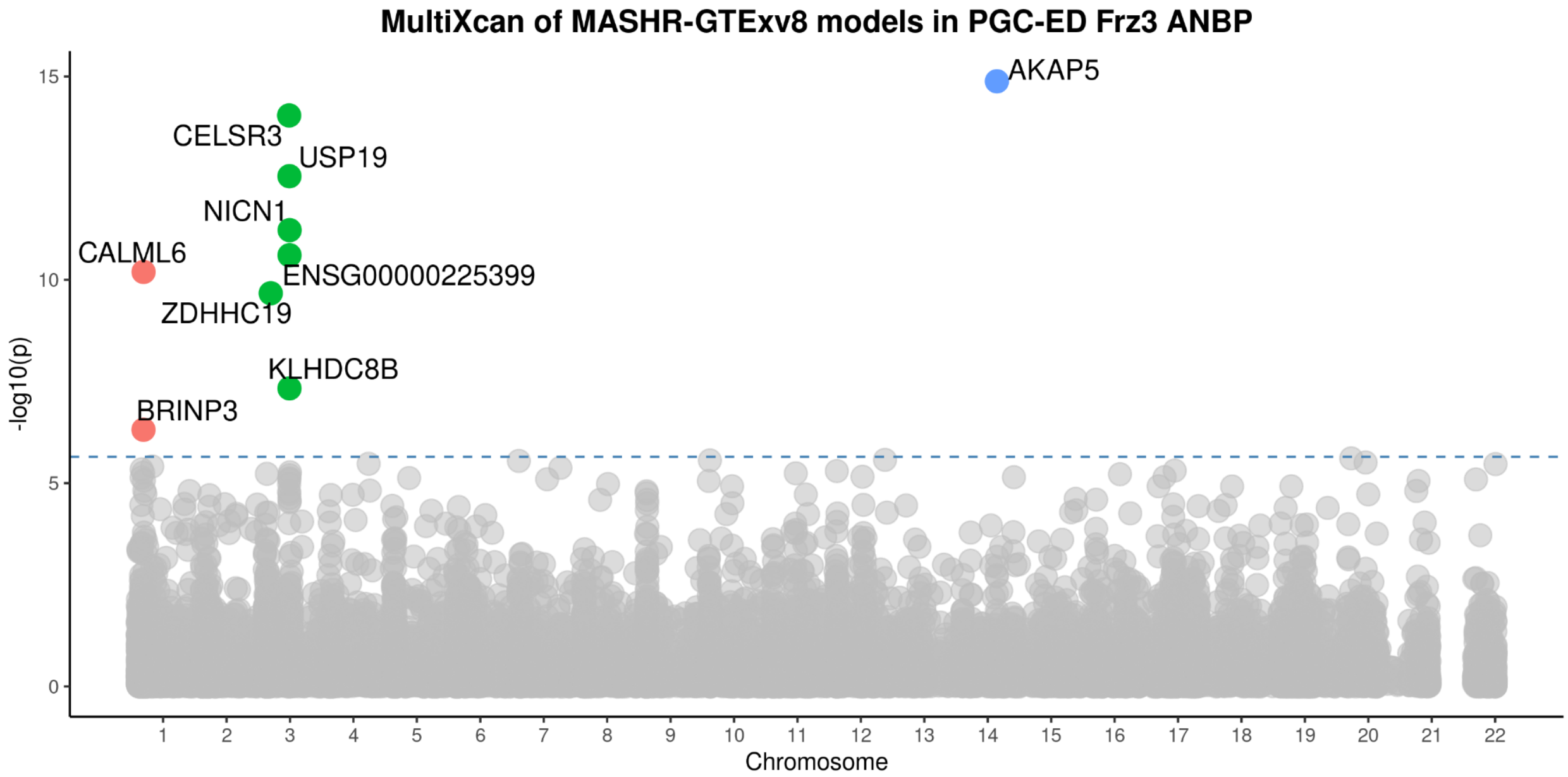

Supplementary Figure 21: Tissue analyses in GTEx human tissues

This figure presents the significance of the enrichment of eating disorder heritability in human tissues; expression data from GTEx version 8. (a) Barchart for the false discovery rate (FDR) on -log10 scale; dashed line indicates FDR threshold at 0.05. No results survived multiple testing corrections. (b) Heatmap of the unadjusted enrichment P value on -log10 scale. Brain tissues (brain-cortex and brain-other) showed relatively higher levels of significance for all tested eating disorders.

<u>*Supplementary Figure 21a*</u>

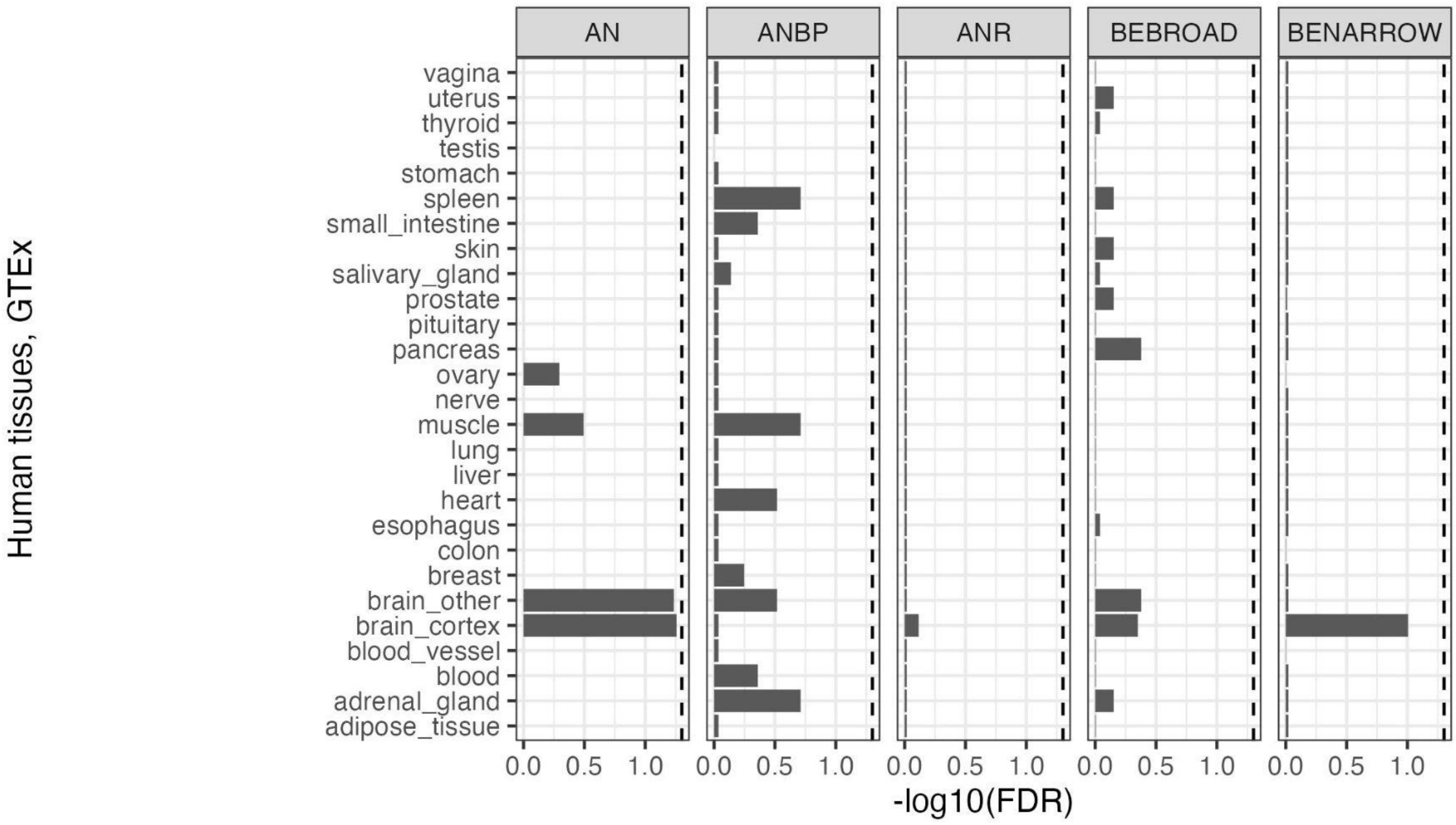

Supplementary Figure 21b

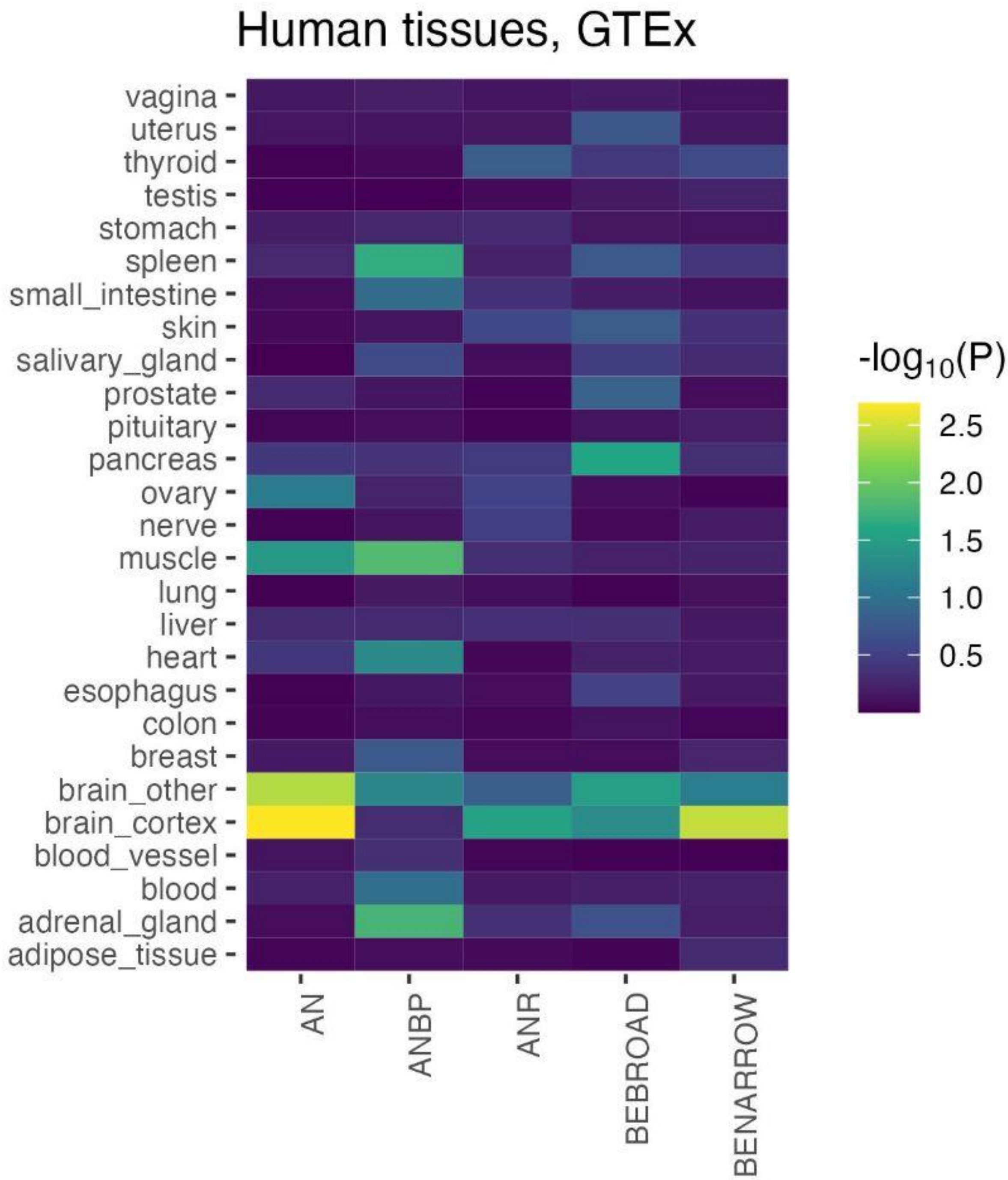

Supplementary Figure 22: Cell-type analyses in human brain cell types

This figure presents the significance of the enrichment of eating disorder heritability in human brain cell type; based on the 31 superclusters from the single-nucleus RNA-sequencing adult brain atlas. (a) Barchart for the false discovery rate (FDR) on -log10 scale; dashed line indicates FDR threshold at 0.05. No results survived multiple testing corrections. (b) Heatmap of the unadjusted enrichment P value on -log10 scale. The highest significance results are observed in the upper-layer intratelencephalic neurons for BEBROAD. For AN, the higher levels of significance were seen in inhibitory neurons, particularly caudal ganglionic eminence (CGE) interneurons, followed by excitatory neurons.

Supplementary Figure 22a

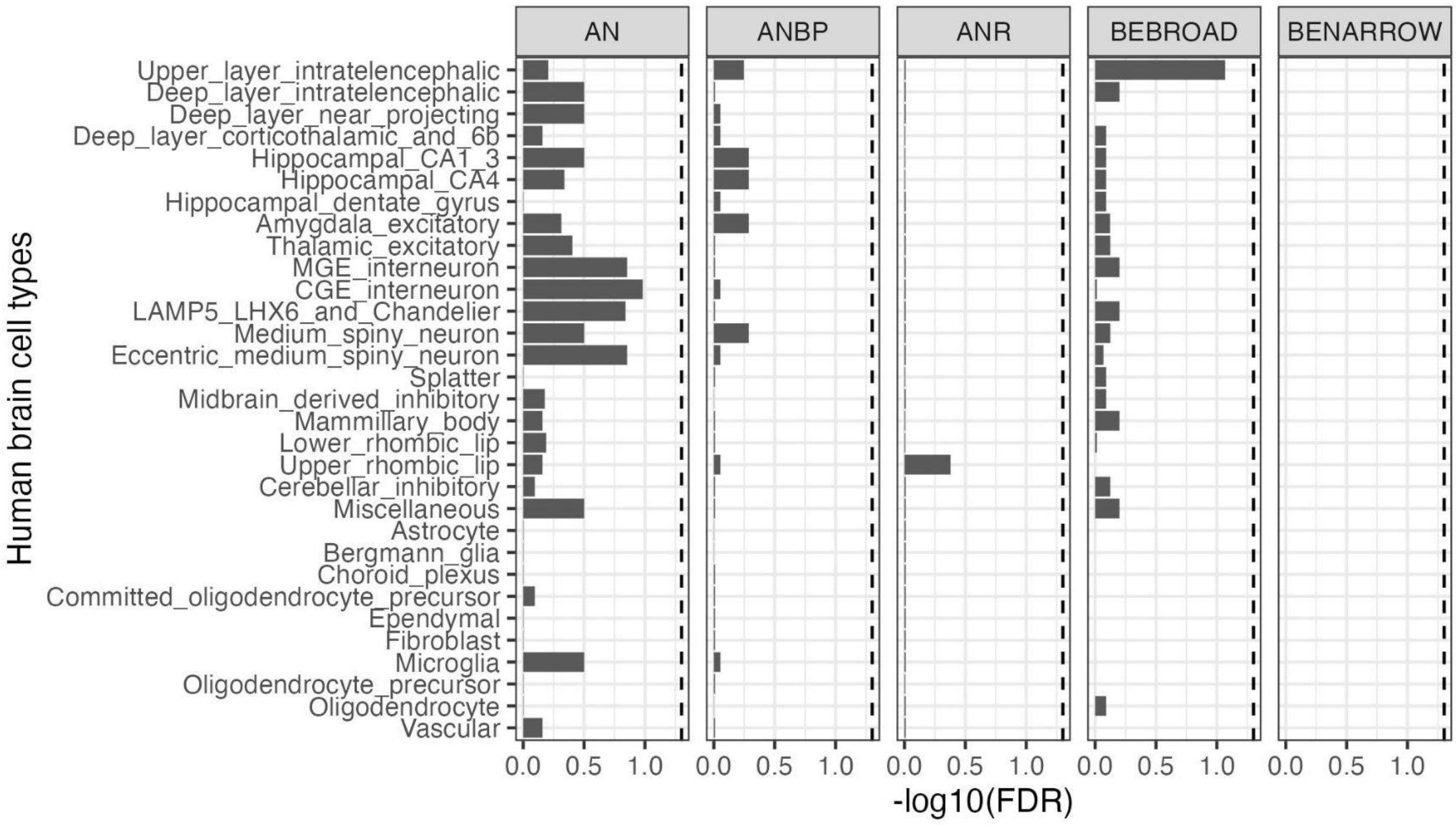

Supplementary Figure 22b

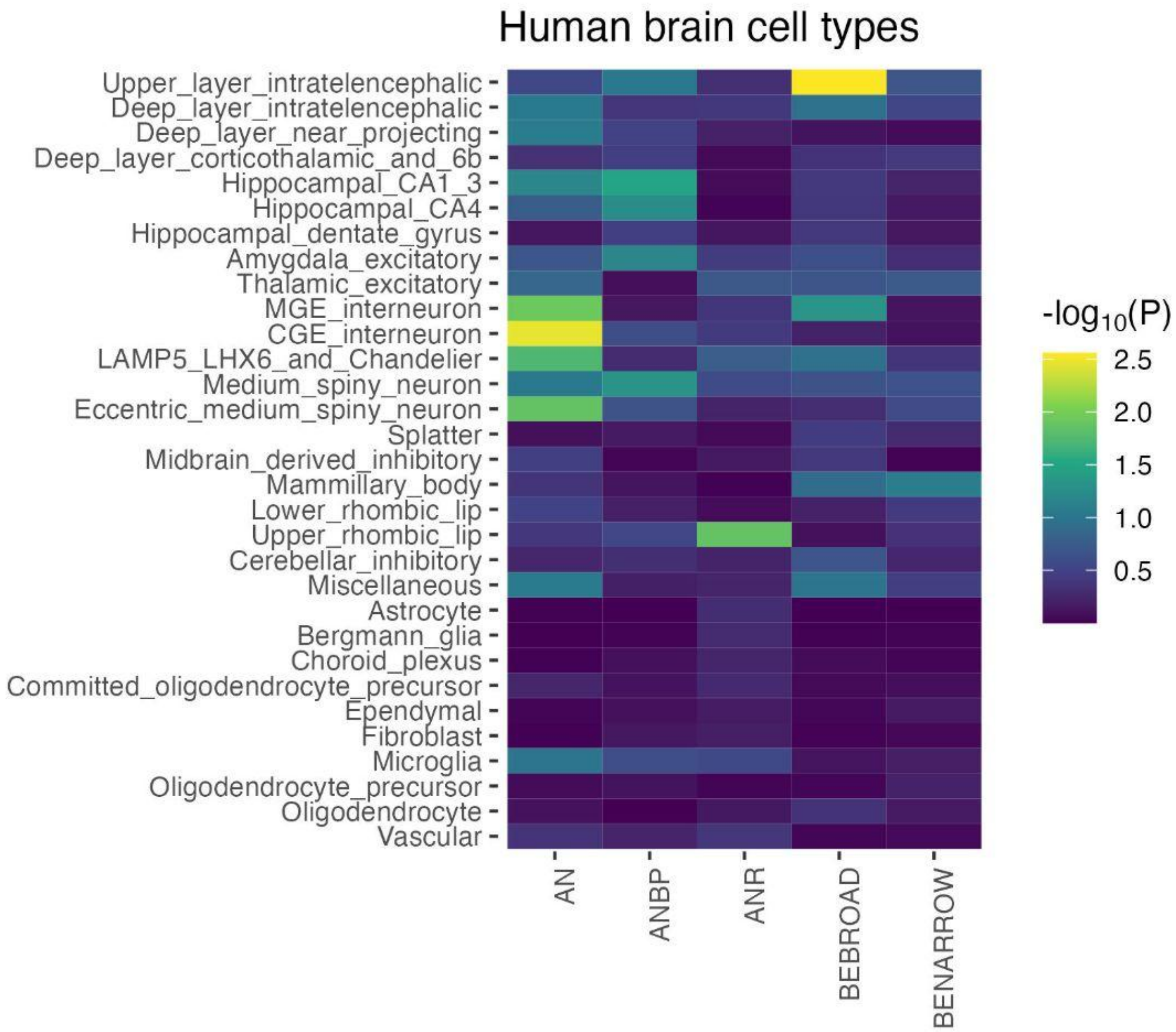

Supplementary Figure 23: Within-and-cross-trait polygenic scoring

Decile plots depict the odds ratio (OR) of each decile of polygenic risk scores of BE-BROAD, BE-NARROW, and AN on the BE-BROAD, BE-NARROW, AN outcomes, with the lowest decile serving as the reference group. The receiver operating characteristic (ROC) curve is a graphical illustration of the performance of a regression model to predict the binary outcome (i.e., case/control status of BE-BROAD, BE-NARROW, or AN here). If a regression model has a ROC curve overlapping with the diagonal grey line in the ROC curve plot, it indicates that the regression model is no better than chance to differentiate cases and controls with an area under the curve (AUC) of 0.5. An AUC greater than 0.5 suggests that the prediction performance of a model is better than chance and an AUC of 1 indicates perfect performance. In each ROC curve plot, ROC curves of two models are plotted: one for the baseline model (e.g., genetic principal components (PCs) as predictor variables) and one for the PRS model (i.e., both PRS and baseline predictors are included as predictor variables). Overall, the PRS model improves the predictive performance on BE-BROAD, BE-NARROW, and AN outcomes, but to a small extent.

Supplementary Figure 23a: Decile plots and area under the receiver operating characteristic (AU-ROC) curve for BE-BROAD PRS with BE-BROAD

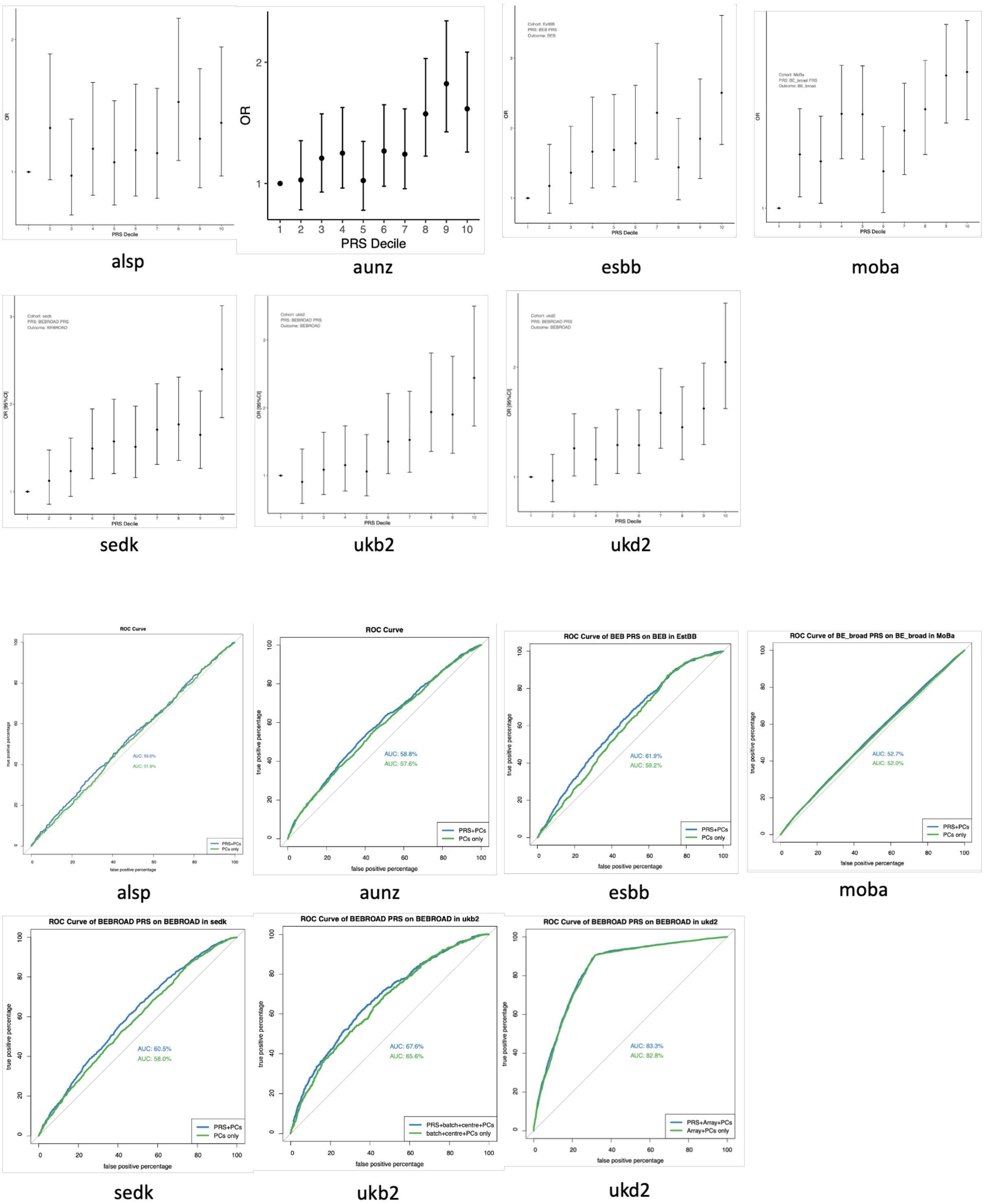

Supplementary Figure 23b: Decile plots and AU-ROC curve for AN PRS with AN

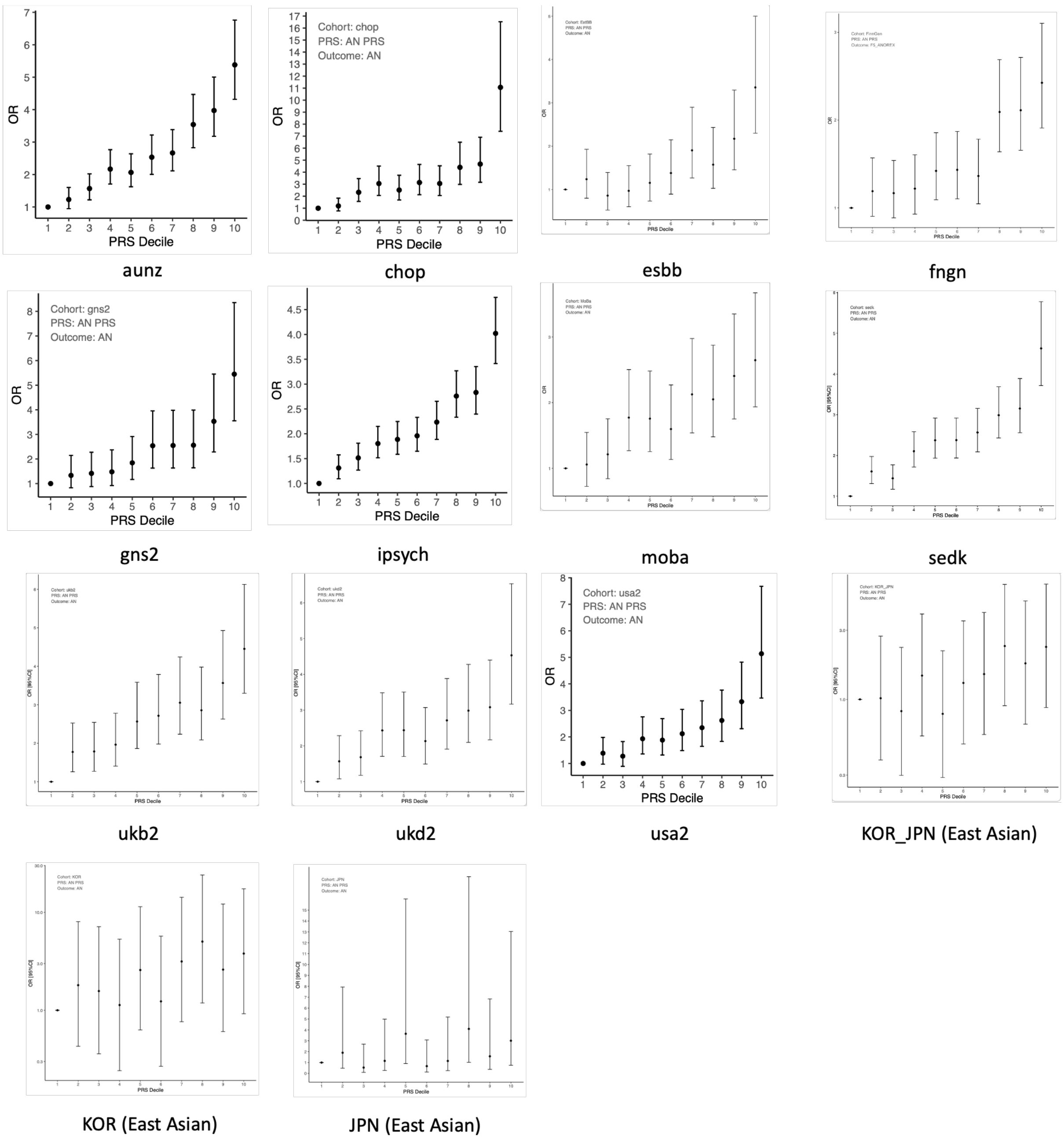

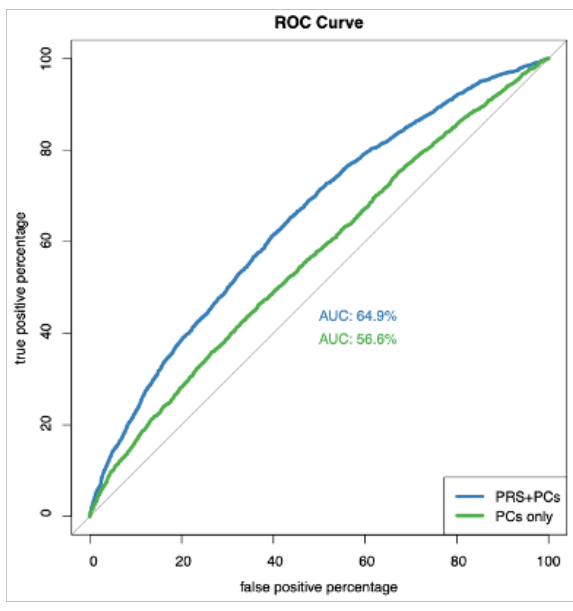

aunz

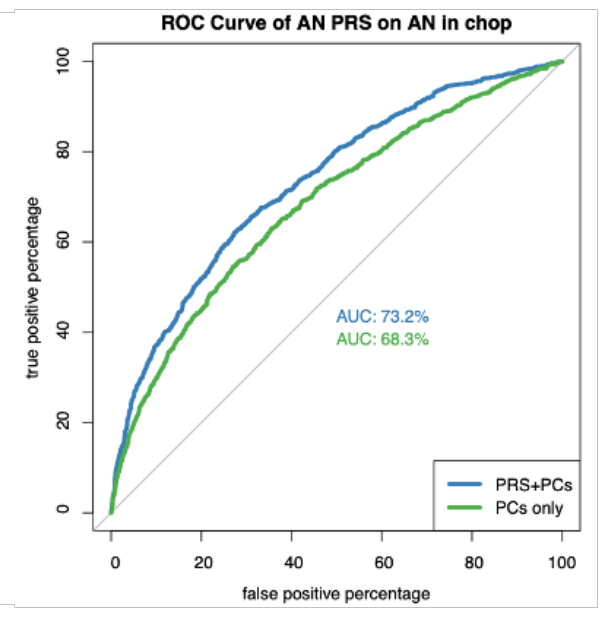

chop

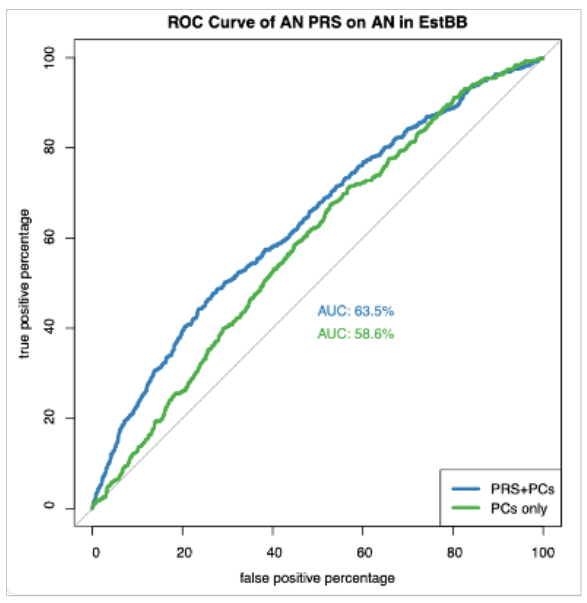

esbb

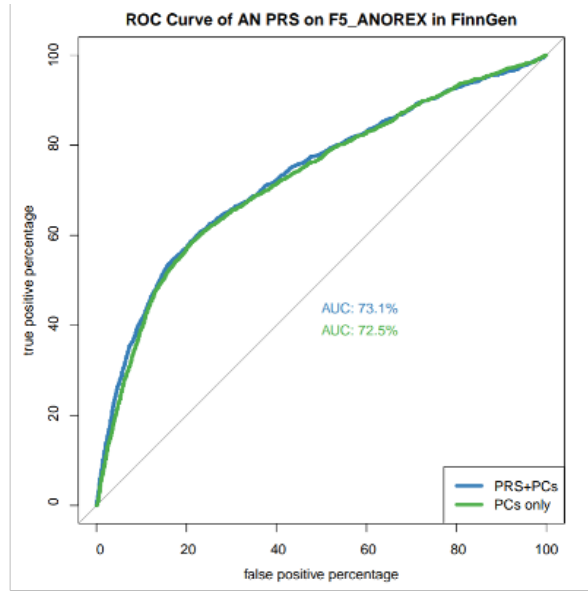

fngn

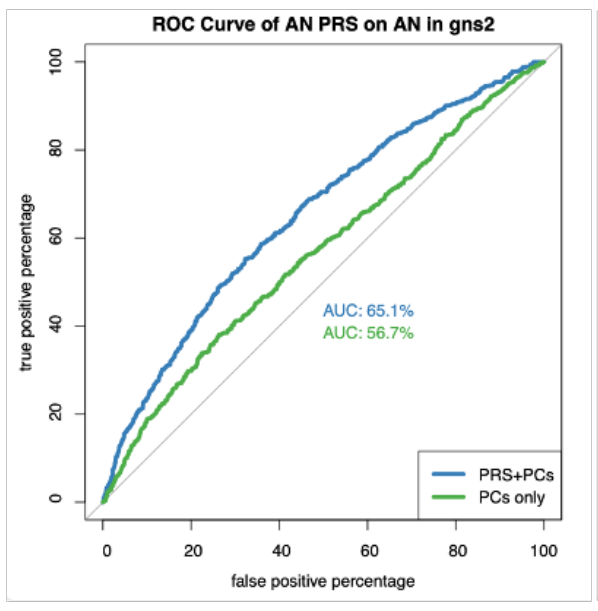

gns2

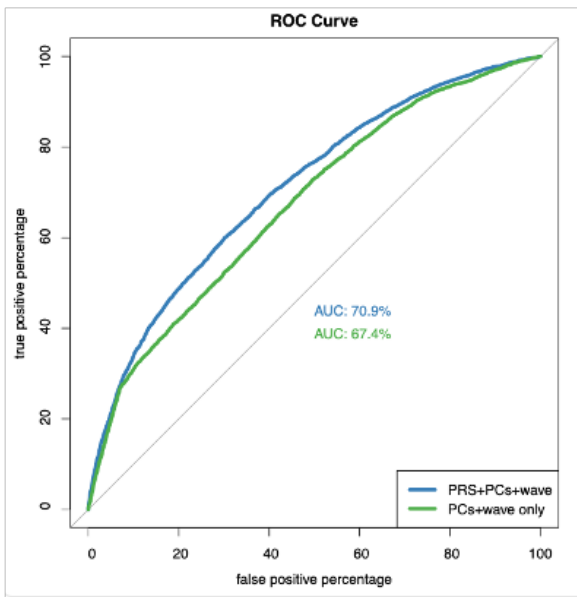

ipsy

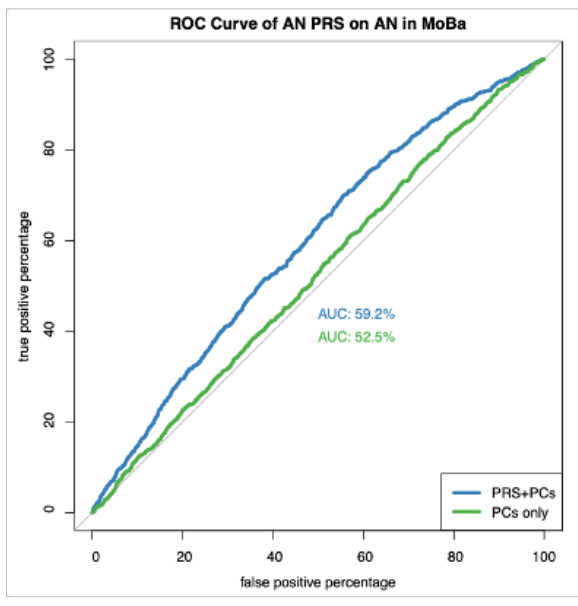

moba

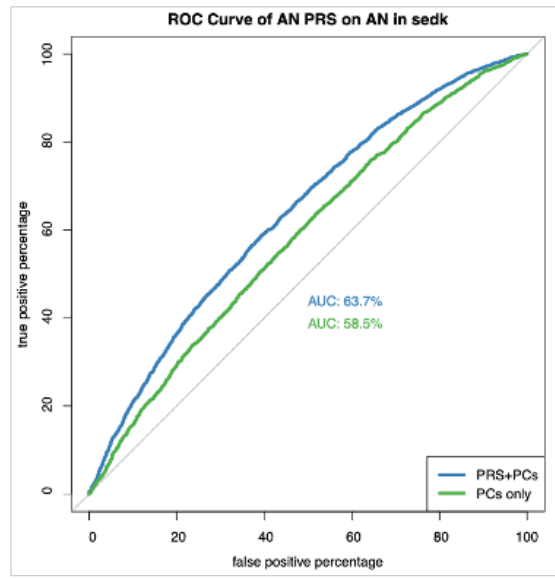

sedk

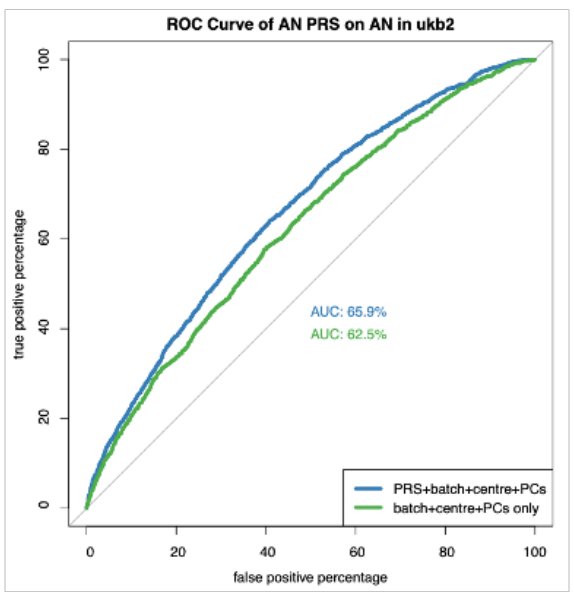

ukb2

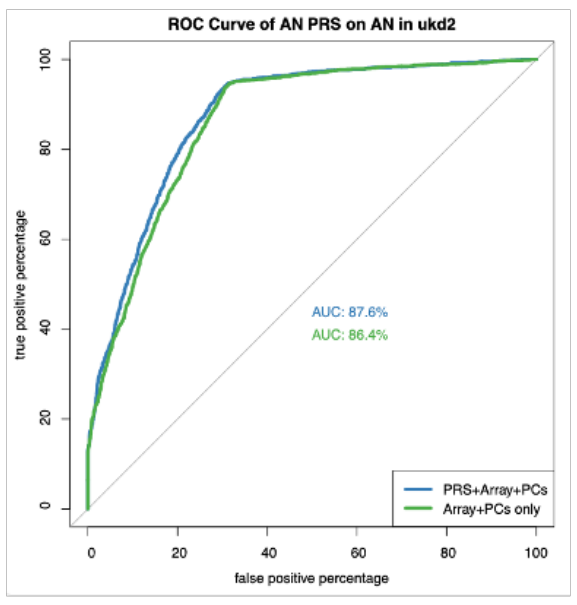

ukd2

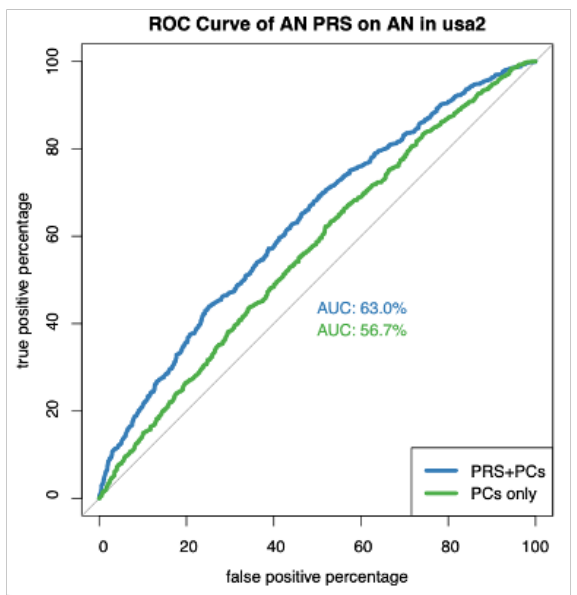

usa2

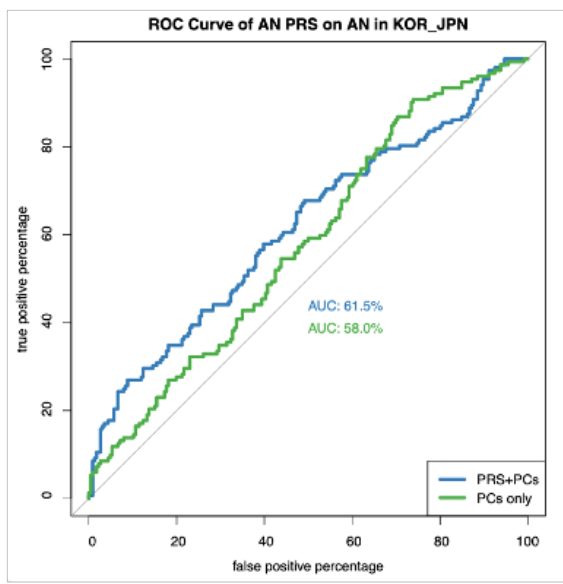

KOR_JPN (East Asian)

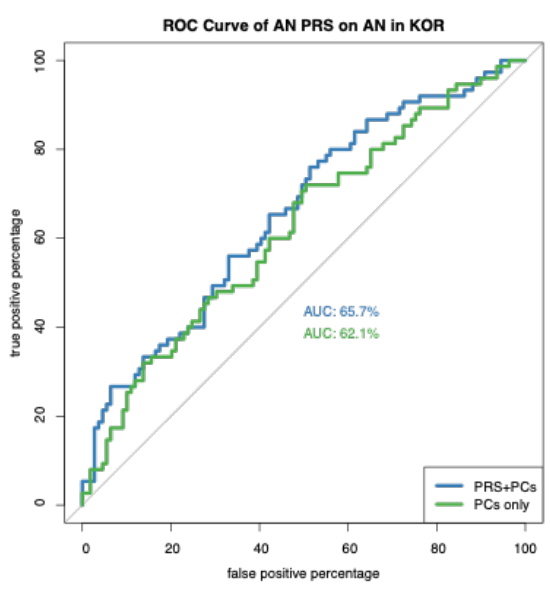

KOR (East Asian)

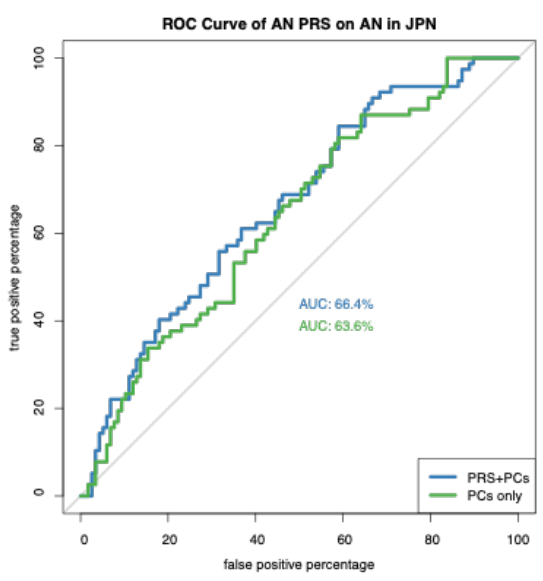

JPN (East Asian)

Supplementary Figure 23c: Decile plots and AU-ROC curve for BE-BROAD PRS with BE-NARROW

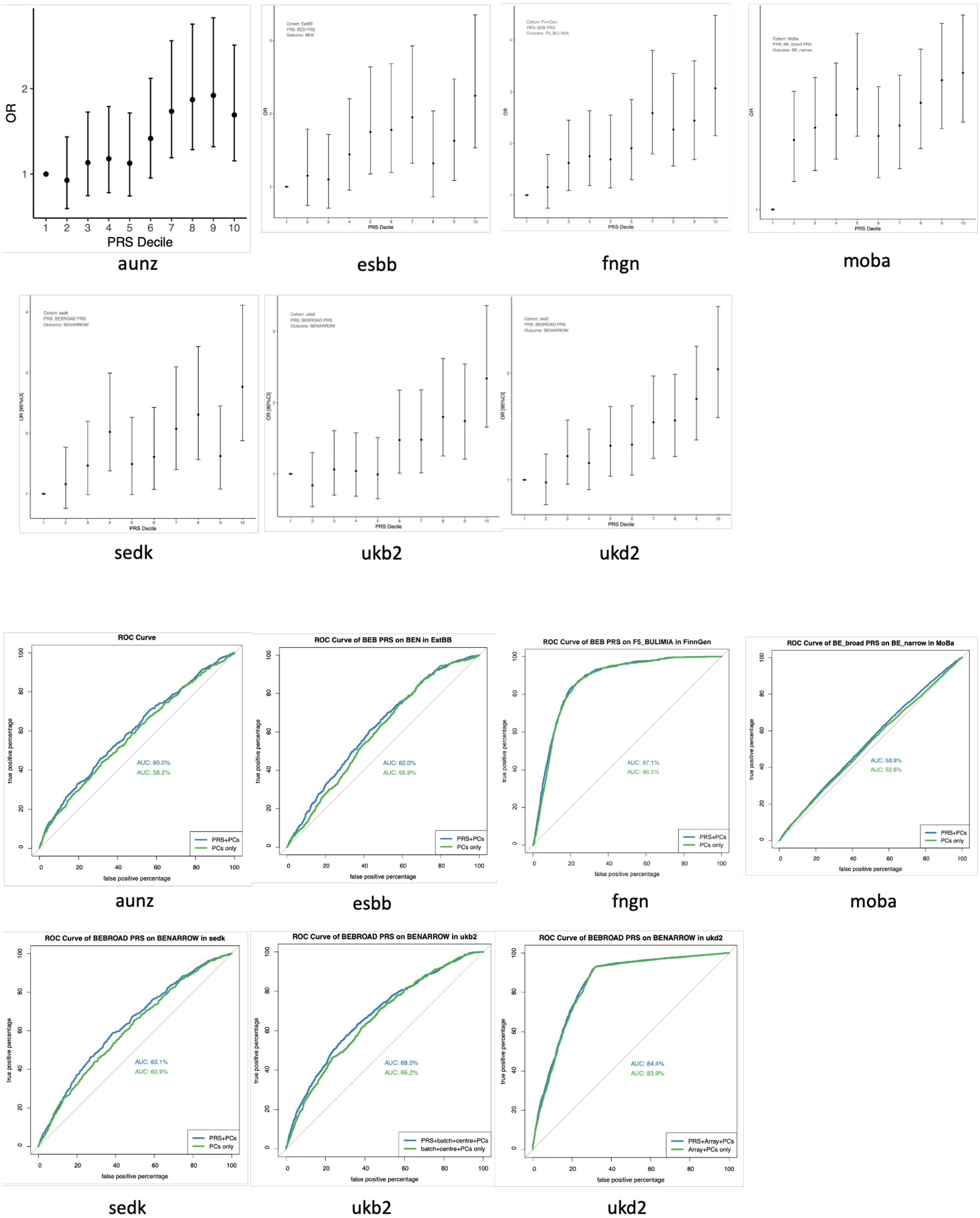

Supplementary Figure 23d: Decile plots and AU-ROC curve for BE-BROAD PRS with AN

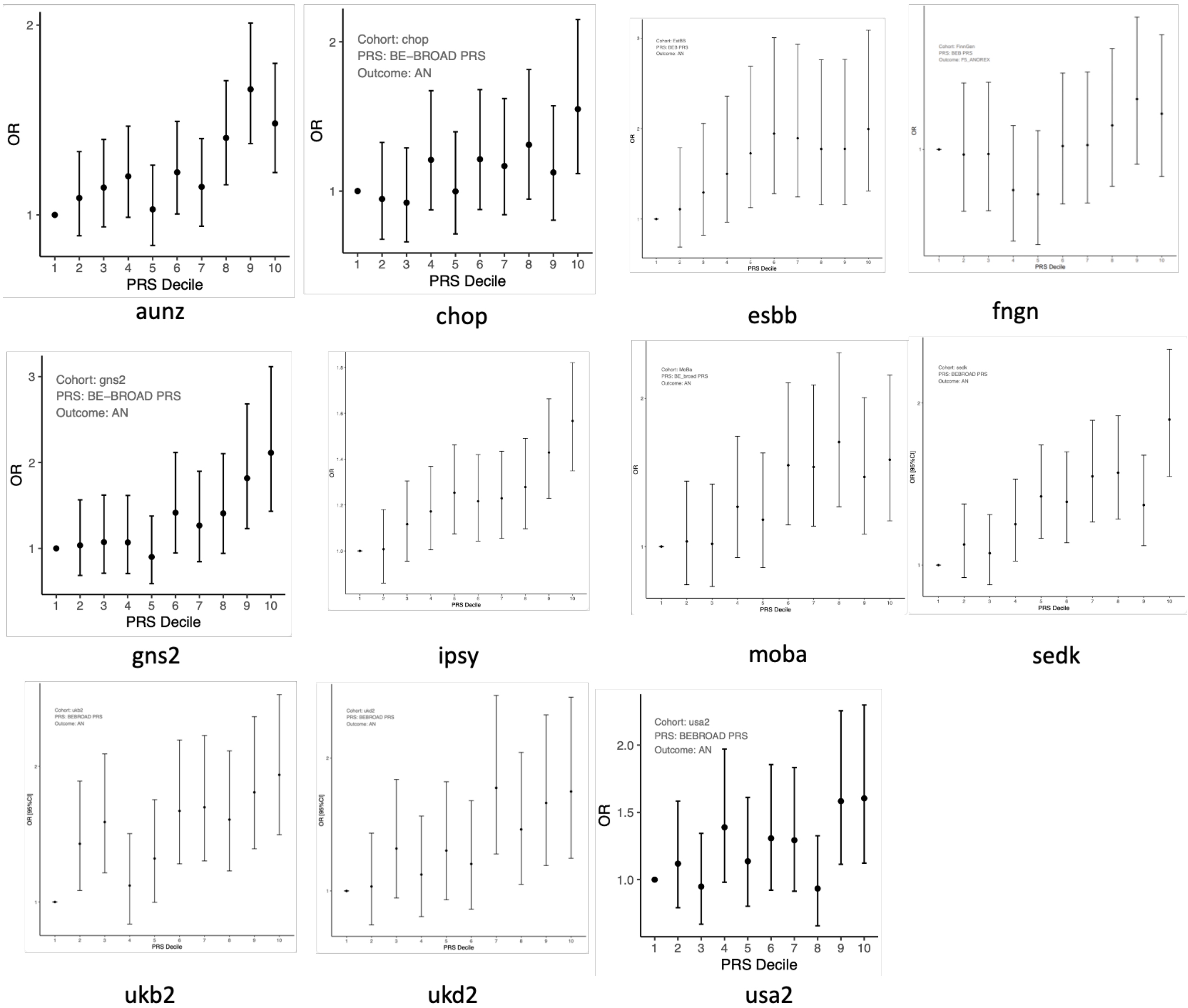

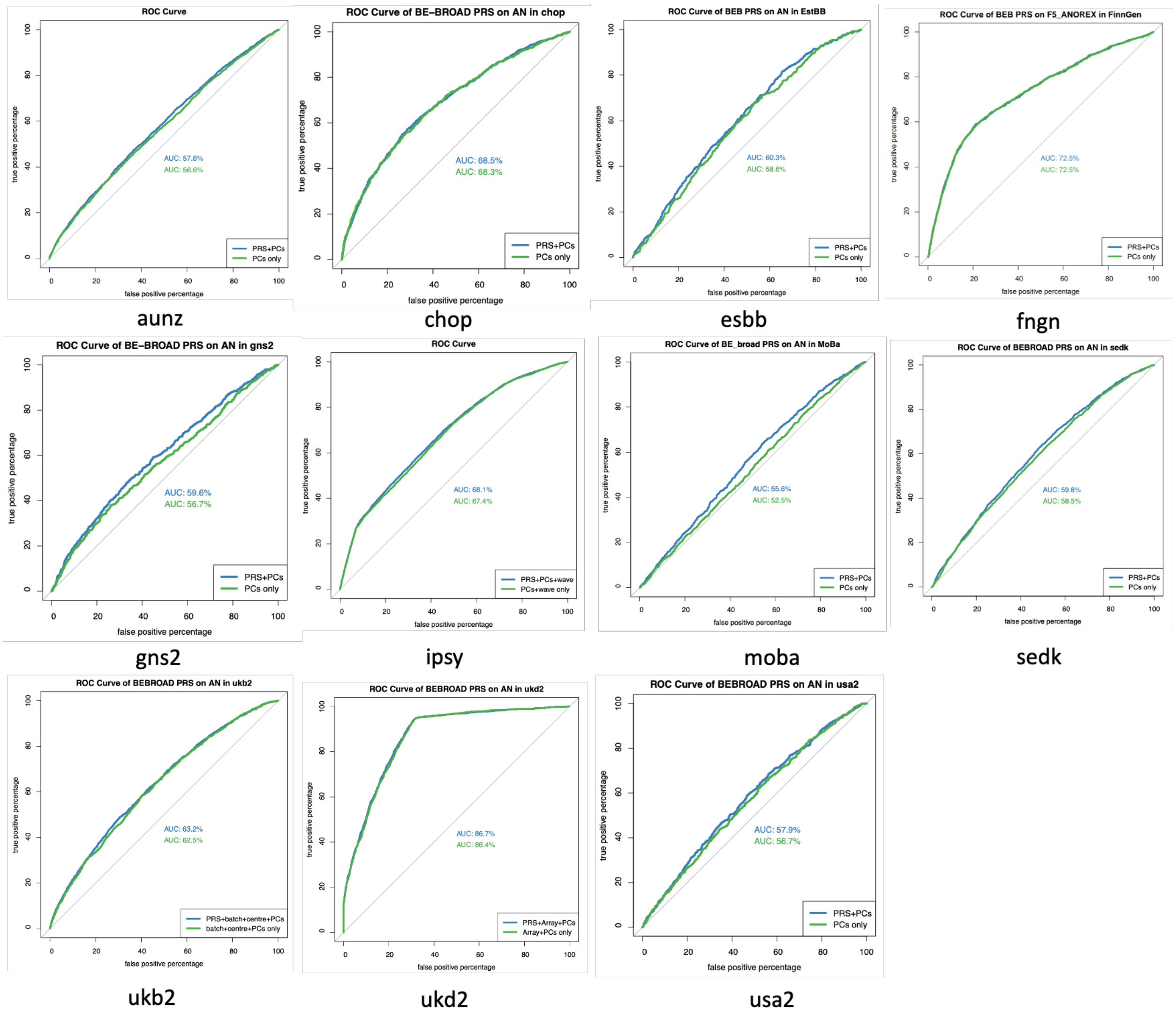

ROC Curve
AUC: 57.6%
AUC: 56.6%
PRS+PCs
PCs only
aunz
ROC Curve of BE–BROAD PRS on AN in chop
AUC: 68.5%
AUC: 68.3%
PRS+PCs
PCs only
chop
ROC Curve of BEB PRS on AN in EstBB
AUC: 60.3%
AUC: 58.6%
PRS+PCs
PCs only
esbb
ROC Curve of BEB PRS on F5_ANOREX in FinnGen
AUC: 72.5%
AUC: 72.5%
PRS+PCs
PCs only
fngn
ROC Curve of BE–BROAD PRS on AN in gns2
AUC: 59.6%
AUC: 56.7%
PRS+PCs
PCs only
gns2
ROC Curve
AUC: 68.1%
AUC: 67.4%
PRS+PCs+wave
PCs+wave only
ipsy
ROC Curve of BE_broad PRS on AN in MoBa
AUC: 55.6%
AUC: 52.5%
PRS+PCs
PCs only
moba
ROC Curve of BEBROAD PRS on AN in sedk
AUC: 59.8%
AUC: 58.5%
PRS+PCs
PCs only
sedk
ROC Curve of BEBROAD PRS on AN in ukb2
AUC: 63.2%
AUC: 62.5%
PRS+batch+centre+PCs
batch+centre+PCs only
ukb2
ROC Curve of BEBROAD PRS on AN in ukd2
AUC: 86.7%
AUC: 86.4%
PRS+Array+PCs
Array+PCs only
ukd2
ROC Curve of BEBROAD PRS on AN in usa2
AUC: 57.9%
AUC: 56.7%
PRS+PCs
PCs only
usa2
true positive percentage
false positive percentage

Supplementary Figure 23e: Decile plots and AU-ROC curve for BE-NARROW PRS with BE-NARROW

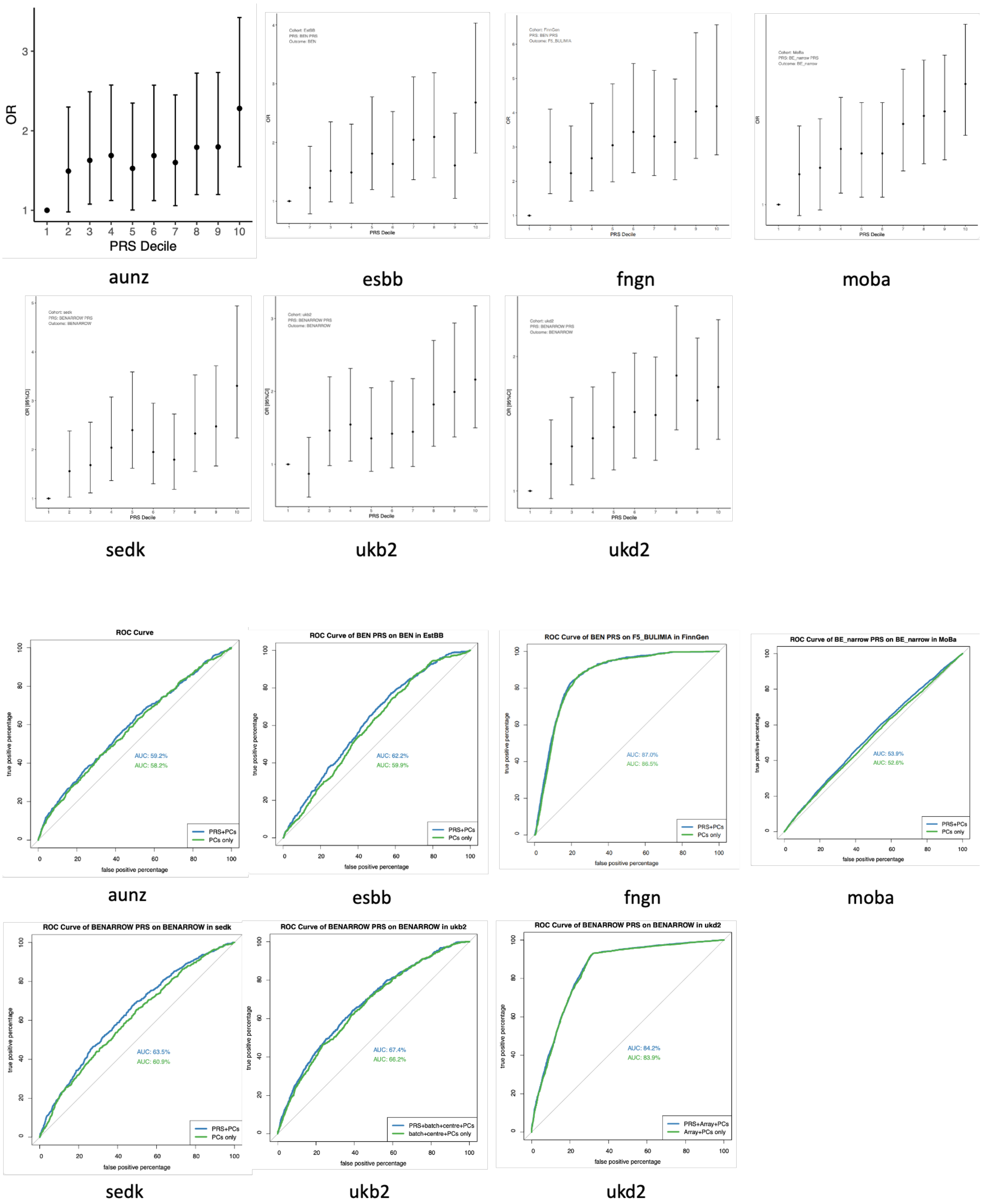

Supplementary Figure 23f: Decile plots and AU-ROC curve for BE-NARROW PRS with BE-BROAD

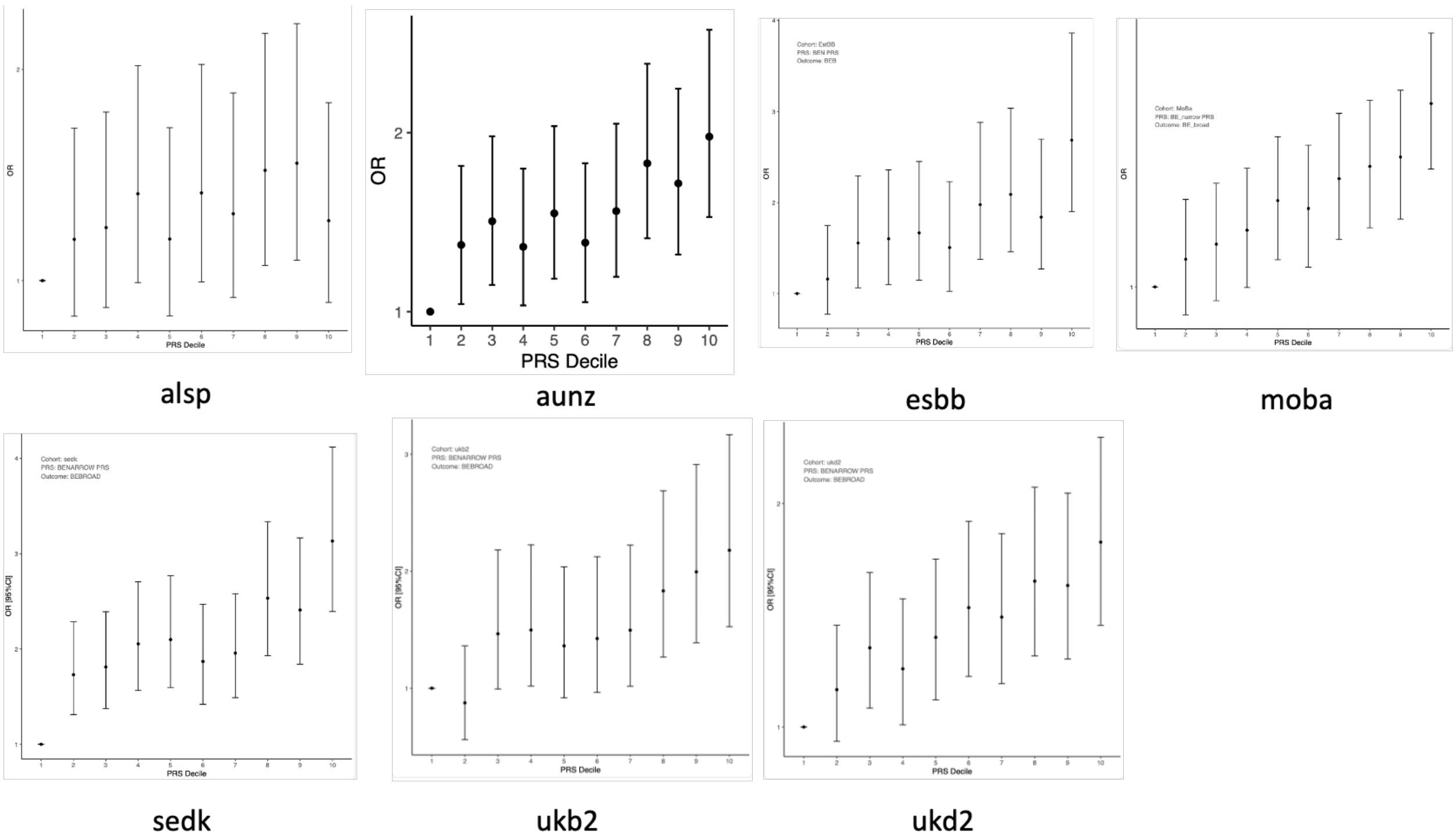

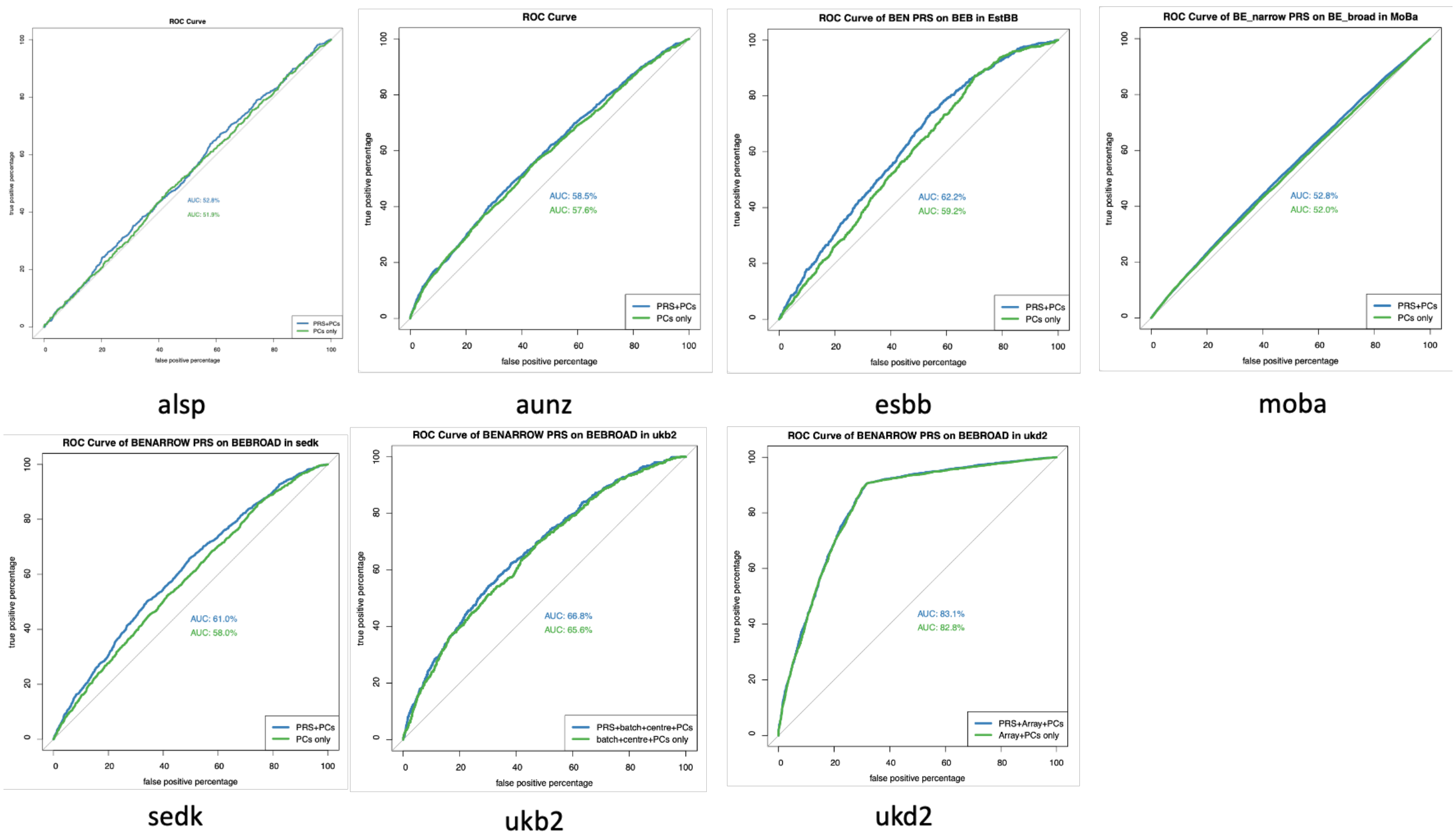

Supplementary Figure 23g: Decile plots and AU-ROC curve for BE-NARROW PRS with AN

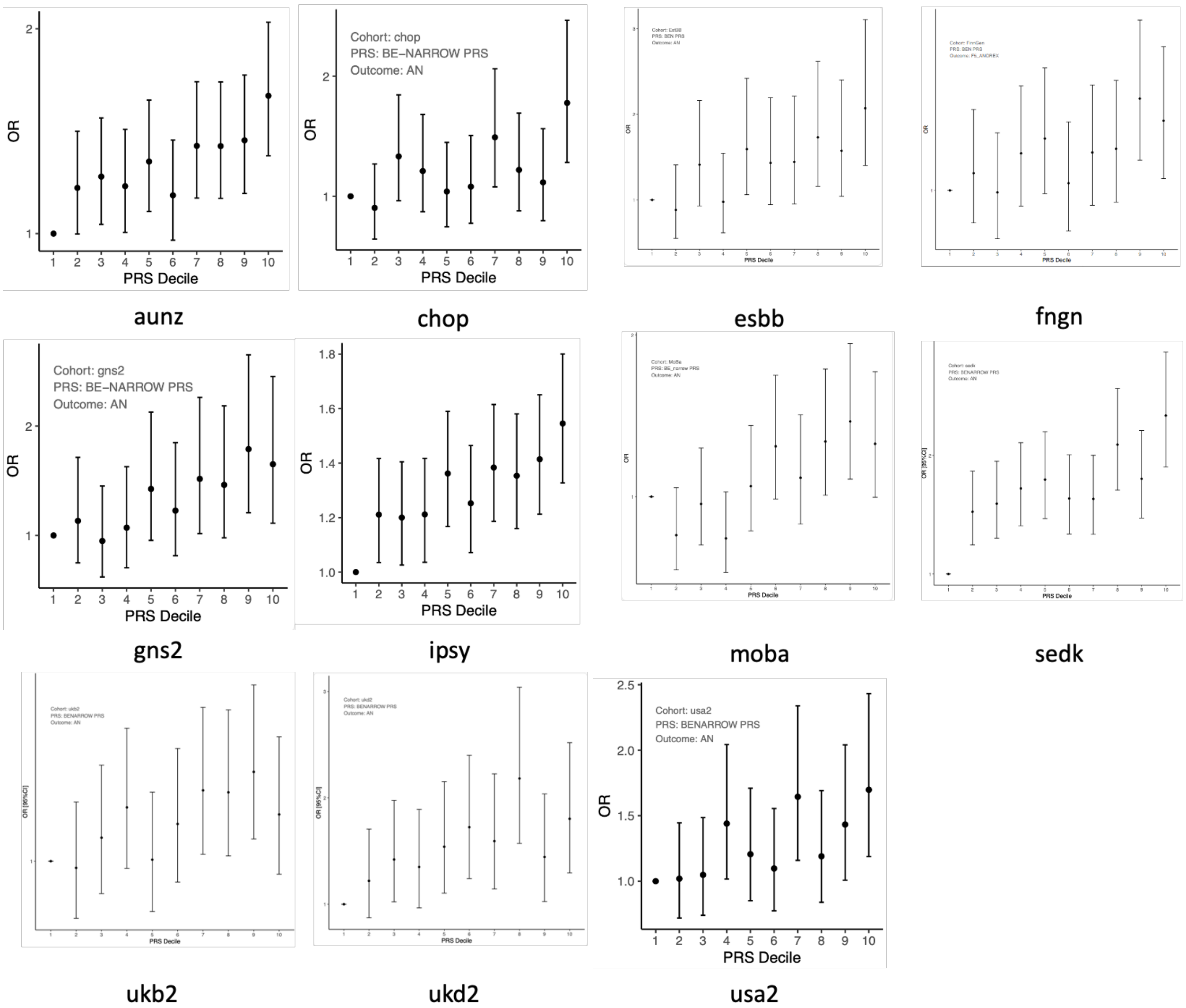

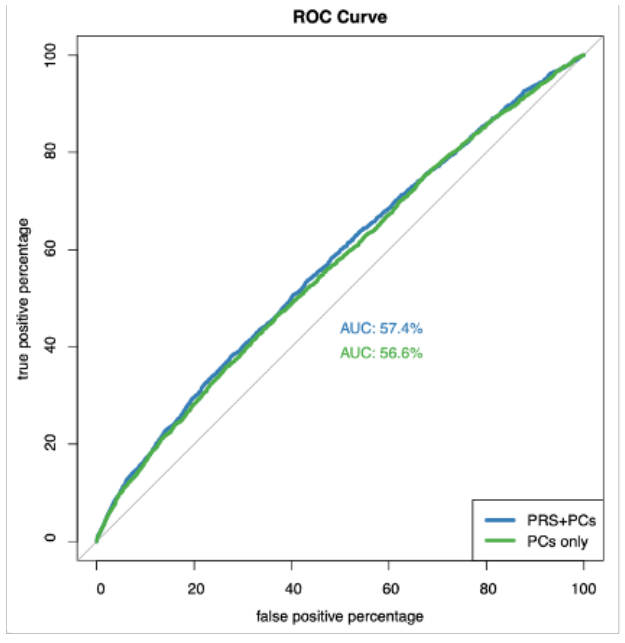

aunz

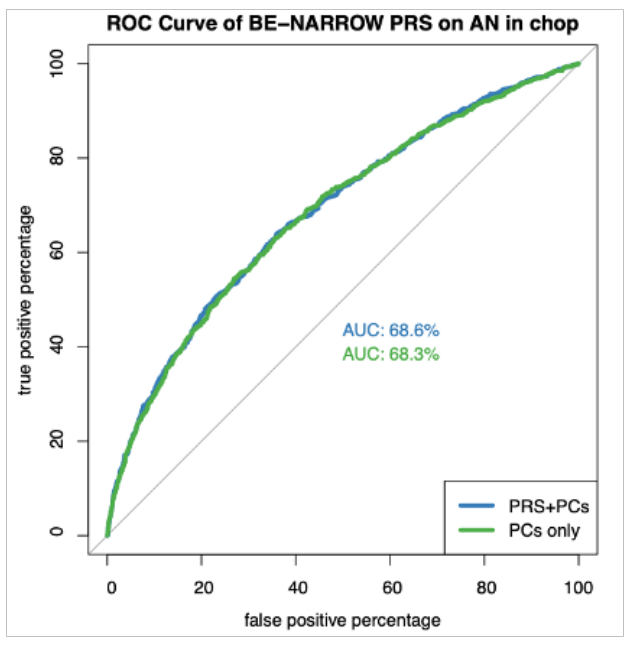

chop

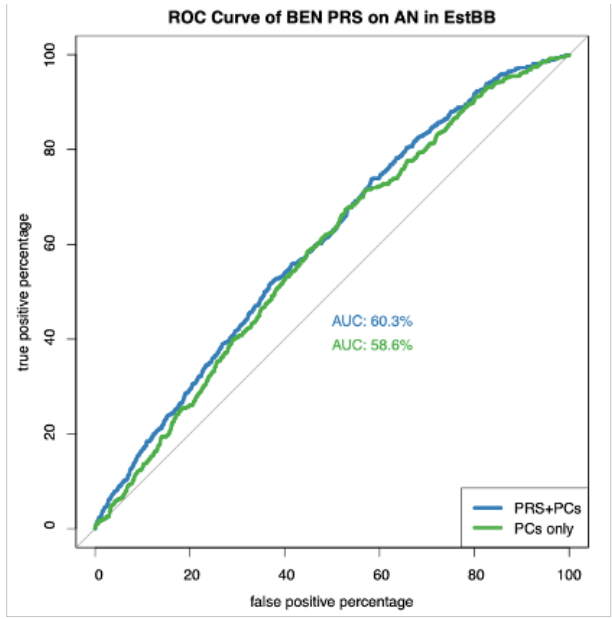

esbb

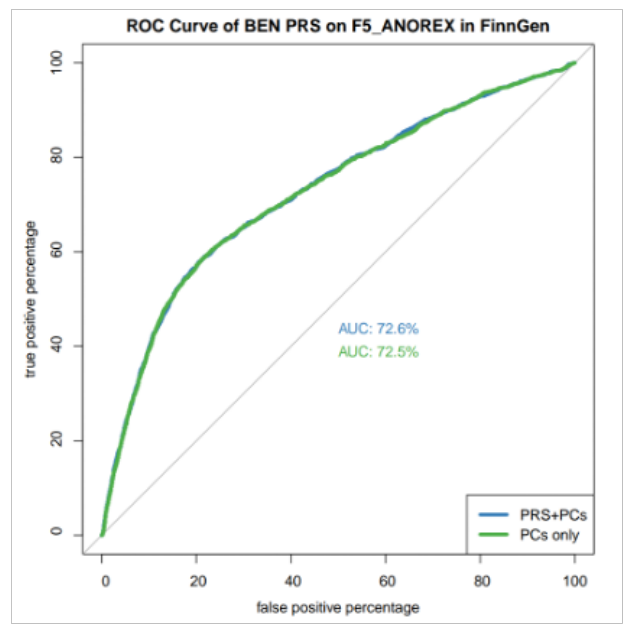

fngn

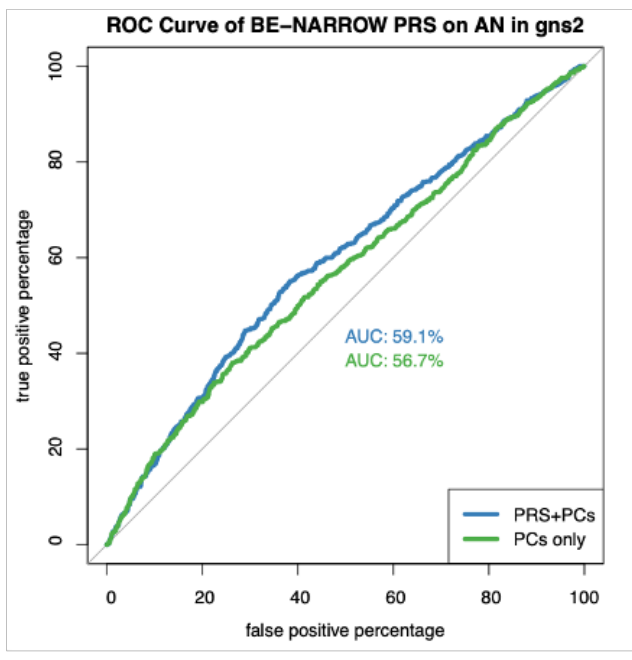

gns2

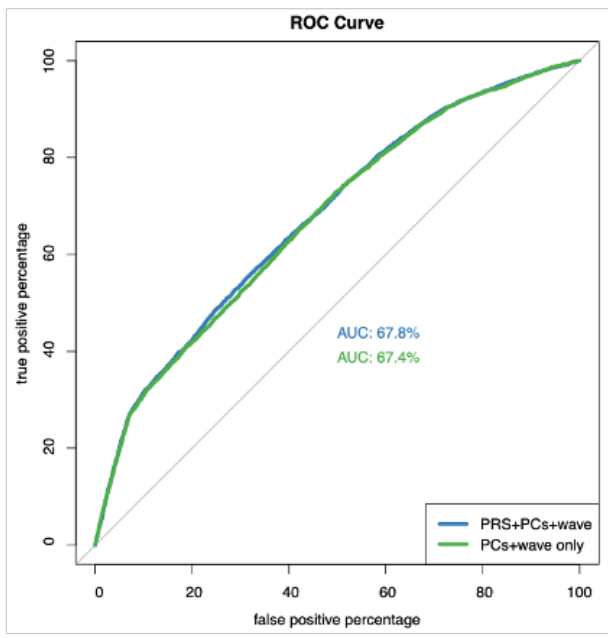

ipsy

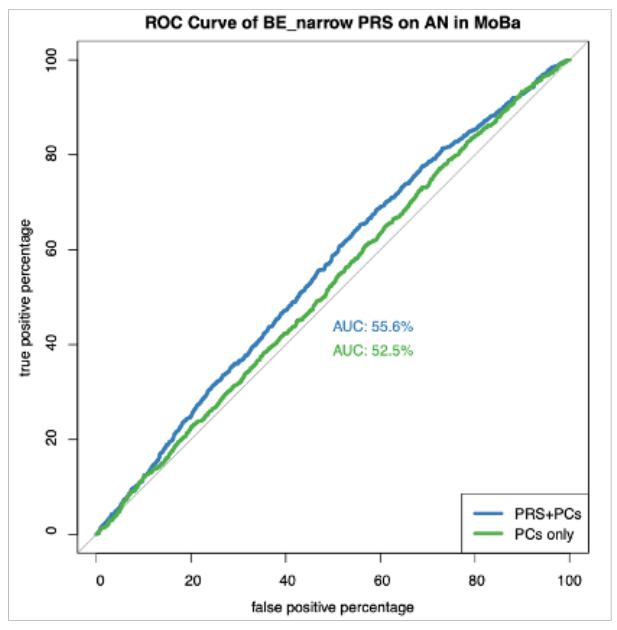

moba

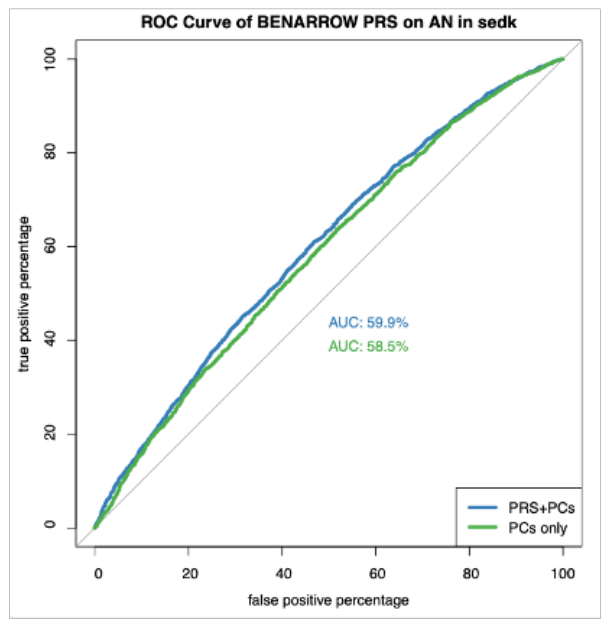

sedk

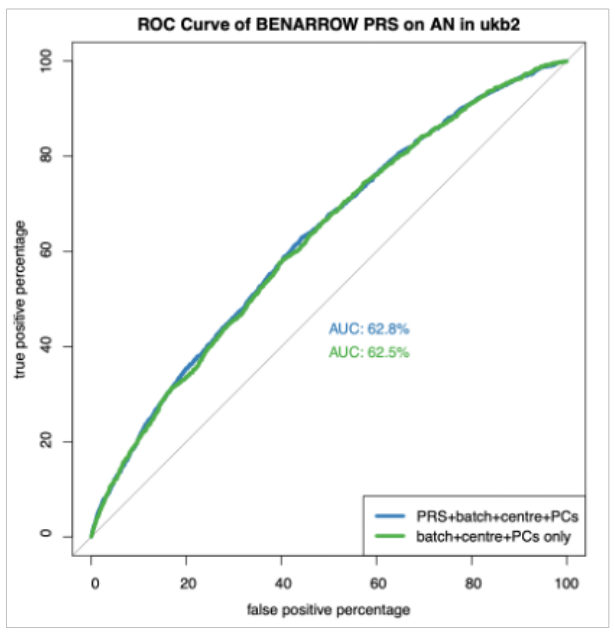

ukb2

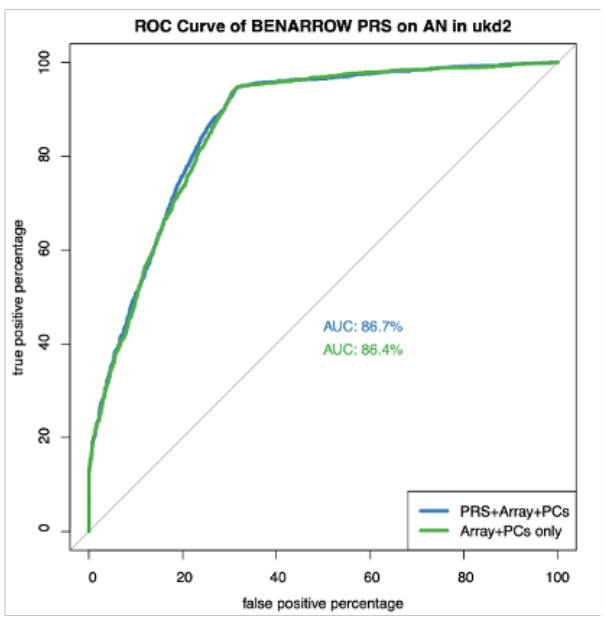

ukd2

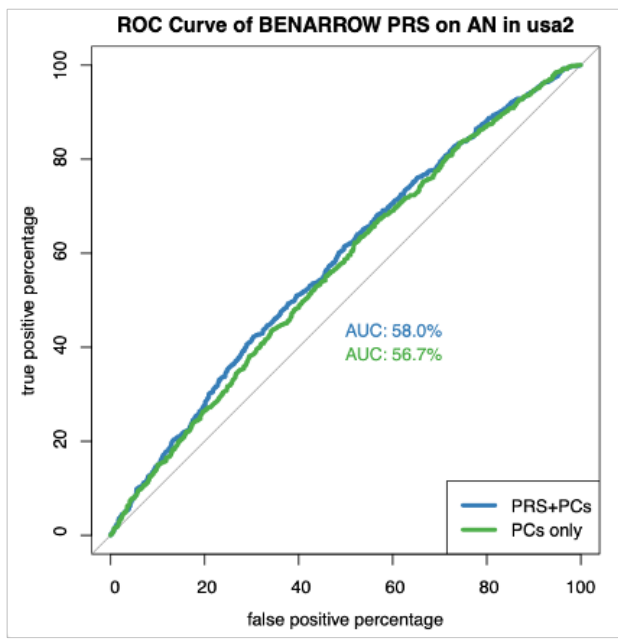

usa2

Supplementary Figure 23h: Decile plots and AU-ROC curve for AN PRS with BE-BROAD

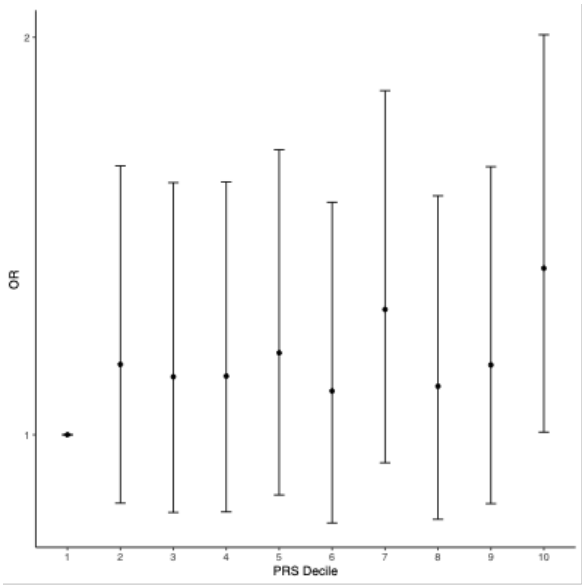

alsp

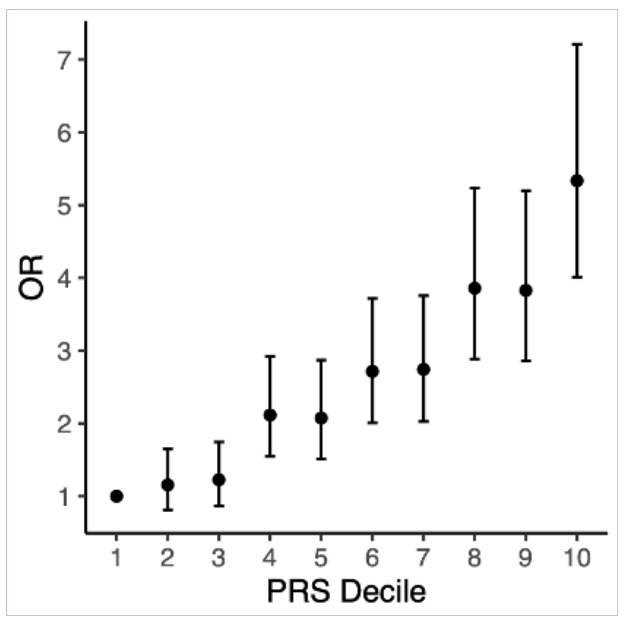

aunz

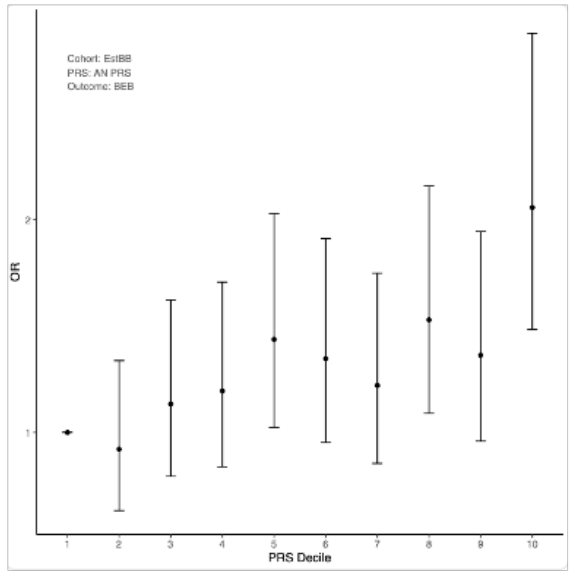

esbb

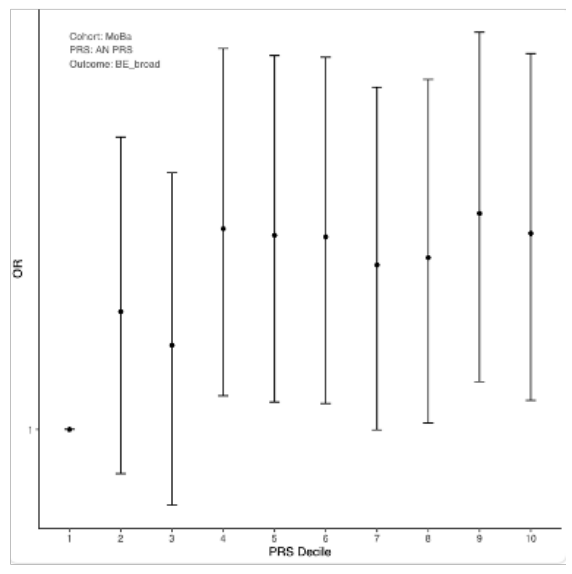

moba

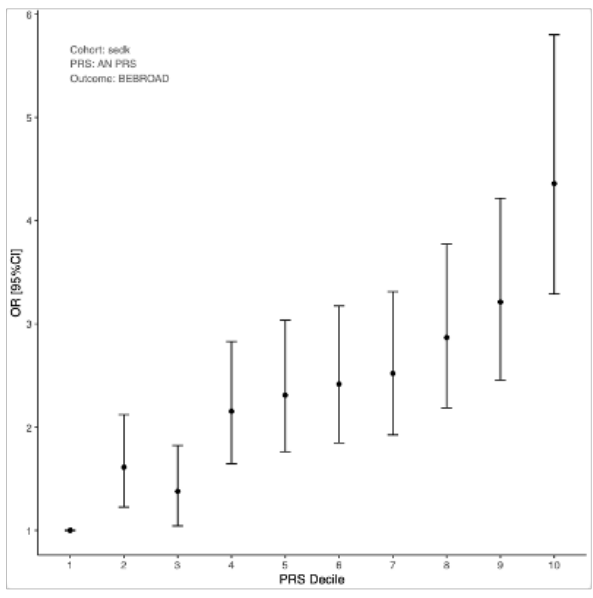

sedk

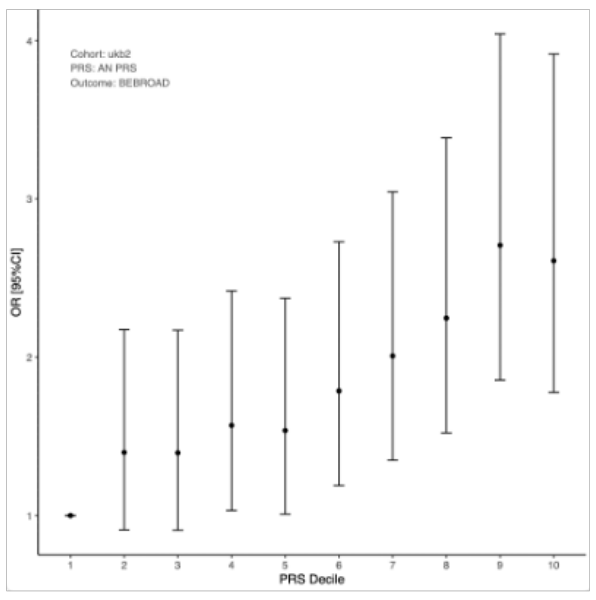

ukb2

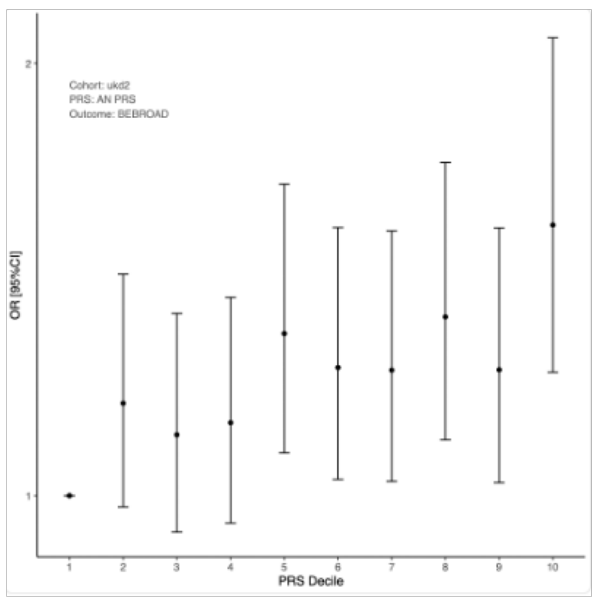

ukd2

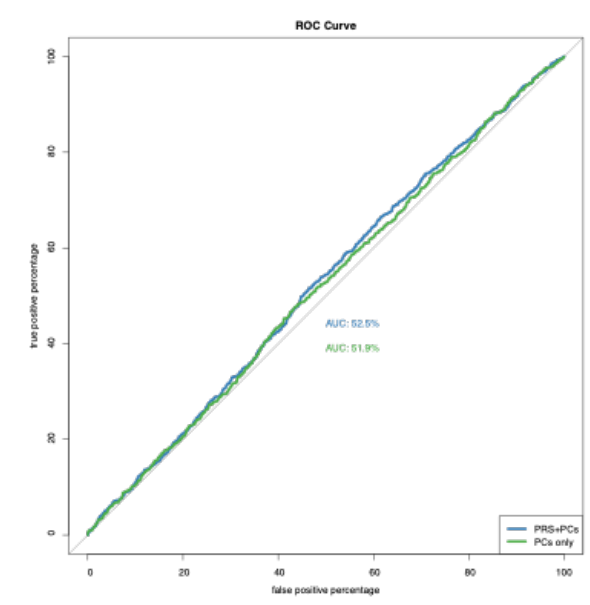

alsp

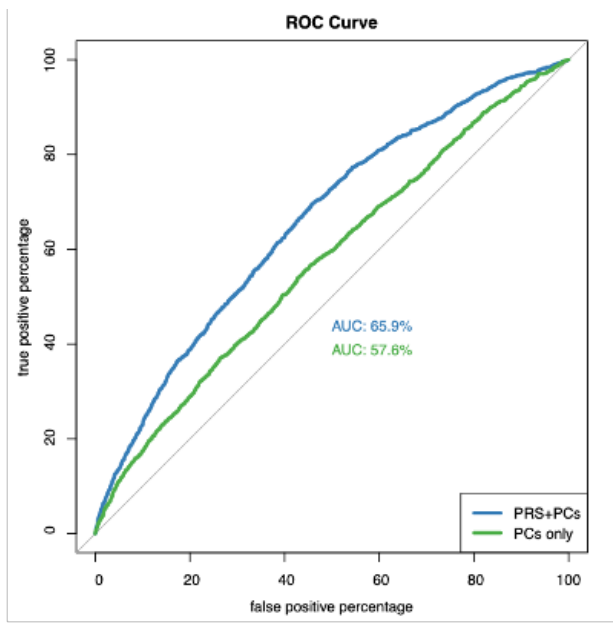

aunz

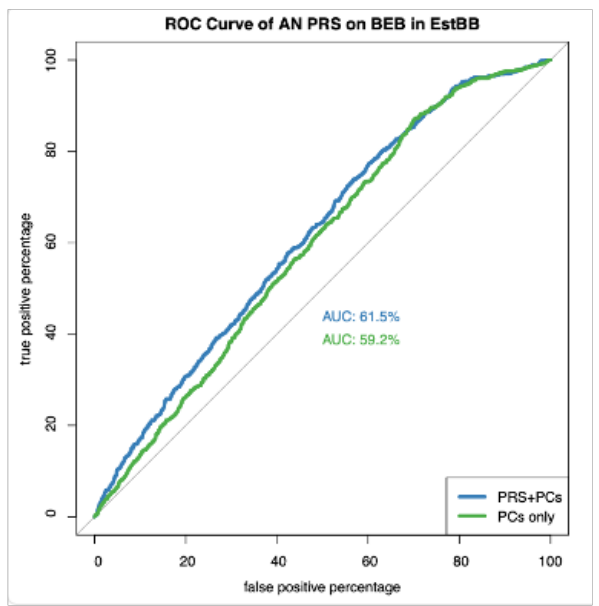

esbb

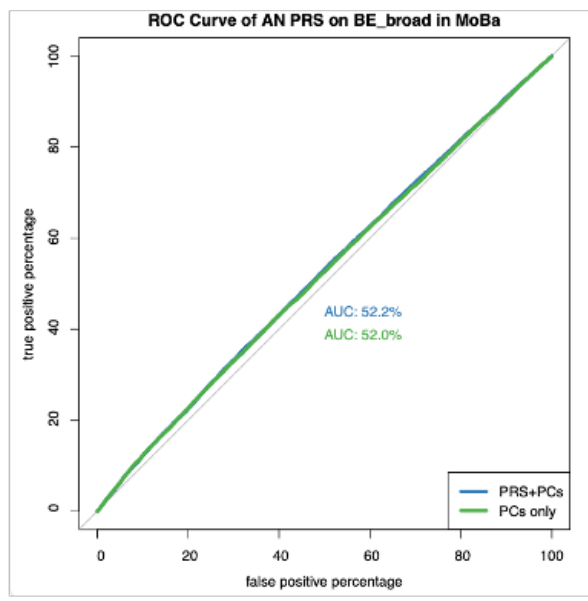

moba

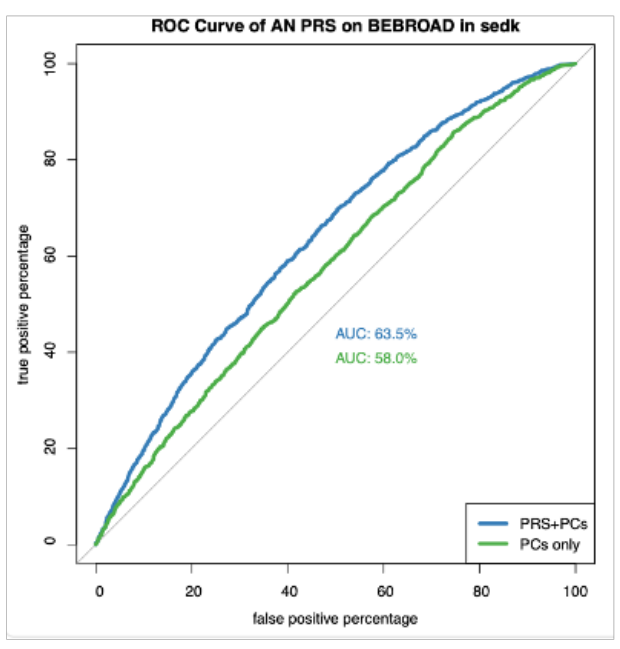

sedk

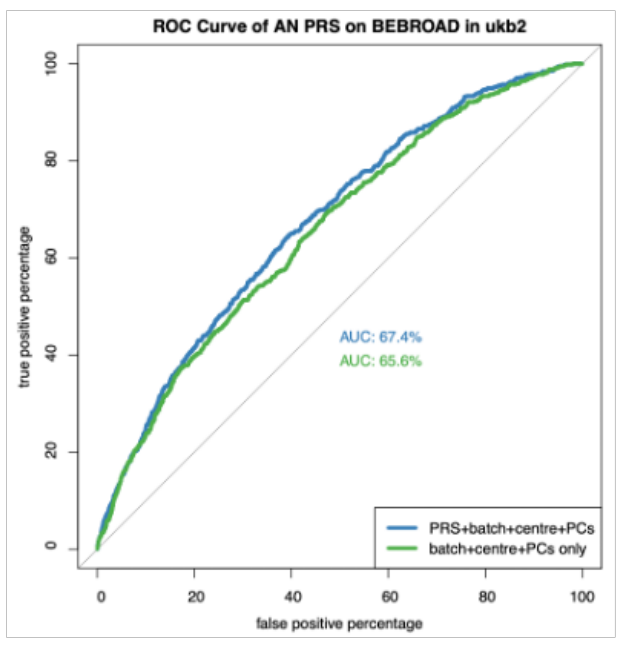

ukb2

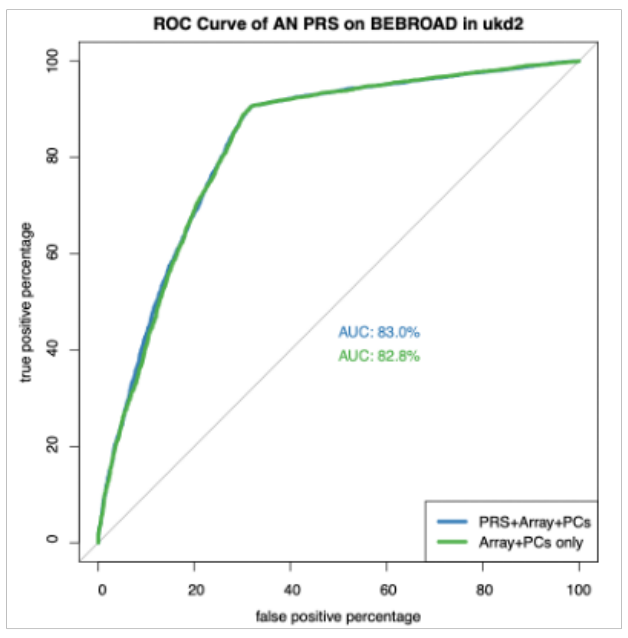

ukd2

Supplementary Figure 23i: Decile plots and AU-ROC curve for AN PRS with BE-NARROW

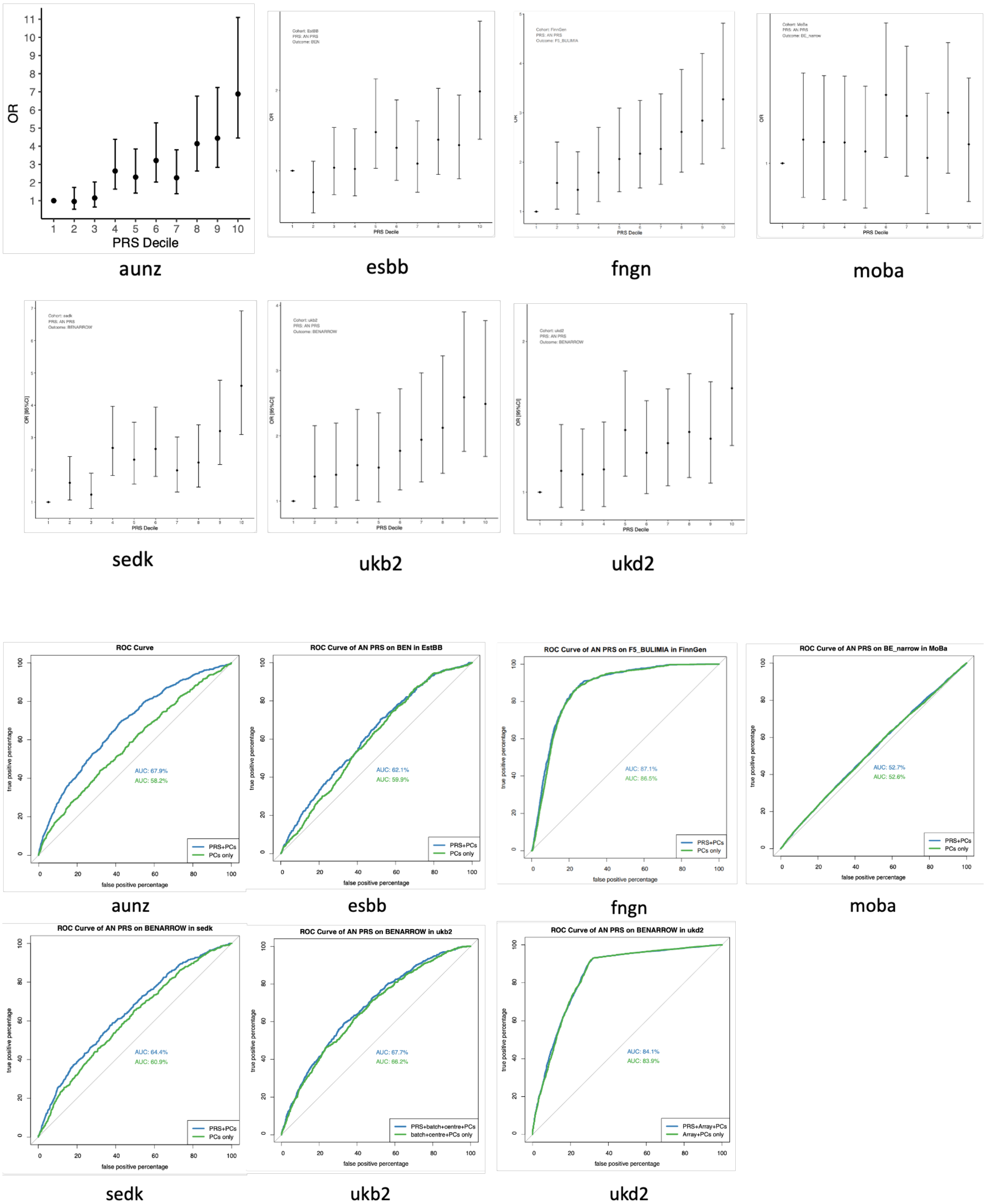

Supplementary Figure 24: Cross-sex polygenic scoring

Decile plots depict the odds ratio (OR) of each decile of female-based polygenic risk scores of BE-BROAD on the BE-BROAD outcome in males, and female-based polygenic risk scores of AN on the AN outcome in males, with the lowest decile serving as the reference group. The receiver operating characteristic (ROC) curve is a graphical illustration of the performance of a regression model to predict the binary outcome (i.e., case/control status of BE-BROAD or AN here). If a regression model has a ROC curve overlapping with the diagonal grey line in the ROC curve plot, it indicates that the regression model is no better than chance to differentiate cases and controls with an area under the curve (AUC) of 0.5. An AUC greater than 0.5 suggests that the prediction performance of a model is better than chance and an AUC of 1 indicates perfect performance. In each ROC curve plot, ROC curves of two models are plotted: one for the baseline model (e.g., genetic principal components (PCs) as predictor variables) and one for the PRS model (i.e., both PRS and baseline predictors are included as predictor variables). The precision-recall (PR) curve is another graphical plot to examine predictive performance and performs more accurate evaluation for imbalance datasets. In contrast to the ROC curve which plots sensitivity (true positives detected/all true positives) and 1- specificity (false positives detected/all true negatives), the PR curve plots precision (true positives detected/all detected positives) and recall (same as sensitivity). PR curves are also plotted for both baseline and PRS models, with a greater PR AUC value indicating better predictive performance.

Supplementary Figure 24a: Decile plots, AU-ROC curve, and area under the precision-recall (AU-PR) curve for female BE-BROAD PRS with BE-BROAD in males

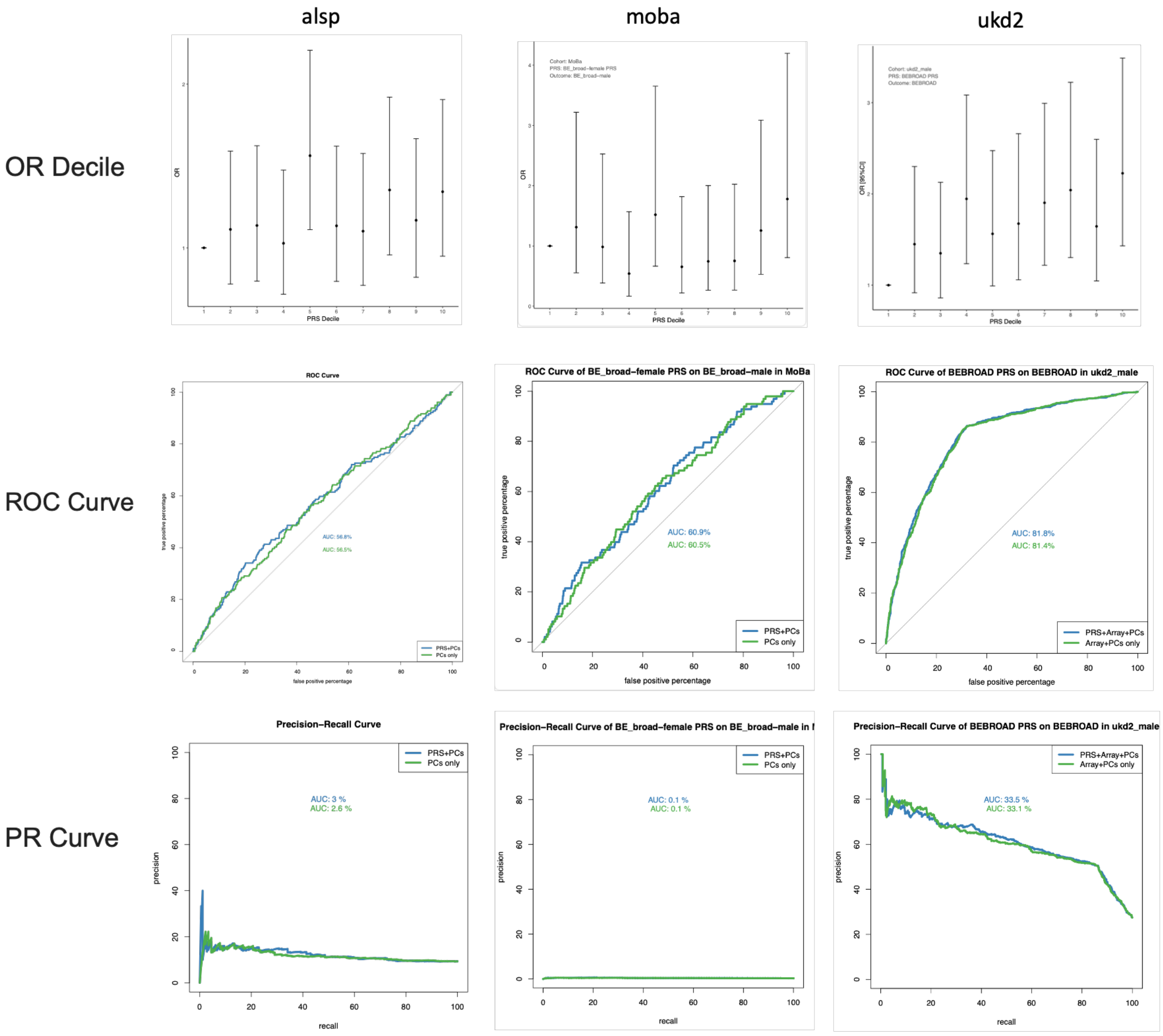

Supplementary Figure 24b: Decile plots, AU-ROC curve, and AU-PR curve female AN PRS with AN in males

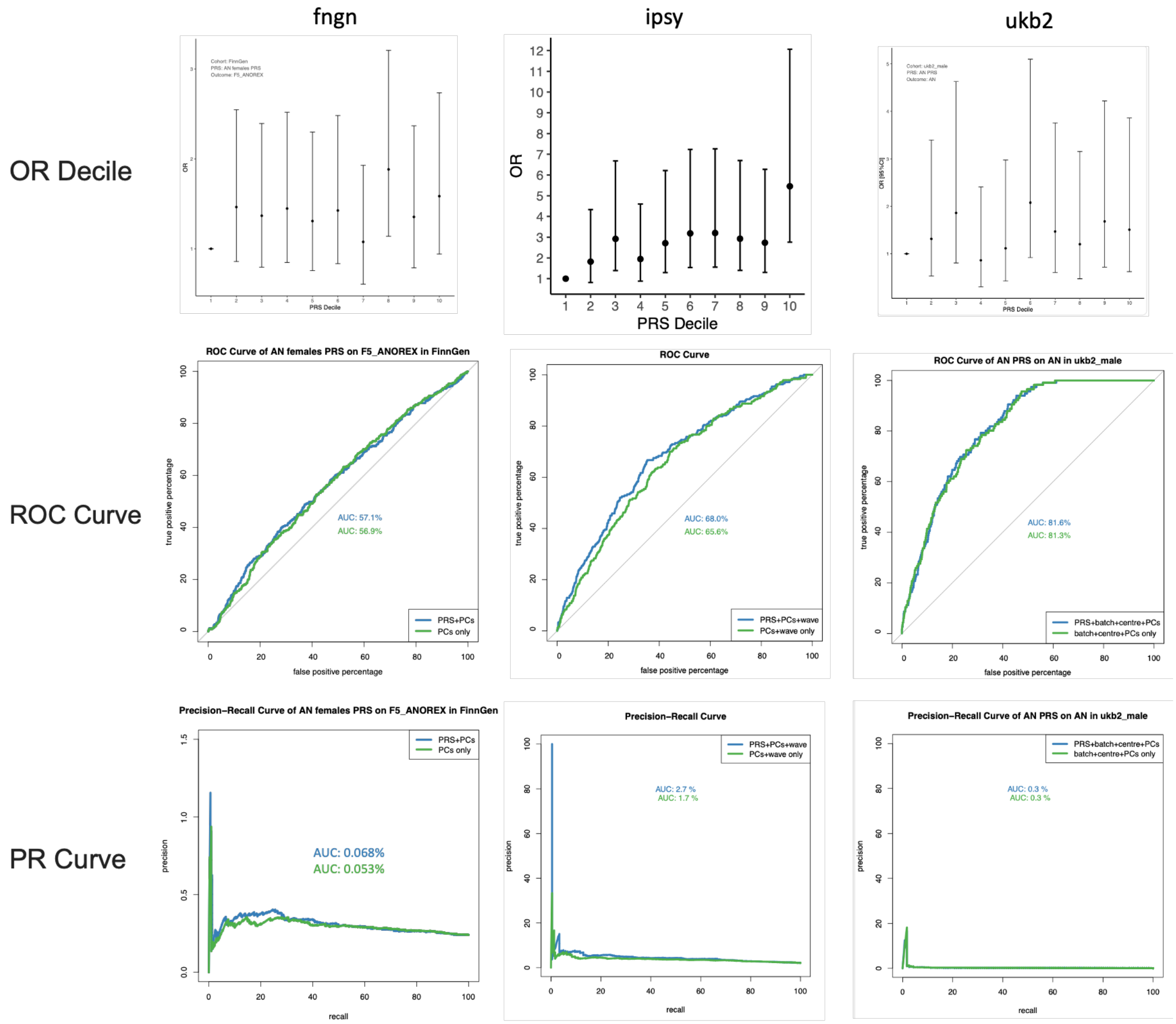

Supplementary Figure 25: Polygenic scoring across comorbid AN and BE-BROAD, BE-BROAD-only, and AN-only subgroups

The plots depict PRS differences (i.e., beta values) for AN PRS, BE-BROAD PRS, and BE-NARROW PRS between each subgroup and the control group across four study cohorts (aunz, sedk, ukb2, ukd2), adjusting for genetic principal components (PCs). Overall, the comorbid (AN_BEB) and AN-only groups exhibit similar high average AN PRS, with the AN-only group having the lowest BE PRSs. The BE-BROAD-only group has the lowest AN PRS but similarly high BE PRSs as the AN_BEB group.

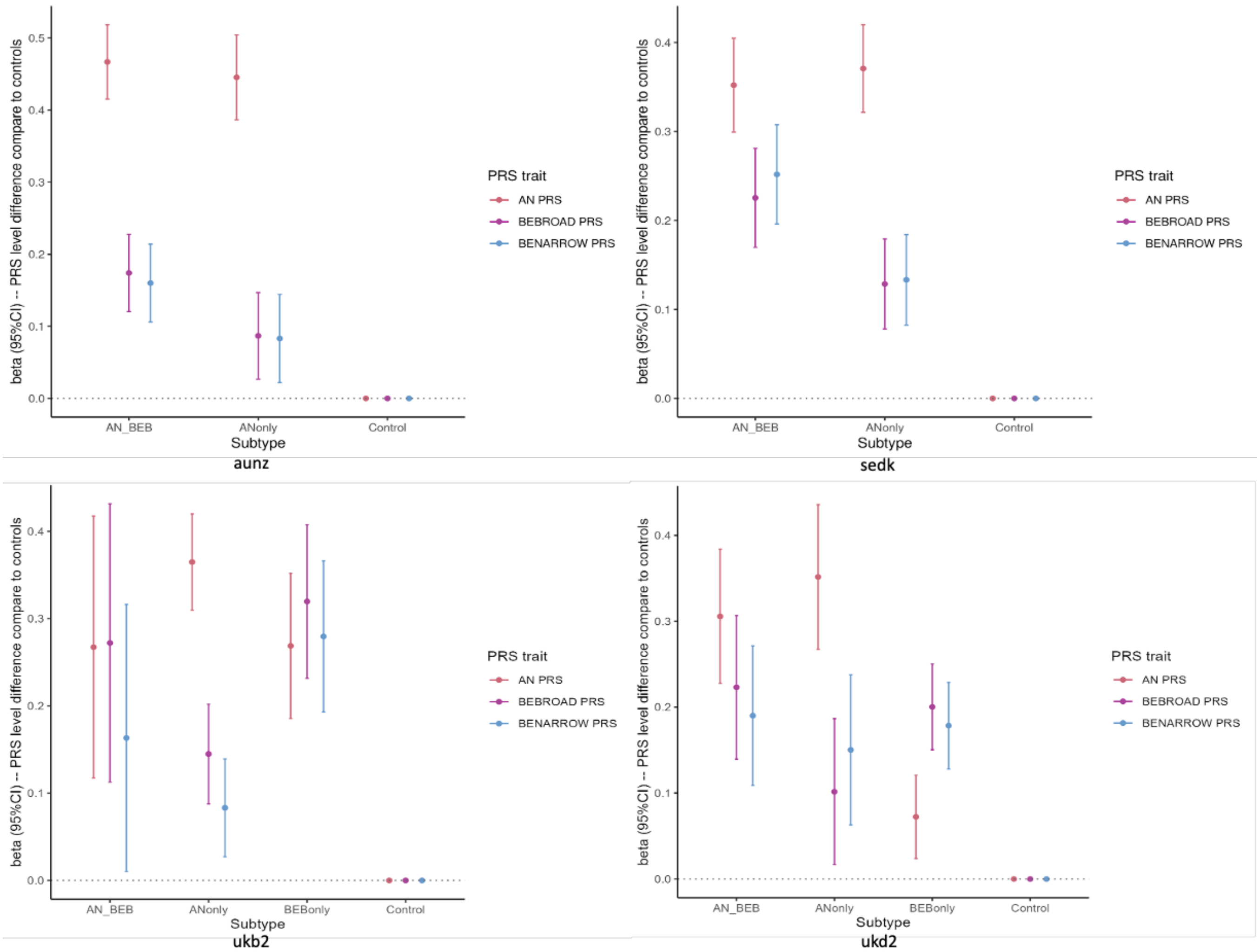

Supplementary Figure 26: Graphic representation of the GWAS-by-subtraction model

Circles represent the latent (anthropometric and non-anthropometric) genetic components of body mass index and binge eating. Squares represent the observed SNP estimates obtained from the GWAS summary statistics of body mass index and binge eating (broadly defined; BE-BROAD). Single-headed arrows indicate linear regressions pointing from the independent variable to the dependent variable, and double-headed arrows indicate variance or covariance relationships.

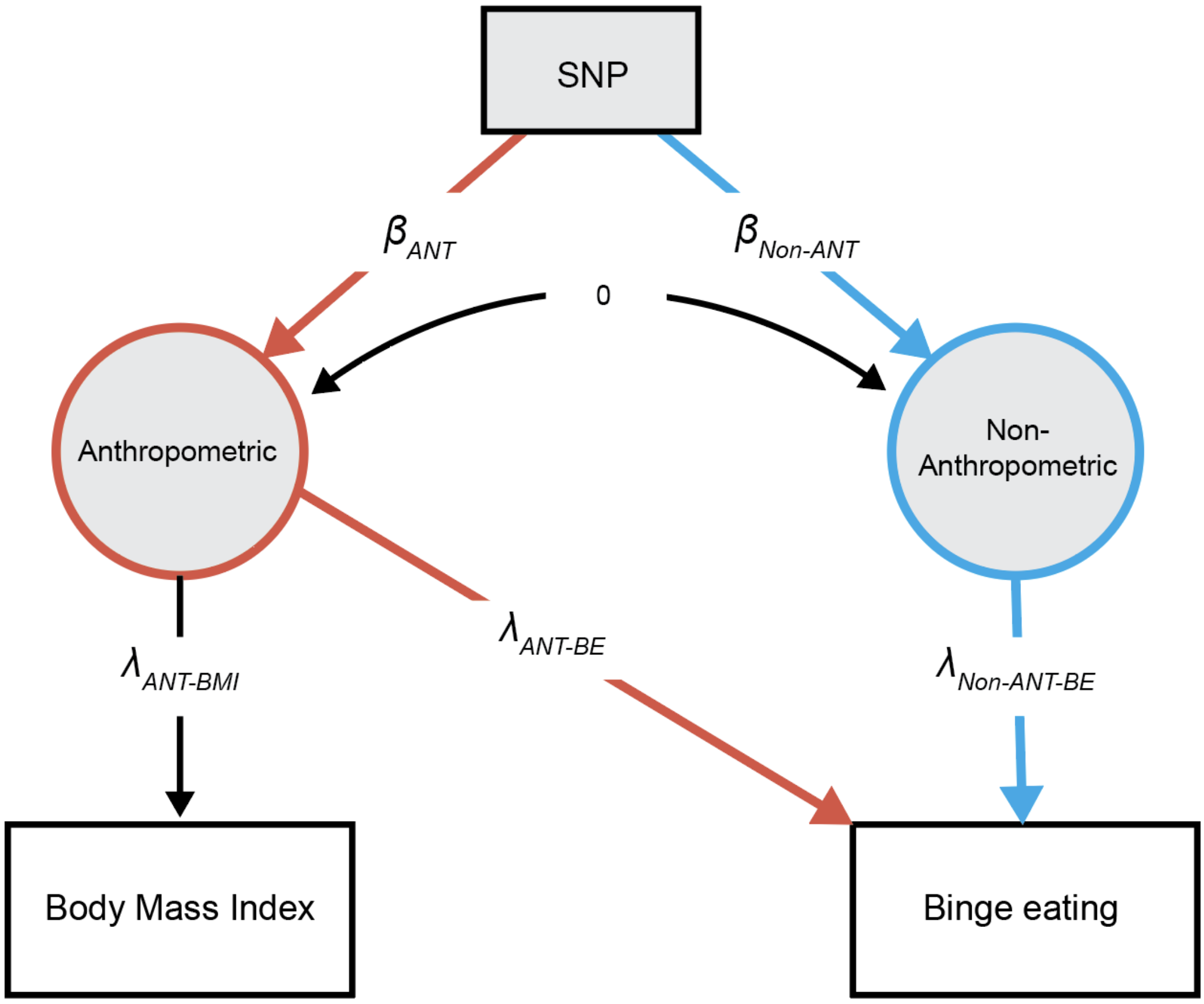

Supplementary Figure 27: Forest and scatter plots from Mendelian randomisation analyses with BE-BROAD

(a, b) BE-BROAD acting on BMI; (c, d) *NonBMI* BE-BROAD acting on BMI; (e, f) BMI acting on BE-BROAD; (g, h) BMI acting on *NonBMI* BE-BROAD. Plots a, c, e, and g show the effect size (beta) and standard error of different Mendelian randomisation analyses of a given exposure on a given outcome. Plots b, d, f, and h show the effect (on the beta scale) and standard error of the effect of each variant (dots) on both exposure and outcome, with regression slopes from different Mendelian randomisation analyses.

Supplementary Figure 27a

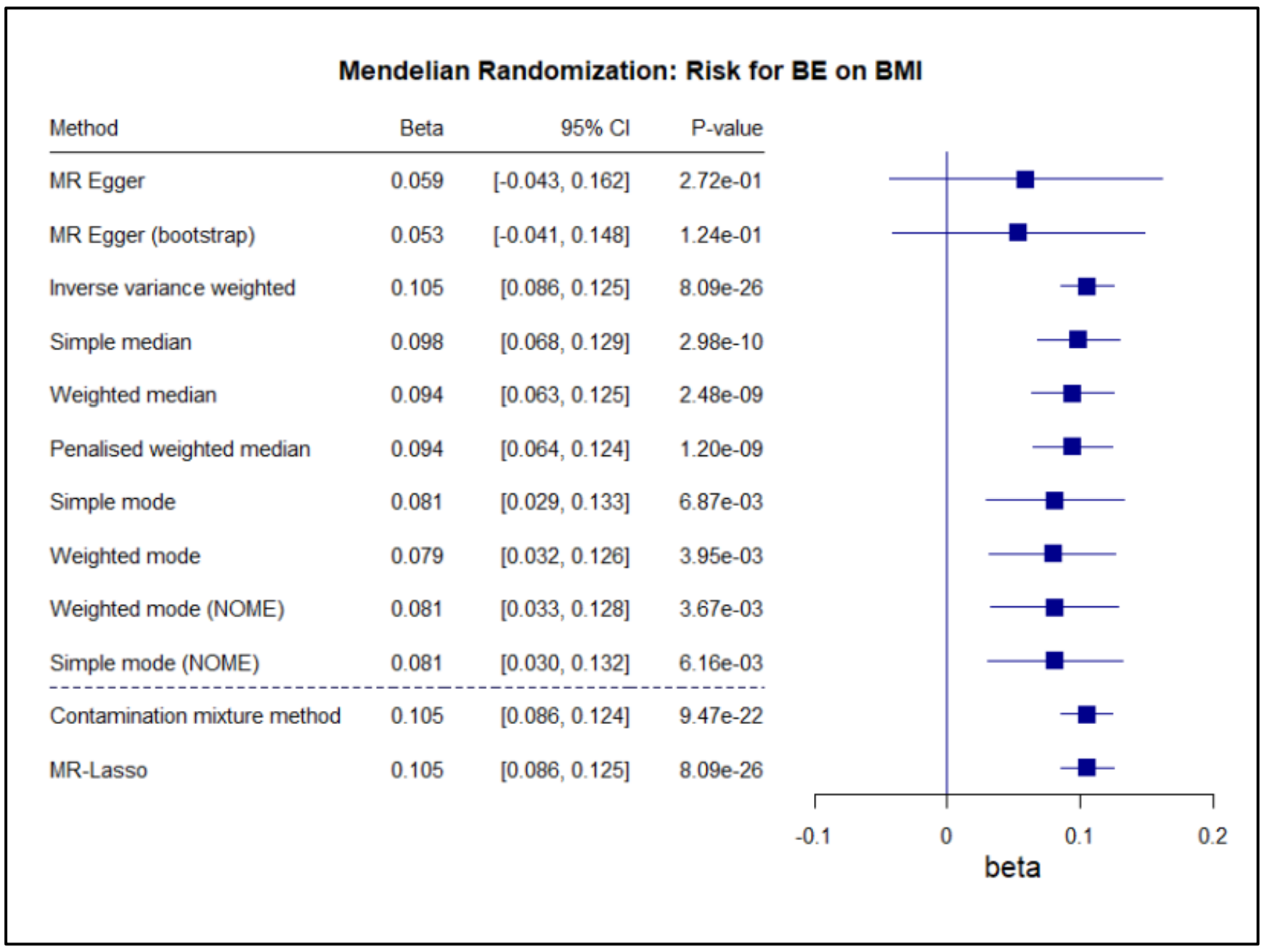

Method	Beta	95% CI	P-value
MR Egger	0.059	[-0.043, 0.162]	2.72e-01
MR Egger (bootstrap)	0.053	[-0.041, 0.148]	1.24e-01
Inverse variance weighted	0.105	[0.086, 0.125]	8.09e-26
Simple median	0.098	[0.068, 0.129]	2.98e-10
Weighted median	0.094	[0.063, 0.125]	2.48e-09
Penalised weighted median	0.094	[0.064, 0.124]	1.20e-09
Simple mode	0.081	[0.029, 0.133]	6.87e-03
Weighted mode	0.079	[0.032, 0.126]	3.95e-03
Weighted mode (NOME)	0.081	[0.033, 0.128]	3.67e-03
Simple mode (NOME)	0.081	[0.030, 0.132]	6.16e-03
Contamination mixture method	0.105	[0.086, 0.124]	9.47e-22
MR-Lasso	0.105	[0.086, 0.125]	8.09e-26

Supplementary Figure 27b

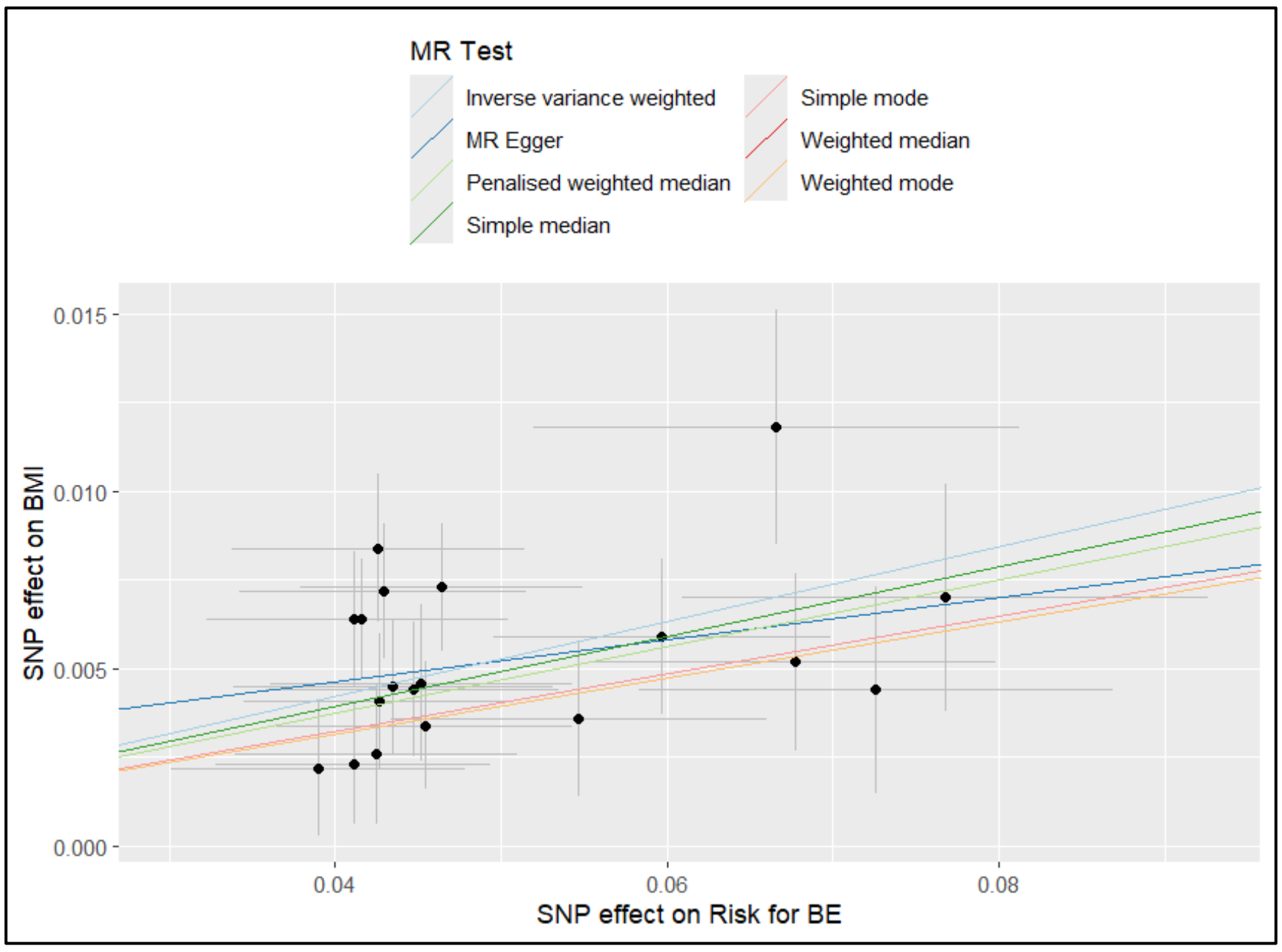

Supplementary Figure 27c

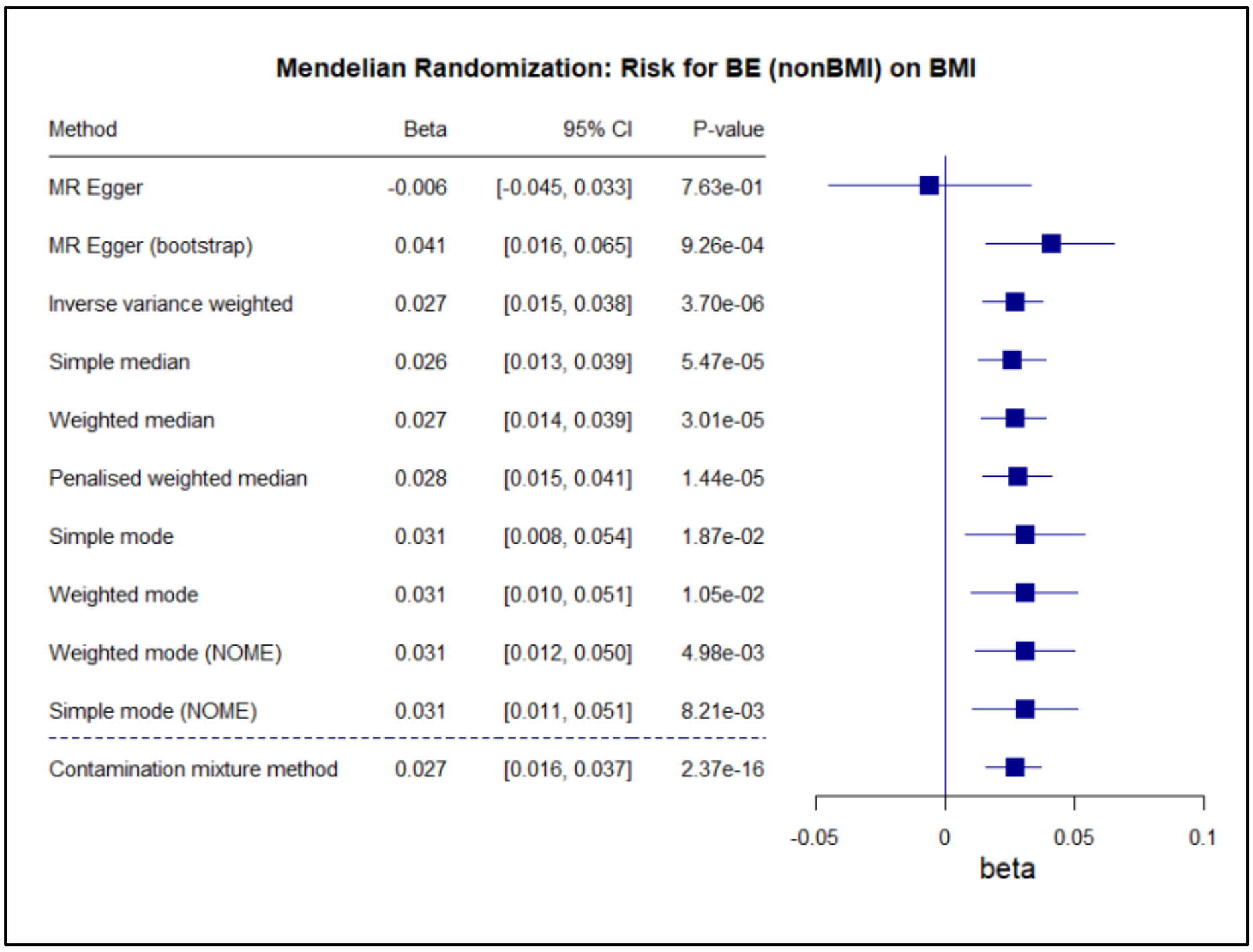

Supplementary Figure 27d

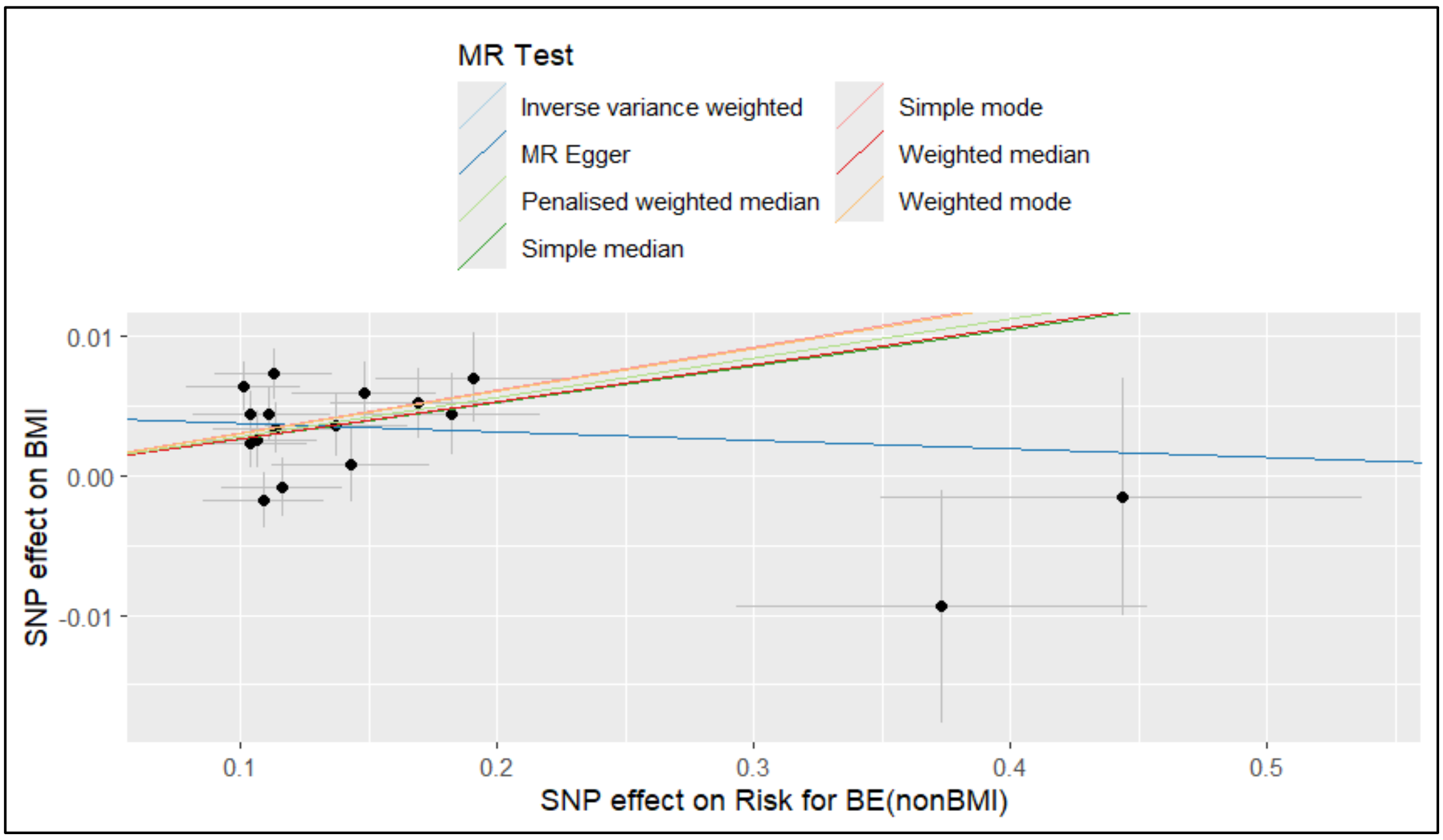

Supplementary Figure 27e

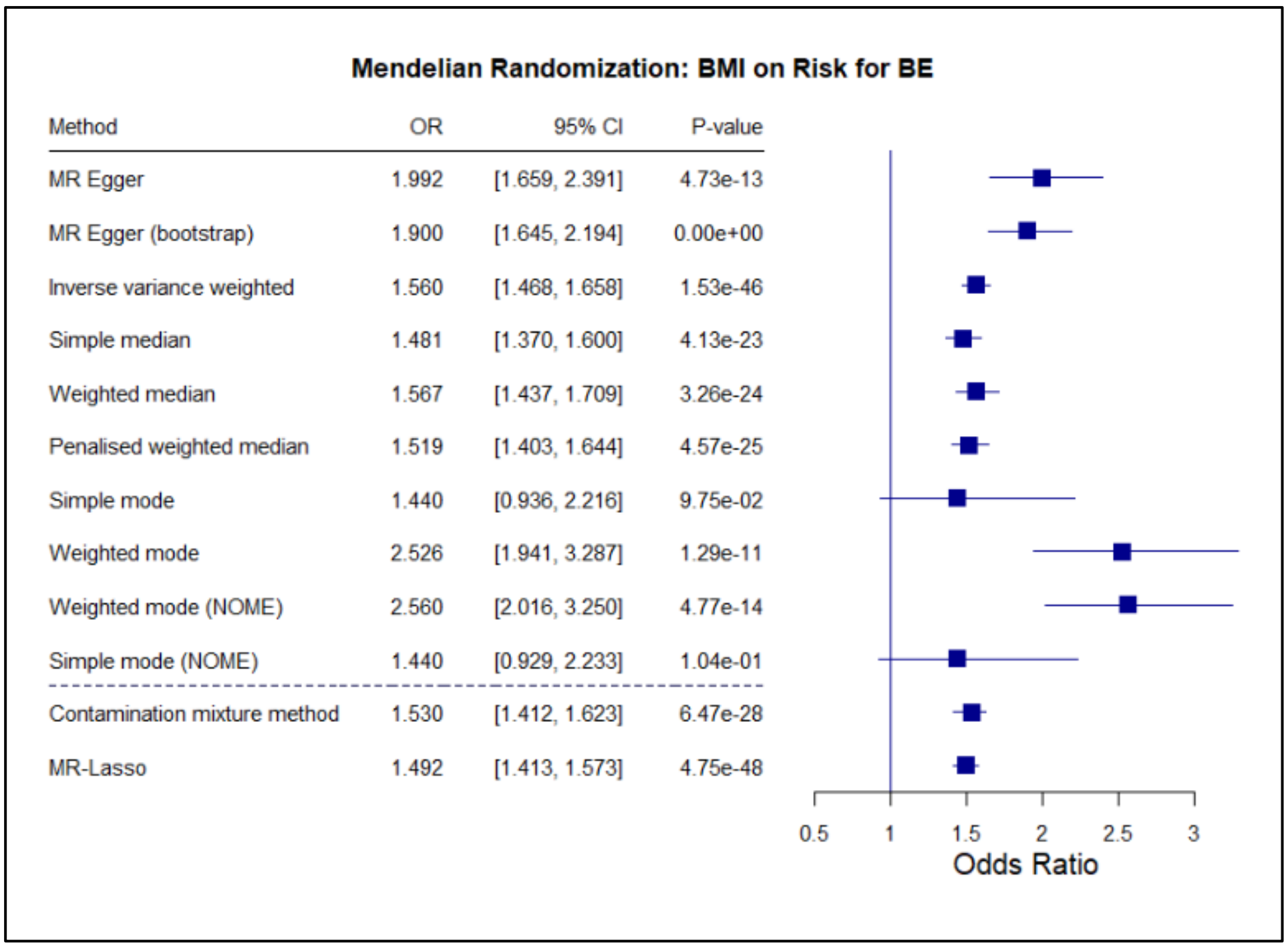

Supplementary Figure 27f

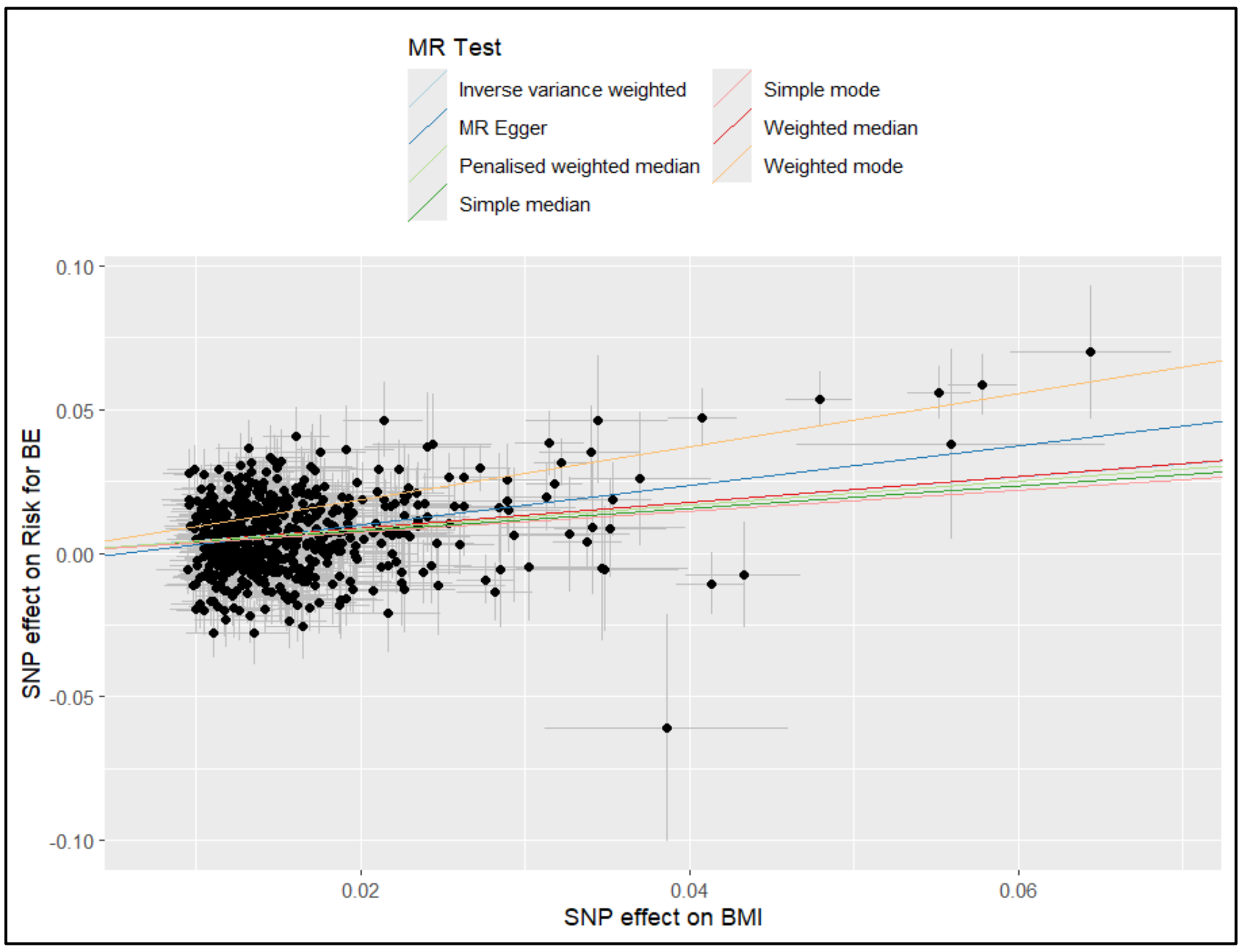

Supplementary Figure 27g

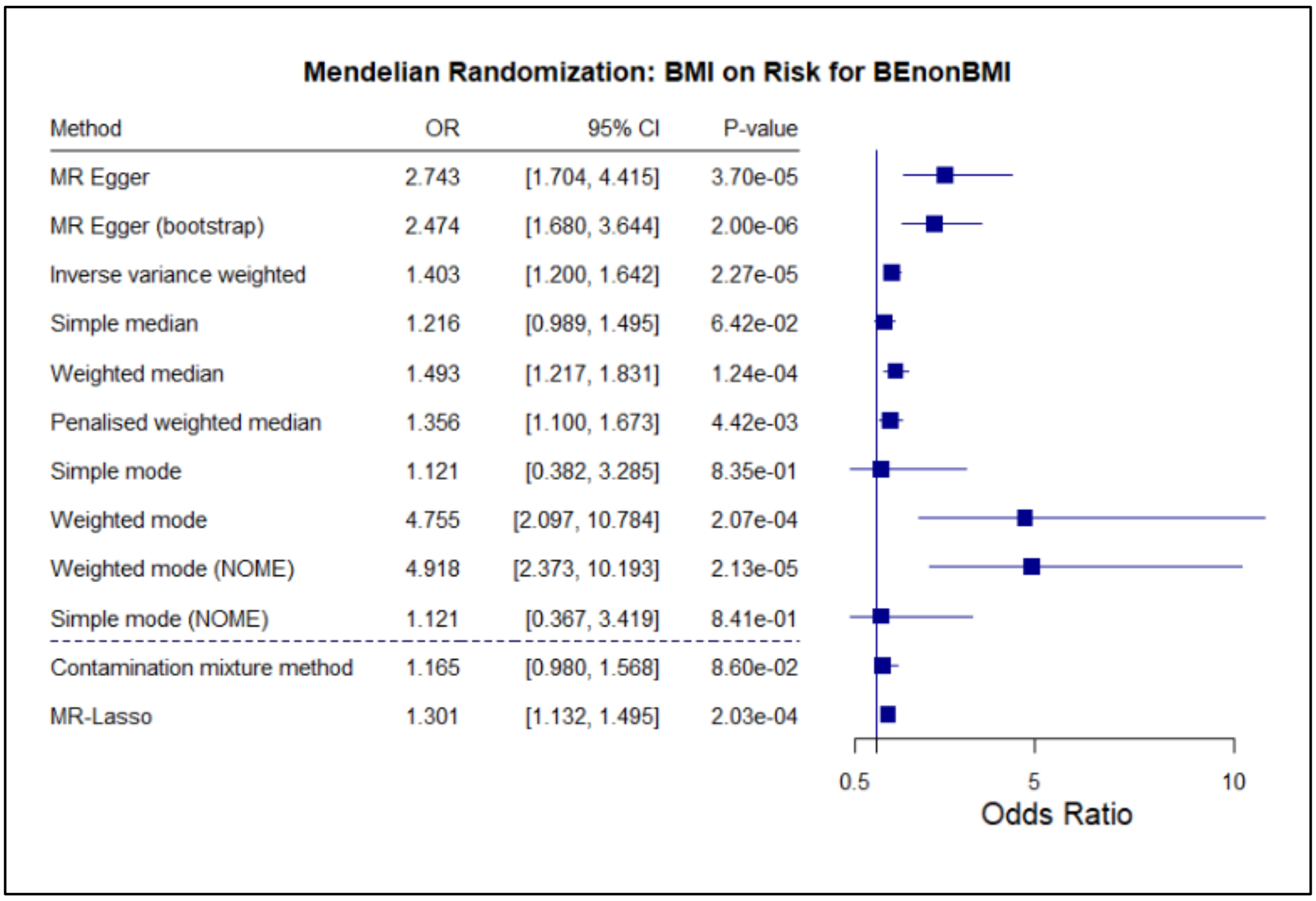

Mendelian Randomization: BMI on Risk for BEnonBMI

Method	OR	95% CI	P-value
MR Egger	2.743	[1.704, 4.415]	3.70e-05
MR Egger (bootstrap)	2.474	[1.680, 3.644]	2.00e-06
Inverse variance weighted	1.403	[1.200, 1.642]	2.27e-05
Simple median	1.216	[0.989, 1.495]	6.42e-02
Weighted median	1.493	[1.217, 1.831]	1.24e-04
Penalised weighted median	1.356	[1.100, 1.673]	4.42e-03
Simple mode	1.121	[0.382, 3.285]	8.35e-01
Weighted mode	4.755	[2.097, 10.784]	2.07e-04
Weighted mode (NOME)	4.918	[2.373, 10.193]	2.13e-05
Simple mode (NOME)	1.121	[0.367, 3.419]	8.41e-01
Contamination mixture method	1.165	[0.980, 1.568]	8.60e-02
MR-Lasso	1.301	[1.132, 1.495]	2.03e-04

Supplementary Figure 27h

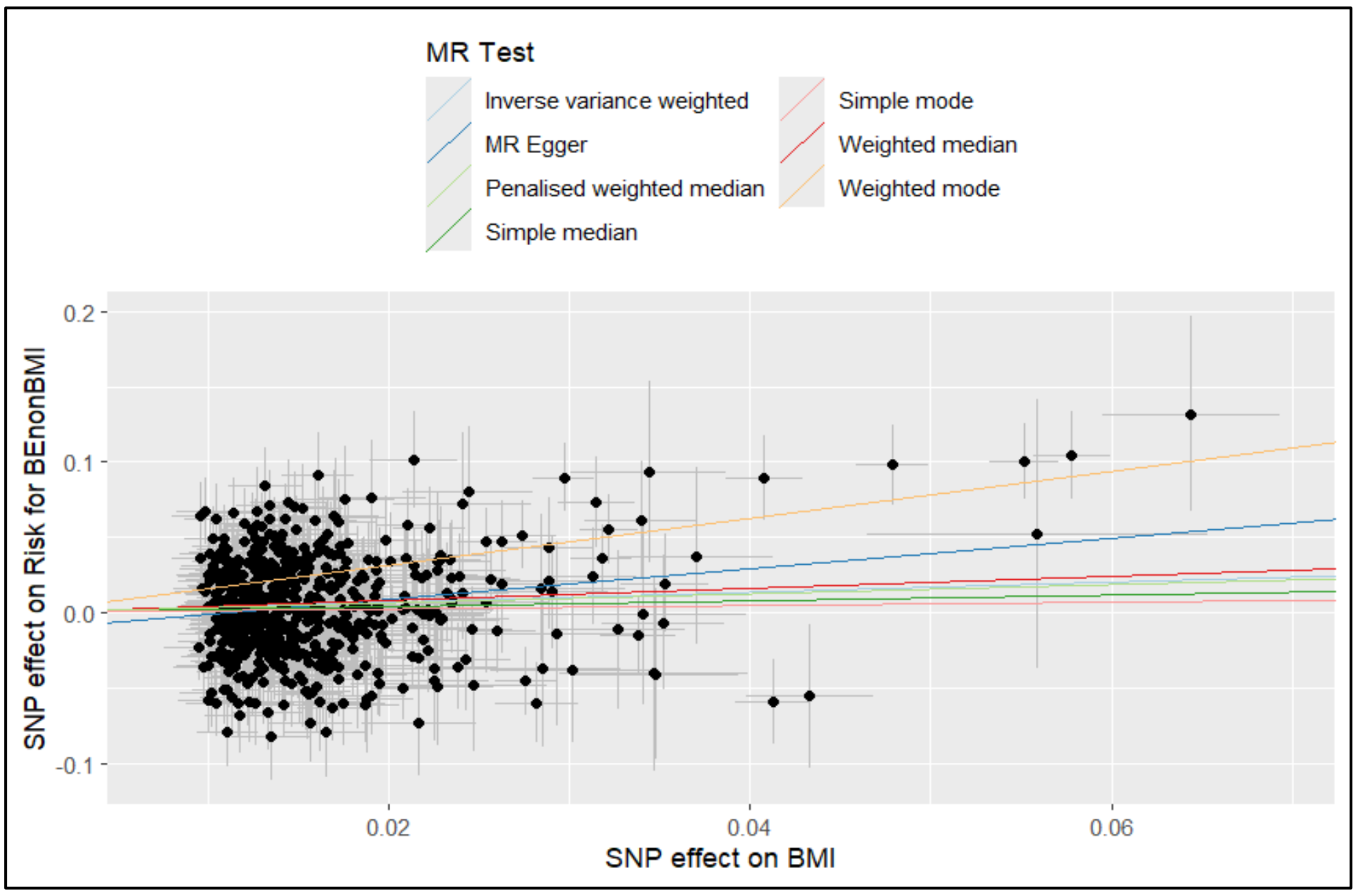

Supplementary Figure 28: Forest and scatter plots from Mendelian randomisation analyses with AN

(a, b) AN acting on BMI; (c, d) *NonBMI* AN acting on BMI; (e, f) BMI acting on AN; (g, h) BMI acting on *NonBMI* AN. Plots a, c, e, and g show the effect size (beta) and standard error of different Mendelian randomisation analyses of a given exposure on a given outcome. Plots b, d, f, and h show the effect (on the beta scale) and standard error of the effect of each variant (dots) on both exposure and outcome, with regression slopes from different Mendelian randomisation analyses.

Supplementary Figure 28a

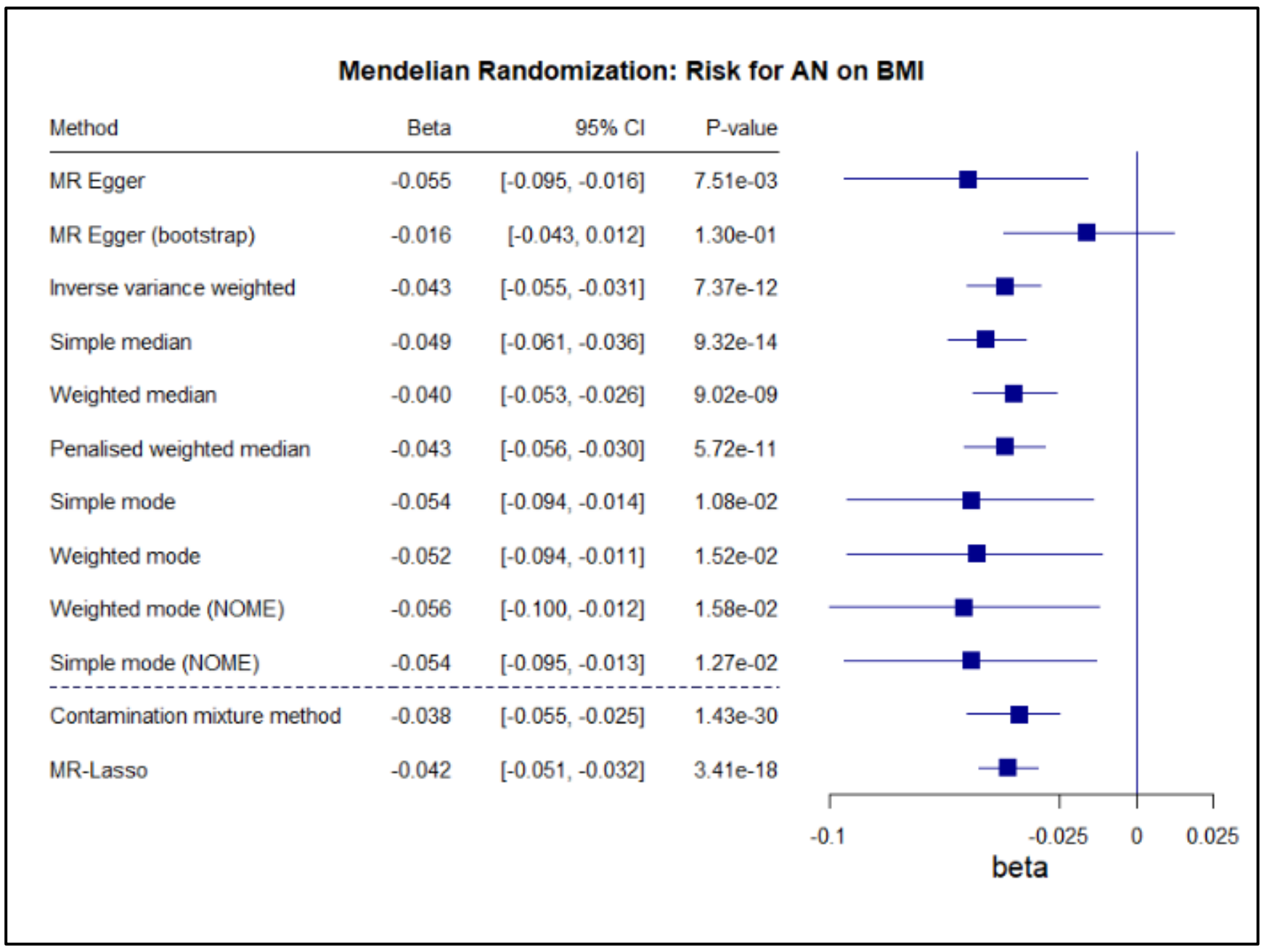

Supplementary Figure 28b

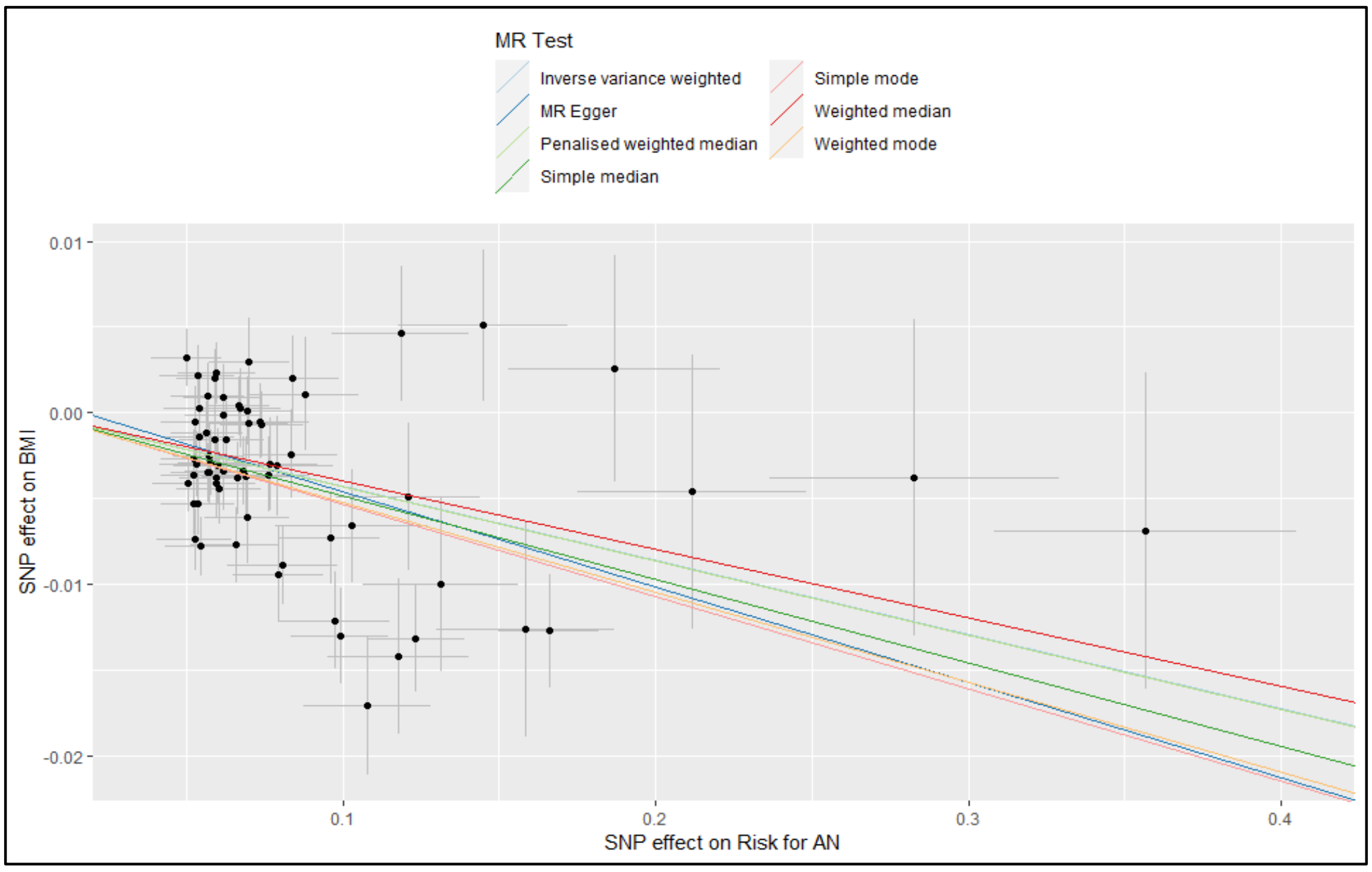

Supplementary Figure 28c

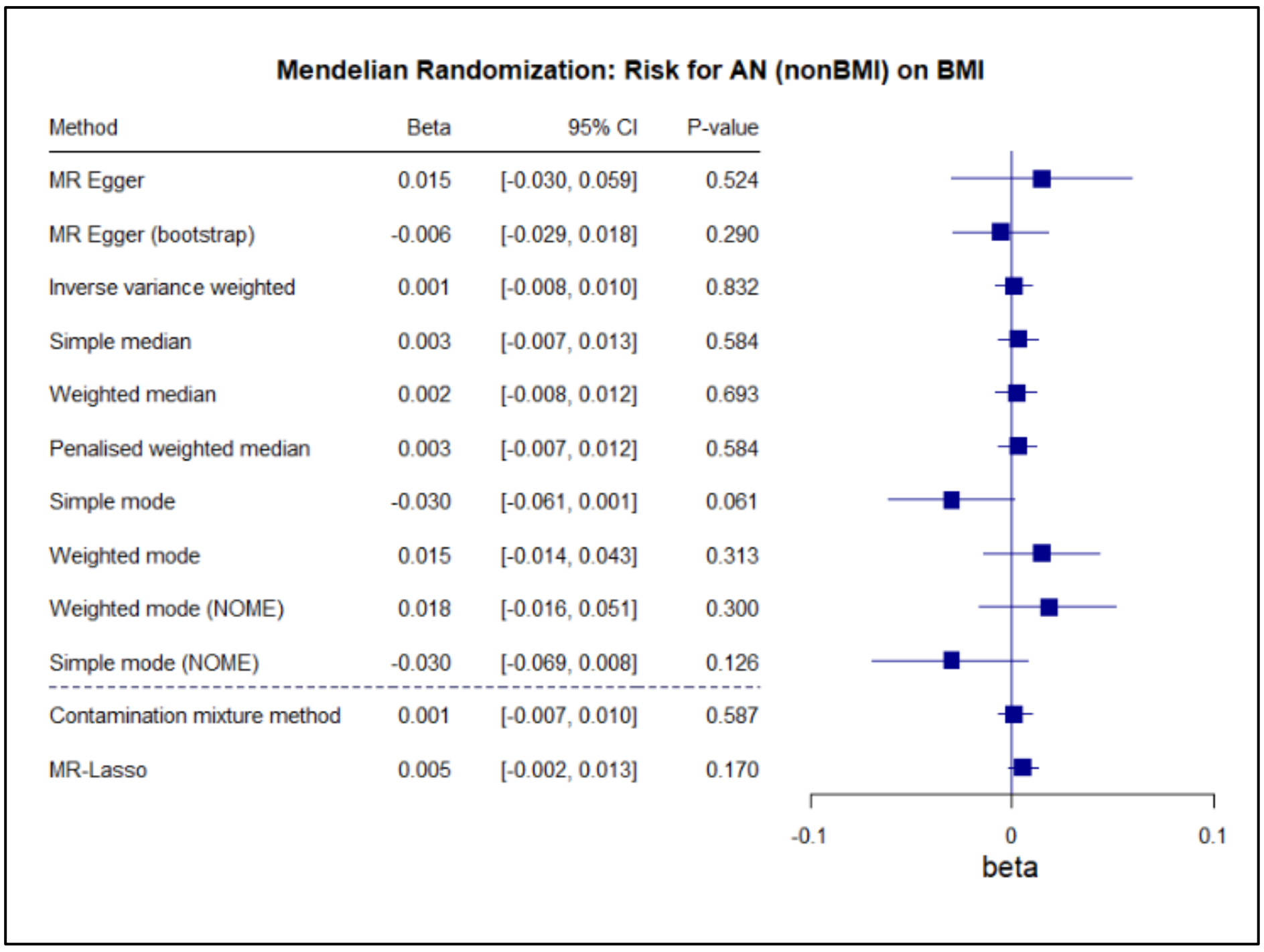

Method	Beta	95% CI	P-value
MR Egger	0.015	[-0.030, 0.059]	0.524
MR Egger (bootstrap)	-0.006	[-0.029, 0.018]	0.290
Inverse variance weighted	0.001	[-0.008, 0.010]	0.832
Simple median	0.003	[-0.007, 0.013]	0.584
Weighted median	0.002	[-0.008, 0.012]	0.693
Penalised weighted median	0.003	[-0.007, 0.012]	0.584
Simple mode	-0.030	[-0.061, 0.001]	0.061
Weighted mode	0.015	[-0.014, 0.043]	0.313
Weighted mode (NOME)	0.018	[-0.016, 0.051]	0.300
Simple mode (NOME)	-0.030	[-0.069, 0.008]	0.126
Contamination mixture method	0.001	[-0.007, 0.010]	0.587
MR-Lasso	0.005	[-0.002, 0.013]	0.170

Supplementary Figure 28d

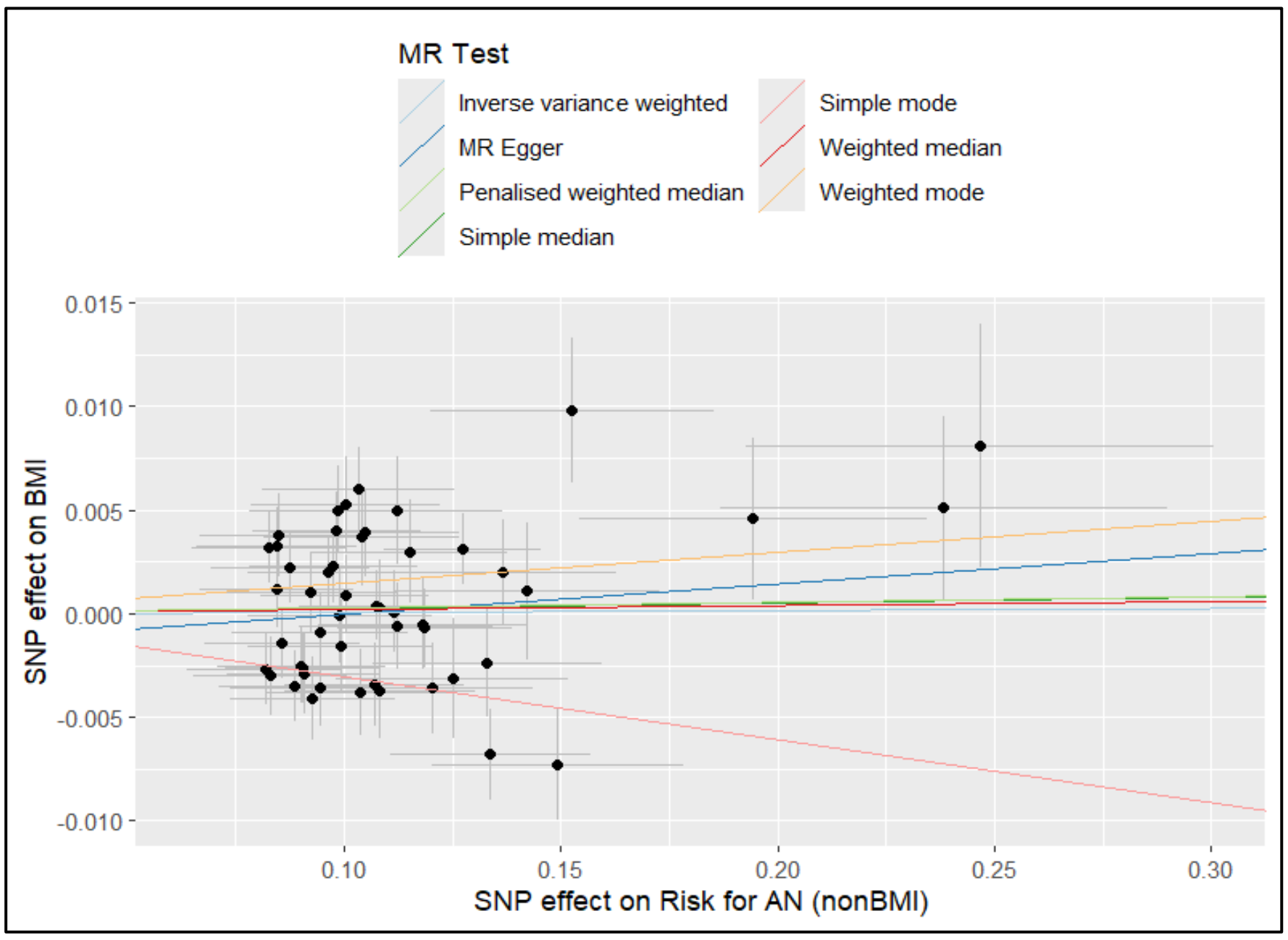

Supplementary Figure 28e

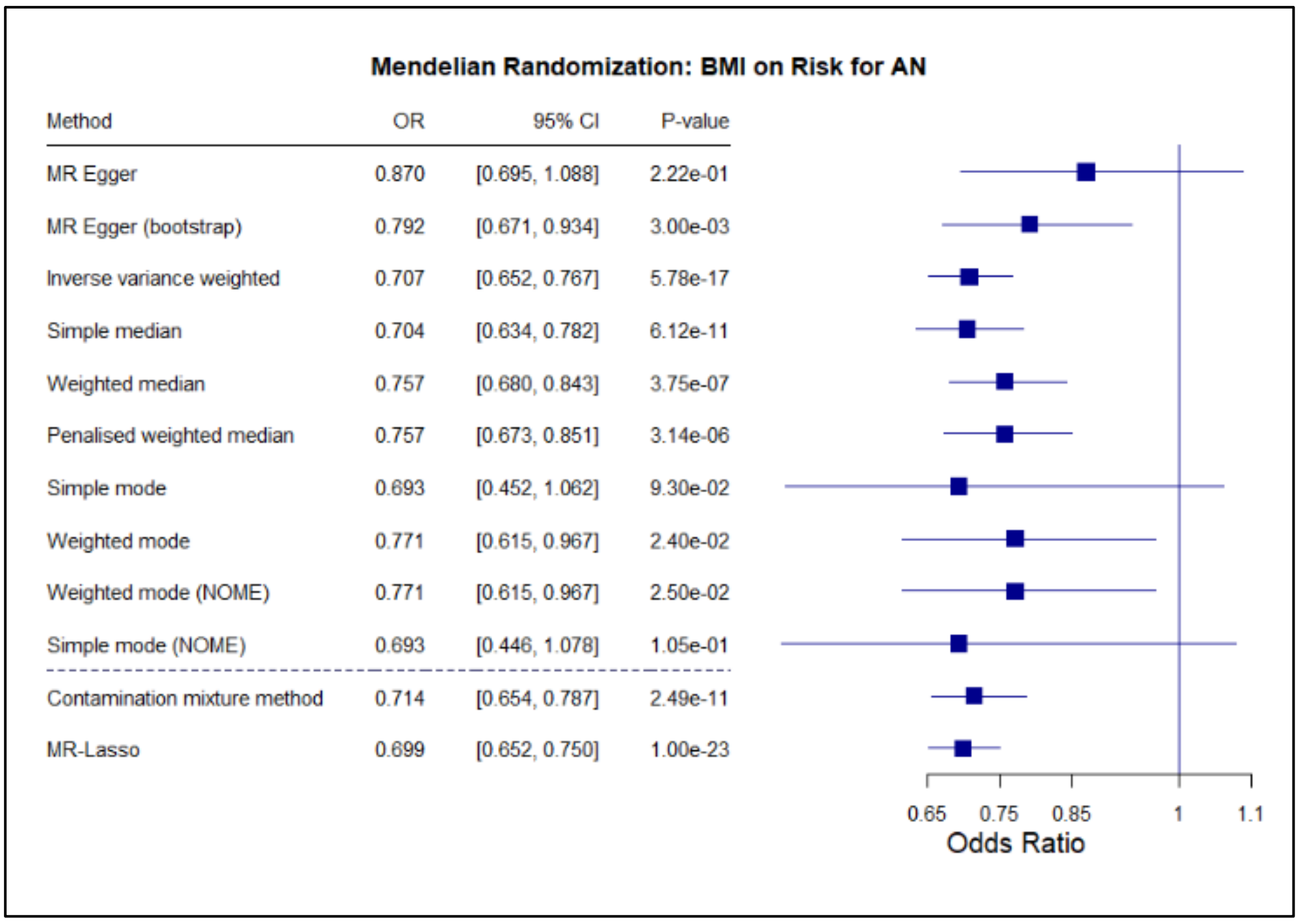

Mendelian Randomization: BMI on Risk for AN

Method	OR	95% CI	P-value
MR Egger	0.870	[0.695, 1.088]	2.22e-01
MR Egger (bootstrap)	0.792	[0.671, 0.934]	3.00e-03
Inverse variance weighted	0.707	[0.652, 0.767]	5.78e-17
Simple median	0.704	[0.634, 0.782]	6.12e-11
Weighted median	0.757	[0.680, 0.843]	3.75e-07
Penalised weighted median	0.757	[0.673, 0.851]	3.14e-06
Simple mode	0.693	[0.452, 1.062]	9.30e-02
Weighted mode	0.771	[0.615, 0.967]	2.40e-02
Weighted mode (NOME)	0.771	[0.615, 0.967]	2.50e-02
Simple mode (NOME)	0.693	[0.446, 1.078]	1.05e-01
Contamination mixture method	0.714	[0.654, 0.787]	2.49e-11
MR-Lasso	0.699	[0.652, 0.750]	1.00e-23

Supplementary Figure 28f

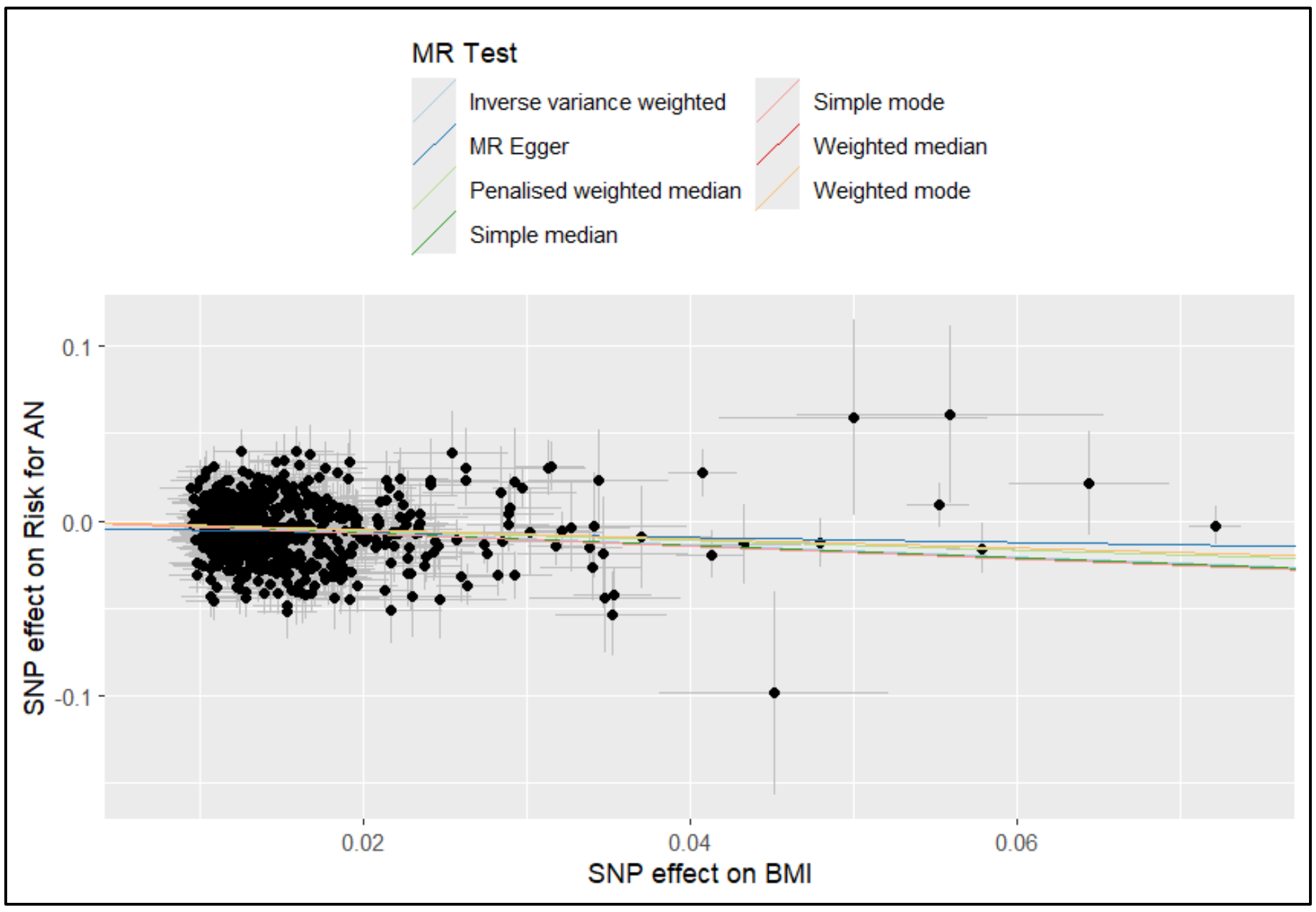

Supplementary Figure 28g

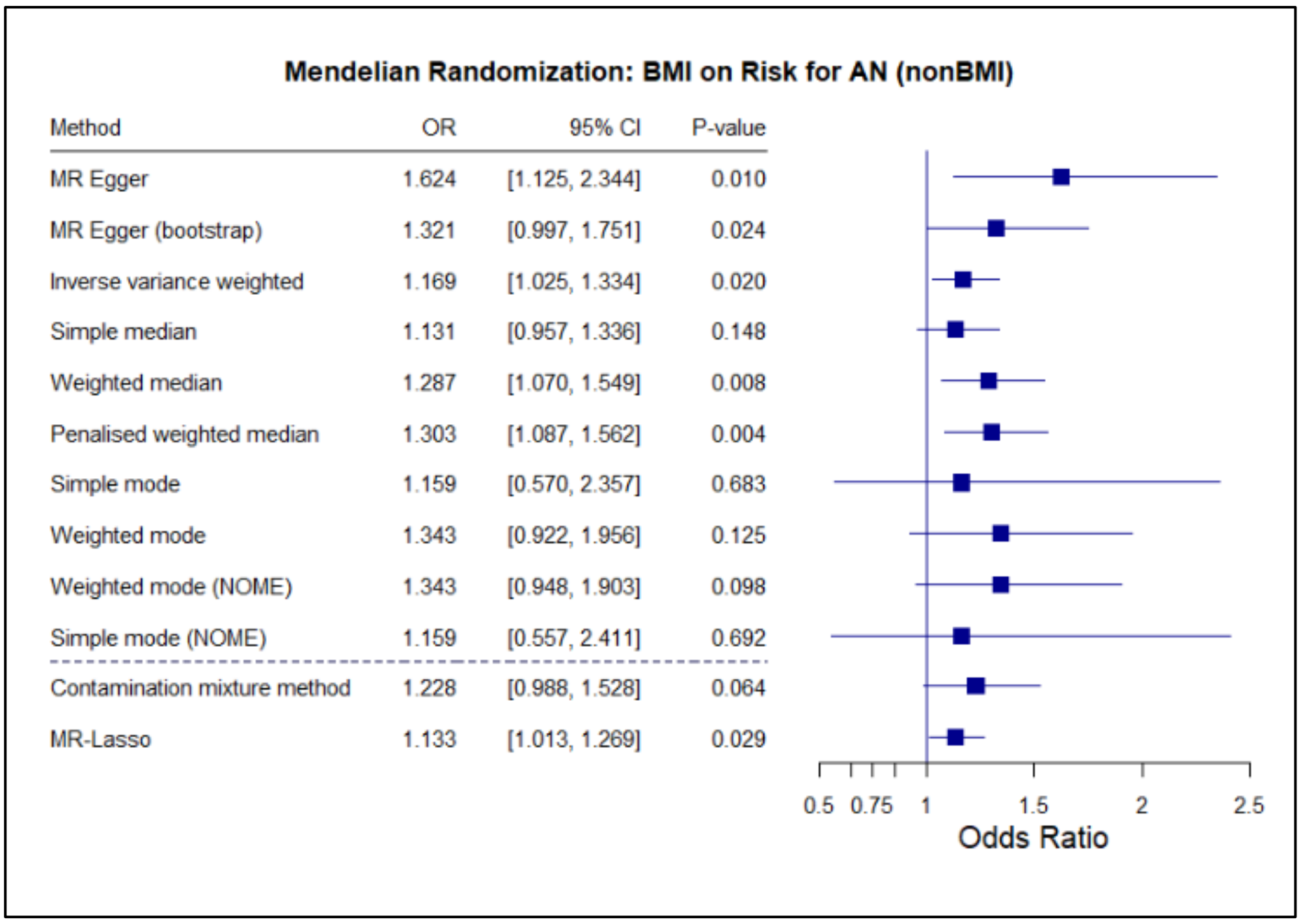

Supplementary Figure 28h

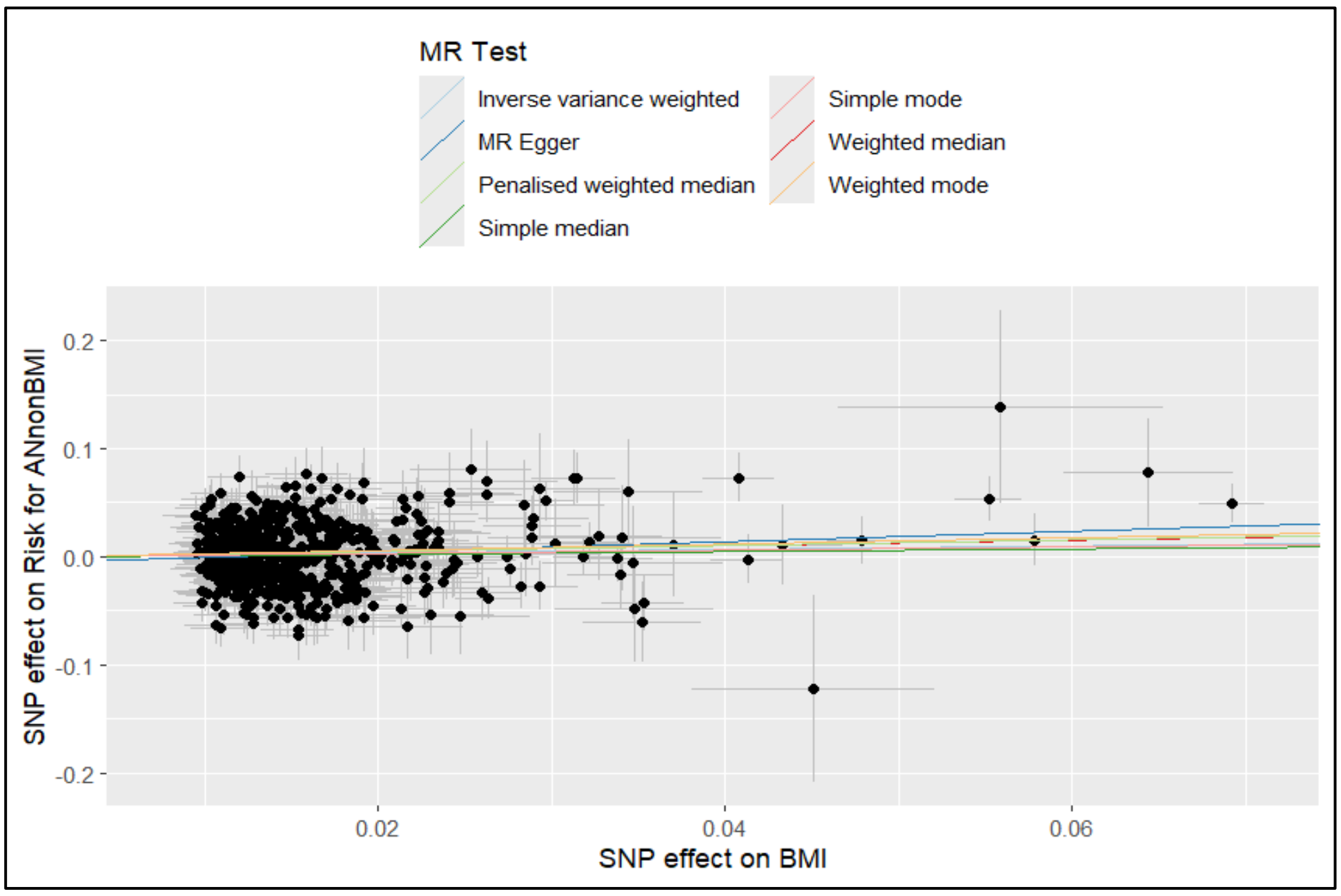

References

1. Watson, H. J. *et al.* Genome-wide association study identifies eight risk loci and implicates metabo-psychiatric origins for anorexia nervosa. *Nat. Genet.* **51**, 1207–1214 (2019).

2. Boraska, V. *et al.* A genome-wide association study of anorexia nervosa. *Mol. Psychiatry* **19**, 1085–1094 (2014).

3. Duncan, L. *et al.* Significant Locus and Metabolic Genetic Correlations Revealed in Genome-Wide Association Study of Anorexia Nervosa. *Am. J. Psychiatry* **174**, 850–858 (2017).

4. Wang, K. *et al.* A genome-wide association study on common SNPs and rare CNVs in anorexia nervosa. *Mol. Psychiatry* **16**, 949–959 (2011).

5. McKay, J. D. *et al.* A genome-wide association study of upper aerodigestive tract cancers conducted within the INHANCE consortium. *PLoS Genet.* **7**, e1001333 (2011).

6. Kaye, W. H. *et al.* A search for susceptibility loci for anorexia nervosa: methods and sample description. *Biol. Psychiatry* **47**, 794–803 (2000).

7. Kaye, W. H. *et al.* Genetic analysis of bulimia nervosa: methods and sample description. *Int. J. Eat. Disord.* **35**, 556–570 (2004).

8. Fichter, M. M. *et al.* Structured interview for anorexia and bulimia nervosa (SIAB): Development of a new instrument for the assessment of eating disorders. *Int. J. Eat. Disord.* **10**, 571–592 (1991).

9. Thornton, L. M. *et al.* The Anorexia Nervosa Genetics Initiative (ANGI): Overview and methods. *Contemp. Clin. Trials* **74**, 61–69 (2018).

10. Bulik, C. M. *et al.* The Binge Eating Genetics Initiative (BEGIN): study protocol. *BMC Psychiatry* **20**, 307 (2020).

11. Kirk, K. M. *et al.* The Anorexia Nervosa Genetics Initiative: Study description and sample characteristics of the Australian and New Zealand arm. *Aust. N. Z. J. Psychiatry* **51**, 583–594 (2017).

12. Olsen, C. M. *et al.* Cohort profile: the QSkin Sun and Health Study. *Int. J. Epidemiol.* **41**, 929–929i (2012).

13. Pedersen, C. B. *et al.* The iPSYCH2012 case-cohort sample: new directions for unravelling genetic and environmental architectures of severe mental disorders. *Mol. Psychiatry* **23**, 6–14 (2018).

14. Pedersen, C. B. The danish civil registration system. *Scand. J. Public Health* **39**, 22–25 (2011).

15. Mors, O., Perto, G. P. & Mortensen, P. B. The danish psychiatric central research register. *Scand. J. Public Health* **39**, 54–57 (2011).

16. Birgegård, A., Björck, C. & Clinton, D. Quality assurance of specialised treatment of eating disorders using large-scale Internet-based collection systems: methods, results and lessons learned from designing the Stepwise database. *Eur. Eat. Disord. Rev.* **18**, 251–259 (2010).

17. Almqvist, C. *et al.* LifeGene--a large prospective population-based study of global relevance. *Eur. J. Epidemiol.* **26**, 67–77 (2011).

18. Reba, L. *et al.* Relationships between features associated with vomiting in purging-type eating disorders. *Int. J. Eat. Disord.* **38**, 287–294 (2005).

19. Jonassaint, C. R. *et al.* Absence of association between specific common variants of the obesity-related FTO gene and psychological and behavioral eating disorder phenotypes. *Am. J. Med. Genet. B Neuropsychiatr. Genet.* **156B**, 454–461 (2011).

20. Byrne, E. M. *et al.* Cohort profile: the Australian genetics of depression study. *BMJ Open* **10**, e032580 (2020).

21. Micali, N. *et al.* Adolescent Eating Disorders Predict Psychiatric, High-Risk Behaviors and Weight Outcomes in Young Adulthood. *J. Am. Acad. Child Adolesc. Psychiatry* **54**, 652-659.e1 (2015).

22. Kann, L. *et al.* Youth risk behavior surveillance--United States, 1995. *J. Sch. Health* **66**, 365–377 (1996).

23. Leitsalu, L. *et al.* Cohort profile: estonian biobank of the estonian genome center, university of tartu. *Int. J. Epidemiol.* **44**, 1137–1147 (2015).

24. Kurki, M. I. *et al.* FinnGen provides genetic insights from a well-phenotyped isolated population. *Nature* **613**, 508–518 (2023).

25. Magnus, P. *et al.* Cohort profile update: the Norwegian Mother and Child Cohort Study (MoBa). *Int. J. Epidemiol.* **45**, 382–388 (2016).

26. Rønningen, K. S. *et al.* The biobank of the Norwegian Mother and Child Cohort Study: a resource for the next 100 years. *Eur. J. Epidemiol.* **21**, 619–625 (2006).

27. Paltiel, L. *et al.* The biobank of the Norwegian Mother and Child Cohort Study – present status. *Nor J Epidemiol* **24**, (2014).

28. Corfield, E. C. *et al.* The Norwegian Mother, Father, and Child cohort study (MoBa) genotyping data resource: MoBaPsychGen pipeline v.1. *BioRxiv* (2022) doi:10.1101/2022.06.23.496289.

29. Sudlow, C. *et al.* UK Biobank: An open access resource for identifying the causes of a wide range of complex diseases of middle and old age. *PLoS Med.* **12**, e1001779 (2015).

30. Davis, K. A. S. *et al.* Mental health in UK Biobank - development, implementation and results from an online questionnaire completed by 157 366 participants: a reanalysis. *BJPsych Open* **6**, e18 (2020).

31. King’s College London e-Research team. King’s Computational Research, Engineering and Technology Environment (CREATE). *King’s College London* (2022) doi:10.18742/rnvf-m076.

32. Davies, M. R. *et al.* The Genetic Links to Anxiety and Depression (GLAD) Study: Online recruitment into the largest recontactable study of depression and anxiety. *Behav. Res. Ther.* **123**, 103503 (2019).

33. 1000 Genomes Project Consortium *et al.* A global reference for human genetic variation. *Nature* **526**, 68–74 (2015).

34. Taliun, D. *et al.* Sequencing of 53,831 diverse genomes from the NHLBI TOPMed Program. *Nature* **590**, 290–299 (2021).

35. Loh, P.-R. *et al.* Reference-based phasing using the Haplotype Reference Consortium panel. *Nat. Genet.* **48**, 1443–1448 (2016).

36. Das, S. *et al.* Next-generation genotype imputation service and methods. *Nat. Genet.* **48**, 1284–1287 (2016).

37. McCarthy, S. *et al.* A reference panel of 64,976 haplotypes for genotype imputation. *Nat. Genet.* **48**, 1279–1283 (2016).

38. Lam, M. *et al.* RICOPILI: rapid imputation for consortias pipeline. *Bioinformatics* **36**, 930–933 (2020).

39. Chen, W. *et al.* Improved analyses of GWAS summary statistics by reducing data heterogeneity and errors. *Nat. Commun.* **12**, 7117 (2021).

40. Bybjerg-Grauholm, J. *et al.* The iPSYCH2015 Case-Cohort sample: updated directions for unravelling genetic and environmental architectures of severe mental disorders. *medRxiv* (2020) doi:10.1101/2020.11.30.20237768.

41. Mitt, M. *et al.* Improved imputation accuracy of rare and low-frequency variants using population-specific high-coverage WGS-based imputation reference panel. *Eur. J. Hum. Genet.* **25**, 869–876 (2017).

42. de Bakker, P. I. W. *et al.* Practical aspects of imputation-driven meta-analysis of genome-wide association studies. *Hum. Mol. Genet.* **17**, R122-8 (2008).

43. Purcell, S., Cherny, S. S. & Sham, P. C. Genetic Power Calculator: design of linkage and association genetic mapping studies of complex traits. *Bioinformatics* **19**, 149–150 (2003).

44. Zheng, Z. *et al.* Leveraging functional genomic annotations and genome coverage to improve polygenic prediction of complex traits within and between ancestries. *Nat. Genet.* **56**, 767–777 (2024).

45. Lawlor, D. A., Harbord, R. M., Sterne, J. A. C., Timpson, N. & Davey Smith, G. Mendelian randomization: using genes as instruments for making causal inferences in epidemiology. *Stat. Med.* **27**, 1133–1163 (2008).

46. Haycock, P. C. *et al.* Best (but oft-forgotten) practices: the design, analysis, and interpretation of Mendelian randomization studies. *Am. J. Clin. Nutr.* **103**, 965–978 (2016).

47. Sanderson, E., Spiller, W. & Bowden, J. Testing and correcting for weak and pleiotropic instruments in two-sample multivariable Mendelian randomization. *Stat. Med.* **40**, 5434–5452 (2021).

48. Bowden, J., Davey Smith, G. & Burgess, S. Mendelian randomization with invalid instruments: effect estimation and bias detection through Egger regression. *Int. J. Epidemiol.* **44**, 512–525 (2015).

49. Bowden, J., Hemani, G. & Davey Smith, G. Invited Commentary: Detecting Individual and Global Horizontal Pleiotropy in Mendelian Randomization-A Job for the Humble Heterogeneity Statistic? *Am. J. Epidemiol.* **187**, 2681–2685 (2018).

50. Burgess, S. & Thompson, S. G. Interpreting findings from Mendelian randomization using the MR-Egger method. *Eur. J. Epidemiol.* **32**, 377–389 (2017).

51. Verbanck, M., Chen, C.-Y., Neale, B. & Do, R. Detection of widespread horizontal pleiotropy in causal relationships inferred from Mendelian randomization between complex traits and diseases. *Nat. Genet.* **50**, 693–698 (2018).

52. Burgess, S., Bowden, J., Fall, T., Ingelsson, E. & Thompson, S. G. Sensitivity Analyses for Robust Causal Inference from Mendelian Randomization Analyses with Multiple Genetic Variants. *Epidemiology* **28**, 30–42 (2017).

53. Burgess, S. *et al.* Guidelines for performing Mendelian randomization investigations: update for summer 2023. *Wellcome Open Res.* **4**, 186 (2019).

54. Burgess, S., Butterworth, A. & Thompson, S. G. Mendelian randomization analysis with multiple genetic variants using summarized data. *Genet. Epidemiol.* **37**, 658–665 (2013).

55. Hartwig, F. P., Davey Smith, G. & Bowden, J. Robust inference in summary data Mendelian randomization via the zero modal pleiotropy assumption. *Int. J. Epidemiol.* **46**, 1985–1998 (2017).

56. Bowden, J., Davey Smith, G., Haycock, P. C. & Burgess, S. Consistent Estimation in Mendelian Randomization with Some Invalid Instruments Using a Weighted Median Estimator. *Genet. Epidemiol.* **40**, 304–314 (2016).

57. Rees, J. M. B., Wood, A. M., Dudbridge, F. & Burgess, S. Robust methods in Mendelian randomization via penalization of heterogeneous causal estimates. *PLoS ONE* **14**, e0222362 (2019).

58. Burgess, S., Foley, C. N., Allara, E., Staley, J. R. & Howson, J. M. M. A robust and efficient method for Mendelian randomization with hundreds of genetic variants. *Nat. Commun.* **11**, 376 (2020).

59. Hartwig, F. P., Davies, N. M., Hemani, G. & Davey Smith, G. Two-sample Mendelian randomization: avoiding the downsides of a powerful, widely applicable but potentially fallible technique. *Int. J. Epidemiol.* **45**, 1717–1726 (2016).

60. Arnold, M., Raffler, J., Pfeufer, A., Suhre, K. & Kastenmüller, G. SNiPA: an interactive, genetic variant-centered annotation browser. *Bioinformatics* **31**, 1334–1336 (2015).

61. Hemani, G. *et al.* The MR-Base platform supports systematic causal inference across the human phenome. *eLife* **7**, (2018).

62. Chang, C. C. *et al.* Second-generation PLINK: rising to the challenge of larger and richer datasets. *Gigascience* **4**, s13742–015–0047–8 (2015).

63. GTEx Consortium *et al.* Genetic effects on gene expression across human tissues. *Nature* **550**, 204–213 (2017).

64. Siletti, K. *et al.* Transcriptomic diversity of cell types across the adult human brain. *Science* **382**, eadd7046 (2023).

65. Bryois, J. *et al.* Genetic identification of cell types underlying brain complex traits yields insights into the etiology of Parkinson's disease. *Nat. Genet.* **52**, 482–493 (2020).

66. Finucane, H. K. *et al.* Heritability enrichment of specifically expressed genes identifies disease-relevant tissues and cell types. *Nat. Genet.* **50**, 621–629 (2018).

67. van der Meer, D. *et al.* Mapping the genetic landscape of psychiatric disorders with the mixer toolset. *Biol. Psychiatry* **98**, 455–465 (2025).

68. American Psychiatric Association. *Diagnostic and Statistical Manual of Mental Disorders (DSM-5®)*. (American Psychiatric Pub, 2013).

69. Walsh, B. T., Hagan, K. E. & Lockwood, C. A systematic review comparing atypical anorexia nervosa and anorexia nervosa. *Int. J. Eat. Disord.* **56**, 798–820 (2023).

70. Saito, T. & Rehmsmeier, M. The precision-recall plot is more informative than the ROC plot when evaluating binary classifiers on imbalanced datasets. *PLoS ONE* **10**, e0118432 (2015).

71. Burstein, D. *et al.* Genome-wide analysis of a model-derived binge eating disorder phenotype identifies risk loci and implicates iron metabolism. *Nat. Genet.* **55**, 1462–1470 (2023).

72. Pulit, S. L. *et al.* Meta-analysis of genome-wide association studies for body fat distribution in 694 649 individuals of European ancestry. *Hum. Mol. Genet.* **28**, 166–174 (2019).

73. Holmberg, M. J. & Andersen, L. W. Collider Bias. *JAMA* **327**, 1282–1283 (2022).
