# Supplementary material for: Genomic meta-analyses of binge-eating behavior and anorexia nervosa yield insights into the unique and shared biology of eating disorder phenotypes
Source: Nat Ment Health. 2026 Aug 19;4(9):1361–77. doi: 10.1038/s44220-026-00698-2 (PMC13553282; doi:10.1038/s44220-026-00698-2)
Supplement: Supplementary file 2 [file 44220_2026_698_MOESM2_ESM.pdf]

Corresponding author(s): JRI Coleman, LM Huckins, CM Bulik, G Breen

Last updated by author(s): May 8, 2026

Reporting Summary

Nature Portfolio wishes to improve the reproducibility of the work that we publish. This form provides structure for consistency and transparency in reporting. For further information on Nature Portfolio policies, see our Editorial Policies and the Editorial Policy Checklist.

Statistics

For all statistical analyses, confirm that the following items are present in the figure legend, table legend, main text, or Methods section.

n/a	Confirmed	
☐	☒	The exact sample size (*n*) for each experimental group/condition, given as a discrete number and unit of measurement
☐	☒	A statement on whether measurements were taken from distinct samples or whether the same sample was measured repeatedly
☐	☒	The statistical test(s) used AND whether they are one- or two-sided<br>*Only common tests should be described solely by name; describe more complex techniques in the Methods section.*
☐	☒	A description of all covariates tested
☐	☒	A description of any assumptions or corrections, such as tests of normality and adjustment for multiple comparisons
☐	☒	A full description of the statistical parameters including central tendency (e.g. means) or other basic estimates (e.g. regression coefficient) AND variation (e.g. standard deviation) or associated estimates of uncertainty (e.g. confidence intervals)
☐	☒	For null hypothesis testing, the test statistic (e.g. *F*, *t*, *r*) with confidence intervals, effect sizes, degrees of freedom and *P* value noted<br>*Give P values as exact values whenever suitable.*
☐	☒	For Bayesian analysis, information on the choice of priors and Markov chain Monte Carlo settings
☒	☐	For hierarchical and complex designs, identification of the appropriate level for tests and full reporting of outcomes
☐	☒	Estimates of effect sizes (e.g. Cohen's *d*, Pearson's *r*), indicating how they were calculated

Our web collection on statistics for biologists contains articles on many of the points above.

Software and code

Policy information about availability of computer code

Data collection	No software was used for data collection for this paper.
Data analysis	We used the following software in data analysis: PLINK1.9 (1.9b_6.17-x86_64); PLINK2 (various versions across sites, version 2.00a2.3_x86_64 used centrally); ReGENIE (various versions across sites); SAIGE (various versions used across sites); LiftOver (no versioning at time of writing); Python (various versions, primarily 2.7.18); BCFTools (1.10.2); HRC-1000G-check-bim (4.3.0, available from https://www.chg.ox.ac.uk/~wrayner/tools/); DENTIST (1.2.0); RICOPILI (2019, Oct); METAL (2011-03-25); GCTA-COJO (1.93.1); PRS-CS (1.1.0); LDSC (1.0.0); CC-GWAS (no versioning at time of writing); GenomicSEM (v0.0.5c); R (various versions); TwoSampleMR (0.6.8); MendelianRandomisation(0.10.0); MRPRESSO (1.0); S-PrediXcan/S-MultiXcan (0.6.11); MAGMA (1.10); MiXeR (1.2.0). Imputation was conducted on various platforms and to various reference panels, but primarily used the TopMED imputation server (https://imputation.biodatacatalyst.nhlbi.nih.gov/) imputing to TopMED freeze 8, using Eagle (2.4) for phasing and Minimac4 for imputation. Linking code between these different software was used but was not central to the research - this code will be made publicly available on GitHub after publication (https://github.com/psychiatric-genomics-consortium/PGC3_EAD_Public).

For manuscripts utilizing custom algorithms or software that are central to the research but not yet described in published literature, software must be made available to editors and reviewers. We strongly encourage code deposition in a community repository (e.g. GitHub). See the Nature Portfolio guidelines for submitting code & software for further information.

Data

Policy information about availability of data

All manuscripts must include a data availability statement. This statement should provide the following information, where applicable:

- Accession codes, unique identifiers, or web links for publicly available datasets
- A description of any restrictions on data availability
- For clinical datasets or third party data, please ensure that the statement adheres to our policy

Individual level genotype data (except in countries where sharing of individual level data is prohibited by national law) and summary statistics used in this study are available to bona fide researchers working in collaboration with a member of the Eating Disorders Working Group of the PGC via secondary analysis proposals (https://pgc.unc.edu/for-researchers/data-access-committee/data-access-information/).

Summary statistics from this work will be made available via Figshare and the PGC website on publication (https://pgc.unc.edu/for-researchers/download-results/).

Research involving human participants, their data, or biological material

Policy information about studies with human participants or human data. See also policy information about sex, gender (identity/presentation), and sexual orientation and race, ethnicity and racism.

Reporting on sex and gender	Our analyses include stratification by sex. As part of our genomic quality control measures, we align genetic sex (inferred through X-chromosome heterozygosity) with reported "sex" – this is inferred to mean sex assigned at birth (and in some cohorts this may be explicitly specified) but in some cases, reported sex may reflect gender or sexual identity and not sex assigned at birth. As such, it is possible that some trans individuals, or individuals whose gender and sexual identity do not otherwise align with their sex assigned at birth, may have been incorrectly identified as sample swap errors and excluded from analysis. However, this is difficult to rectify, especially in cohorts collected some time ago where recontact of participants is not possible. We do not refer to gender in our analyses.<br><br>We conducted female-only GWAS, and used this to perform female-to-male polygenic score analysis. We did not conduct male-only GWAS as sample sizes were too small. Overall, our analysis includes 39,279 individuals reporting binge-eating behaviours and 1,227,436 comparison individuals, of whom 37,580 and 691,808 individuals respectively are female. Data was available on 1487 and 79,144 male individuals. For anorexia nervosa, we include 24,223 individuals with a history of anorexia nervosa and 1,243,971 comparison individuals, of which 21,898 and 694,722 individuals are female. Male data for the anorexia nervosa analyses was available for 718 and 353,303 individuals. Differences between sex stratified numbers and overall numbers reflect cohorts where stratified data were too small for inclusion.
Reporting on race, ethnicity, or other socially relevant groupings	Our analyses are mostly restricted to individuals from European ancestries. By this, we refer to homogeneous groupings of individuals on principal component axes derived from genome-wide genotype data, which reflect global human population structure. Given the locales from which we collected data, the majority grouping in most cohorts was comprised of participants who reported European ethnicities, and so we designate the participants in these groups as being of European ancestries. We recognise the Eurocentric predominance of data within genomics, and we are working to expand recruitment of participants to studies of eating disorder genetics to better reflect the global ancestry distribution. As an initial step, we include polygenic risk scoring analyses from our European ancestry GWAS into two cohorts from East Asian ancestries (defined parallel to the process described above).
Population characteristics	We do not consider population characteristics as covariates in our analyses as they were focused on discovering new variant-trait associations. The relationship between genetic and environmental factors in eating disorders is an important area of research, but the small effect sizes typical of common genetic effects on psychiatric traits necessitate the collection of very large sample sizes, which would be impaired by additionally requiring samples to have similarly-collected information on population characteristics. We anticipate that subsets of our underlying data will be used by future researchers to examine these more nuanced questions of gene-environment interplay.
Recruitment	Participants were recruited using a variety of different methods implemented across the different sites and cohorts. Broadly, we identified cases and controls based on clinical diagnoses, diagnostic algorithms, and/or self-report questionnaires. Most cohorts collected data from clinical eating disorder services - however, a substantial proportion of our anorexia nervosa analyses came from the ANGI project, which recruited participants via professional organisations such as the Academy for Eating Disorders, conventional media, social media, (press) conferences and support groups, and word-of-mouth. There are additionally large components of the study drawn from population cohorts like the MoBA cohort and the iPSYCH cohort, while other large components are drawn from population biobanks like UK Biobank, Estonian Biobank, and FinnGEN. Epidemiological biases likely affect each of our cohorts, although it is likely that some of these biases will not act consistently across cohorts. As such, these biases likely contribute to increasing the heterogeneity of our data, reducing precision, but will have less effect on biasing specific effect estimates.
Ethics oversight	Price Foundation Collaborative Group and Children's Hospital of Philadelphia (CHOP): Ethical approval was obtained by each of the individual study sites (Cornell University, University of California at Los Angeles, University of Pittsburgh, University of Toronto, University of London, and University of Munich.). For the CHOP controls, CHOP's Research Ethics Board approved the study, and informed consent was obtained from all participants or their parents. Anorexia Nervosa Genetics Initiative (ANGI): ANGI-Australia and New Zealand (ANGI-ANZ) and Queensland Skin Study (QSkin): For ANGI-ANZ, all participants provided informed consent. Ethical approval for the Australian arm of the study was obtained by the QIMR Berfhoger Human Research Ethics Committee; for New Zealand, this was provided by the Health and Disability Ethics Committee of the New Zealand Ministry of Health. For QSkin, ethical approval was obtained by the QIMR Berghofer Human Research Ethics Committee (QIMR-HEC approval P1309, P2034). ANGI-Denmark (ANGI-DK) and the The Lundbeck Foundation Initiative for

Integrative Psychiatric Research (iPSYCH): Participants from ANGI-DK and the iPSYCH study did not provide written informed consent, as an exemption from consent was provided by the Danish Scientific Ethics Committee (Videnskabsetisk Komité). This exemption was most recently approved in 2018. Additional Danish cases: All participants provided written informed consent before participating in the study. The study was approved by the Capital Region of Denmark's Committees on Health Research Ethics (De Videnskabsetiske Komiteer for Region Hovedstaden) (approval number H-KF-01-024/01). ANGI-United States of America (ANGI-US): ANGI-US was approved by the Institutional Review Board of the University of North Carolina at Chapel Hill (IRB: 13-0081). All participants were broadly consented for research. ANGI-Sweden, LifeGENE and Binge-Eating Genetics Initiative (BEGIN): This project has been approved by the ethical review board in Sweden. The fundament of the ethical permit comes from ANGI Sweden (dnr 2013/112-31/2) which has later been amended to also include individuals with another diagnosis than AN (dnr 2014/1563) and to change the study name to BEGIN (dnr 2016/1852-32). For LifeGene, the Regional Ethical Review Board of Stockholm provided ethical approval, and all participants provided online consent. Australian Genetics of Depression Study: Ethics approval for all aspects of the project was obtained from the QIMR Berghofer Human Research Ethics Committee (P2118 & P3434). Avon Longitudinal Study of Parents and Children (ALSPAC): Ethical approval for the study was obtained from the ALSPAC Ethics and Law Committee and the Local Research Ethics Committees. Informed consent for the use of data collected via questionnaires and clinics was obtained from participants following the recommendations of the ALSPAC Ethics and Law Committee at the time. The main caregiver initially provided consent for child participation, and from the age of 16 years, the offspring themselves have provided informed written consent. The Vanderbilt University biorepository (BioVU): The study was approved by the Vanderbilt University Medical Center Institutional Review Board (IRB #201609). The Estonian Biobank: The research project has obtained approval from the Estonian Council on Bioethics and Human Research (1.1-12/624). The Finnish Study of Genetics (FinnGEN): Written informed consent was obtained from all the study participants. For the Finnish Institute of Health and Welfare (THL)-driven FinnGen preparatory project and FinnGen project, all patients and control subjects had provided informed consent for biobank research, based on the Finnish Biobank Act. Alternatively, the North Karelia Project (FINRISK) and Health 2000 cohorts were based on study specific consents and later transferred to the THL Biobank after approval by Valvira, the National Supervisory Authority for Welfare and Health. Recruitment protocols followed the biobank protocols approved by Valvira. Data from the Eating Disorders Continuum (EDC) at the Douglas Mental Health University Institute in Montreal: In all cases, there was consent to participation in one or more of the original studies, as well as to sharing of DNA with other research groups for additional genetic studies. All aspects were approved by the Research Ethics Boards of the Douglas Mental Health University Institute or Montreal West Island Integrated University Health and Social Services Centre. Jannsen: The CAPSS220BED clinical study (ClinicalTrials.gov ID: NCT00210808) was conducted by Johnson & Johnson Pharmaceutical Research & Development, L.L.C., and was approved by the appropriate ethical review boards and followed the principles outlined in the Declaration of Helsinki for all human investigations. In addition, informed consent has been obtained from the study participants. The Norwegian Mother, Father and Child Cohort Study (MoBa): The establishment of MoBa and initial data collection was based on a licence from the Norwegian Data Protection Agency and approval from The Regional Committees for Medical and Health Research Ethics. The MoBa cohort is currently regulated by the Norwegian Health Registry Act. The current study was approved by The Regional Committees for Medical and Health Research Ethics (20311). Utrecht Research Group Eating Disorders (URGE) cohort: Informed consent was obtained from all patients who provided DNA. The Biobank Ethics Committee of the University of Utrecht gave ethical approval for this work. UK Biobank: UK Biobank is registered as a Research Tissue Bank (North West - Haydock Research Ethics Committee, 21/NW/0157). All data and sample applications may use this ethical clearance to conduct their research. Separate Research Ethics Committee or other ethical clearance is not required. This research was conducted under application 82087 (PI: Jonathan Coleman). Charlotte's Helix: Charlotte's Helix participants were recruited using ethics approval provided by the Research Ethics Committee South Central Oxford C (15/SC/0388). The Genetic Links to Anxiety and Depression (GLAD) Study, and the National Institute for Health Research (NIHR) Bioresource: The GLAD Study was approved by the London - Fulham Research Ethics Committee on 21st August 2018 (REC reference: 18/LO/1218) following a full review by the committee. The NIHR BioResource has been approved as a Research Tissue Bank by the East of England - Cambridge Central Committee (REC reference: 17/EE/0025). Korean Study of Anorexia Nervosa: The study was approved by the Institutional Review Board of Inje University (INJE 2016-01-003-002). Genetics Consortium for Anorexia Nervosa: Each study site (Medical University of Vienna, Toronto General Hospital, Charles University, University of Helsinki, Institut National de la Santé et de la Recherche Médicale, University of Pittsburgh, University of North Carolina at Chapel Hill, Roseneck Hospital for Behavioral Medicine, University of Munich, Kings College London, Laureate Psychiatric Hospital, Weill Cornell Medical College, Sheppard Pratt Health System, Neuropsychiatric Research Institute Fargo, University of California at Los Angeles, University of Pennsylvania, University of Birmingham, University of Duisburg-Essen, GlaxoSmithKline Leeds, Athens University, Medical School, Padua/Verona/Treviso/Vicenza/Portogruaro hospitals, University Medical Center Utrecht, Altrecht in Zeist, Poznan University of Medical Sciences, Center for Genomic Regulation Barcelona, Department of Psychiatry University Hospital of Bellvitge, Karolinska Institutet, McLean Hospital/Harvard Medical School, Vanderbilt University School of Medicine) obtained informed consent from all participants, as well as permission from local ethical committees.

Note that full information on the approval of the study protocol must also be provided in the manuscript.

Field-specific reporting

Please select the one below that is the best fit for your research. If you are not sure, read the appropriate sections before making your selection.

☐ Life sciences ☒ Behavioural & social sciences ☐ Ecological, evolutionary & environmental sciences

For a reference copy of the document with all sections, see nature.com/documents/nr-reporting-summary-flat.pdf

Behavioural & social sciences study design

All studies must disclose on these points even when the disclosure is negative.

Study description	Quantitative cross-sectional, observational, case-control study.
Research sample	Individuals reporting binge-eating behaviour and/or anorexia nervosa and comparison individuals. Recruited from multiple cohorts across the USA, Europe, and Australia, as well as additional cohorts from the Republic of Korea and Japan. Recruits are likely of various ages (not reported for all cohorts) and are predominantly female due to the higher prevalence of anorexia nervosa in women. The sample is not population representative, as it is in most cases deliberately over-recruited for eating disorders. The sample was designed to maximise available data for individuals with eating disorder phenotypes and genome-wide genotype data.
Sampling strategy	Sampling varied across cohorts, dependent on the original aim of the cohort. This analysis is secondary data analysis of pre-existing cohort data. No a priori effect size calculation was performed, as the intention of recruitment was to recruit all available individuals with eating disorder phenotypes and genome-wide genotype data. Post-recruitment effect size calculations were performed to indicate effect sizes that this study was powered to detect – these are not post hoc power calculations as they are not dependent on the observed statistics of the sample, only the sample size.
Data collection	Participants reported phenotypic data via self-report questionnaires, or phenotypes were gathered from medical records or interviews. Genotypic data was obtained by genome-wide genotyping using various microarrays.
Timing	The period of data collection varies by cohort.
Data exclusions	Data exclusions varied by cohort. In general, individuals were excluded if medical or psychiatric conditions were present that could have confounded a diagnosis of anorexia nervosa. Control individuals were generally screened for psychiatric illnesses, although this was not possible in all cohorts.
Non-participation	This was a cross-sectional study, and as such non-participation does not apply.
Randomization	Participants were not allocated into experimental groups.

Reporting for specific materials, systems and methods

We require information from authors about some types of materials, experimental systems and methods used in many studies. Here, indicate whether each material, system or method listed is relevant to your study. If you are not sure if a list item applies to your research, read the appropriate section before selecting a response.

Materials & experimental systems

n/a	Involved in the study	
☒	☐	Antibodies
☒	☐	Eukaryotic cell lines
☒	☐	Palaeontology and archaeology
☒	☐	Animals and other organisms
☒	☐	Clinical data
☒	☐	Dual use research of concern
☒	☐	Plants

Methods

n/a	Involved in the study	
☒	☐	ChIP-seq
☒	☐	Flow cytometry
☒	☐	MRI-based neuroimaging

Plants

Seed stocks	This field has persisted incorrectly in the form – not applicable.
Novel plant genotypes	This field has persisted incorrectly in the form – not applicable.
Authentication	This field has persisted incorrectly in the form – not applicable.
