# Supplementary material for: Genomic meta-analyses of binge-eating behavior and anorexia nervosa yield insights into the unique and shared biology of eating disorder phenotypes
Source: Nat Ment Health. 2026 Aug 19;4(9):1361–77. doi: 10.1038/s44220-026-00698-2 (PMC13553282; doi:10.1038/s44220-026-00698-2)
Supplement: Supplementary file 3 [file 44220_2026_698_MOESM3_ESM.pdf]

nature portfolio

Peer Review File

Genome-wide association studies of binge-eating behaviour and anorexia nervosa yield insights into the unique and shared biology of eating disorder phenotypes

Corresponding Author: Dr Jonathan Coleman

This file contains all reviewer reports in order by version, followed by all author rebuttals in order by version.

Version 0:

Reviewer comments:

Reviewer #1

(Remarks to the Author)
This substantially revised manuscript addresses all of the concerns that I expressed in my initial review.

One typo: Line 659: "casual mixture modeling"

Reviewer #2

(Remarks to the Author)
This is a revised manuscript describing, in impressive detail, a GWAS of individuals with anorexia nervosa, bulimia nervosa, and binge-eating disorder. The authors have been very responsive to the prior review and I believe the manuscript has been significantly improved. The focus on binge eating is clearer and the findings are interesting and important.

I am afraid, however, that I still have trouble understanding how some of the groups were created (I apologize if I'm missing something obvious, but other readers may also have trouble following.).

More specifically, I am confused about the handling of individuals with anorexia nervosa, binge eating/purging subtype. In the authors' response to reviewers, they state,
"'Case' individuals for the BE-BROAD analysis comprise all individuals who report binge-eating behaviours. This includes individuals with the binge-eating subtype of anorexia nervosa…"
But this is not clear in Table 1; the diagnostic codes in the last column for BE-BROAD do not appear to include any for anorexia nervosa--more specifically, F50.02. This should be clarified.

This also raises a question about the interesting analyses comparing BE-BROAD to anorexia nervosa. At least superficially, it seems that individuals with the binge/purge subtype could be included in either (or both??) groups. Was the BE-BROAD group compared only to individuals with the restricting subtype? I think these also need to be clarified.

Related to these issues, the acronym AN-BE is used on page 14 without a definition.

I would also point out that not all individuals with the binge/purge subtype of anorexia nervosa engage in binge eating; some purge after episodes of eating that are not binges. This is a minor point but the authors might wish to mention.

Reviewer #3

(Remarks to the Author)
This is a new genome-wide association study (GWAS) release from the Psychiatric Genomics Consortium (PGC) Eating Disorder (ED) Working Group, with 56,344 unique cases and over 1.2M controls. This represents a major expansion over prior PGC GWAS installments. A major focus of the paper is the parallel analyses of binge eating disorder (BE) and anorexia nervosa (AN) including varying narrow/broader phenotypic definitions and sensitivity analyses. Beyond simple

locus discovery, the authors have performed detailed and near-comprehensive characterizations of each trait's genetic architecture and shared genetic basis with a range of traits, including modeling eating disorder specific genetic variance not shared with body mass index (BMI).

MAIN CONCERNS

(1) The authors make an important point in the introduction, namely that standard DSM and ICD based phenotypic definitions "often exist on a continuum, overlap, co-occur, present heterogeneously, and morph over time". This idea could be better explored in the context of the current study, as it applies to the non-negligible overlap between BE-BROAD (18%) and AN cases (30%). It would ease readability and interpretation if this overlap was stated in the main text; I finally tracked it down in Supplementary Table 11. Similarly, it is relevant for presentation of the MiXeR results.

(2) The abstract refers to "the first genomic meta-analysis of binge-eating behaviour" and in the introduction, "we present the first GWAS of BE across multiple cohorts", and in the discussion "first genome-wide analysis of BE"--is this true, strictly speaking? There is some passing mention of model-derived BE phenotype in the Million Veteran Program. Do we mean "diagnosed BE", specifically?

(3) The presentation of directions of effect and significance levels for SNP-rg comparisons of BE-BROAD and AN with "other traits" should be improved -- please give the point estimates (or point to their location) in text, and make clearer which P-values correspond to which comparisons. We are asking for this because the results are quite interesting and warrant more detailed discussion.

(4) Given the emphasis on the MiXeR analysis, the interpretation could be clearer, e.g. the minimum possible overlap model. Similarly, what do the genetic distance analyses tell us? This seems to mirror the univariate heritability results. These results can probably be moved to the supplement.

(5) Could the authors say a bit more on the CNS versus GI tissue specfic expression? Given the GWAS-by-subtraction approach employed, the authors might have considered metaXcan, as that could have straightforwardly been applied to the AN/BE-specific results to clarify how much of these GI-related enrichment remained.this applies to the stratified LDSC too. This will strengthen the authors' argument about AN as a metabo-psychiatric disorder...

LESS SERIOUS CONCERNS

(6) Some interesting results could be better explored. For example, the BE-AN difference from AN in terms of BE-BROAD PRS is interesting - is that a hint of impulsivitiy specific effects? Similarly, could you clarify "However, there was a significant positive SNP-rg between ADHD and non-BMI AN, suggesting that opposing rg of AN and ADHD with BMI were previously masking this association"?

(7) The female-only analysis doesn't really add much. Did the authors perform a male-only analysis? Of course, we wouldn't expect to have statistical power for discovery, but it would be good to see the effect sizes for the top loci, and a male-female directional concordance (sign) test.

MINOR CONCERNS

(8) It is odd for an abstract to announce planned future work "incorporat[ing] multiple diagnoses and global ancestries". Consider reframing this as a limitation.

(9) The number of phenotypes/acronyms is excessive given that the primary emphasis is on "BE-BROAD", and detracts from clarity of takeaway. Given that these are highly correlated phenotypes, this can be more neatly presented as sensitivity analyses.

(10) please make clearer which summary statistics will be made freely available versus only to "bona fide researchers".

Version 1:

Reviewer comments:

Reviewer #2

(Remarks to the Author)
I appreciate the authors' responses and the clarifications about the makeup of the sample(s). I believe this has improved the manuscript and I have no further comments.

Reviewer #3

(Remarks to the Author)
The authors have been thoughtful and thorough in responding to the reviewers' critiques. We appreciate the detailed

documentation of how and where the manuscript has been revised. Overall, this is a meticulously documented and methodologically advanced study that distinctly moves the ED genetics literature forward.

Open Access This Peer Review File is licensed under a Creative Commons Attribution 4.0 International License, which permits use, sharing, adaptation, distribution and reproduction in any medium or format, as long as you give appropriate credit to the original author(s) and the source, provide a link to the Creative Commons license, and indicate if changes were made.
In cases where reviewers are anonymous, credit should be given to 'Anonymous Referee' and the source.
The images or other third party material in this Peer Review File are included in the article's Creative Commons license, unless indicated otherwise in a credit line to the material. If material is not included in the article's Creative Commons license and your intended use is not permitted by statutory regulation or exceeds the permitted use, you will need to obtain permission directly from the copyright holder.
To view a copy of this license, visit https://creativecommons.org/licenses/by/4.0/

<u>Response to Reviewers for *"Genome-wide association studies of binge-eating behaviour and anorexia nervosa yield insights into the unique and shared biology of eating disorder phenotypes"*, Termorshuizen, Davies, Lee et al.</u>

<u>Editorial points</u>

- Please fill out the STROBE checklist providing text excerpts from the manuscript for each point.

> We now provide this checklist, as well as the relevant checklist for Mendelian randomisation studies. Where the relevant text is succinct, we have provided it, otherwise we indicate that the entire referenced section addresses the checklist item.

- Please add the flowchart as the main figure reporting numbers of individuals at each stage of study – i.e. numbers potentially eligible, examined for eligibility, confirmed eligible, included in the study, completing follow-up, and analyzed.

> Due to the number of studies included in the analysis, we have not included this information as a main figure. However, we supply information on initial and final participant numbers within Supplementary Table 1.

- Please indicate the study design (cross-sectional, case-control or cohort) in the Abstract or title according to the STROBE requirements.

> We have amended the abstract accordingly
>
> Page 10, lines 4-5: *We conducted the first genomic meta-analysis of case-control studies of binge-eating behaviour* […]

- Please add a Table describing all demographic characteristics of the sample (sex/gender, age, ethnicity etc.) to the main text.

> Information on genetic ancestry (the relationship of this to ethnicity is expanded on in the revision below) and on genetically-determined sex is provided in Supplementary Table 1. We have added to the main text to point the reader to this information. As age cannot confound a direction of effect from genetic variants (present at conception) to lifetime diagnoses, we did not control for age in this study, and so do not report participant ages.
>
> Page 12, lines 6-7: *Genetic ancestry and genetically derived sex of participants is in Supplementary Table 1.*
>
> Page 41, lines 25-26: *Details on genetic ancestry definition are included in the Supplementary Methods.*

- To have more information on how representative the study sample is, please include data on study participants' ethnicity in Methods and Table describing demographic data. If ethnicity is not reported, the reason should be stated in

Methods. The Methods section should include an explanation of who identified participant ethnicity and the source of the classifications used (e.g. self-report or selection, investigator, database, electronic health record, survey instrument). Specific ethnic categories are preferred over collective terms, when possible. Categories included in groups labeled as “other” should be defined.

Due to space constraints, we have extended the discussion of genetic ancestry in the Supplementary Methods to describe its relationship to ethnicity in this study.

Page 41, lines 25-26: *Details on genetic ancestry definition are included in the Supplementary Methods.*

Supplementary Material, Page 33: *We then calculated principal components (PCs) on the set of pruned SNPs using reference data from the 1000 Genomes Project [33], which we limited to variants with genotyping call rate>99%, minor allele frequency (MAF)>0.01, and HWE deviations of P>10-3. For each cohort, we then plotted the PCs to visualise the largest homogenous cluster. This clustering indicates approximately shared genetic ancestry. Individuals outside of this largest cluster were typically in the minority and were excluded from further analysis. For studies included in the main analysis, the participants in the largest cluster self-identified as from European ethnic groups and were labelled as being of European genetic ancestry. For the Korean and Japanese studies used in the follow-up analyses, the participants in the largest cluster self-identified as from East Asian ethnic groups and so were labelled as being of East Asian genetic ancestry.*

- Please provide details on which datasets used in the current study were publicly available and which not; for the latter please explain in the Methods section how the access was obtained and provide details on permission to use the data. Please also add to the Methods whether a written/signed informed consent was obtained from participants.

We have added data access information to Supplementary Table 1 and have amended the Methods accordingly.

Page 23, line 5: *Data access is described in Supplementary Table 1.*

Page 41, lines 5-6: *Data access information is included in Supplementary Table 1.*

We have added to the Ethics statement in the Methods section to direct the reader to information on informed consent, as below. We have amended the Supplementary Note to ensure all study descriptions include an explicit statement of consent (not shown below).

Page 41, lines 3-5: *This work is a secondary analysis of data from individual studies. We provide ethics and statements of informed consent (or exclusion*

from informed consent) and Institutional Review Board approval for each study in the Supplementary Note.

*** Include a “Response to referees” document detailing, point-by-point, how you addressed each referee comment. If no action was taken to address a point, you must provide a compelling argument. This response will be sent back to the referees along with the revised manuscript.**

This is the requested document.

*** Please highlight all changes in the manuscript text file providing an annotated version with tracked changes as well as non-annotated text.**

We have provided these documents

*** If you have not done so already please begin to revise your manuscript so that it conforms to our policy and format instructions here: https://www.nature.com/natmentalhealth/submission-guidelines/aip-and-formatting**
Refer also to any guidelines provided in this letter.

We have revised this accordingly. Please note that this has resulted in a considerable amount of the main text and methods being moved to the Supplement or being paraphrased to meet word limits.

*** Include a revised version of any required reporting checklist. It will be available to referees (and, potentially, statisticians) to aid in their evaluation if the manuscript goes back for peer review. A revised checklist is essential for re-review of the paper.**

We have revised the original editorial policy and reporting summary documents – please revert to us if alternative checklists are required.

When submitting the revised version of your manuscript, please pay close attention to our href="https://www.nature.com/nature-research/editorial-policies/image-integrity">Digital Image Integrity Guidelines.

We have reviewed these guidelines – our figures are compliant.

Finally, please ensure that you retain unprocessed data and metadata files after publication, ideally archiving data in perpetuity, as these may be requested during the peer review and production process or after publication if any issues arise.

We have retained the data that underlies the analyses reported in this manuscript.

Reviewers' Comments:

<u>Reviewer #1 (Remarks to the Author):</u>

This substantially revised manuscript addresses all of the concerns that I expressed in my initial review.

> We thank the reviewer for their comments.

One typo: Line 659: "casual mixture modeling"

> Thank you for noting this typo – it has been corrected to "causal" (as have the three instances of the same typo in the Supplement…)

<u>Reviewer #2 (Remarks to the Author):</u>

This is a revised manuscript describing, in impressive detail, a GWAS of individuals with anorexia nervosa, bulimia nervosa, and binge-eating disorder. The authors have been very responsive to the prior review and I believe the manuscript has been significantly improved. The focus on binge eating is clearer and the findings are interesting and important.

> We are pleased the reviewer feels our responses to their previous comments have improved the work. We hope the further revisions below will make the manuscript clearer for readers.

I am afraid, however, that I still have trouble understanding how some of the groups were created (I apologize if I'm missing something obvious, but other readers may also have trouble following.).

More specifically, I am confused about the handling of individuals with anorexia nervosa, binge eating/purging subtype. In the authors' response to reviewers, they state,
"'Case' individuals for the BE-BROAD analysis comprise all individuals who report binge-eating behaviours. This includes individuals with the binge-eating subtype of anorexia nervosa…"
But this is not clear in Table 1; the diagnostic codes in the last column for BE-BROAD do not appear to include any for anorexia nervosa--more specifically, F50.02. This should be clarified.

> We apologise for the lack of clarity here. Per Table 1, participants are included as cases for BE-BROAD if they have evidence of binge-eating, which is operationalised by satisfying at least one of three criteria. One criterion is a clinical diagnosis of bulimia nervosa or binge-eating disorder (as indicated by the ICD codes), where binge eating is a diagnostic requirement. As the reviewer notes below, binge eating is not a diagnostic requirement for AN-BP, as individuals can receive this diagnosis in the absence of binge eating if they show purging behaviours. As such, F50.02 is not included. However, many individuals with AN-BP in our analysis also meet criteria for BE-BROAD, because they explicitly endorse binge-eating (as operationalised by the other two criteria).

We have amended the Methods and the legend for Table 1 to clarify this:

Page 35, Table 1 Legend: *Phenotype definitions. Diagnostic codes are shown with coding system in round brackets, and equivalent codes in square brackets. Note that diagnostic codes relate to "diagnosis" criteria, and that some criteria for BE do not require a diagnosis. Control definitions are shown in order of preference.*

Page 41, lines 10-16: *Broadly, we identified cases and controls based on clinical diagnoses, diagnostic algorithms, and/or self-report questionnaires[42]. AN (and its subtypes) is a diagnostic phenotype, requiring case participants to meet clinical or research diagnostic definitions. BE-BROAD is a symptom phenotype: cases either explicitly endorsed BE as a symptom or had a diagnosis requiring BE (specifically bulimia nervosa or binge-eating disorder; Table 1). The binge eating/purging subtype of AN can be diagnosed without BE, and so is not sufficient for inclusion as a BE-BROAD case.*

This also raises a question about the interesting analyses comparing BE-BROAD to anorexia nervosa. At least superficially, it seems that individuals with the binge/purge subtype could be included in either (or both??) groups. Was the BE-BROAD group compared only to individuals with the restricting subtype? I think these also need to be clarified.

While we agree that the comparison of BE-BROAD and AN-R is scientifically very interesting, we did not do this comparison, because the AN-R GWAS analysis is underpowered. This is apparent from its notably lower heritability Z-score compared to our other phenotypes, and its notably higher attenuation ratio. However, we were conscious of the potential biases that the overlap between BE-BROAD and AN could produce. It was for this reason that we conducted the sensitivity analysis removing cohorts ascertained for AN from the BE-BROAD analysis (referred to as BE-BROAD-NAAN). We draw the reviewer's attention to the sections reproduced below, and to the relevant sections of the Supplementary Material.

Page 15, Lines 9-12: *Because 18% of BE-BROAD cases had (known) AN (and 30% of AN cases met criteria for BE-BROAD), BE-BROAD results may include AN-related effects (Supplementary Table 11). To test this, we repeated the BE-BROAD GWAS, excluding studies that focused on AN recruitment (Supplementary Results).*

Page 46, line 5-6: *As a sensitivity analysis, we restricted BE-BROAD to studies that were not ascertained for AN, reasoning that this might better capture BE behaviour outside of AN.*

Page 51, lines 2-5: *A considerable portion (13%) of BE-BROAD cases were recruited through studies ascertained for AN. To understand the influence of AN on this BE-BROAD phenotype, we conducted an additional BE-BROAD meta-analysis excluded studies ascertained for AN (Supplementary Table 11).*

Related to these issues, the acronym AN-BE is used on page 14 without a definition.

Thank you for noting this – it was a typo and has been corrected to “AN”.

I would also point out that not all individuals with the binge/purge subtype of anorexia nervosa engage in binge eating; some purge after episodes of eating that are not binges. This is a minor point but the authors might wish to mention.

Indeed – this is important, and feeds into our response to the reviewer’s first point. We have also tried to make this clear to the reader in the Introduction (within the bounds of word limit) and in the Supplementary Material.

Page 11, line 5: *binge eating/purging (AN-BP), featuring binge eating (BE) and/or purging behaviours.*

Supplementary Material, Page 40: *some individuals with AN-BP may never have had a binge-eating episode but are classified for AN-BP due to the presence of purging behaviour.*

We thank the reviewer for all of their points raised.

<u>Reviewer #3 (Remarks to the Author):</u>

This is a new genome-wide association study (GWAS) release from the Psychiatric Genomics Consortium (PGC) Eating Disorder (ED) Working Group, with 56,344 unique cases and over 1.2M controls. This represents a major expansion over prior PGC GWAS installments. A major focus of the paper is the parallel analyses of binge eating disorder (BE) and anorexia nervosa (AN) including varying narrow/broader phenotypic definitions and sensitivity analyses. Beyond simple locus discovery, the authors have performed detailed and near-comprehensive characterizations of each trait's genetic architecture and shared genetic basis with a range of traits, including modeling eating disorder specific genetic variance not shared with body mass index (BMI).

We thank the reviewer for providing a new review of the work, for their kind comments here, and for their helpful points below.

MAIN CONCERNS

(1) The authors make an important point in the introduction, namely that standard DSM and ICD based phenotypic definitions "often exist on a continuum, overlap, co-occur, present heterogeneously, and morph over time". This idea could be better explored in the context of the current study, as it applies to the non-negligible overlap between BE-BROAD (18%) and AN

cases (30%). It would ease readability and interpretation if this overlap was stated in the main text; I finally tracked it down in Supplementary Table 11. Similarly, it is relevant for presentation of the MiXeR results.

Thank you for raising this point. We have expanded the relevant section of the main text to include these percentages as below:

Page 15, lines 9-12: *Because 18% of BE-BROAD cases had (known) AN (and 30% of AN cases met criteria for BE-BROAD), BE-BROAD results may include AN-related effects (Supplementary Table 11). To test this, we repeated the BE-BROAD GWAS, excluding studies that focused on AN recruitment (Supplementary Results).*

(2) The abstract refers to "the first genomic meta-analysis of binge-eating behaviour" and in the introduction, "we present the first GWAS of BE across multiple cohorts", and in the discussion "first genome-wide analysis of BE"--is this true, strictly speaking? There is some passing mention of model-derived BE phenotype in the Million Veteran Program. Do we mean "diagnosed BE", specifically?

Our analysis is of binge-eating behaviour as a symptom of multiple eating disorders, which has not been previously examined. The only previous GWAS analysis in this area was the study in MVP, which was a model-derived phenotype attempting to capture binge-eating disorder, which is a distinct phenotype with features beyond the symptom of binge eating alone, and which doesn't include other disorders featuring binge-eating (bulimia nervosa, the binging/purging subtype of anorexia nervosa) or binge-eating in the absence of disorder. We have clarified this in the text:

Pages 11-12, lines 27-1: *We present the first GWAS of the transdiagnostic symptom of BE behaviour*

Page 12, lines 10-12: *We contrast BE-BROAD with GWAS of a model-derived proxy binge-eating disorder phenotype from the Million Veteran Program[9] (Supplementary Material).*

Supplementary Material, Page 67: *Our BE-BROAD definition captures binge eating across multiple eating disorders (and in the absence of a diagnosed disorder), whereas the phenotype from Burstein et al sought to approximate a specific eating disorder (binge eating disorder), which has characteristics beyond the symptom of binge eating alone.*

(3) The presentation of directions of effect and significance levels for SNP-rg comparisons of BE-BROAD and AN with "other traits" should be improved -- please give the point estimates (or point to their location) in text, and make clearer which P-values correspond to which comparisons. We are asking for this because the results are quite interesting and warrant more detailed discussion.

We deliberately chose not to include numerous point estimates in the text to improve readability. We point the reader to the relevant Table and Supplementary Tables, which include all point estimates, standard errors, and P-values. This is included at the start of each relevant paragraph – we do not repeat this information to retain readability (and to reduce word count).

(4) Given the emphasis on the MiXeR analysis, the interpretation could be clearer, e.g. the minimum possible overlap model. Similarly, what do the genetic distance analyses tell us? This seems to mirror the univariate heritability results. These results can probably be moved to the supplement.

We agree the relevance of this finding was unclear, and we have expanded on the genetic distance analysis results in the Discussion. We have expanded the discussion of the minimum possible overlap model in the Supplementary Material and point the reader to this in the main text.

Page 21, lines 18-20: *Individuals with AN were as genetically distant from individuals with BE as they were from unaffected individuals, emphasising the distinct nature of these eating disorder-related phenotypes.*

Page 13, lines 19-23: *Bivariate MiXeR yielded similar SNP-rg (0.49, SE=0.01), and indicated 2654 overlapping causal variants (83% of all BE-BROAD causal variants) with highly concordant effects (95%; Figure 2a). This was not distinguishable from the minimum plausible overlap given the genetic correlation observed, an overlap of >2450 fully concordant causal variants (Supplementary Results, Supplementary Table 8).*

Supplementary Material, Page 50: *Bivariate MiXeR analyses should be viewed with caution in this instance. MiXeR compares the best overlap model (fit by optimised weighted log likelihood) with models for the extremes of plausible overlap. First, it compares to a maximal model, where all causal variants estimated for the first trait overlap with those estimated for the second trait (where the first trait has the fewer causal variants estimated). Second, it compares to the minimal model, which is the least overlap of causal variants possible given the observed genetic correlation. In our analyses, the low polygenicity of BE-BROAD restricts the modelling space for the MiXeR model. If one assumes that all causal variants that are shared are concordant in their direction of effect, then >75% of the putative causal variants for BE-BROAD have to be shared with AN. As a result, although the MiXeR best overlap model fit better than the maximal model, it did not fit better than the minimal model (Supplementary Table 8).*

(5) Could the authors say a bit more on the CNS versus GI tissue specfic expression? Given the GWAS-by-subtraction approach employed, the authors might have considered metaXcan, as that could have straightforwardly been applied to the AN/BE-specific results to clarify how much of these GI-related enrichment remained.this applies to the stratified LDSC too. This will strengthen the authors' argument about AN as a metabo-psychiatric disorder...

We highlighted experiment-wide significant tissue expression in the CNS and GI tissues from BE-BROAD and from AN because these are tissue groups of a priori interest. However, as noted in the Supplementary Material, the significant gene expression in these tissue types is consistent with the number of CNS and GI tissues tested. There is not an excess of gene expression in GI-related tissues (or in the CNS). As such, we have removed the focus on CNS and GI tissues.

Similarly, we did not see enrichment of these tissues in stratified LDSC analyses. As such (and given the large number of analyses already in the paper, and the limited word count), we do not think sensitivity analyses of these non-significant results are warranted.

LESS SERIOUS CONCERNS

(6) Some interesting results could be better explored. For example, the BE-AN difference from AN in terms of BE-BROAD PRS is interesting - is that a hint of impulsivitiy specific effects? Similarly, could you clarify "However, there was a significant positive SNP-rg between ADHD and non-BMI AN, suggesting that opposing rg of AN and ADHD with BMI were previously masking this association"?

Our subgroup PRS results show that BE-BROAD PRS predicts BE-BROAD both generally (when comparing BE-BROAD to controls), and in the context of AN (comparing BE+AN to AN alone). While we are pleased to observe this, we feel it is the expected finding. Given word limits, we have added this point to the Supplementary Materials.

Supplementary Material, Page 66: *Our results are consistent with BE-BROAD PRS predicting BE-BROAD both in comparison to controls and in the context of AN. Similarly, AN PRS is broadly able to distinguish individuals with AN from controls and in the context of broadly defined BE.*

We have tried to clarify the point about the effect of the shared genetic component between AN and BMI on the genetic correlation of AN with ADHD.

Page 20, lines 7-10: *BE-BROAD was positively genetically correlated with ADHD, whilst AN showed no significant association; however, this may result from an underlying positive SNP-rg between ADHD and the psychiatric component of AN being nullified by negative SNP-rg between ADHD and the component of AN shared with BMI.*

(7) The female-only analysis doesn't really add much. Did the authors perform a male-only analysis? Of course, we wouldn't expect to have statistical power for discovery, but it would be good to see the effect sizes for the top loci, and a male-female directional concordance (sign) test.

As noted by the reviewer, the male-only analysis is unfortunately too small to have meaningful power for analysis. We are actively working to increase sample sizes in males with eating disorders, and we hope to be able to

conduct these analyses more effectively in future work, which would also serve to increase the value of the female-only analyses. At present, the lack of substantial differences between the main analyses and the female-only analyses serve to demonstrate that the main findings cannot be explained by sex differences between our cases and controls.

MINOR CONCERNS

(8) It is odd for an abstract to announce planned future work "incorporat[ing] multiple diagnoses and global ancestries". Consider reframing this as a limitation.

Given the word limit of an abstract, we feel the existing phrasing allows us to acknowledge the implicit limitations of the current work, and to assert our intention to address these in future. A PubMed search for the phrase “Future research should” in abstracts identifies over 40,000 uses of this phrasing alone (https://pubmed.ncbi.nlm.nih.gov/?term=%22Future+research+should%22%5BTitle%2FAbstract%5D&sort=), and so this type of statement is not especially unusual.

(9) The number of phenotypes/acronyms is excessive given that the primary emphasis is on "BE-BROAD", and detracts from clarity of takeaway. Given that these are highly correlated phenotypes, this can be more neatly presented as sensitivity analyses.

We believe that the additional phenotypes (which are either primary [AN] or are sensitivity analyses) are importantly distinct from BE-BROAD and add meaningfully to the takeaway.

(10) please make clearer which summary statistics will be made freely available versus only to "bona fide researchers".

We have redrafted this statement to clarify as below:

Page 23, lines 5-13: *Data access is described in Supplementary Table 1. Available individual level genotype data (except in countries where sharing of individual level data is prohibited by national law) and summary statistics from individual studies used in this study are available to researchers via collaboration with study PIs or (for a subset of studies) via a secondary analysis proposal to the Eating Disorders Working Group of the PGC via (https://pgc.unc.edu/for-researchers/data-access-committee/data-access-information/).*

Summary statistics of all GWAS meta-analyses reported in this work will be made available via Figshare and the PGC website on publication, conditional on agreeing to terms and conditions of use (https://pgc.unc.edu/for-researchers/download-results/).

We thank the reviewer for all of their points raised.
